# Violence in adulthood amplifies the health correlates of childhood maltreatment

**DOI:** 10.1186/s12889-025-22469-x

**Published:** 2025-03-29

**Authors:** Rickard Pettersson, Mattias Strandh, Steven Lucas

**Affiliations:** 1 Department of Social Work, Umeå University, Umeå, SE-901 87 Sweden; 2 Department of Women’s and Children’s Health, Uppsala University, Uppsala, SE-751 85 Sweden

**Keywords:** Exposure to violence, Adverse childhood experiences, Poly-victimization, Revictimization, Life-course, Mental health, Physical health, Health-related risk behavior

## Abstract

**Background:**

Although experiences of violence are detrimental and may occur throughout the lifespan, few studies have examined the long-term health correlates of violence in both childhood and adulthood.

**Objective:**

To examine the association of exposure to child maltreatment (CM) as well as severe violence in adulthood with mental and physical health problems and health-related risk behaviors in adulthood.

**Methods:**

The study was cross-sectional and applied a novel survey instrument among a random sample of 10 337 Swedish women and men aged 18–74. Logistic regression was applied to calculate odds ratios.

**Results:**

Exposure to 0, 1, 2 or 3 or more types of CM showed graded associations for depression, anxiety, self-harm and PTSD in adulthood. Irritable bowel syndrome, fibromyalgia and obesity showed modest correlations. No significant associations were found between CM and ischemic heart disease (IHD), type 2 diabetes or cancer, although the ORs were in line with several previous ACE studies. When exposure to severe violence in adulthood was added to CM, odds ratios increased dramatically for mental health problems and health-related risk behaviors, suggesting that revictimization may moderate or mediate this relationship.

**Conclusions:**

The results underscore the importance of studying violence exposure in a life-course perspective and suggest that the relationship between childhood adversities and long-term physical health problems in adulthood may be affected by the traumatic effects of revictimization in adult life. This points to the importance of early identification of child maltreatment and provision of robust services to protect children, treat symtoms of trauma, and enhance resilience to decrease the risk of poor health outcomes.

**Supplementary Information:**

The online version contains supplementary material available at 10.1186/s12889-025-22469-x.

## Background

Globally, a substantial proportion of the world’s population has been exposed to some kind of interpersonal violence, which poses a major threat to public health [1, 2]. In a Swedish context, 46% of women and 38% of men have experienced severe sexual, physical and/or emotional violence during their lifetime [3]. The short-term effects of exposure to violence such as physical injuries or death are evident, but a growing body of evidence suggests that violence is also associated with poorer health in childhood and adulthood [4–6]. The Adverse Childhood Experiences study (ACE-study) created a surge in research on the long-term health consequences of violence, neglect and household dysfunction during childhood [7]. After more than two decades, this study remains the basis for hypotheses and inquiries into the mechanisms underlying the poorer health outcomes seen among those who experienced adversity in childhood in a dose-dependent manner.

Systematic reviews and meta analyses have shown strong associations between adversities in childhood and mental health problems and health-related risk behaviors later in life, but also associations to long-term physical health problems such as ischemic heart disease (IHD), type 2 diabetes and cancer [8–11]. Most of the reported studies were cross-sectional in design and relied on self-report data from adults or adolescents. A majority of the literature regarding ACEs has applied a score for the number of adverse experience types reported by the individuals, treating all childhood adversities equally. This has been questioned by some researchers, and several studies have reported that experiences of child maltreatment (physical, psychological and sexual violence, physical and psychological neglect) show higher odds of mental health problems later in life compared to the household dysfunction aspects of the ACE model (parental mental illness, substance abuse or incarceration) [12, 13]. In one study, exposure to intimate partner violence (IPV) between adults in the household showed odds ratios closer to the child maltreatment aspects than to household dysfunction [12]. Although the findings are significant, these studies have been based on small populations that in most cases have been non-random, and to our knowledge, the relative health associations of child maltreatment versus household dysfunction have not been examined in large national samples of both women and men.

Individuals exposed to one type of violence are at increased risk of other types of victimization [14, 15], and those who suffer from violence in childhood are disproportionately exposed to victimization in adult life [16, 17]. Consequently, in order to understand the relationship between exposure to violence in childhood and long-term health consequences it is important to take poly-victimization, revictimization and the lifetime burden of violence into account. To our knowledge, no studies in Sweden have examined experiences of violence in childhood as well as in adulthood and the long-term health correlates of cumulative exposure. One report showed that lifetime experiences of violence were linked to poor health among 60–85 year old women and men in Sweden, although experiences in childhood versus adulthood were not separated [18]. Another study among 60–84 year old women and men showed that those exposed to physical or psychological violence over the past 12 months had high odds of mental and physical health problems compared to the unexposed group, but no information was gathered about maltreatment during childhood [19]. 

Recently, a model was put forth to provide a theory-based framework to understand and act on the mechanisms linking traumatic experiences and poor health outcomes [20]. The Trauma-Informed Theory of Individual Health Behavior (TTB) posits that an individual’s ability to make decisions that enhance the likelihood of positive health outcomes following exposure to childhood trauma is based on: (1) the types and severity of trauma they have been exposed to; (2) how this trauma is manifested physiologically (i.e., the trauma response); and (3) factors enhancing resilience to undertake behavior change despite this trauma response. Accordingly, childhood experiences of maltreatment may negatively affect the ability of some individuals to make positive choices regarding health-related behaviors but also to avoid environments that increase the likelihood of violence later in life. Among the individuals who remain in a trauma-perpetuating environment, long-term negative health effects may be more pronounced as the lifetime burden of violence exposure increases. Research over the past decade has pointed to toxic stress as a likely biological mechanism by which trauma leads to ill health, through both neurodevelopmental and physiological effects including cardiovascular and immunological systems.

The present study was initiated to fill gaps in the current understanding of health correlations of exposure to child maltreatment, poly-victimization and revictimization in a lifetime perspective in a Swedish context. The aim was to examine the association of exposure to child maltreatment and severe violence exposure in adulthood, separately and in combination, with long-term health correlates measured as mental and physical health problems and health-related risk behaviors in adulthood.

## Methods

This is a cross-sectional study based on a survey distributed to a randomly selected population of women and men in Sweden. The data were collected in the spring of 2012 using a novel questionnaire based on questions from the original Adverse Childhood Experiences (ACE) study [7] and a previous national violence prevalence study in Sweden [21]. The first section concerned current sociodemographic information, followed by items regarding family conditions during childhood, including physical and emotional neglect as well as parental substance abuse, mental illness, suicide attempts and criminal behavior. Exposure to sexual, physical and psychological violence of varying severity was inquired about separately for ages 0 through 14, 15 through 17 and from 18 on. For exposure before the age of 18, identical but separate sections addressed violence perpetrated by adults and that perpetrated by peers for each type of violence. Other question sections included self-reported physical and mental health as well as health-related risk behaviors such as smoking and alcohol consumption (Table 1).

**Table 1:** Description of outcome variables, measures used and criteria for positivity

	Measure	Criterion for positivity
**Mental health problems**		
Depression	Hospital Anxiety and Depression Scale [25]	11 points or above, representing probable depression (maximum 21 points)
Anxiety	Hospital Anxiety and Depression Scale	11 points or above, representing probable anxiety (maximum 21 points)
Post-traumatic stress symptoms (PTSD)	PCL [26]	9 points (maximum 18 points)
Self-harm	Self report	Any positive reponse regarding physical self-harm, suicidal ideation or suicide attempts
Somatization	PHQ-15 [27]	10 points or above (maximum 24)
**Physical health problems**		
Irritable bowel syndrome (IBS)	Self report	Any positive response
Fibromyalgia	Self report	Any positive response
Ischemic heart disease (IHD)	Self report	Any positive response regarding heart attack, angina or heart failure
Chronic obstructive pulmonary disease (COPD)	Self report	Any positive response
Type 2 diabetes	Self report	Any positive response
Cancer	Self report	Any positive response
Obesity	Calculated BMI	BMI 30 or above
**Health-related risk behaviors**		
Heavy smoking	Self report	Smoking more than 20 cigarettes per day
Alcohol misuse	AUDIT (28)	8 points or above for men, 6 points or above for women
Drug abuse	Self report	Any positive response

Face and content validity of the questionnaire were evaluated by Statistics Sweden with the use of expert review and cognitive interviews with persons with and without a history of violence exposure. Following slight adjustments, the final Violence and Health survey instrument consisted of 97 questions with a total of over 300 sub-items [3, 22].

Questionnaires were distributed to 10 000 women and 10 000 men 18 to 74 years of age randomly selected from the population registry of Sweden. An opt-out strategy was applied with an introduction letter providing information about the study and that the individual could choose not to participate by contacting the research group or Statistics Sweden. Those who did not opt out received a subsequent letter with instructions about how to complete the survey online, and paper questionnaires were sent to those who did not respond online. A maximum of four reminders were sent. Respondents were informed that by completing the online or paper version of the questionnaire they gave their informed consent for their data to be handled as described above. Out of a total of 20 000 respondents, 10 337 individuals participated in the survey, giving a response rate of 52% (56% for women, 48% for men). An analysis of non-responders was performed taking sociodemographic factors and welfare-based variables into account, and a multi-factorial weighting algorithm was created to correct for underrepresented respondent groups [23]. The study was approved by the Ethical Review Board in Uppsala (Dnr 2011/156).

### Measures

A broad definition of interpersonal violence similar to that suggested by the World Health Organization was applied in this study, including physical, psychological and sexual violence throughout the lifespan as well as exposure to violence between parents and physical and psychological neglect in childhood [1]. The variables included in the analyses were dichotomous or categorical with mutually exclusive groups.

### Exposure variables

A categorical variable corresponding to the maltreatment dimensions of the ACE questionnaire was constructed for exposure to zero, one, two or three or more types of child maltreatment (sexual violence by an adult, physical or psychological violence by an adult in the home, physical neglect, psychological neglect, IPV between parents) before the age of 18. IPV between parents was included in the maltreatment variable instead of in the household dysfunction variable described below because it is increasingly recognized as a type of violence against children and has been criminalized in several countries, including Sweden [24].

Childhood maltreatment was operationalized as follows: Having been exposed to sexual abuse by an adult before age 18; Having sometimes/often been hit with a closed fist, hit with a weapon or other object or often subjected to other forms of physical violence by an adult in the home; Often having been threatened with physical violence, often humiliated or bullied by an adult in the home; having been exposed to physical neglect; having been exposed to psychological neglect; or repeatedly having seen or heard violence between one’s parents. For each maltreatment type, any response indicating exposure was counted as a positive outcome.

In order to assess how severe victimization in adulthood impacts the association of childhood victimization to health problems, a separate categorical variable was created that included the childhood maltreatment categories described above with or without exposure to severe violence in adulthood, resulting in eight mutually excusive categories (Table 2).

**Table 2:** Exposure to violence in a lifetime perspective in number and percent of women and men based on categorical variables for child maltreatment (CM) and CM with or without severe violence in adulthood

			*N* (%)	
Number of CM types	Severe violence in adulthood	Total	Women	Men
		Child maltreatment*		
0	n.a.	6043 (65.9)	3193 (63.4)	2850 (69.0)
1	n.a.	1589 (17.3)	911 (18.1)	678 (16.4)
2	n.a.	744 (8.1)	437 (8.7)	307 (7.4)
≥3	n.a.	793 (8.6)	498 (9.9)	295 (7.1)
		Child maltreatment + severe violence in adulthood*		
0	No	5038 (56.3)	2655 (53.9)	2383 (59.2)
1	No	1062 (11.9)	596 (12.1)	466 (11.6)
2	No	545 (5.1)	244 (5.0)	210 (5.2)
≥3	No	377 (4.2)	221 (4.5)	156 (3.9)
0	Yes	875 (9.8)	473 (9.6)	402 (10.0)
1	Yes	483 (5.4)	293 (5.9)	190 (4.7)
2	Yes	269 (3.0)	179 (3.6)	90 (2.2)
≥3	Yes	393 (4.4)	264 (5.4)	129 (3.2)

^*^Statistically significant differences were found between women and men for both categorical variables, *p* < 0.001

Severe violence in adulthood was operationalized as follows:

As an adult having been forced to have sexual intercourse or similar sexual acts (including attempts); Having been hit with a fist/object, kicked or subjected to violence with a weapon; Systematically and repeatedly subjected to psychological violence. Any response indicating severe violence exposure was counted as a positive outcome.

Aspects of household dysfunction corresponding to those in the ACE questionnaire were grouped into a single dichotomous variable for experiences of growing up in a household where parents had mental illness, suicidal behavior, alcohol abuse, use of street drugs, criminal behavior (incarcerated several times) or separation/divorce.

### Outcome variables

Current physical and psychological health was assessed using previously validated instruments [25–28], as well as by self-report (Table 1). These conditions were chosen due to their significant public health relevance and their use in previous ACE research.

### Background variables

The background variables included in the analyses were sex (male/female), predominant type of residence during childhood (owned home, rental), self-reported parental immigrant status (at least one parent born in a Nordic country/both parents born elsewhere) and parents’ highest level of education (at least one parent with high school education/both parents below high school), age (collected from registry data and categorized in groups aged 17–25/26–35/36–45/46–55/56–65/66–74) (Table 3).

**Table 3:** Distribution of household dysfunction and background variables among women and men in number and percent

		*N* (%)	
Variable	Total	Women	Men
Household dysfunction (yes) ^*^	2643 (26.2)	1543 (27.9)	1100 (24.2)
Sex	10 337 (100)	5681 (55.0)	4656 (45.0)
Residence type (rental)	2573 (26.2)	1427 (26.5)	1146 (26.0)
Parent immigrant status (yes)	768 (7.6)	424 (7.7)	344 (7.6)
Parental education (< high school) ^*^	4558 (48.9)	2429 (47.3)	2129 (50.8)
Age group^*^			
17–24	1206 (11.7)	714 (12.6)	492 (10.6)
25–34	1549 (15.0)	870 (15.3)	679 (14.6)
35–44	1880 (18.2)	1073 (18.9)	807 (17.3)
45–54	2011 (19.5)	1142 (20.1)	869 (18.7)
55–64	2151 (20.8)	1138 (20.0)	1014 (21.8)
65–74	1538 (14.9)	744 (13.1)	794 (17.1)

^*^Statistically significant differences between women and men, *p* < 0.001

### Data analysis

Weighted data were compared to non-weighted data with respect to the descriptive statistics for violence exposure and health outcomes. As any differences found were negligible, we chose to apply non-weighted data for all analyses. Pearson’s chi square was used to assess potential differences between women and men with respect to maltreatment/violence exposure, health problems and background variables. Associations between exposure to child maltreatment with or without violence in adulthood and specific measures of mental and physical health were assessed using logistic regression analyses. First, the child maltreatment exposure variable was used as the independent variable in a non-adjusted model with the health outcome variables as dependent. In an adjusted model, the household dysfunction variable and all background variables were then added to the logistic regression. Finally, the child maltreatment exposure variable was replaced with the variable including both childhood maltreatment and severe violence in adulthood as the independent variable in the adjusted model mentioned above. Results from the logistic regression analyses are presented as odds ratios (OR) with 95% confidence intervals (95% CI). Data were analyzed using SPSS version 28 (IBM Corp.). A significance level of *p* < 0.05 and two-tailed analyses were applied throughout.

## Results

Descriptive statistics for exposure to severe violence in a lifetime perspective are presented in Table 2. The largest groups of victims were those exposed to one type of child maltreatment. Women to a greater extent have been victimized to severe violence in a dose-dependent manner compared to men in a lifetime perspective, and exposure to severe violence in childhood is very often combined with exposure to severe violence in adulthood.

Descriptive statistics for respondent characteristics related to mental and physical health problems and health-related risk behaviors are presented in Table 4. Mental health problems were significantly more common among women compared to men, as were IBS, fibromyalgia and cancer. Men were significantly more likely than women to have IHD, type 2 diabetes, obesity and all three of the health-related risk behaviors.

**Table 4:** Respondent characteristics related to mental, physical and health-related risk behaviors

			*N* (%)	
**Mental health symptoms**		Total	Women	Men
Depression		859 (8.7)	490 (9.1)	369 (8.3)
Anxiety^***^		491 (5.0)	359 (6.6)	132 (3.0)
Post-traumatic stress symptoms (PTSD)^***^		689 (7.1)	480 (9.1)	209 (4.8)
Self-harm^***^		1 197 (11.7)	815 (14.5)	382 (8.3)
Somatization^***^		570 (6.1)	411 (8.0)	159 (3.7)
**Physical health symptoms**				
Irritable bowel syndrome (IBS) ^***^		470 (4.5)	344 (6.1)	126 (2.7)
Fibromyalgia^***^		225 (2.2)	195 (3.4)	30 (0.6)
Ischemic heart disease (IHD) ^***^		532 (5.1)	183 (3.2)	349 (7.5)
Chronic obstructive pulmonary disease (COPD)		184 (1.8)	105 (1.8)	79 (1.7)
Type 2 diabetes^***^		383 (3.7)	153 (2.7)	230 (4.9)
Cancer^**^		455 (4.4)	279 (4.9)	176 (3.8)
Obesity^*^		1 377 (13.6)	711 (12.8)	666 (14.6)
**Health-related risk behaviors**				
Heavy smoking^***^		423 (4.2)	158 (2.8)	265 (5.8)
Hazardous drinking^***^		1 853 (18.6)	920 (16.8)	933 (20.8)
Drug abuse^*^		55 (0.5)	22 (0.4)	33 (0.7)

Statistically significant differences between women and men: ^***^p < 0.001, ^**^p < 0.01, ^*^p < 0.05

In the non-adjusted logistic regression regarding exposure to CM, exposure to CM was strongly associated with all the mental health variables in a graded fashion (Table 5).

**Table 5:** Non-adjusted odds ratios of health correlates relative to exposure to 1, 2 or 3 + types of child maltreatment (CM) compared to no maltreatment exposure. Logistic regression using categorical variables. Numbers in parentheses indicate the upper and lower boundaries of the 95% confidence intervals

Number of CM types^1^	Depression	Anxiety	PTSD	Self-harm	Somatization
1	2.50^***^ (2.05, 3.03)	2.42^***^ (1.86, 3.13)	2.76^***^ (2.21, 3.45)	2.53^***^ (2.14, 3.00)	2.33^***^ (1.83, 2.95)
2	2.69^***^ (2.09, 3.45)	2.93^***^ (2.14, 4.03)	3.62^***^ (2.77, 4.75)	3.23^***^ (2.62, 3.98)	3.31^***^ (2.50, 4.39)
≥3	6.01^***^ (4.91, 7.37)	7.09^***^ (5.54, 9.09)	10.15^***^ (8.19, 12.57)	7.85^***^ (6.58, 9.37)	5.12^***^ (4.00, 6.54)
	IBS	Fibromyalgia	IHD	COPD	Diabetes Type II
1	1.24 (0.95, 1.62)	1.63^*^ (1.11, 2.41)	1.01 (0.78, 1.32)	1.67^*^ (1.11, 2.51)	0.88 (0.64, 1.21)
2	1.74^***^ (1.26, 2.40)	2.18^**^ (1.37, 3.48)	1.52^**^ (1.11, 2.07)	1.57 (0.90, 2.75)	1.32 (0.91, 1.92)
≥3	2.41^***^ (1.82, 3.20)	3.07^***^ (2.05, 4.59)	1.15 (0.82, 1.61)	3.01^***^ (1.96, 4.61)	1.16 (0.80, 1.70)
	Cancer	Obesity	Heavy smoking	Hazardous drinking	Drug abuse
1	0.98 (0.74, 1.30)	1.09 (0.92, 1.28)	1.61^***^ (1.22, 2.12)	1.53^***^ (1.33, 1.75)	1.43 (0.56, 3.66)
2	1.17 (0.82, 1.67)	1.47^***^ (1.20, 1.81)	2.70^***^ (1.98, 3.70)	1.42^***^ (1.17, 1.71)	3.06^*^ (1.20, 7.85)
≥3	1.16 (0.82, 1.64)	1.42^***^ (1.16, 1.74)	2.99^***^ (2.23, 4.02)	1.82^***^ (1.53, 2.17)	6.28^***^ (3.01, 13.10)

^1^Number of maltreatment types in childhood (physical abuse, sexual abuse, psychological abuse, physical neglect, psychological neglect, exposure to parental IPV). ^***^p < 0.001, ^**^p < 0.01, ^*^p < 0.05

Significant associations for IBS and fibromyalgia were found for nearly all CM levels. IHD, COPD and obesity showed inconsistent patterns with some significant correlations but no clear connection to the level of CM exposure. No significant relationships were found between any exposure level of CM and reports of cancer or type 2 diabetes. Exposure to CM was associated with all of the health-related risk behaviors.

In the adjusted model, only slight changes in ORs were seen for most health outcomes (Table 6). Tables showing the complete results of the adjusted regression analyses are included as supplementary material, only the results regarding the exposure variables are presented here for the sake of brevity. Household dysfuction was significantly associated with PTSD, self-harm, heavy smoking and hazardous drinking, with ORs (95% CI) between 1.20 (1.01, 1.42) and 1.44 (1.25, 1.65). Of the background variables, only sex and age were consistently associated with most health outcomes.

**Table 6:** Adjusted odds ratios^1^ of health correlates relative to exposure to 0, 1, 2 or ≥3 types of child maltreatment (CM). Logistic regression using categorical variables. Numbers in parentheses indicate the upper and lower boundaries of the 95% confidence intervals

Number of CM types^2^	Depression	Anxiety	PTSD	Self-harm	Somatization
0	1.0	1.0	1.0	1.0	1.0
1	2.49^***^ (2.00, 3.10)	2.36^***^ (1.77, 3.16)	2.58^***^ (2.00, 3.33)	2.64^***^ (2.18, 3.21)	2.16^***^ (1.65, 2.83)
2	2.45^***^ (1.81, 3.30)	2.89^***^ (2.01, 4.17)	3.65^***^ (2.68, 4.98)	3.37^***^ (2.61, 4.35)	3.06^***^ (2.22, 4.22)
≥3	6.23^***^ (4.82, 8.05)	6.24^***^ (4.53, 8.61)	7.64^***^ (5.79, 10.08)	8.86^***^ (7.02, 11.19)	4.46^***^ (3.28, 6.08)
	IBS	Fibromyalgia	IHD	COPD	Diabetes Type II
0	1.0	1.0	1.0	1.0	1.0
1	1.07 (0.79, 1.46)	1.44 (0.94, 2.21)	0.85 (0.61, 1.19)	1.77^*^ (1.10, 2.84)	1.00 (0.70, 1.43)
2	1.80^***^ (1.27, 2.56)	1.67 (0.99, 2.82)	1.47^*^ (1.02, 2.13)	1.29 (0.65, 2.56)	1.25 (0.81, 1.93)
≥3	2.18^***^ (1.55, 3.07)	2.09^*^ (1.26, 3.49)	1.33 (0.88, 2.01)	3.06^***^ (1.77, 5.30)	0.98 (0.59, 1.63)
	Cancer	Obesity	Heavy smoking	Hazardous drinking	Drug abuse
0	1.0	1.0	1.0	1.0	1.0
1	0.91 (0.66, 1.25)	1.12 (0.93, 1.35)	1.64^*^ (1.18, 2.26)	1.64^***^ (1.40, 1.92)	1.09 (0.35, 3.38)
2	0.97 (0.64, 1.47)	1.40^*^ (1.10, 1.78)	2.68^***^ (1.86, 3.86)	1.55^***^ (1.24, 1.95)	2.83 (0.97, 8.28)
≥3	1.21 (0.81, 1.82)	1.37^*^ (1.06, 1.76)	2.73^***^ (1.88, 3.95)	1.95^***^ (1.56, 2.43)	3.44^*^ (1.24, 9.50)

^1^All analyses were adjusted for exposure to household dysfunction (yes/no), respondent’s sex (male/female), predominant type of residence during childhood (owned home/rental), parents’ level of education (at least one parent with high school or above/both parents below high school), parents’ country of birth (at least one parent born in the Nordic countries/both parents born elsewhere), and age group in years at response (17–25/26–35/36–45/46–55/56–65/66–74). ^2^Number of maltreatment types in childhood (physical abuse, sexual abuse, psychological abuse, physical neglect, psychological neglect, exposure to parental IPV). ^***^p < 0.001, ^**^p < 0.01, ^*^p < 0.05

When severe violence was added to the adjusted regression model, a clear shift was seen toward higher odds for all mental health outcomes as well as the health-related risk behaviors compared to the groups who had experienced CM but not adulthood violence (Table 7). No such increase was found for the physical health problems, although the most exposed group did show significantly higher odds of cancer.

**Table 7:** Adjusted odds ratios^1^ of health correlates relative to exposure to 0, 1, 2 or ≥3 types of childhood maltreatment (CM) alone or in combination with exposure to violence in adulthood (AH). Logistic regression using categorical variables. Numbers in parentheses indicate the upper and lower boundaries of the 95% confidence intervals

Number of CM types^2^	Depression	Anxiety	PTSD	Self-harm	Somatization
0	1.0	1.0	1.0	1.0	1.0
1	2.35^***^ (1.78, 3.11)	2.24^***^ (1.51, 3.33)	2.46^***^ (1.73, 3.50)	2.26^***^ (1.75, 2.94)	1.98^***^ (1.39, 2.83)
2	2.32^***^ (1.55, 3.47)	2.72^***^ (1.60, 4.64)	3.08^***^ (1.94, 4.89)	3.08^***^ (2.16, 4.40)	2.31^***^ (1.43, 3.73)
≥3	4.86^***^ (3.35, 7.05)	4.72^***^ (2.85, 7.82)	5.87^***^ (3.82, 9.01)	5.7^***^ (3.98, 8.08)	3.80^***^ (2.42, 5.98)
0 + AH	2.12^***^ (1.57, 2.86)	3.31^***^ (2.29, 4.79)	3.97^***^ (2.87, 5.50)	2.85^***^ (2.21, 3.66)	2.62^***^ (1.84, 3.73)
1 + AH	3.98^***^ (2.88, 5.50)	4.98^***^ (3.32, 7.47)	6.31^***^ (4.43, 8.98)	5.93^***^ (4.50, 7.82)	4.27^***^ (2.91, 6.27)
2 + AH	3.53^***^ (2.31, 5.41)	5.37^***^ (3.28, 8.80)	8.59^***^ (5.70, 12.94)	6.12^***^ (4.29, 8.74)	6.53^***^ (4.32, 9.87)
≥3 + AH	9.60^***^ (6.97, 13.21)	12.02^***^ (8.14, 17.74)	16.34^***^ (11.59, 23.06)	17.4^***^ (12.98, 23.34)	7.84^***^ (5.35, 11.50)
	IBS	Fibromyalgia	IHD	COPD	Diabetes Type II
0	1.0	1.0	1.0	1.0	1.0
1	1.04 (0.70, 1.53)	1.39 (0.81, 2.38)	0.85 (0.58, 1.25)	1.77 (1.03, 3.03)	0.96 (0.63, 1.46)
2	1.63^*^ (1.0, 2.63)	1.83^*^ (0.94, 3.56)	1.48 (0.95, 2.30)	0.58 (0.18, 1.87)	0.92 (0.51, 1.67)
≥3	1.95^**^ (1.18, 3.21)	2.17^**^ (1.07, 4.43)	1.34 (0.79, 2.26)	2.59^*^ (1.26, 5.33)	1.01 (0.52, 1.94)
0 + AH	1.54^*^ (1.07, 2.22)	2.07 (1.23, 3.50)	1.05 (0.69, 1.62)	1.02 (0.46, 2.27)	1.37 (0.87, 2.15)
1 + AH	1.55 (0.98, 2.46)	2.18^*^ (1.14, 4.18)	0.75 (0.39, 1.46)	1.82 (0.81, 4.11)	1.25 (0.67, 2.31)
2 + AH	2.64^***^ (1.6, 4.26)	2.13 (0.99, 4.61)	1.51 (0.81, 2.83)	2.74^*^ (1.20, 6.24)	2.05^*^ (1.09, 3.85)
≥3 + AH	2.53^***^ (1.63, 3.94)	2.50^**^ (1.28, 4.91)	1.29 (0.69, 2.43)	3.85^***^ (1.89, 7.83)	0.94 (0.42, 2.09)
	Cancer	Obesity	Heavy smoking	Hazardous drinking	Drug abuse
0	1.0	1.0	1.0	1.0	1.0
1	0.99 (0.69, 1.43)	1.14 (0.91, 1.42)	1.80^**^ (1.24, 2.62)	1.46^***^ (1.20, 1.79)	0.78 (0.17, 3.56)
2	0.84 (0.49, 1.46)	1.51^**^ (1.12, 2.04)	1.72^*^ (1.01, 2.90)	1.40^*^ (1.04, 1.90)	0.88 (0.11, 6.99)
≥3	0.85 (0.46, 1.58)	1.34 (0.94, 1.90)	1.85^*^ (1.07, 3.19)	1.58^*^ (1.13, 2.20)	1.00 (0.12, 8.11)
0 + AH	1.14 (0.77, 1.71)	1.23 (0.97, 1.55)	1.53 (0.97, 2.41)	1.93^***^ (1.59, 2.35)	1.00 (0.22, 4.50)
1 + AH	0.79 (0.43, 1.45)	1.20 (0.88, 1.64)	1.50 (0.84, 2.68)	2.75^***^ (2.15, 3.50)	1.69 (0.37, 7.82)
2 + AH	1.29 (0.69, 2.38)	1.36 (0.92, 2.01)	5.20^***^ (3.22, 8.40)	2.37^***^ (1.70, 3.30)	5.67^**^ (1.71, 18.83)
≥3 + AH	1.77^*^ (1.06, 2.94)	1.53^*^ (1.09, 2.14)	4.13^***^ (2.61, 6.54)	2.80^***^ (2.11, 3.71)	4.23^**^ (1.32, 13.55)

^1^All analyses were adjusted for exposure to household dysfunction (yes/no), respondent’s sex (male/female), predominant type of residence during childhood (owned home/rental), parents’ level of education (at least one parent with high school or above/both parents below high school), parents’ country of birth (at least one parent born in the Nordic countries/both parents born elsewhere), and age in years at response (17–25/26–35/36–45/46–55/56–65/66–74). ^2^Number of maltreatment types in childhood (physical abuse, sexual abuse, psychological abuse, physical neglect, psychological neglect, exposure to parental IPV). ^***^p < 0.001, ^**^p < 0.01, ^*^p < 0.05

## Discussion

This article has examined exposure to child maltreatment and severe violence in adulthood to study their respective contributions to associations with many of the major public health problems in Sweden. The results demonstrated that polyvictimization in childhood was clearly associated with all of the analyzed mental health outcomes and health-related risk behaviours in a graded fashion similar to previous findings from ACE studies in other countries, while the physical health problems studied did not demonstrate such associations. Revictimization in adulthood was associated with significant increases in the odds of mental health problems and risk behaviors compared to CM alone. As demonstrated in previous studies, women were the victims of violence in a life-course perspective to a greater extent than men [2].

Overall, the graded associations found between exposure to CM and several of the mental health problems showed higher ORs than previously reported in studies using the ACE questionnaire [29]. For example, our findings regarding the association between exposure to three types of CM and reports of anxiety, self-harm and PTSD in adulthood showed odds ratios that were twice to three times as high as previous studies from the US using four or more ACE types as the cutoff [8]. Several factors might underlie these differences. Our exposure variables included only aspects of child maltreatment, whereas the ACE scale includes other types of adversities such as parental divorce that may not be as clearly associated with mental health problems. Our results regarding the household dysfunction variable, which showed relatively small ORs to only a few of the health outcomes lends support to this notion. Studies have indicated that the prevalence of mental health problems in the total population is similar in the US, UK and Sweden, making it unlikely that this would explain these differences [30]. However, the relatively small proportion of respondents with three types of CM in the present analyses may represent a particularly vulnerable group with respect to psychosocial factors that increase the risk of mental health problems. This needs to be further investigated.

Our findings of modest correlations with the IBS and fibromyalgia are in agreement with findings from previous ACE studies regarding IBS and chronic pain [31, 32]. With regard to IHD and type 2 diabetes, we found no significant associations, although the ORs demonstrated were in line with several previous ACE studies. The literature is somewhat inconsistent regarding these disease outcomes, with most studies reporting similar ORs of which some are statistically significant while others are not [8, 11]. Our results regarding obesity are in agreement with an ACE study that limited the exposure variables to violence or severe violence during childhood [33]. Rates of IHD, type 2 diabetes and obesity are lower in Sweden than in the US and the United Kingdom, where a majority of the ACE studies have been conducted [34–36]. As regression analyses are sensitive to group sizes, this may have been a contributing factor to the lack of significant correlations to these health outcomes in our material. To our knowledge this has not been studied and presents an interesting avenue for further research.

According to TTB, individuals exposed to violence who have or are able to develop resilience factors including safety, autonomy, trauma awareness and trust are more likely to have positive coping strategies and reduced risk of remaining in trauma-perpetuating environments [20]. Against this background, our findings that individuals who had experienced CM but who were not exposed to severe violence in adulthood were less at risk of many of the health problems studied here than those who were revictimized as adults are intriguing. This may represent a more resilient group compared to those exposed to both CM and adult violence. In this respect, severe violence in adulthood may act as a moderator or mediator of the health consequences of CM.

### Strengths and limitations

A strength of this study is that it is based on a large sample of both women and men in Sweden. Furthermore, the risk of non-response bias has been decreased by careful analysis of the attrition and subsequent calibration and weighting of data, based on sets of information from national registries. Another strength is that we were able to break down data regarding victimization with respect to experiences in childhood and adulthood. This provided the opportunity to analyze associations between health problems and health-related risk behaviors and exposure to CM alone or together with victimization in adulthood.

Limitations of this study include the retrospective nature of the data, which may entail recall bias. Although we tried to reach a representative sample of the population, certain groups, particularly those who are hard to reach due to homelessness, severe illness or incarceration, are underrepresented. This may have affected the results, as these groups are known to have higher levels of violence exposure and poorer health. Another limitation is the relatively broad measures of victimization used to assess severe violence, without detailed information regarding the circumstances, frequency or duration of each type of severe violence exposure. For example, we cannot discern to what extent the results apply to specific subgroups, such as children repeatedly victimized at an early age or adults occasionally victimized many years ago. Also, the survey was carried out in 2012, and changes in violence exposure and health problems may have changed somewhat during the years that have passed since the data were collected. As in all survey studies, the present results are limited by the cross-sectional nature of the data, with fixed responses to complicated issues. Another limitation is that it is not possible to determine whether the health problems and health-related risk behaviors were present before or after the initial event of violence exposure. This is a major limitation in most cross-sectional studies and must be taken into account when interpreting the results.

It is important to note that the main outcome variables, which divide the respondents into mutually exclusive groups with respect to exposure to one, two or three or more types of CM with or without violence exposure in adulthood, may not adequately represent the complex ecological reality of abuse.

## Conclusion

The results underscore the importance of studying violence exposure in a life-course perspective and suggest that the relationship between childhood adversities and long-term physical health problems in adulthood is likely affected by the traumatic effects of revictimization in adult life. This points to the importance of early identification of child maltreatment and provision of robust services to protect children, treat symtoms of trauma, and enhance resilience to decrease the risk of poor health outcomes.

## Electronic supplementary material

Below is the link to the electronic supplementary material.

Supplementary Material 1

## Data Availability

The data that supports the findings of this study are available from The National Centre for Knowledge on Men’s Violence Against Women, Uppsala University but restrictions apply to the availability of these data, which were used under licence for the current study, and so are not publicly available.

## References

[1] Krug EG, Mercy JA, Dahlberg LL, Zwi AB. The world report on violence and health. Lancet. 2002;360(9339):1083–8. 12384003 10.1016/S0140-6736(02)11133-0

[2] World Health Organization. Global status report on violence prevention 2014 [Internet]. Geneva: World Health Organization. 2014 [cited 2024 Oct 5]. 274 p. Available from: https://iris.who.int/handle/10665/145086

[3] Andersson T, Heimer G, Lucas S. Violence and health in Sweden: a National prevalence study on exposure to violence among women and men and its association to health. National Centre for Knowledge on Men’s Violence Against Women (NCK); 2015.

[4] Annerbäck EM, Sahlqvist L, Svedin CG, Wingren G, Gustafsson PA. Child physical abuse and concurrence of other types of child abuse in Sweden-Associations with health and risk behaviors. Child Abuse Negl. 2012;36(7–8):585–95. 22854707 10.1016/j.chiabu.2012.05.006

[5] American Academy of Pediatrics, Stirling J, the Committee on Child Abuse and Neglect and Section on Adoption and Foster Care, American Academy of Child and Adolescent, Psychiatry, Amaya-Jackson L et al. National Center for Child Traumatic Stress,. Understanding the Behavioral and Emotional Consequences of Child Abuse. Pediatrics. 2008;122(3):667–73. 10.1542/peds.2008-1885 18762538

[6] Madigan S, Deneault A, Racine N, Park J, Thiemann R, Zhu J, et al. Adverse childhood experiences: a meta-analysis of prevalence and moderators among half a million adults in 206 studies. World Psychiatry. 2023;22(3):463–71. 37713544 10.1002/wps.21122 PMC10503911

[7] Felitti VJ, Anda RF, Nordenberg D, Williamson DF, Spitz AM, Edwards V, et al. Relationship of childhood abuse and household dysfunction to many of the leading causes of death in adults. The adverse childhood experiences (ACE) study. Am J Prev Med. 1998;14(4):245–58. 9635069 10.1016/s0749-3797(98)00017-8

[8] Petruccelli K, Davis J, Berman T. Adverse childhood experiences and associated health outcomes: A systematic review and meta-analysis. Child Abuse Negl. 2019;97:104127. 31454589 10.1016/j.chiabu.2019.104127

[9] Zhu S, Shan S, Liu W, Li S, Hou L, Huang X, et al. Adverse childhood experiences and risk of diabetes: A systematic review and meta-analysis. J Glob Health. 2022;12:04082. 36318589 10.7189/jogh.12.04082 PMC9624439

[10] Godoy LC, Frankfurter C, Cooper M, Lay C, Maunder R, Farkouh ME. Association of adverse childhood experiences with cardiovascular disease later in life: A review. JAMA Cardiol. 2021;6(2):228. 33263716 10.1001/jamacardio.2020.6050

[11] Holman DM, Ports KA, Buchanan ND, Hawkins NA, Merrick MT, Metzler M, et al. The association between adverse childhood experiences and risk of cancer in adulthood: A systematic review of the literature. Pediatrics. 2016;138(Supplement1):S81–91. 27940981 10.1542/peds.2015-4268L PMC5892430

[12] Negriff S. ACEs are not equal: examining the relative impact of household dysfunction versus childhood maltreatment on mental health in adolescence. Soc Sci Med. 2020;245:112696. 31785426 10.1016/j.socscimed.2019.112696 PMC6961803

[13] Atzl VM, Narayan AJ, Rivera LM, Lieberman AF. Adverse childhood experiences and prenatal mental health: type of aces and age of maltreatment onset. J Fam Psychol. 2019;33(3):304–14. 30802085 10.1037/fam0000510

[14] Turner HA, Finkelhor D, Ormrod R. Poly-Victimization in a National sample of children and youth. Am J Prev Med. 2010;38(3):323–30. 20171535 10.1016/j.amepre.2009.11.012

[15] Finkelhor D, Ormrod R, Turner H, Holt M. Pathways to Poly-Victimization. Child Maltreat. 2009;14(4):316–29. 19837972 10.1177/1077559509347012

[16] Widom CS, Czaja SJ, Dutton MA. Childhood victimization and lifetime revictimization. Child Abuse Negl. 2008;32(8):785–96. 18760474 10.1016/j.chiabu.2007.12.006 PMC2572709

[17] McIntyre JK, Spatz Widom C. Childhood victimization and crime victimization. J Interpers Violence. 2011;26(4):640–63. 20505112 10.1177/0886260510365868

[18] Simmons J, Swahnberg K. Lifetime prevalence of polyvictimization among older adults in Sweden, associations with ill-heath, and the mediating effect of sense of coherence. BMC Geriatr. 2021;21(1):129. 33596824 10.1186/s12877-021-02074-4 PMC7891035

[19] Olofsson N, Lindqvist K, Danielsson I. Fear of crime and psychological and physical abuse associated with ill health in a Swedish population aged 65–84 years. Public Health. 2012;126(4):358–64. 22386619 10.1016/j.puhe.2012.01.015

[20] Marks C, Pearson JL, Zúñiga ML, Martin N, Werb D, Smith LR. Articulating the Trauma-Informed theory of individual health behavior. Stress Health. 2022;38(1):154–62. 34009751 10.1002/smi.3068 PMC9035290

[21] Lundgren E. Captured queen: Men’s violence against women in ‘equal’ Sweden: a prevalence study. Umeå Uppsala Stockholm: Brottsoffermyndigheten; Univ.; Fritze distributör; 2002.

[22] Öberg M, Skalkidou A, Heimer G, Lucas S. Sexual violence against women in Sweden: associations with combined childhood violence and sociodemographic factors. Scand J Public Health. 2020;140349482093901. 10.1177/1403494820939015 32720565

[23] Särndal CE, Lundström S. Estimation in surveys with nonresponse. Hoboken, NJ: Wiley; 2005. p. 199.

[24] Ho NG. Children– the hidden or direct victims of domestic abuse? J Social Welf Family Law. 2022;44(4):512–28.

[25] Herrmann C. International experiences with the hospital anxiety and depression Scale-A review of validation data and clinical results. J Psychosom Res. 1997;42(1):17–41. 9055211 10.1016/s0022-3999(96)00216-4

[26] Ruggiero K, Ben K, Scotti J, Rabalais A. Psychometric properties of the PTSD Checklist—Civilian version. J Trauma Stress. 2003;16(5):495–502. 14584634 10.1023/A:1025714729117

[27] Kroenke K, Spitzer RL, Williams JBW. The PHQ-15: validity of a new measure for evaluating the severity of somatic symptoms. Psychosom Med. 2002;64(2):258–66. 11914441 10.1097/00006842-200203000-00008

[28] Bergman H, ALCOHOL USE AMONG SWEDES AND A PSYCHOMETRIC EVALUATION OF, THE ALCOHOL USE DISORDERS IDENTIFICATION TEST. Alcohol Alcohol. 2002;37(3):245–51. 12003912 10.1093/alcalc/37.3.245

[29] Dube SR, Felitti VJ, Dong M, Giles WH, Anda RF. The impact of adverse childhood experiences on health problems: evidence from four birth cohorts dating back to 1900. Prev Med. 2003;37(3):268–77. 12914833 10.1016/s0091-7435(03)00123-3

[30] Tikkanen R, Fields K, Williams RD III, Abrams MK. Mental Health Conditions and Substance Use: Comparing U.S. Needs and Treatment Capacity with Those in Other High-Income Countries [Internet]. Commonwealth Fund; 2020 [cited 2024 Oct 5]. Available from: https://www.commonwealthfund.org/publications/issue-briefs/2020/may/mental-health-conditions-substance-use-comparing-us-other-countries

[31] Bussières A, Hancock MJ, Elklit A, Ferreira ML, Ferreira PH, Stone LS, et al. Adverse childhood experience is associated with an increased risk of reporting chronic pain in adulthood: a stystematic review and meta-analysis. Eur J Psychotraumatology. 2023;14(2):2284025. 10.1080/20008066.2023.2284025 PMC10993817 38111090

[32] Joshee S, Lim L, Wybrecht A, Berriesford R, Riddle M. Meta-analysis and systematic review of the association between adverse childhood events and irritable bowel syndrome. J Investig Med. 2022;70(6):1342–51. 35086857 10.1136/jim-2021-002109

[33] Williamson DF, Thompson TJ, Anda RF, Dietz WH, Felitti V. Body weight and obesity in adults and self-reported abuse in childhood. Int J Obes Relat Metab Disord. 2002;26(8):1075–82. 12119573 10.1038/sj.ijo.0802038

[34] Schneider EC, Shah A, Doty MM, Roosa Tikkanen, Fields K, Williams IIRD, Mirror. Mirror 2021: Reflecting Poorly [Internet]. Commonwealth Fund; 2021 [cited 2024 Oct 5]. Available from: https://www.commonwealthfund.org/publications/fund-reports/2021/aug/mirror-mirror-2021-reflecting-poorly

[35] Johansson G. Overweight and obesity in Sweden. A five year follow-up, 2004–2008. Scand J Public Health. 2010;38(8):803–9. 20823045 10.1177/1403494810378917

[36] Moran AE, Forouzanfar MH, Roth GA, Mensah GA, Ezzati M, Flaxman A, et al. The global burden of ischemic heart disease in 1990 and 2010: the global burden of disease 2010 study. Circulation. 2014;129(14):1493–501. 24573351 10.1161/CIRCULATIONAHA.113.004046 PMC4181601

