# Supplementary material for: Violence in adulthood amplifies the health correlates of childhood maltreatment
Source: BMC Public Health. 2025 Mar 29;25:1193. doi: 10.1186/s12889-025-22469-x (PMC11954337; doi:10.1186/s12889-025-22469-x)
Supplement: Supplementary file 1 — Supplementary Material 1 [file 12889_2025_22469_MOESM1_ESM.pdf]

Logistic Regression

Notes

Output Created		05-OCT-2024 11:47:28
Comments		
Input	Data	/Users/stevenlc/Library/CloudStorage/OneDrive-Privat/ICloud filer/Doktorander/Rickard/Artikel 3/Revision art 3/Artikel3_revision.sav
	Active Dataset	DataSet7
	Filter	<none>
	Weight	<none>
	Split File	<none>
	N of Rows in Working Data File	10337
Missing Value Handling	Definition of Missing	User-defined missing values are treated as missing
Syntax		LOGISTIC REGRESSION VARIABLES PTSD_score_pos /METHOD=ENTER Maltreatment_0to3 /CONTRAST (Maltreatment_0to3)=Indicator(1) /PRINT=CI(95) /CRITERIA=PIN(0.05) POUT(0.10) ITERATE(20) CUT(0.5).
Resources	Processor Time	00:00:00,38
	Elapsed Time	00:00:00,00

Case Processing Summary

Unweighted Cases[a]		N	Percent
Selected Cases	Included in Analysis	8709	84.3
	Missing Cases	1628	15.7
	Total	10337	100.0
Unselected Cases		0	.0
Total		10337	100.0

a. If weight is in effect, see classification table for the total number of cases.

Dependent Variable Encoding

Original Value	Internal Value
1.00	0
2.00	1

Categorical Variables Codings

		Frequency	Parameter coding (1)	(2)	(3)
Maltreatment_0to3	.00	5790	.000	.000	.000
	1.00	1505	1.000	.000	.000
	2.00	687	.000	1.000	.000
	3.00	727	.000	.000	1.000

Block 0: Beginning Block

Classification Table[a,b]

Observed			Predicted PTSS 1.00	2.00	Percentage Correct
Step 0	PTSS	1.00	8085	0	100.0
		2.00	624	0	.0
	Overall Percentage				92.8

a. Constant is included in the model.

b. The cut value is .500

Variables in the Equation

		B	S.E.	Wald	df	Sig.	Exp(B)
Step 0	Constant	-2.562	.042	3801.230	1	.000	.077

Variables not in the Equation

			Score	df	Sig.
Step 0	Variables	Maltreatment_0to3	585.321	3	<.001
		Maltreatment_0to3(1)	11.730	1	<.001
		Maltreatment_0to3(2)	23.989	1	<.001
		Maltreatment_0to3(3)	480.360	1	<.001
	Overall Statistics		585.321	3	<.001

Block 1: Method = Enter

Omnibus Tests of Model Coefficients

		Chi-square	df	Sig.
Step 1	Step	436.052	3	<.001
	Block	436.052	3	<.001
	Model	436.052	3	<.001

Model Summary

Step	-2 Log likelihood	Cox & Snell R Square	Nagelkerke R Square
1	4055.811[a]	.049	.121

a. Estimation terminated at iteration number 6 because parameter estimates changed by less than .001.

Classification Table[a]

Observed			Predicted PTSS 1.00	Predicted PTSS 2.00	Percentage Correct
Step 1	PTSS	1.00	8085	0	100.0
		2.00	624	0	.0
	Overall Percentage				92.8

a. The cut value is .500

Variables in the Equation

		B	S.E.	Wald	df	Sig.
Step 1[a]	Maltreatment_0to3			454.181	3	<.001
	Maltreatment_0to3(1)	1.015	.114	79.434	1	<.001
	Maltreatment_0to3(2)	1.287	.138	87.091	1	<.001
	Maltreatment_0to3(3)	2.317	.109	448.358	1	<.001
	Constant	-3.300	.071	2163.243	1	.000

Variables in the Equation

		Exp(B)	95% C.I.for EXP(B) Lower	95% C.I.for EXP(B) Upper
Step 1[a]	Maltreatment_0to3			
	Maltreatment_0to3(1)	2.758	2.207	3.448
	Maltreatment_0to3(2)	3.623	2.765	4.748
	Maltreatment_0to3(3)	10.146	8.187	12.573
	Constant	.037		

a. Variable(s) entered on step 1: Maltreatment_0to3.

Logistic Regression

Notes

Output Created		05-OCT-2024 11:47:28
Comments		
Input	Data	/Users/stevenlc/Library/CloudStorage/OneDrive-Privat/ICloud filer/Doktorander/Rickard/Artikel 3/Revision art 3/Artikel3_revision.sav
	Active Dataset	DataSet7
	Filter	<none>
	Weight	<none>
	Split File	<none>
	N of Rows in Working Data File	10337
Missing Value Handling	Definition of Missing	User-defined missing values are treated as missing
Syntax		LOGISTIC REGRESSION VARIABLES PTSD_score_pos /METHOD=ENTER Maltreatment_0to3 Householddysf_parent Kon barnboendeny utbildningmammapappa_ny fodelselandmammapappa_ny alderskategorier /CONTRAST (Maltreatment_0to3)=Indicator(1) /CONTRAST (Householddysf_parent)=Indicator(1) /CONTRAST (Kon)=Indicator(1) /CONTRAST (barnboendeny)=Indicator(1) /CONTRAST (utbildningmammapappa_ny)=Indicator(1) /CONTRAST (fodelselandmammapappa_ny)=Indicator(1) /CONTRAST (alderskategorier)=Indicator(1) /PRINT=CI(95) /CRITERIA=PIN(0.05) POUT(0.10) ITERATE(20) CUT(0.5).
Resources	Processor Time	00:00:00,44
	Elapsed Time	00:00:01,00

Case Processing Summary

Unweighted Cases[a]		N	Percent
Selected Cases	Included in Analysis	7282	70.4
	Missing Cases	3055	29.6
	Total	10337	100.0
Unselected Cases		0	.0
Total		10337	100.0

a. If weight is in effect, see classification table for the total number of cases.

Dependent Variable Encoding

Original Value	Internal Value
1.00	0
2.00	1

Categorical Variables Codings

		Frequency	Parameter coding		
			(1)	(2)	(3)
Age categories	17-25	878	.000	.000	.000
	26-35	1160	1.000	.000	.000
	36-45	1380	.000	1.000	.000
	46-55	1442	.000	.000	1.000
	56-65	1500	.000	.000	.000
	66-74	922	.000	.000	.000
Maltreatment_0to3	.00	4963	.000	.000	.000
	1.00	1228	1.000	.000	.000
	2.00	549	.000	1.000	.000
	3.00	542	.000	.000	1.000
Parent education	At least one parent high school	3896	.000		
	Both parents below high school	3386	1.000		
Householddysf_parent	.00	5505	.000		
	1.00	1777	1.000		
Sex	Man	3276	.000		
	Kvinna	4006	1.000		
Immigrant status	At least one Nordic parent	6827	.000		
	Both parents born elsewhere	455	1.000		
Residence type	Owned home	5470	.000		
	Rental	1812	1.000		

Categorical Variables Codings

		Parameter coding (4)	Parameter coding (5)
Age categories	17-25	.000	.000
	26-35	.000	.000
	36-45	.000	.000
	46-55	.000	.000
	56-65	1.000	.000
	66-74	.000	1.000
Maltreatment_0to3	.00		
	1.00		
	2.00		
	3.00		
Parent education	At least one parent high school		
	Both parents below high school		
Householddysf_parent	.00		
	1.00		
Sex	Man		
	Kvinna		
Immigrant status	At least one Nordic parent		
	Both parents born elsewhere		
Residence type	Owned home		
	Rental		

Block 0: Beginning Block

Classification Table[a,b]

Observed			Predicted PTSS 1.00	Predicted PTSS 2.00	Percentage Correct
Step 0	PTSS	1.00	6804	0	100.0
		2.00	478	0	.0
	Overall Percentage				93.4

a. Constant is included in the model.

b. The cut value is .500

Variables in the Equation

		B	S.E.	Wald	df	Sig.	Exp(B)
Step 0	Constant	-2.656	.047	3149.814	1	.000	.070

Variables not in the Equation

			Score	df	Sig.
Step 0	Variables	Maltreatment_0to3	389.734	3	<.001
		Maltreatment_0to3(1)	13.797	1	<.001
		Maltreatment_0to3(2)	32.815	1	<.001
		Maltreatment_0to3(3)	289.768	1	<.001
		Householddysf_parent(1)	134.719	1	<.001
		Sex(1)	45.659	1	<.001
		Residence type(1)	13.091	1	<.001
		Parent education(1)	18.438	1	<.001
		Immigrant status(1)	13.992	1	<.001
		Age categories	75.912	5	<.001
		Age categories(1)	7.986	1	.005
		Age categories(2)	.306	1	.580
		Age categories(3)	.403	1	.526
		Age categories(4)	11.090	1	<.001
		Age categories(5)	28.505	1	<.001
	Overall Statistics		525.536	13	<.001

Block 1: Method = Enter

Omnibus Tests of Model Coefficients

		Chi-square	df	Sig.
Step 1	Step	437.535	13	<.001
	Block	437.535	13	<.001
	Model	437.535	13	<.001

Model Summary

Step	-2 Log likelihood	Cox & Snell R Square	Nagelkerke R Square
1	3090.092[a]	.058	.152

a. Estimation terminated at iteration number 6 because parameter estimates changed by less than .001.

Classification Table[a]

Observed			Predicted PTSS 1.00	Predicted PTSS 2.00	Percentage Correct
Step 1	PTSS	1.00	6799	5	99.9
		2.00	474	4	.8
	Overall Percentage				93.4

a. The cut value is .500

Variables in the Equation

		B	S.E.	Wald	df	Sig.
Step 1[a]	Maltreatment_0to3			218.894	3	<.001
	Maltreatment_0to3(1)	.948	.130	53.397	1	<.001
	Maltreatment_0to3(2)	1.296	.158	67.570	1	<.001
	Maltreatment_0to3(3)	2.033	.141	207.091	1	<.001
	Householddysf_parent(1)	.403	.110	13.395	1	<.001
	Sex(1)	.552	.106	26.941	1	<.001
	Residence type(1)	.142	.115	1.543	1	.214
	Parent education(1)	-.121	.117	1.067	1	.302
	Immigrant status(1)	.244	.173	2.006	1	.157
	Age categories			47.861	5	<.001
	Age categories(1)	-.428	.158	7.309	1	.007
	Age categories(2)	-.764	.164	21.700	1	<.001
	Age categories(3)	-.701	.166	17.819	1	<.001
	Age categories(4)	-1.022	.187	30.008	1	<.001
	Age categories(5)	-1.477	.259	32.635	1	<.001
	Constant	-3.107	.149	432.860	1	<.001

Variables in the Equation

		Exp(B)	95% C.I.for EXP(B)	
			Lower	Upper
Step 1[a]	Maltreatment_0to3			
	Maltreatment_0to3(1)	2.581	2.001	3.329
	Maltreatment_0to3(2)	3.654	2.683	4.977
	Maltreatment_0to3(3)	7.638	5.790	10.075
	Householddysf_parent(1)	1.496	1.206	1.855
	Sex(1)	1.736	1.410	2.138
	Residence type(1)	1.153	.921	1.444
	Parent education(1)	.886	.705	1.114
	Immigrant status(1)	1.277	.910	1.791
	Age categories			
	Age categories(1)	.652	.478	.889
	Age categories(2)	.466	.338	.642
	Age categories(3)	.496	.358	.687
	Age categories(4)	.360	.250	.519
	Age categories(5)	.228	.138	.379
	Constant	.045		

a. Variable(s) entered on step 1: Maltreatment_0to3, Householddysf_parent, Sex, Residence type, Parent education, Immigrant status, Age categories.

Logistic Regression

Notes

Output Created		05-OCT-2024 11:47:29
Comments		
Input	Data	/Users/stevenlc/Library/CloudStorage/OneDrive-Privat/ICloud filer/Doktorander/Rickard/Artikel 3/Revision art 3/Artikel3_revision.sav
	Active Dataset	DataSet7
	Filter	<none>
	Weight	<none>
	Split File	<none>
	N of Rows in Working Data File	10337
Missing Value Handling	Definition of Missing	User-defined missing values are treated as missing
Syntax		LOGISTIC REGRESSION VARIABLES PTSD_score_pos /METHOD=ENTER Lifetimeabuseindex /CONTRAST (Lifetimeabuseindex)=Indicator(1) /PRINT=CI(95) /CRITERIA=PIN(0.05) POUT(0.10) ITERATE(20) CUT(0.5).
Resources	Processor Time	00:00:00,40
	Elapsed Time	00:00:00,00

Case Processing Summary

Unweighted Cases[a]		N	Percent
Selected Cases	Included in Analysis	8518	82.4
	Missing Cases	1819	17.6
	Total	10337	100.0
Unselected Cases		0	.0
Total		10337	100.0

a. If weight is in effect, see classification table for the total number of cases.

Dependent Variable Encoding

Original Value	Internal Value
1.00	0
2.00	1

Categorical Variables Codings

		Frequency	Parameter coding (1)	(2)	(3)	(4)	(5)
Lifetimeabuseindex	.00	4838	.000	.000	.000	.000	.000
	1.00	1008	1.000	.000	.000	.000	.000
	2.00	424	.000	1.000	.000	.000	.000
	3.00	351	.000	.000	1.000	.000	.000
	4.00	833	.000	.000	.000	1.000	.000
	5.00	458	.000	.000	.000	.000	1.000
	6.00	247	.000	.000	.000	.000	.000
	7.00	359	.000	.000	.000	.000	.000

Categorical Variables Codings

		Parameter coding (6)	(7)
Lifetimeabuseindex	.00	.000	.000
	1.00	.000	.000
	2.00	.000	.000
	3.00	.000	.000
	4.00	.000	.000
	5.00	.000	.000
	6.00	1.000	.000
	7.00	.000	1.000

Block 0: Beginning Block

Classification Table[a,b]

Observed			Predicted PTSS 1.00	PTSS 2.00	Percentage Correct
Step 0	PTSS	1.00	7908	0	100.0
		2.00	610	0	.0
	Overall Percentage				92.8

a. Constant is included in the model.

b. The cut value is .500

Variables in the Equation

		B	S.E.	Wald	df	Sig.	Exp(B)
Step 0	Constant	-2.562	.042	3717.707	1	.000	.077

Variables not in the Equation

			Score	df	Sig.
Step 0	Variables	Lifetimeabuseindex	777.317	7	<.001
		Lifetimeabuseindex(1)	.874	1	.350
		Lifetimeabuseindex(2)	.069	1	.792
		Lifetimeabuseindex(3)	64.076	1	<.001
		Lifetimeabuseindex(4)	6.022	1	.014
		Lifetimeabuseindex(5)	50.649	1	<.001
		Lifetimeabuseindex(6)	65.475	1	<.001
		Lifetimeabuseindex(7)	466.669	1	<.001
	Overall Statistics		777.317	7	<.001

Block 1: Method = Enter

Omnibus Tests of Model Coefficients

		Chi-square	df	Sig.
Step 1	Step	573.563	7	<.001
	Block	573.563	7	<.001
	Model	573.563	7	<.001

Model Summary

Step	-2 Log likelihood	Cox & Snell R Square	Nagelkerke R Square
1	3818.174[a]	.065	.162

a. Estimation terminated at iteration number 6 because parameter estimates changed by less than .001.

Classification Table[a]

			Predicted		
			PTSS		Percentage Correct
	Observed		1.00	2.00	
Step 1	PTSS	1.00	7908	0	100.0
		2.00	610	0	.0
	Overall Percentage				92.8

a. The cut value is .500

Variables in the Equation

		B	S.E.	Wald	df	Sig.
Step 1[a]	Lifetimeabuseindex			550.414	7	<.001
	Lifetimeabuseindex(1)	.947	.157	36.458	1	<.001
	Lifetimeabuseindex(2)	1.010	.213	22.587	1	<.001
	Lifetimeabuseindex(3)	2.102	.166	160.668	1	<.001
	Lifetimeabuseindex(4)	1.337	.150	79.636	1	<.001
	Lifetimeabuseindex(5)	1.926	.158	149.447	1	<.001
	Lifetimeabuseindex(6)	2.250	.182	152.426	1	<.001
	Lifetimeabuseindex(7)	3.043	.142	457.406	1	<.001
	Constant	-3.622	.090	1609.561	1	.000

Variables in the Equation

			95% C.I.for EXP(B)	
		Exp(B)	Lower	Upper
Step 1[a]	Lifetimeabuseindex			
	Lifetimeabuseindex(1)	2.578	1.896	3.505
	Lifetimeabuseindex(2)	2.746	1.810	4.164
	Lifetimeabuseindex(3)	8.181	5.911	11.322
	Lifetimeabuseindex(4)	3.809	2.839	5.109
	Lifetimeabuseindex(5)	6.861	5.038	9.343
	Lifetimeabuseindex(6)	9.492	6.640	13.567
	Lifetimeabuseindex(7)	20.975	15.870	27.722
	Constant	.027		

a. Variable(s) entered on step 1: Lifetimeabuseindex.

Logistic Regression

Notes

Output Created		05-OCT-2024 11:47:29
Comments		
Input	Data	/Users/stevenlc/Library/CloudStorage/OneDrive-Privat/ICloud filer/Doktorander/Rickard/Artikel 3/Revision art 3/Artikel3_revision.sav
	Active Dataset	DataSet7
	Filter	<none>
	Weight	<none>
	Split File	<none>
	N of Rows in Working Data File	10337
Missing Value Handling	Definition of Missing	User-defined missing values are treated as missing
Syntax		LOGISTIC REGRESSION VARIABLES PTSD_score_pos /METHOD=ENTER Lifetimeabuseindex Householddysf_parent Kon barnboendeny utbildningmammapappa_ny fodelselandmammapappa_ny alderskategorier /CONTRAST (Lifetimeabuseindex)=Indicator(1) /CONTRAST (Householddysf_parent)=Indicator(1) /CONTRAST (Kon)=Indicator(1) /CONTRAST (barnboendeny)=Indicator(1) /CONTRAST (utbildningmammapappa_ny)=Indicator(1) /CONTRAST (fodelselandmammapappa_ny)=Indicator(1) /CONTRAST (alderskategorier)=Indicator(1) /PRINT=CI(95) /CRITERIA=PIN(0.05) POUT(0.10) ITERATE(20) CUT(0.5).
Resources	Processor Time	00:00:00,47
	Elapsed Time	00:00:01,00

Case Processing Summary

Unweighted Cases[a]		N	Percent
Selected Cases	Included in Analysis	7141	69.1
	Missing Cases	3196	30.9
	Total	10337	100.0
Unselected Cases		0	.0
Total		10337	100.0

a. If weight is in effect, see classification table for the total number of cases.

Dependent Variable Encoding

Original Value	Internal Value
1.00	0
2.00	1

Categorical Variables Codings

		Frequency	Parameter coding (1)	(2)	(3)
Lifetimeabuseindex	.00	4166	.000	.000	.000
	1.00	826	1.000	.000	.000
	2.00	333	.000	1.000	.000
	3.00	254	.000	.000	1.000
	4.00	706	.000	.000	.000
	5.00	376	.000	.000	.000
	6.00	204	.000	.000	.000
	7.00	276	.000	.000	.000
Age categories	17-25	859	.000	.000	.000
	26-35	1139	1.000	.000	.000
	36-45	1354	.000	1.000	.000
	46-55	1412	.000	.000	1.000
	56-65	1474	.000	.000	.000
	66-74	903	.000	.000	.000
Parent education	At least one parent high school	3808	.000		
	Both parents below high school	3333	1.000		
Householddysf_parent	.00	5406	.000		
	1.00	1735	1.000		
Sex	Man	3209	.000		
	Kvinna	3932	1.000		
Immigrant status	At least one Nordic parent	6695	.000		
	Both parents born elsewhere	446	1.000		
Residence type	Owned home	5358	.000		
	Rental	1783	1.000		

Categorical Variables Codings

		Parameter coding			
		(4)	(5)	(6)	(7)
Lifetimeabuseindex	.00	.000	.000	.000	.000
	1.00	.000	.000	.000	.000
	2.00	.000	.000	.000	.000
	3.00	.000	.000	.000	.000
	4.00	1.000	.000	.000	.000
	5.00	.000	1.000	.000	.000
	6.00	.000	.000	1.000	.000
	7.00	.000	.000	.000	1.000
Age categories	17-25	.000	.000		
	26-35	.000	.000		
	36-45	.000	.000		
	46-55	.000	.000		
	56-65	1.000	.000		
	66-74	.000	1.000		
Parent education	At least one parent high school				
	Both parents below high school				
Householddysf_parent	.00				
	1.00				
Sex	Man				
	Kvinna				
Immigrant status	At least one Nordic parent				
	Both parents born elsewhere				
Residence type	Owned home				
	Rental				

Block 0: Beginning Block

Classification Table[a,b]

Observed			Predicted PTSS 1.00	Predicted PTSS 2.00	Percentage Correct
Step 0	PTSS	1.00	6673	0	100.0
		2.00	468	0	.0
	Overall Percentage				93.4

a. Constant is included in the model.

b. The cut value is .500

Variables in the Equation

		B	S.E.	Wald	df	Sig.	Exp(B)
Step 0	Constant	-2.657	.048	3088.215	1	.000	.070

Variables not in the Equation

			Score	df	Sig.
Step 0	Variables	Lifetimeabuseindex	565.892	7	<.001
		Lifetimeabuseindex(1)	.382	1	.537
		Lifetimeabuseindex(2)	.519	1	.471
		Lifetimeabuseindex(3)	22.454	1	<.001
		Lifetimeabuseindex(4)	9.992	1	.002
		Lifetimeabuseindex(5)	47.998	1	<.001
		Lifetimeabuseindex(6)	62.905	1	<.001
		Lifetimeabuseindex(7)	327.157	1	<.001
		Householddysf_parent(1)	130.085	1	<.001
		Sex(1)	43.120	1	<.001
		Residence type(1)	10.369	1	.001
		Parent education(1)	18.965	1	<.001
		Immigrant status(1)	13.759	1	<.001
		Age categories	78.671	5	<.001
		Age categories(1)	9.302	1	.002
		Age categories(2)	.334	1	.563
		Age categories(3)	.287	1	.592
		Age categories(4)	13.072	1	<.001
		Age categories(5)	27.096	1	<.001
	Overall Statistics		680.762	17	<.001

Block 1: Method = Enter

Omnibus Tests of Model Coefficients

		Chi-square	df	Sig.
Step 1	Step	538.693	17	<.001
	Block	538.693	17	<.001
	Model	538.693	17	<.001

Model Summary

Step	-2 Log likelihood	Cox & Snell R Square	Nagelkerke R Square
1	2916.674[a]	.073	.189

a. Estimation terminated at iteration number 7 because parameter estimates changed by less than .001.

Classification Table[a]

Observed			Predicted PTSS 1.00	Predicted PTSS 2.00	Percentage Correct
Step 1	PTSS	1.00	6666	7	99.9
		2.00	455	13	2.8
	Overall Percentage				93.5

a. The cut value is .500

Variables in the Equation

		B	S.E.	Wald	df	Sig.
Step 1[a]	Lifetimeabuseindex			307.089	7	<.001
	Lifetimeabuseindex(1)	.902	.179	25.308	1	<.001
	Lifetimeabuseindex(2)	1.125	.236	22.756	1	<.001
	Lifetimeabuseindex(3)	1.770	.219	65.508	1	<.001
	Lifetimeabuseindex(4)	1.379	.166	69.347	1	<.001
	Lifetimeabuseindex(5)	1.842	.180	104.709	1	<.001
	Lifetimeabuseindex(6)	2.150	.209	105.578	1	<.001
	Lifetimeabuseindex(7)	2.794	.176	253.361	1	<.001
	Householddysf_parent(1)	.364	.113	10.419	1	.001
	Sex(1)	.511	.109	22.081	1	<.001
	Residence type(1)	.103	.118	.759	1	.384
	Parent education(1)	-.037	.120	.097	1	.756
	Immigrant status(1)	.296	.178	2.784	1	.095
	Age categories			50.633	5	<.001
	Age categories(1)	-.481	.162	8.828	1	.003
	Age categories(2)	-.902	.169	28.584	1	<.001
	Age categories(3)	-.847	.172	24.318	1	<.001
	Age categories(4)	-1.093	.192	32.268	1	<.001
	Age categories(5)	-1.414	.262	29.115	1	<.001
	Constant	-3.361	.163	427.149	1	<.001

Variables in the Equation

		Exp(B)	95% C.I.for EXP(B) Lower	Upper
Step 1[a]	Lifetimeabuseindex			
	Lifetimeabuseindex(1)	2.463	1.734	3.500
	Lifetimeabuseindex(2)	3.082	1.941	4.893
	Lifetimeabuseindex(3)	5.871	3.824	9.013
	Lifetimeabuseindex(4)	3.972	2.871	5.496
	Lifetimeabuseindex(5)	6.310	4.434	8.979
	Lifetimeabuseindex(6)	8.588	5.698	12.942
	Lifetimeabuseindex(7)	16.344	11.587	23.055
	Householddysf_parent(1)	1.439	1.154	1.795
	Sex(1)	1.666	1.347	2.062
	Residence type(1)	1.109	.879	1.398
	Parent education(1)	.963	.762	1.219
	Immigrant status(1)	1.345	.950	1.906
	Age categories			
	Age categories(1)	.618	.450	.849
	Age categories(2)	.406	.292	.565
	Age categories(3)	.429	.306	.600
	Age categories(4)	.335	.230	.489
	Age categories(5)	.243	.145	.406
	Constant	.035		

a. Variable(s) entered on step 1: Lifetimeabuseindex, Householddysf_parent, Sex, Residence type, Parent education, Immigrant status, Age categories.

Logistic Regression

Notes

Output Created		05-OCT-2024 11:47:30
Comments		
Input	Data	/Users/stevenlc/Library/CloudStorage/OneDrive-Privat/ICloud filer/Doktorander/Rickard/Artikel 3/Revision art 3/Artikel3_revision.sav
	Active Dataset	DataSet7
	Filter	<none>
	Weight	<none>
	Split File	<none>
	N of Rows in Working Data File	10337
Missing Value Handling	Definition of Missing	User-defined missing values are treated as missing
Syntax		LOGISTIC REGRESSION VARIABLES HAD_probable_depression /METHOD=ENTER Maltreatment_0to3 /CONTRAST (Maltreatment_0to3)=Indicator(1) /PRINT=CI(95) /CRITERIA=PIN(0.05) POUT(0.10) ITERATE(20) CUT(0.5).
Resources	Processor Time	00:00:00,39
	Elapsed Time	00:00:00,00

Case Processing Summary

Unweighted Cases[a]		N	Percent
Selected Cases	Included in Analysis	8833	85.5
	Missing Cases	1504	14.5
	Total	10337	100.0
Unselected Cases		0	.0
Total		10337	100.0

a. If weight is in effect, see classification table for the total number of cases.

Dependent Variable Encoding

Original Value	Internal Value
1.00	0
2.00	1

Categorical Variables Codings

		Frequency	Parameter coding (1)	(2)	(3)
Maltreatment_0to3	.00	5839	.000	.000	.000
	1.00	1519	1.000	.000	.000
	2.00	720	.000	1.000	.000
	3.00	755	.000	.000	1.000

Block 0: Beginning Block

Classification Table[a,b]

Observed			Predicted Depression 1.00	2.00	Percentage Correct
Step 0	Depression	1.00	8087	0	100.0
		2.00	746	0	.0
	Overall Percentage				91.6

a. Constant is included in the model.

b. The cut value is .500

Variables in the Equation

		B	S.E.	Wald	df	Sig.	Exp(B)
Step 0	Constant	-2.383	.038	3879.457	1	.000	.092

Variables not in the Equation

			Score	df	Sig.
Step 0	Variables	Maltreatment_0to3	366.832	3	<.001
		Maltreatment_0to3(1)	25.409	1	<.001
		Maltreatment_0to3(2)	16.665	1	<.001
		Maltreatment_0to3(3)	266.293	1	<.001
	Overall Statistics		366.832	3	<.001

Block 1: Method = Enter

Omnibus Tests of Model Coefficients

		Chi-square	df	Sig.
Step 1	Step	302.115	3	<.001
	Block	302.115	3	<.001
	Model	302.115	3	<.001

Model Summary

Step	-2 Log likelihood	Cox & Snell R Square	Nagelkerke R Square
1	4812.542[a]	.034	.076

a. Estimation terminated at iteration number 6 because parameter estimates changed by less than .001.

Classification Table[a]

Observed			Predicted Depression 1.00	Predicted Depression 2.00	Predicted Percentage Correct
Step 1	Depression	1.00	8087	0	100.0
		2.00	746	0	.0
	Overall Percentage				91.6

a. The cut value is .500

Variables in the Equation

		B	S.E.	Wald	df	Sig.
Step 1[a]	Maltreatment_0to3			316.635	3	<.001
	Maltreatment_0to3(1)	.914	.100	84.116	1	<.001
	Maltreatment_0to3(2)	.988	.128	59.951	1	<.001
	Maltreatment_0to3(3)	1.794	.104	298.423	1	<.001
	Constant	-2.933	.060	2410.337	1	.000

Variables in the Equation

		Exp(B)	95% C.I.for EXP(B) Lower	95% C.I.for EXP(B) Upper
Step 1[a]	Maltreatment_0to3			
	Maltreatment_0to3(1)	2.495	2.052	3.033
	Maltreatment_0to3(2)	2.685	2.091	3.447
	Maltreatment_0to3(3)	6.013	4.905	7.370
	Constant	.053		

a. Variable(s) entered on step 1: Maltreatment_0to3.

Logistic Regression

Notes

Output Created		05-OCT-2024 11:47:30
Comments		
Input	Data	/Users/stevenlc/Library/CloudStorage/OneDrive-Privat/ICloud filer/Doktorander/Rickard/Artikel 3/Revision art 3/Artikel3_revision.sav
	Active Dataset	DataSet7
	Filter	<none>
	Weight	<none>
	Split File	<none>
	N of Rows in Working Data File	10337
Missing Value Handling	Definition of Missing	User-defined missing values are treated as missing
Syntax		LOGISTIC REGRESSION VARIABLES HAD_probable_depression /METHOD=ENTER Maltreatment_0to3 Householddysf_parent Kon barnboendeny utbildningmammapappa_ny fodelselandmammapappa_ny alderskategorier /CONTRAST (Maltreatment_0to3)=Indicator(1) /CONTRAST (Householddysf_parent)=Indicator(1) /CONTRAST (Kon)=Indicator(1) /CONTRAST (barnboendeny)=Indicator(1) /CONTRAST (utbildningmammapappa_ny)=Indicator(1) /CONTRAST (fodelselandmammapappa_ny)=Indicator(1) /CONTRAST (alderskategorier)=Indicator(1) /PRINT=CI(95) /CRITERIA=PIN(0.05) POUT(0.10) ITERATE(20) CUT(0.5).
Resources	Processor Time	00:00:00,44
	Elapsed Time	00:00:00,00

Case Processing Summary

Unweighted Cases[a]		N	Percent
Selected Cases	Included in Analysis	7370	71.3
	Missing Cases	2967	28.7
	Total	10337	100.0
Unselected Cases		0	.0
Total		10337	100.0

a. If weight is in effect, see classification table for the total number of cases.

Dependent Variable Encoding

Original Value	Internal Value
1.00	0
2.00	1

Categorical Variables Codings

			Parameter coding		
		Frequency	(1)	(2)	(3)
Age categories	17-25	885	.000	.000	.000
	26-35	1165	1.000	.000	.000
	36-45	1395	.000	1.000	.000
	46-55	1468	.000	.000	1.000
	56-65	1521	.000	.000	.000
	66-74	936	.000	.000	.000
Maltreatment_0to3	.00	4998	.000	.000	.000
	1.00	1239	1.000	.000	.000
	2.00	574	.000	1.000	.000
	3.00	559	.000	.000	1.000
Parent education	At least one parent high school	3943	.000		
	Both parents below high school	3427	1.000		
Householddysf_parent	.00	5566	.000		
	1.00	1804	1.000		
Sex	Man	3316	.000		
	Kvinna	4054	1.000		
Immigrant status	At least one Nordic parent	6910	.000		
	Both parents born elsewhere	460	1.000		
Residence type	Owned home	5522	.000		
	Rental	1848	1.000		

Categorical Variables Codings

		Parameter coding (4)	Parameter coding (5)
Age categories	17-25	.000	.000
	26-35	.000	.000
	36-45	.000	.000
	46-55	.000	.000
	56-65	1.000	.000
	66-74	.000	1.000
Maltreatment_0to3	.00		
	1.00		
	2.00		
	3.00		
Parent education	At least one parent high school		
	Both parents below high school		
Householddysf_parent	.00		
	1.00		
Sex	Man		
	Kvinna		
Immigrant status	At least one Nordic parent		
	Both parents born elsewhere		
Residence type	Owned home		
	Rental		

Block 0: Beginning Block

Classification Table[a,b]

Observed			Predicted: Depression 1.00	Predicted: Depression 2.00	Predicted: Percentage Correct
Step 0	Depression	1.00	6795	0	100.0
		2.00	575	0	.0
	Overall Percentage				92.2

a. Constant is included in the model.

b. The cut value is .500

Variables in the Equation

		B	S.E.	Wald	df	Sig.	Exp(B)
Step 0	Constant	-2.470	.043	3233.205	1	.000	.085

Variables not in the Equation

			Score	df	Sig.
Step 0	Variables	Maltreatment_0to3	267.044	3	<.001
		Maltreatment_0to3(1)	24.173	1	<.001
		Maltreatment_0to3(2)	7.786	1	.005
		Maltreatment_0to3(3)	196.205	1	<.001
		Householddysf_parent(1)	19.090	1	<.001
		Sex(1)	1.231	1	.267
		Residence type(1)	.781	1	.377
		Parent education(1)	1.791	1	.181
		Immigrant status(1)	13.037	1	<.001
		Age categories	15.420	5	.009
		Age categories(1)	2.821	1	.093
		Age categories(2)	2.130	1	.144
		Age categories(3)	.072	1	.788
		Age categories(4)	7.008	1	.008
		Age categories(5)	4.369	1	.037
	Overall Statistics		291.452	13	<.001

Block 1: Method = Enter

Omnibus Tests of Model Coefficients

		Chi-square	df	Sig.
Step 1	Step	239.790	13	<.001
	Block	239.790	13	<.001
	Model	239.790	13	<.001

Model Summary

Step	-2 Log likelihood	Cox & Snell R Square	Nagelkerke R Square
1	3797.558[a]	.032	.076

a. Estimation terminated at iteration number 6 because parameter estimates changed by less than .001.

Classification Table[a]

	Observed		Predicted Depression 1.00	Predicted Depression 2.00	Predicted Percentage Correct
Step 1	Depression	1.00	6795	0	100.0
		2.00	575	0	.0
	Overall Percentage				92.2

a. The cut value is .500

Variables in the Equation

		B	S.E.	Wald	df	Sig.
Step 1[a]	Maltreatment_0to3			208.036	3	<.001
	Maltreatment_0to3(1)	.911	.113	65.351	1	<.001
	Maltreatment_0to3(2)	.894	.153	34.220	1	<.001
	Maltreatment_0to3(3)	1.829	.131	194.429	1	<.001
	Householddysf_parent(1)	-.100	.107	.877	1	.349
	Sex(1)	.007	.090	.005	1	.942
	Residence type(1)	-.070	.107	.429	1	.512
	Parent education(1)	.046	.102	.205	1	.651
	Immigrant status(1)	.294	.158	3.488	1	.062
	Age categories			15.619	5	.008
	Age categories(1)	-.114	.160	.506	1	.477
	Age categories(2)	-.151	.157	.923	1	.337
	Age categories(3)	-.363	.164	4.923	1	.026
	Age categories(4)	-.583	.177	10.921	1	<.001
	Age categories(5)	-.516	.201	6.611	1	.010
	Constant	-2.693	.142	358.669	1	<.001

Variables in the Equation

		Exp(B)	95% C.I.for EXP(B)	
			Lower	Upper
Step 1[a]	Maltreatment_0to3			
	Maltreatment_0to3(1)	2.488	1.995	3.103
	Maltreatment_0to3(2)	2.445	1.812	3.298
	Maltreatment_0to3(3)	6.227	4.815	8.052
	Householddysf_parent(1)	.905	.734	1.116
	Sex(1)	1.007	.844	1.201
	Residence type(1)	.933	.757	1.149
	Parent education(1)	1.047	.857	1.280
	Immigrant status(1)	1.342	.986	1.828
	Age categories			
	Age categories(1)	.892	.652	1.221
	Age categories(2)	.860	.633	1.170
	Age categories(3)	.696	.505	.959
	Age categories(4)	.558	.395	.789
	Age categories(5)	.597	.403	.885
	Constant	.068		

a. Variable(s) entered on step 1: Maltreatment_0to3, Householddysf_parent, Sex, Residence type, Parent education, Immigrant status, Age categories.

Logistic Regression

Notes

Output Created		05-OCT-2024 11:47:30
Comments		
Input	Data	/Users/stevenlc/Library/CloudStorage/OneDrive-Privat/ICloud filer/Doktorander/Rickard/Artikel 3/Revision art 3/Artikel3_revision.sav
	Active Dataset	DataSet7
	Filter	<none>
	Weight	<none>
	Split File	<none>
	N of Rows in Working Data File	10337
Missing Value Handling	Definition of Missing	User-defined missing values are treated as missing
Syntax		LOGISTIC REGRESSION VARIABLES HAD_probable_depression /METHOD=ENTER Lifetimeabuseindex /CONTRAST (Lifetimeabuseindex)=Indicator(1) /PRINT=CI(95) /CRITERIA=PIN(0.05) POUT(0.10) ITERATE(20) CUT(0.5).
Resources	Processor Time	00:00:00,41
	Elapsed Time	00:00:01,00

Case Processing Summary

Unweighted Cases[a]		N	Percent
Selected Cases	Included in Analysis	8637	83.6
	Missing Cases	1700	16.4
	Total	10337	100.0
Unselected Cases		0	.0
Total		10337	100.0

a. If weight is in effect, see classification table for the total number of cases.

Dependent Variable Encoding

Original Value	Internal Value
1.00	0
2.00	1

Categorical Variables Codings

		Frequency	Parameter coding (1)	(2)	(3)	(4)	(5)
Lifetimeabuseindex	.00	4865	.000	.000	.000	.000	.000
	1.00	1011	1.000	.000	.000	.000	.000
	2.00	440	.000	1.000	.000	.000	.000
	3.00	363	.000	.000	1.000	.000	.000
	4.00	856	.000	.000	.000	1.000	.000
	5.00	466	.000	.000	.000	.000	1.000
	6.00	263	.000	.000	.000	.000	.000
	7.00	373	.000	.000	.000	.000	.000

Categorical Variables Codings

		Parameter coding (6)	(7)
Lifetimeabuseindex	.00	.000	.000
	1.00	.000	.000
	2.00	.000	.000
	3.00	.000	.000
	4.00	.000	.000
	5.00	.000	.000
	6.00	1.000	.000
	7.00	.000	1.000

Block 0: Beginning Block

Classification Table[a,b]

Observed			Predicted Depression 1.00	Depression 2.00	Percentage Correct
Step 0	Depression	1.00	7914	0	100.0
		2.00	723	0	.0
	Overall Percentage				91.6

a. Constant is included in the model.

b. The cut value is .500

Variables in the Equation

		B	S.E.	Wald	df	Sig.	Exp(B)
Step 0	Constant	-2.393	.039	3793.581	1	.000	.091

Variables not in the Equation

			Score	df	Sig.
Step 0	Variables	Lifetimeabuseindex	403.012	7	<.001
		Lifetimeabuseindex(1)	4.406	1	.036
		Lifetimeabuseindex(2)	2.624	1	.105
		Lifetimeabuseindex(3)	64.923	1	<.001
		Lifetimeabuseindex(4)	.189	1	.664
		Lifetimeabuseindex(5)	28.404	1	<.001
		Lifetimeabuseindex(6)	16.537	1	<.001
		Lifetimeabuseindex(7)	198.833	1	<.001
	Overall Statistics		403.012	7	<.001

Block 1: Method = Enter

Omnibus Tests of Model Coefficients

		Chi-square	df	Sig.
Step 1	Step	333.040	7	<.001
	Block	333.040	7	<.001
	Model	333.040	7	<.001

Model Summary

Step	-2 Log likelihood	Cox & Snell R Square	Nagelkerke R Square
1	4637.335[a]	.038	.086

a. Estimation terminated at iteration number 6 because parameter estimates changed by less than .001.

Classification Table[a]

			Predicted		
			Depression		Percentage Correct
Observed			1.00	2.00	
Step 1	Depression	1.00	7914	0	100.0
		2.00	723	0	.0
	Overall Percentage				91.6

a. The cut value is .500

Variables in the Equation

		B	S.E.	Wald	df	Sig.
Step 1[a]	Lifetimeabuseindex			339.039	7	<.001
	Lifetimeabuseindex(1)	.896	.126	50.811	1	<.001
	Lifetimeabuseindex(2)	.936	.171	30.020	1	<.001
	Lifetimeabuseindex(3)	1.687	.149	128.010	1	<.001
	Lifetimeabuseindex(4)	.741	.140	28.099	1	<.001
	Lifetimeabuseindex(5)	1.351	.147	84.011	1	<.001
	Lifetimeabuseindex(6)	1.365	.185	54.210	1	<.001
	Lifetimeabuseindex(7)	2.147	.135	253.701	1	<.001
	Constant	-3.084	.070	1936.858	1	.000

Variables in the Equation

		Exp(B)	95% C.I.for EXP(B)	
			Lower	Upper
Step 1[a]	Lifetimeabuseindex			
	Lifetimeabuseindex(1)	2.451	1.915	3.136
	Lifetimeabuseindex(2)	2.550	1.824	3.564
	Lifetimeabuseindex(3)	5.404	4.034	7.238
	Lifetimeabuseindex(4)	2.097	1.595	2.758
	Lifetimeabuseindex(5)	3.861	2.892	5.154
	Lifetimeabuseindex(6)	3.918	2.724	5.635
	Lifetimeabuseindex(7)	8.557	6.570	11.144
	Constant	.046		

a. Variable(s) entered on step 1: Lifetimeabuseindex.

Logistic Regression

Notes

Output Created		05-OCT-2024 11:47:31
Comments		
Input	Data	/Users/stevenlc/Library/CloudStorage/OneDrive-Privat/ICloud filer/Doktorander/Rickard/Artikel 3/Revision art 3/Artikel3_revision.sav
	Active Dataset	DataSet7
	Filter	<none>
	Weight	<none>
	Split File	<none>
	N of Rows in Working Data File	10337
Missing Value Handling	Definition of Missing	User-defined missing values are treated as missing
Syntax		LOGISTIC REGRESSION VARIABLES HAD_probable_depression /METHOD=ENTER Lifetimeabuseindex Householddysf_parent Kon barnboendeny utbildningmammapappa_ny fodelselandmammapappa_ny alderskategorier /CONTRAST (Lifetimeabuseindex)=Indicator(1) /CONTRAST (Householddysf_parent)=Indicator(1) /CONTRAST (Kon)=Indicator(1) /CONTRAST (barnboendeny)=Indicator(1) /CONTRAST (utbildningmammapappa_ny)=Indicator(1) /CONTRAST (fodelselandmammapappa_ny)=Indicator(1) /CONTRAST (alderskategorier)=Indicator(1) /PRINT=CI(95) /CRITERIA=PIN(0.05) POUT(0.10) ITERATE(20) CUT(0.5).
Resources	Processor Time	00:00:00,46
	Elapsed Time	00:00:00,00

Case Processing Summary

Unweighted Cases[a]		N	Percent
Selected Cases	Included in Analysis	7227	69.9
	Missing Cases	3110	30.1
	Total	10337	100.0
Unselected Cases		0	.0
Total		10337	100.0

a. If weight is in effect, see classification table for the total number of cases.

Dependent Variable Encoding

Original Value	Internal Value
1.00	0
2.00	1

Categorical Variables Codings

		Frequency	Parameter coding (1)	(2)	(3)
Lifetimeabuseindex	.00	4185	.000	.000	.000
	1.00	827	1.000	.000	.000
	2.00	344	.000	1.000	.000
	3.00	263	.000	.000	1.000
	4.00	724	.000	.000	.000
	5.00	384	.000	.000	.000
	6.00	217	.000	.000	.000
	7.00	283	.000	.000	.000
Age categories	17-25	866	.000	.000	.000
	26-35	1143	1.000	.000	.000
	36-45	1368	.000	1.000	.000
	46-55	1439	.000	.000	1.000
	56-65	1494	.000	.000	.000
	66-74	917	.000	.000	.000
Parent education	At least one parent high school	3854	.000		
	Both parents below high school	3373	1.000		
Householddysf_parent	.00	5466	.000		
	1.00	1761	1.000		
Sex	Man	3250	.000		
	Kvinna	3977	1.000		
Immigrant status	At least one Nordic parent	6776	.000		
	Both parents born elsewhere	451	1.000		
Residence type	Owned home	5409	.000		
	Rental	1818	1.000		

Categorical Variables Codings

		Parameter coding (4)	(5)	(6)	(7)
Lifetimeabuseindex	.00	.000	.000	.000	.000
	1.00	.000	.000	.000	.000
	2.00	.000	.000	.000	.000
	3.00	.000	.000	.000	.000
	4.00	1.000	.000	.000	.000
	5.00	.000	1.000	.000	.000
	6.00	.000	.000	1.000	.000
	7.00	.000	.000	.000	1.000
Age categories	17-25	.000	.000		
	26-35	.000	.000		
	36-45	.000	.000		
	46-55	.000	.000		
	56-65	1.000	.000		
	66-74	.000	1.000		
Parent education	At least one parent high school				
	Both parents below high school				
Householddysf_parent	.00				
	1.00				
Sex	Man				
	Kvinna				
Immigrant status	At least one Nordic parent				
	Both parents born elsewhere				
Residence type	Owned home				
	Rental				

Block 0: Beginning Block

Classification Table[a,b]

Observed			Predicted Depression 1.00	2.00	Percentage Correct
Step 0	Depression	1.00	6667	0	100.0
		2.00	560	0	.0
	Overall Percentage				92.3

a. Constant is included in the model.

b. The cut value is .500

Variables in the Equation

		B	S.E.	Wald	df	Sig.	Exp(B)
Step 0	Constant	-2.477	.044	3169.629	1	.000	.084

Variables not in the Equation

			Score	df	Sig.
Step 0	Variables	Lifetimeabuseindex	314.822	7	<.001
		Lifetimeabuseindex(1)	3.188	1	.074
		Lifetimeabuseindex(2)	.806	1	.369
		Lifetimeabuseindex(3)	30.799	1	<.001
		Lifetimeabuseindex(4)	1.022	1	.312
		Lifetimeabuseindex(5)	28.559	1	<.001
		Lifetimeabuseindex(6)	9.868	1	.002
		Lifetimeabuseindex(7)	179.519	1	<.001
		Householddysf_parent(1)	19.012	1	<.001
		Sex(1)	1.497	1	.221
		Residence type(1)	.857	1	.355
		Parent education(1)	1.836	1	.175
		Immigrant status(1)	14.664	1	<.001
		Age categories	16.708	5	.005
		Age categories(1)	2.623	1	.105
		Age categories(2)	2.471	1	.116
		Age categories(3)	.076	1	.783
		Age categories(4)	7.836	1	.005
		Age categories(5)	4.504	1	.034
	Overall Statistics		338.923	17	<.001

Block 1: Method = Enter

Omnibus Tests of Model Coefficients

		Chi-square	df	Sig.
Step 1	Step	273.476	17	<.001
	Block	273.476	17	<.001
	Model	273.476	17	<.001

Model Summary

Step	-2 Log likelihood	Cox & Snell R Square	Nagelkerke R Square
1	3666.525[a]	.037	.088

a. Estimation terminated at iteration number 6 because parameter estimates changed by less than .001.

Classification Table[a]

Observed			Predicted: Depression 1.00	Predicted: Depression 2.00	Percentage Correct
Step 1	Depression	1.00	6667	0	100.0
		2.00	560	0	.0
	Overall Percentage				92.3

a. The cut value is .500

Variables in the Equation

		B	S.E.	Wald	df	Sig.
Step 1[a]	Lifetimeabuseindex			235.681	7	<.001
	Lifetimeabuseindex(1)	.854	.143	35.597	1	<.001
	Lifetimeabuseindex(2)	.842	.206	16.731	1	<.001
	Lifetimeabuseindex(3)	1.582	.190	69.416	1	<.001
	Lifetimeabuseindex(4)	.750	.153	23.894	1	<.001
	Lifetimeabuseindex(5)	1.381	.165	70.080	1	<.001
	Lifetimeabuseindex(6)	1.262	.217	33.852	1	<.001
	Lifetimeabuseindex(7)	2.261	.163	192.703	1	<.001
	Householddysf_parent(1)	-.112	.109	1.051	1	.305
	Sex(1)	-.002	.092	.000	1	.985
	Residence type(1)	-.065	.108	.362	1	.548
	Parent education(1)	.089	.104	.730	1	.393
	Immigrant status(1)	.370	.159	5.437	1	.020
	Age categories			14.924	5	.011
	Age categories(1)	-.165	.163	1.029	1	.310
	Age categories(2)	-.208	.159	1.710	1	.191
	Age categories(3)	-.429	.166	6.641	1	.010
	Age categories(4)	-.612	.180	11.625	1	<.001
	Age categories(5)	-.497	.204	5.930	1	.015
	Constant	-2.822	.149	360.397	1	<.001

Variables in the Equation

		Exp(B)	95% C.I.for EXP(B) Lower	Upper
Step 1[a]	Lifetimeabuseindex			
	Lifetimeabuseindex(1)	2.350	1.775	3.111
	Lifetimeabuseindex(2)	2.321	1.550	3.473
	Lifetimeabuseindex(3)	4.862	3.352	7.054
	Lifetimeabuseindex(4)	2.117	1.567	2.859
	Lifetimeabuseindex(5)	3.979	2.880	5.498
	Lifetimeabuseindex(6)	3.533	2.309	5.405
	Lifetimeabuseindex(7)	9.595	6.973	13.205
	Householddysf_parent(1)	.894	.722	1.107
	Sex(1)	.998	.834	1.195
	Residence type(1)	.937	.758	1.158
	Parent education(1)	1.093	.891	1.341
	Immigrant status(1)	1.448	1.061	1.978
	Age categories			
	Age categories(1)	.848	.617	1.166
	Age categories(2)	.812	.595	1.109
	Age categories(3)	.651	.470	.902
	Age categories(4)	.542	.381	.771
	Age categories(5)	.608	.408	.908
	Constant	.059		

a. Variable(s) entered on step 1: Lifetimeabuseindex, Householddysf_parent, Sex, Residence type, Parent education, Immigrant status, Age categories.

Logistic Regression

Notes

Output Created		05-OCT-2024 11:47:31
Comments		
Input	Data	/Users/stevenlc/Library/CloudStorage/OneDrive-Privat/ICloud filer/Doktorander/Rickard/Artikel 3/Revision art 3/Artikel3_revision.sav
	Active Dataset	DataSet7
	Filter	<none>
	Weight	<none>
	Split File	<none>
	N of Rows in Working Data File	10337
Missing Value Handling	Definition of Missing	User-defined missing values are treated as missing
Syntax		LOGISTIC REGRESSION VARIABLES HAD_probable_ångest /METHOD=ENTER Maltreatment_0to3 /CONTRAST (Maltreatment_0to3)=Indicator(1) /PRINT=CI(95) /CRITERIA=PIN(0.05) POUT(0.10) ITERATE(20) CUT(0.5).
Resources	Processor Time	00:00:00,40
	Elapsed Time	00:00:01,00

Case Processing Summary

Unweighted Cases[a]		N	Percent
Selected Cases	Included in Analysis	8835	85.5
	Missing Cases	1502	14.5
	Total	10337	100.0
Unselected Cases		0	.0
Total		10337	100.0

a. If weight is in effect, see classification table for the total number of cases.

Dependent Variable Encoding

Original Value	Internal Value
1.00	0
2.00	1

Categorical Variables Codings

		Frequency	Parameter coding (1)	(2)	(3)
Maltreatment_0to3	.00	5825	.000	.000	.000
	1.00	1528	1.000	.000	.000
	2.00	723	.000	1.000	.000
	3.00	759	.000	.000	1.000

Block 0: Beginning Block

Classification Table[a,b]

Observed			Predicted Ångest 1.00	2.00	Percentage Correct
Step 0	Ångest	1.00	8398	0	100.0
		2.00	437	0	.0
	Overall Percentage				95.1

a. Constant is included in the model.

b. The cut value is .500

Variables in the Equation

		B	S.E.	Wald	df	Sig.	Exp(B)
Step 0	Constant	-2.956	.049	3629.154	1	.000	.052

Variables not in the Equation

			Score	df	Sig.
Step 0	Variables	Maltreatment_0to3	297.425	3	<.001
		Maltreatment_0to3(1)	7.723	1	.005
		Maltreatment_0to3(2)	11.859	1	<.001
		Maltreatment_0to3(3)	239.883	1	<.001
	Overall Statistics		297.425	3	<.001

Block 1: Method = Enter

Omnibus Tests of Model Coefficients

		Chi-square	df	Sig.
Step 1	Step	227.017	3	<.001
	Block	227.017	3	<.001
	Model	227.017	3	<.001

Model Summary

Step	-2 Log likelihood	Cox & Snell R Square	Nagelkerke R Square
1	3252.722[a]	.025	.078

a. Estimation terminated at iteration number 6 because parameter estimates changed by less than .001.

Classification Table[a]

Observed			Predicted		
			Ångest		Percentage Correct
			1.00	2.00	
Step 1	Ångest	1.00	8398	0	100.0
		2.00	437	0	.0
	Overall Percentage				95.1

a. The cut value is .500

Variables in the Equation

		B	S.E.	Wald	df	Sig.
Step 1[a]	Maltreatment_0to3			243.712	3	<.001
	Maltreatment_0to3(1)	.882	.132	44.511	1	<.001
	Maltreatment_0to3(2)	1.076	.162	44.315	1	<.001
	Maltreatment_0to3(3)	1.959	.126	240.160	1	<.001
	Constant	-3.573	.080	1974.809	1	.000

Variables in the Equation

		Exp(B)	95% C.I.for EXP(B)	
			Lower	Upper
Step 1[a]	Maltreatment_0to3			
	Maltreatment_0to3(1)	2.416	1.864	3.130
	Maltreatment_0to3(2)	2.934	2.137	4.028
	Maltreatment_0to3(3)	7.093	5.537	9.088
	Constant	.028		

a. Variable(s) entered on step 1: Maltreatment_0to3.

Logistic Regression

Notes

Output Created		05-OCT-2024 11:47:32
Comments		
Input	Data	/Users/stevenlc/Library/CloudStorage/OneDrive-Privat/ICloud filer/Doktorander/Rickard/Artikel 3/Revision art 3/Artikel3_revision.sav
	Active Dataset	DataSet7
	Filter	<none>
	Weight	<none>
	Split File	<none>
	N of Rows in Working Data File	10337
Missing Value Handling	Definition of Missing	User-defined missing values are treated as missing
Syntax		LOGISTIC REGRESSION VARIABLES HAD_probable_ångest /METHOD=ENTER Maltreatment_0to3 Householddysf_parent Kon barnboendeny utbildningmammapappa_ny fodelselandmammapappa_ny alderskategorier /CONTRAST (Maltreatment_0to3)=Indicator(1) /CONTRAST (Householddysf_parent)=Indicator(1) /CONTRAST (Kon)=Indicator(1) /CONTRAST (barnboendeny)=Indicator(1) /CONTRAST (utbildningmammapappa_ny)=Indicator(1) /CONTRAST (fodelselandmammapappa_ny)=Indicator(1) /CONTRAST (alderskategorier)=Indicator(1) /PRINT=CI(95) /CRITERIA=PIN(0.05) POUT(0.10) ITERATE(20) CUT(0.5).
Resources	Processor Time	00:00:00,45
	Elapsed Time	00:00:00,00

Case Processing Summary

Unweighted Cases[a]		N	Percent
Selected Cases	Included in Analysis	7357	71.2
	Missing Cases	2980	28.8
	Total	10337	100.0
Unselected Cases		0	.0
Total		10337	100.0

a. If weight is in effect, see classification table for the total number of cases.

Dependent Variable Encoding

Original Value	Internal Value
1.00	0
2.00	1

Categorical Variables Codings

			Parameter coding		
		Frequency	(1)	(2)	(3)
Age categories	17-25	874	.000	.000	.000
	26-35	1158	1.000	.000	.000
	36-45	1390	.000	1.000	.000
	46-55	1452	.000	.000	1.000
	56-65	1534	.000	.000	.000
	66-74	949	.000	.000	.000
Maltreatment_0to3	.00	4976	.000	.000	.000
	1.00	1244	1.000	.000	.000
	2.00	577	.000	1.000	.000
	3.00	560	.000	.000	1.000
Parent education	At least one parent high school	3932	.000		
	Both parents below high school	3425	1.000		
Householddysf_parent	.00	5548	.000		
	1.00	1809	1.000		
Sex	Man	3302	.000		
	Kvinna	4055	1.000		
Immigrant status	At least one Nordic parent	6891	.000		
	Both parents born elsewhere	466	1.000		
Residence type	Owned home	5502	.000		
	Rental	1855	1.000		

Categorical Variables Codings

		Parameter coding (4)	Parameter coding (5)
Age categories	17-25	.000	.000
	26-35	.000	.000
	36-45	.000	.000
	46-55	.000	.000
	56-65	1.000	.000
	66-74	.000	1.000
Maltreatment_0to3	.00		
	1.00		
	2.00		
	3.00		
Parent education	At least one parent high school		
	Both parents below high school		
Householddysf_parent	.00		
	1.00		
Sex	Man		
	Kvinna		
Immigrant status	At least one Nordic parent		
	Both parents born elsewhere		
Residence type	Owned home		
	Rental		

Block 0: Beginning Block

Classification Table[a,b]

Observed			Predicted Ångest 1.00	Predicted Ångest 2.00	Percentage Correct
Step 0	Ångest	1.00	7011	0	100.0
		2.00	346	0	.0
	Overall Percentage				95.3

a. Constant is included in the model.

b. The cut value is .500

Variables in the Equation

		B	S.E.	Wald	df	Sig.	Exp(B)
Step 0	Constant	-3.009	.055	2984.977	1	.000	.049

Variables not in the Equation

			Score	df	Sig.
Step 0	Variables	Maltreatment_0to3	209.875	3	<.001
		Maltreatment_0to3(1)	9.973	1	.002
		Maltreatment_0to3(2)	11.933	1	<.001
		Maltreatment_0to3(3)	158.705	1	<.001
		Householddysf_parent(1)	68.939	1	<.001
		Sex(1)	49.113	1	<.001
		Residence type(1)	3.504	1	.061
		Parent education(1)	15.866	1	<.001
		Immigrant status(1)	6.280	1	.012
		Age categories	98.328	5	<.001
		Age categories(1)	9.647	1	.002
		Age categories(2)	.225	1	.635
		Age categories(3)	1.139	1	.286
		Age categories(4)	25.354	1	<.001
		Age categories(5)	25.328	1	<.001
	Overall Statistics		354.363	13	<.001

Block 1: Method = Enter

Omnibus Tests of Model Coefficients

		Chi-square	df	Sig.
Step 1	Step	311.994	13	<.001
	Block	311.994	13	<.001
	Model	311.994	13	<.001

Model Summary

Step	-2 Log likelihood	Cox & Snell R Square	Nagelkerke R Square
1	2478.895[a]	.042	.132

a. Estimation terminated at iteration number 7 because parameter estimates changed by less than .001.

Classification Table[a]

			Predicted		
			Ångest		Percentage Correct
	Observed		1.00	2.00	
Step 1	Ångest	1.00	7011	0	100.0
		2.00	346	0	.0
	Overall Percentage				95.3

a. The cut value is .500

Variables in the Equation

		B	S.E.	Wald	df	Sig.
Step 1[a]	Maltreatment_0to3			130.761	3	<.001
	Maltreatment_0to3(1)	.859	.148	33.536	1	<.001
	Maltreatment_0to3(2)	1.062	.187	32.251	1	<.001
	Maltreatment_0to3(3)	1.832	.164	125.179	1	<.001
	Householddysf_parent(1)	.213	.128	2.753	1	.097
	Sex(1)	.711	.127	31.445	1	<.001
	Residence type(1)	.088	.135	.426	1	.514
	Parent education(1)	.017	.135	.016	1	.899
	Immigrant status(1)	.124	.202	.376	1	.540
	Age categories			70.709	5	<.001
	Age categories(1)	-.516	.173	8.923	1	.003
	Age categories(2)	-.927	.183	25.739	1	<.001
	Age categories(3)	-.856	.183	21.849	1	<.001
	Age categories(4)	-1.647	.230	51.473	1	<.001
	Age categories(5)	-1.926	.315	37.486	1	<.001
	Constant	-3.265	.167	381.988	1	<.001

Variables in the Equation

		Exp(B)	95% C.I.for EXP(B)	
			Lower	Upper
Step 1[a]	Maltreatment_0to3			
	Maltreatment_0to3(1)	2.360	1.765	3.156
	Maltreatment_0to3(2)	2.893	2.005	4.174
	Maltreatment_0to3(3)	6.244	4.530	8.606
	Householddysf_parent(1)	1.237	.962	1.591
	Sex(1)	2.036	1.588	2.610
	Residence type(1)	1.092	.838	1.422
	Parent education(1)	1.017	.781	1.324
	Immigrant status(1)	1.132	.762	1.682
	Age categories			
	Age categories(1)	.597	.425	.837
	Age categories(2)	.396	.276	.566
	Age categories(3)	.425	.297	.608
	Age categories(4)	.193	.123	.302
	Age categories(5)	.146	.079	.270
	Constant	.038		

a. Variable(s) entered on step 1: Maltreatment_0to3, Householddysf_parent, Sex, Residence type, Parent education, Immigrant status, Age categories.

Logistic Regression

Notes

Output Created		05-OCT-2024 11:47:32
Comments		
Input	Data	/Users/stevenlc/Library/CloudStorage/OneDrive-Privat/ICloud filer/Doktorander/Rickard/Artikel 3/Revision art 3/Artikel3_revision.sav
	Active Dataset	DataSet7
	Filter	<none>
	Weight	<none>
	Split File	<none>
	N of Rows in Working Data File	10337
Missing Value Handling	Definition of Missing	User-defined missing values are treated as missing
Syntax		LOGISTIC REGRESSION VARIABLES HAD_probable_ångest /METHOD=ENTER Lifetimeabuseindex /CONTRAST (Lifetimeabuseindex)=Indicator(1) /PRINT=CI(95) /CRITERIA=PIN(0.05) POUT(0.10) ITERATE(20) CUT(0.5).
Resources	Processor Time	00:00:00,40
	Elapsed Time	00:00:01,00

Case Processing Summary

Unweighted Cases[a]		N	Percent
Selected Cases	Included in Analysis	8640	83.6
	Missing Cases	1697	16.4
	Total	10337	100.0
Unselected Cases		0	.0
Total		10337	100.0

a. If weight is in effect, see classification table for the total number of cases.

Dependent Variable Encoding

Original Value	Internal Value
1.00	0
2.00	1

Categorical Variables Codings

			Parameter coding				
		Frequency	(1)	(2)	(3)	(4)	(5)
Lifetimeabuseindex	.00	4861	.000	.000	.000	.000	.000
	1.00	1021	1.000	.000	.000	.000	.000
	2.00	445	.000	1.000	.000	.000	.000
	3.00	367	.000	.000	1.000	.000	.000
	4.00	847	.000	.000	.000	1.000	.000
	5.00	466	.000	.000	.000	.000	1.000
	6.00	260	.000	.000	.000	.000	.000
	7.00	373	.000	.000	.000	.000	.000

Categorical Variables Codings

		Parameter coding	
		(6)	(7)
Lifetimeabuseindex	.00	.000	.000
	1.00	.000	.000
	2.00	.000	.000
	3.00	.000	.000
	4.00	.000	.000
	5.00	.000	.000
	6.00	1.000	.000
	7.00	.000	1.000

Block 0: Beginning Block

Classification Table[a,b]

			Predicted		
			Ångest		Percentage Correct
	Observed		1.00	2.00	
Step 0	Ångest	1.00	8212	0	100.0
		2.00	428	0	.0
	Overall Percentage				95.0

a. Constant is included in the model.

b. The cut value is .500

Variables in the Equation

		B	S.E.	Wald	df	Sig.	Exp(B)
Step 0	Constant	-2.954	.050	3550.317	1	.000	.052

Variables not in the Equation

			Score	df	Sig.
Step 0	Variables	Lifetimeabuseindex	409.225	7	<.001
		Lifetimeabuseindex(1)	.302	1	.583
		Lifetimeabuseindex(2)	.055	1	.815
		Lifetimeabuseindex(3)	31.474	1	<.001
		Lifetimeabuseindex(4)	7.155	1	.007
		Lifetimeabuseindex(5)	27.555	1	<.001
		Lifetimeabuseindex(6)	30.791	1	<.001
		Lifetimeabuseindex(7)	240.132	1	<.001
	Overall Statistics		409.225	7	<.001

Block 1: Method = Enter

Omnibus Tests of Model Coefficients

		Chi-square	df	Sig.
Step 1	Step	310.097	7	<.001
	Block	310.097	7	<.001
	Model	310.097	7	<.001

Model Summary

Step	-2 Log likelihood	Cox & Snell R Square	Nagelkerke R Square
1	3096.652[a]	.035	.108

a. Estimation terminated at iteration number 7 because parameter estimates changed by less than .001.

Classification Table[a]

	Observed		Predicted Ångest 1.00	Predicted Ångest 2.00	Percentage Correct
Step 1	Ångest	1.00	8212	0	100.0
		2.00	428	0	.0
	Overall Percentage				95.0

a. The cut value is .500

Variables in the Equation

		B	S.E.	Wald	df	Sig.
Step 1[a]	Lifetimeabuseindex			309.899	7	<.001
	Lifetimeabuseindex(1)	.832	.180	21.279	1	<.001
	Lifetimeabuseindex(2)	.858	.245	12.226	1	<.001
	Lifetimeabuseindex(3)	1.790	.194	85.035	1	<.001
	Lifetimeabuseindex(4)	1.253	.169	54.644	1	<.001
	Lifetimeabuseindex(5)	1.675	.184	82.859	1	<.001
	Lifetimeabuseindex(6)	1.899	.214	78.695	1	<.001
	Lifetimeabuseindex(7)	2.596	.161	260.878	1	<.001
	Constant	-3.863	.101	1461.610	1	.000

Variables in the Equation

			95% C.I.for EXP(B)	
		Exp(B)	Lower	Upper
Step 1[a]	Lifetimeabuseindex			
	Lifetimeabuseindex(1)	2.297	1.613	3.271
	Lifetimeabuseindex(2)	2.358	1.458	3.814
	Lifetimeabuseindex(3)	5.988	4.093	8.759
	Lifetimeabuseindex(4)	3.500	2.511	4.879
	Lifetimeabuseindex(5)	5.341	3.723	7.660
	Lifetimeabuseindex(6)	6.682	4.392	10.166
	Lifetimeabuseindex(7)	13.416	9.790	18.385
	Constant	.021		

a. Variable(s) entered on step 1: Lifetimeabuseindex.

Logistic Regression

Notes

Output Created		05-OCT-2024 11:47:33
Comments		
Input	Data	/Users/stevenlc/Library/CloudStorage/OneDrive-Privat/ICloud filer/Doktorander/Rickard/Artikel 3/Revision art 3/Artikel3_revision.sav
	Active Dataset	DataSet7
	Filter	<none>
	Weight	<none>
	Split File	<none>
	N of Rows in Working Data File	10337
Missing Value Handling	Definition of Missing	User-defined missing values are treated as missing
Syntax		LOGISTIC REGRESSION VARIABLES HAD_probable_ångest<br>/METHOD=ENTER Lifetimeabuseindex Householddysf_parent Kon barnboendeny utbildningmammapappa_ny fodelselandmammapappa_ny alderskategorier<br>/CONTRAST (Lifetimeabuseindex)=Indicator(1)<br>/CONTRAST (Householddysf_parent)=Indicator(1)<br>/CONTRAST (Kon)=Indicator(1)<br>/CONTRAST (barnboendeny)=Indicator(1)<br>/CONTRAST (utbildningmammapappa_ny)=Indicator(1)<br>/CONTRAST (fodelselandmammapappa_ny)=Indicator(1)<br>/CONTRAST (alderskategorier)=Indicator(1)<br>/PRINT=CI(95)<br>/CRITERIA=PIN(0.05) POUT(0.10) ITERATE(20) CUT(0.5).
Resources	Processor Time	00:00:00,48
	Elapsed Time	00:00:00,00

Case Processing Summary

Unweighted Cases[a]		N	Percent
Selected Cases	Included in Analysis	7214	69.8
	Missing Cases	3123	30.2
	Total	10337	100.0
Unselected Cases		0	.0
Total		10337	100.0

a. If weight is in effect, see classification table for the total number of cases.

Dependent Variable Encoding

Original Value	Internal Value
1.00	0
2.00	1

Categorical Variables Codings

			Parameter coding		
		Frequency	(1)	(2)	(3)
Lifetimeabuseindex	.00	4173	.000	.000	.000
	1.00	832	1.000	.000	.000
	2.00	349	.000	1.000	.000
	3.00	266	.000	.000	1.000
	4.00	714	.000	.000	.000
	5.00	385	.000	.000	.000
	6.00	214	.000	.000	.000
	7.00	281	.000	.000	.000
Age categories	17-25	854	.000	.000	.000
	26-35	1136	1.000	.000	.000
	36-45	1364	.000	1.000	.000
	46-55	1420	.000	.000	1.000
	56-65	1509	.000	.000	.000
	66-74	931	.000	.000	.000
Parent education	At least one parent high school	3841	.000		
	Both parents below high school	3373	1.000		
Householddysf_parent	.00	5449	.000		
	1.00	1765	1.000		
Sex	Man	3233	.000		
	Kvinna	3981	1.000		
Immigrant status	At least one Nordic parent	6757	.000		
	Both parents born elsewhere	457	1.000		
Residence type	Owned home	5388	.000		
	Rental	1826	1.000		

Categorical Variables Codings

		Parameter coding (4)	(5)	(6)	(7)
Lifetimeabuseindex	.00	.000	.000	.000	.000
	1.00	.000	.000	.000	.000
	2.00	.000	.000	.000	.000
	3.00	.000	.000	.000	.000
	4.00	1.000	.000	.000	.000
	5.00	.000	1.000	.000	.000
	6.00	.000	.000	1.000	.000
	7.00	.000	.000	.000	1.000
Age categories	17-25	.000	.000		
	26-35	.000	.000		
	36-45	.000	.000		
	46-55	.000	.000		
	56-65	1.000	.000		
	66-74	.000	1.000		
Parent education	At least one parent high school				
	Both parents below high school				
Householddysf_parent	.00				
	1.00				
Sex	Man				
	Kvinna				
Immigrant status	At least one Nordic parent				
	Both parents born elsewhere				
Residence type	Owned home				
	Rental				

Block 0: Beginning Block

Classification Table[a,b]

Observed			Predicted Ångest 1.00	2.00	Percentage Correct
Step 0	Ångest	1.00	6877	0	100.0
		2.00	337	0	.0
	Overall Percentage				95.3

a. Constant is included in the model.

b. The cut value is .500

Variables in the Equation

		B	S.E.	Wald	df	Sig.	Exp(B)
Step 0	Constant	-3.016	.056	2921.956	1	.000	.049

Variables not in the Equation

			Score	df	Sig.
Step 0	Variables	Lifetimeabuseindex	309.735	7	<.001
		Lifetimeabuseindex(1)	.023	1	.880
		Lifetimeabuseindex(2)	.195	1	.659
		Lifetimeabuseindex(3)	9.800	1	.002
		Lifetimeabuseindex(4)	7.487	1	.006
		Lifetimeabuseindex(5)	27.210	1	<.001
		Lifetimeabuseindex(6)	21.205	1	<.001
		Lifetimeabuseindex(7)	190.571	1	<.001
		Householddysf_parent(1)	63.808	1	<.001
		Sex(1)	45.354	1	<.001
		Residence type(1)	2.254	1	.133
		Parent education(1)	17.648	1	<.001
		Immigrant status(1)	7.122	1	.008
		Age categories	96.901	5	<.001
		Age categories(1)	9.321	1	.002
		Age categories(2)	.150	1	.698
		Age categories(3)	.429	1	.513
		Age categories(4)	23.704	1	<.001
		Age categories(5)	24.086	1	<.001
	Overall Statistics		433.823	17	<.001

Block 1: Method = Enter

Omnibus Tests of Model Coefficients

		Chi-square	df	Sig.
Step 1	Step	358.728	17	<.001
	Block	358.728	17	<.001
	Model	358.728	17	<.001

Model Summary

Step	-2 Log likelihood	Cox & Snell R Square	Nagelkerke R Square
1	2364.209 [a]	.049	.154

a. Estimation terminated at iteration number 7 because parameter estimates changed by less than .001.

Classification Table[a]

Observed			Predicted Ångest 1.00	2.00	Percentage Correct
Step 1	Ångest	1.00	6876	1	100.0
		2.00	336	1	.3
	Overall Percentage				95.3

a. The cut value is .500

Variables in the Equation

		B	S.E.	Wald	df	Sig.
Step 1[a]	Lifetimeabuseindex			181.418	7	<.001
	Lifetimeabuseindex(1)	.807	.202	16.015	1	<.001
	Lifetimeabuseindex(2)	1.002	.271	13.614	1	<.001
	Lifetimeabuseindex(3)	1.553	.257	36.443	1	<.001
	Lifetimeabuseindex(4)	1.198	.188	40.724	1	<.001
	Lifetimeabuseindex(5)	1.606	.207	60.422	1	<.001
	Lifetimeabuseindex(6)	1.681	.252	44.494	1	<.001
	Lifetimeabuseindex(7)	2.486	.199	156.295	1	<.001
	Householddysf_parent(1)	.164	.131	1.569	1	.210
	Sex(1)	.672	.129	27.183	1	<.001
	Residence type(1)	.046	.139	.110	1	.741
	Parent education(1)	.048	.138	.124	1	.725
	Immigrant status(1)	.190	.205	.863	1	.353
	Age categories			67.898	5	<.001
	Age categories(1)	-.583	.177	10.889	1	<.001
	Age categories(2)	-1.021	.186	30.073	1	<.001
	Age categories(3)	-1.003	.189	28.027	1	<.001
	Age categories(4)	-1.631	.232	49.247	1	<.001
	Age categories(5)	-1.826	.317	33.214	1	<.001
	Constant	-3.459	.180	370.220	1	<.001

Variables in the Equation

		Exp(B)	95% C.I.for EXP(B) Lower	Upper
Step 1[a]	Lifetimeabuseindex			
	Lifetimeabuseindex(1)	2.241	1.509	3.326
	Lifetimeabuseindex(2)	2.723	1.599	4.636
	Lifetimeabuseindex(3)	4.724	2.854	7.820
	Lifetimeabuseindex(4)	3.313	2.293	4.787
	Lifetimeabuseindex(5)	4.982	3.323	7.468
	Lifetimeabuseindex(6)	5.372	3.278	8.803
	Lifetimeabuseindex(7)	12.017	8.138	17.744
	Householddysf_parent(1)	1.179	.911	1.525
	Sex(1)	1.958	1.521	2.520
	Residence type(1)	1.047	.798	1.375
	Parent education(1)	1.050	.801	1.376
	Immigrant status(1)	1.210	.810	1.807
	Age categories			
	Age categories(1)	.558	.395	.789
	Age categories(2)	.360	.250	.519
	Age categories(3)	.367	.253	.532
	Age categories(4)	.196	.124	.309
	Age categories(5)	.161	.087	.300
	Constant	.031		

a. Variable(s) entered on step 1: Lifetimeabuseindex, Householddysf_parent, Sex, Residence type, Parent education, Immigrant status, Age categories.

Logistic Regression

Notes

Output Created		05-OCT-2024 11:47:33
Comments		
Input	Data	/Users/stevenlc/Library/CloudStorage/OneDrive-Privat/ICloud filer/Doktorander/Rickard/Artikel 3/Revision art 3/Artikel3_revision.sav
	Active Dataset	DataSet7
	Filter	<none>
	Weight	<none>
	Split File	<none>
	N of Rows in Working Data File	10337
Missing Value Handling	Definition of Missing	User-defined missing values are treated as missing
Syntax		LOGISTIC REGRESSION VARIABLES Any_selfharm /METHOD=ENTER Maltreatment_0to3 /CONTRAST (Maltreatment_0to3)=Indicator(1) /PRINT=CI(95) /CRITERIA=PIN(0.05) POUT(0.10) ITERATE(20) CUT(0.5).
Resources	Processor Time	00:00:00,38
	Elapsed Time	00:00:00,00

Case Processing Summary

Unweighted Cases[a]		N	Percent
Selected Cases	Included in Analysis	9123	88.3
	Missing Cases	1214	11.7
	Total	10337	100.0
Unselected Cases		0	.0
Total		10337	100.0

a. If weight is in effect, see classification table for the total number of cases.

Dependent Variable Encoding

Original Value	Internal Value
1.00	0
2.00	1

Categorical Variables Codings

		Frequency	Parameter coding (1)	(2)	(3)
Maltreatment_0to3	.00	6019	.000	.000	.000
	1.00	1576	1.000	.000	.000
	2.00	741	.000	1.000	.000
	3.00	787	.000	.000	1.000

Block 0: Beginning Block

Classification Table[a,b]

Observed			Predicted Self harm 1.00	2.00	Percentage Correct
Step 0	Self harm	1.00	8043	0	100.0
		2.00	1080	0	.0
	Overall Percentage				88.2

a. Constant is included in the model.

b. The cut value is .500

Variables in the Equation

		B	S.E.	Wald	df	Sig.	Exp(B)
Step 0	Constant	-2.008	.032	3838.511	1	.000	.134

Variables not in the Equation

			Score	df	Sig.
Step 0	Variables	Maltreatment_0to3	657.814	3	<.001
		Maltreatment_0to3(1)	25.091	1	<.001
		Maltreatment_0to3(2)	39.950	1	<.001
		Maltreatment_0to3(3)	495.453	1	<.001
	Overall Statistics		657.814	3	<.001

Block 1: Method = Enter

Omnibus Tests of Model Coefficients

		Chi-square	df	Sig.
Step 1	Step	537.418	3	<.001
	Block	537.418	3	<.001
	Model	537.418	3	<.001

Model Summary

Step	-2 Log likelihood	Cox & Snell R Square	Nagelkerke R Square
1	6098.452[a]	.057	.111

a. Estimation terminated at iteration number 5 because parameter estimates changed by less than .001.

Classification Table[a]

Observed			Predicted: Self harm 1.00	Predicted: Self harm 2.00	Percentage Correct
Step 1	Self harm	1.00	8043	0	100.0
		2.00	1080	0	.0
	Overall Percentage				88.2

a. The cut value is .500

Variables in the Equation

		B	S.E.	Wald	df	Sig.
Step 1[a]	Maltreatment_0to3			552.030	3	<.001
	Maltreatment_0to3(1)	.929	.086	115.603	1	<.001
	Maltreatment_0to3(2)	1.173	.107	120.832	1	<.001
	Maltreatment_0to3(3)	2.061	.090	522.806	1	<.001
	Constant	-2.621	.051	2613.257	1	.000

Variables in the Equation

		Exp(B)	95% C.I.for EXP(B) Lower	95% C.I.for EXP(B) Upper
Step 1[a]	Maltreatment_0to3			
	Maltreatment_0to3(1)	2.531	2.137	2.998
	Maltreatment_0to3(2)	3.232	2.622	3.984
	Maltreatment_0to3(3)	7.851	6.580	9.367
	Constant	.073		

a. Variable(s) entered on step 1: Maltreatment_0to3.

Logistic Regression

Notes

Output Created		05-OCT-2024 11:47:33
Comments		
Input	Data	/Users/stevenlc/Library/CloudStorage/OneDrive-Privat/ICloud filer/Doktorander/Rickard/Artikel 3/Revision art 3/Artikel3_revision.sav
	Active Dataset	DataSet7
	Filter	<none>
	Weight	<none>
	Split File	<none>
	N of Rows in Working Data File	10337
Missing Value Handling	Definition of Missing	User-defined missing values are treated as missing
Syntax		LOGISTIC REGRESSION VARIABLES Any_selfharm /METHOD=ENTER Maltreatment_0to3 Householddysf_parent Kon barnboendeny utbildningmammapappa_ny fodelselandmammapappa_ny alderskategorier /CONTRAST (Maltreatment_0to3)=Indicator(1) /CONTRAST (Householddysf_parent)=Indicator(1) /CONTRAST (Kon)=Indicator(1) /CONTRAST (barnboendeny)=Indicator(1) /CONTRAST (utbildningmammapappa_ny)=Indicator(1) /CONTRAST (fodelselandmammapappa_ny)=Indicator(1) /CONTRAST (alderskategorier)=Indicator(1) /PRINT=CI(95) /CRITERIA=PIN(0.05) POUT(0.10) ITERATE(20) CUT(0.5).
Resources	Processor Time	00:00:00,44
	Elapsed Time	00:00:01,00

Case Processing Summary

Unweighted Cases[a]		N	Percent
Selected Cases	Included in Analysis	7595	73.5
	Missing Cases	2742	26.5
	Total	10337	100.0
Unselected Cases		0	.0
Total		10337	100.0

a. If weight is in effect, see classification table for the total number of cases.

Dependent Variable Encoding

Original Value	Internal Value
1.00	0
2.00	1

Categorical Variables Codings

		Frequency	Parameter coding (1)	(2)	(3)
Age categories	17-25	908	.000	.000	.000
	26-35	1188	1.000	.000	.000
	36-45	1426	.000	1.000	.000
	46-55	1506	.000	.000	1.000
	56-65	1583	.000	.000	.000
	66-74	984	.000	.000	.000
Maltreatment_0to3	.00	5140	.000	.000	.000
	1.00	1283	1.000	.000	.000
	2.00	588	.000	1.000	.000
	3.00	584	.000	.000	1.000
Parent education	At least one parent high school	4049	.000		
	Both parents below high school	3546	1.000		
Householddysf_parent	.00	5741	.000		
	1.00	1854	1.000		
Sex	Man	3401	.000		
	Kvinna	4194	1.000		
Immigrant status	At least one Nordic parent	7115	.000		
	Both parents born elsewhere	480	1.000		
Residence type	Owned home	5686	.000		
	Rental	1909	1.000		

Categorical Variables Codings

		Parameter coding (4)	Parameter coding (5)
Age categories	17-25	.000	.000
	26-35	.000	.000
	36-45	.000	.000
	46-55	.000	.000
	56-65	1.000	.000
	66-74	.000	1.000
Maltreatment_0to3	.00		
	1.00		
	2.00		
	3.00		
Parent education	At least one parent high school		
	Both parents below high school		
Householddysf_parent	.00		
	1.00		
Sex	Man		
	Kvinna		
Immigrant status	At least one Nordic parent		
	Both parents born elsewhere		
Residence type	Owned home		
	Rental		

Block 0: Beginning Block

Classification Table[a,b]

	Observed		Predicted Self harm 1.00	Predicted Self harm 2.00	Predicted Percentage Correct
Step 0	Self harm	1.00	6740	0	100.0
		2.00	855	0	.0
	Overall Percentage				88.7

a. Constant is included in the model.

b. The cut value is .500

Variables in the Equation

		B	S.E.	Wald	df	Sig.	Exp(B)
Step 0	Constant	-2.065	.036	3234.580	1	.000	.127

Variables not in the Equation

			Score	df	Sig.
Step 0	Variables	Maltreatment_0to3	468.941	3	<.001
		Maltreatment_0to3(1)	30.040	1	<.001
		Maltreatment_0to3(2)	24.997	1	<.001
		Maltreatment_0to3(3)	344.731	1	<.001
		Householddysf_parent(1)	159.187	1	<.001
		Sex(1)	56.397	1	<.001
		Residence type(1)	.008	1	.927
		Parent education(1)	108.563	1	<.001
		Immigrant status(1)	.564	1	.452
		Age categories	280.817	5	<.001
		Age categories(1)	70.917	1	<.001
		Age categories(2)	.775	1	.379
		Age categories(3)	3.164	1	.075
		Age categories(4)	63.567	1	<.001
		Age categories(5)	76.248	1	<.001
	Overall Statistics		833.795	13	<.001

Block 1: Method = Enter

Omnibus Tests of Model Coefficients

		Chi-square	df	Sig.
Step 1	Step	783.590	13	<.001
	Block	783.590	13	<.001
	Model	783.590	13	<.001

Model Summary

Step	-2 Log likelihood	Cox & Snell R Square	Nagelkerke R Square
1	4561.215[a]	.098	.194

a. Estimation terminated at iteration number 6 because parameter estimates changed by less than .001.

Classification Table[a]

			Predicted		
			Self harm		Percentage Correct
Observed			1.00	2.00	
Step 1	Self harm	1.00	6699	41	99.4
		2.00	784	71	8.3
	Overall Percentage				89.1

a. The cut value is .500

Variables in the Equation

		B	S.E.	Wald	df	Sig.
Step 1[a]	Maltreatment_0to3			360.965	3	<.001
	Maltreatment_0to3(1)	.972	.099	96.014	1	<.001
	Maltreatment_0to3(2)	1.215	.130	86.848	1	<.001
	Maltreatment_0to3(3)	2.182	.119	335.433	1	<.001
	Householddysf_parent(1)	.182	.087	4.315	1	.038
	Sex(1)	.455	.082	31.073	1	<.001
	Residence type(1)	-.010	.097	.010	1	.920
	Parent education(1)	-.320	.095	11.359	1	<.001
	Immigrant status(1)	-.642	.169	14.368	1	<.001
	Age categories			172.195	5	<.001
	Age categories(1)	-.237	.117	4.119	1	.042
	Age categories(2)	-.798	.124	41.493	1	<.001
	Age categories(3)	-1.079	.133	65.857	1	<.001
	Age categories(4)	-1.579	.156	102.697	1	<.001
	Age categories(5)	-2.083	.220	89.851	1	<.001
	Constant	-2.008	.110	335.221	1	<.001

Variables in the Equation

		Exp(B)	95% C.I.for EXP(B)	
			Lower	Upper
Step 1[a]	Maltreatment_0to3			
	Maltreatment_0to3(1)	2.643	2.176	3.210
	Maltreatment_0to3(2)	3.371	2.611	4.353
	Maltreatment_0to3(3)	8.861	7.016	11.191
	Householddysf_parent(1)	1.199	1.010	1.423
	Sex(1)	1.575	1.343	1.849
	Residence type(1)	.990	.819	1.197
	Parent education(1)	.726	.603	.875
	Immigrant status(1)	.526	.378	.734
	Age categories			
	Age categories(1)	.789	.627	.992
	Age categories(2)	.450	.353	.574
	Age categories(3)	.340	.262	.441
	Age categories(4)	.206	.152	.280
	Age categories(5)	.125	.081	.192
	Constant	.134		

a. Variable(s) entered on step 1: Maltreatment_0to3, Householddysf_parent, Sex, Residence type, Parent education, Immigrant status, Age categories.

Logistic Regression

Notes

Output Created		05-OCT-2024 11:47:34
Comments		
Input	Data	/Users/stevenlc/Library/CloudStorage/OneDrive-Privat/ICloud filer/Doktorander/Rickard/Artikel 3/Revision art 3/Artikel3_revision.sav
	Active Dataset	DataSet7
	Filter	<none>
	Weight	<none>
	Split File	<none>
	N of Rows in Working Data File	10337
Missing Value Handling	Definition of Missing	User-defined missing values are treated as missing
Syntax		LOGISTIC REGRESSION VARIABLES Any_selfharm /METHOD=ENTER Lifetimeabuseindex /CONTRAST (Lifetimeabuseindex)=Indicator(1) /PRINT=CI(95) /CRITERIA=PIN(0.05) POUT(0.10) ITERATE(20) CUT(0.5).
Resources	Processor Time	00:00:00,40
	Elapsed Time	00:00:00,00

Case Processing Summary

Unweighted Cases[a]		N	Percent
Selected Cases	Included in Analysis	8911	86.2
	Missing Cases	1426	13.8
	Total	10337	100.0
Unselected Cases		0	.0
Total		10337	100.0

a. If weight is in effect, see classification table for the total number of cases.

Dependent Variable Encoding

Original Value	Internal Value
1.00	0
2.00	1

Categorical Variables Codings

		Frequency	Parameter coding (1)	(2)	(3)	(4)	(5)
Lifetimeabuseindex	.00	5018	.000	.000	.000	.000	.000
	1.00	1055	1.000	.000	.000	.000	.000
	2.00	452	.000	1.000	.000	.000	.000
	3.00	375	.000	.000	1.000	.000	.000
	4.00	873	.000	.000	.000	1.000	.000
	5.00	479	.000	.000	.000	.000	1.000
	6.00	268	.000	.000	.000	.000	.000
	7.00	391	.000	.000	.000	.000	.000

Categorical Variables Codings

		Parameter coding (6)	(7)
Lifetimeabuseindex	.00	.000	.000
	1.00	.000	.000
	2.00	.000	.000
	3.00	.000	.000
	4.00	.000	.000
	5.00	.000	.000
	6.00	1.000	.000
	7.00	.000	1.000

Block 0: Beginning Block

Classification Table[a,b]

Observed			Predicted: Self harm 1.00	Self harm 2.00	Percentage Correct
Step 0	Self harm	1.00	7866	0	100.0
		2.00	1045	0	.0
	Overall Percentage				88.3

a. Constant is included in the model.

b. The cut value is .500

Variables in the Equation

		B	S.E.	Wald	df	Sig.	Exp(B)
Step 0	Constant	-2.019	.033	3758.507	1	.000	.133

Variables not in the Equation

			Score	df	Sig.
Step 0	Variables	Lifetimeabuseindex	865.640	7	<.001
		Lifetimeabuseindex(1)	.619	1	.431
		Lifetimeabuseindex(2)	3.801	1	.051
		Lifetimeabuseindex(3)	54.513	1	<.001
		Lifetimeabuseindex(4)	7.437	1	.006
		Lifetimeabuseindex(5)	92.354	1	<.001
		Lifetimeabuseindex(6)	58.193	1	<.001
		Lifetimeabuseindex(7)	486.033	1	<.001
	Overall Statistics		865.640	7	<.001

Block 1: Method = Enter

Omnibus Tests of Model Coefficients

		Chi-square	df	Sig.
Step 1	Step	696.887	7	<.001
	Block	696.887	7	<.001
	Model	696.887	7	<.001

Model Summary

Step	-2 Log likelihood	Cox & Snell R Square	Nagelkerke R Square
1	5744.905[a]	.075	.146

a. Estimation terminated at iteration number 6 because parameter estimates changed by less than .001.

Classification Table[a]

Observed			Predicted		
			Self harm		Percentage Correct
			1.00	2.00	
Step 1	Self harm	1.00	7866	0	100.0
		2.00	1045	0	.0
	Overall Percentage				88.3

a. The cut value is .500

Variables in the Equation

		B	S.E.	Wald	df	Sig.
Step 1[a]	Lifetimeabuseindex			677.388	7	<.001
	Lifetimeabuseindex(1)	.772	.117	43.858	1	<.001
	Lifetimeabuseindex(2)	1.097	.147	55.600	1	<.001
	Lifetimeabuseindex(3)	1.696	.137	154.333	1	<.001
	Lifetimeabuseindex(4)	1.093	.115	91.024	1	<.001
	Lifetimeabuseindex(5)	1.789	.122	214.926	1	<.001
	Lifetimeabuseindex(6)	1.843	.152	147.228	1	<.001
	Lifetimeabuseindex(7)	2.735	.119	527.822	1	<.001
	Constant	-2.863	.062	2101.580	1	.000

Variables in the Equation

		Exp(B)	95% C.I.for EXP(B)	
			Lower	Upper
Step 1[a]	Lifetimeabuseindex			
	Lifetimeabuseindex(1)	2.164	1.722	2.719
	Lifetimeabuseindex(2)	2.995	2.245	3.996
	Lifetimeabuseindex(3)	5.451	4.171	7.123
	Lifetimeabuseindex(4)	2.982	2.383	3.732
	Lifetimeabuseindex(5)	5.986	4.712	7.604
	Lifetimeabuseindex(6)	6.313	4.688	8.502
	Lifetimeabuseindex(7)	15.411	12.204	19.461
	Constant	.057		

a. Variable(s) entered on step 1: Lifetimeabuseindex.

Logistic Regression

Notes

Output Created		05-OCT-2024 11:47:34
Comments		
Input	Data	/Users/stevenlc/Library/CloudStorage/OneDrive-Privat/ICloud filer/Doktorander/Rickard/Artikel 3/Revision art 3/Artikel3_revision.sav
	Active Dataset	DataSet7
	Filter	<none>
	Weight	<none>
	Split File	<none>
	N of Rows in Working Data File	10337
Missing Value Handling	Definition of Missing	User-defined missing values are treated as missing
Syntax		LOGISTIC REGRESSION VARIABLES Any_selfharm /METHOD=ENTER Lifetimeabuseindex Householddysf_parent Kon barnboendeny utbildningmammapappa_ny fodelselandmammapappa_ny alderskategorier /CONTRAST (Lifetimeabuseindex)=Indicator(1) /CONTRAST (Householddysf_parent)=Indicator(1) /CONTRAST (Kon)=Indicator(1) /CONTRAST (barnboendeny)=Indicator(1) /CONTRAST (utbildningmammapappa_ny)=Indicator(1) /CONTRAST (fodelselandmammapappa_ny)=Indicator(1) /CONTRAST (alderskategorier)=Indicator(1) /PRINT=CI(95) /CRITERIA=PIN(0.05) POUT(0.10) ITERATE(20) CUT(0.5).
Resources	Processor Time	00:00:00,47
	Elapsed Time	00:00:01,00

Case Processing Summary

Unweighted Cases[a]		N	Percent
Selected Cases	Included in Analysis	7441	72.0
	Missing Cases	2896	28.0
	Total	10337	100.0
Unselected Cases		0	.0
Total		10337	100.0

a. If weight is in effect, see classification table for the total number of cases.

Dependent Variable Encoding

Original Value	Internal Value
1.00	0
2.00	1

Categorical Variables Codings

			Parameter coding		
		Frequency	(1)	(2)	(3)
Lifetimeabuseindex	.00	4305	.000	.000	.000
	1.00	860	1.000	.000	.000
	2.00	352	.000	1.000	.000
	3.00	271	.000	.000	1.000
	4.00	737	.000	.000	.000
	5.00	396	.000	.000	.000
	6.00	221	.000	.000	.000
	7.00	299	.000	.000	.000
Age categories	17-25	887	.000	.000	.000
	26-35	1165	1.000	.000	.000
	36-45	1399	.000	1.000	.000
	46-55	1475	.000	.000	1.000
	56-65	1553	.000	.000	.000
	66-74	962	.000	.000	.000
Parent education	At least one parent high school	3952	.000		
	Both parents below high school	3489	1.000		
Householddysf_parent	.00	5632	.000		
	1.00	1809	1.000		
Sex	Man	3327	.000		
	Kvinna	4114	1.000		
Immigrant status	At least one Nordic parent	6970	.000		
	Both parents born elsewhere	471	1.000		
Residence type	Owned home	5565	.000		
	Rental	1876	1.000		

Categorical Variables Codings

		Parameter coding			
		(4)	(5)	(6)	(7)
Lifetimeabuseindex	.00	.000	.000	.000	.000
	1.00	.000	.000	.000	.000
	2.00	.000	.000	.000	.000
	3.00	.000	.000	.000	.000
	4.00	1.000	.000	.000	.000
	5.00	.000	1.000	.000	.000
	6.00	.000	.000	1.000	.000
	7.00	.000	.000	.000	1.000
Age categories	17-25	.000	.000		
	26-35	.000	.000		
	36-45	.000	.000		
	46-55	.000	.000		
	56-65	1.000	.000		
	66-74	.000	1.000		
Parent education	At least one parent high school				
	Both parents below high school				
Householddysf_parent	.00				
	1.00				
Sex	Man				
	Kvinna				
Immigrant status	At least one Nordic parent				
	Both parents born elsewhere				
Residence type	Owned home				
	Rental				

Block 0: Beginning Block

Classification Table[a,b]

Observed			Predicted		
			Self harm		Percentage Correct
			1.00	2.00	
Step 0	Self harm	1.00	6612	0	100.0
		2.00	829	0	.0
	Overall Percentage				88.9

a. Constant is included in the model.

b. The cut value is .500

Variables in the Equation

		B	S.E.	Wald	df	Sig.	Exp(B)
Step 0	Constant	-2.076	.037	3176.048	1	.000	.125

Variables not in the Equation

			Score	df	Sig.
Step 0	Variables	Lifetimeabuseindex	678.693	7	<.001
		Lifetimeabuseindex(1)	.044	1	.835
		Lifetimeabuseindex(2)	1.008	1	.315
		Lifetimeabuseindex(3)	21.926	1	<.001
		Lifetimeabuseindex(4)	9.425	1	.002
		Lifetimeabuseindex(5)	87.172	1	<.001
		Lifetimeabuseindex(6)	40.657	1	<.001
		Lifetimeabuseindex(7)	400.637	1	<.001
		Householddysf_parent(1)	153.957	1	<.001
		Sex(1)	56.753	1	<.001
		Residence type(1)	.007	1	.932
		Parent education(1)	104.881	1	<.001
		Immigrant status(1)	.458	1	.498
		Age categories	278.770	5	<.001
		Age categories(1)	72.895	1	<.001
		Age categories(2)	.742	1	.389
		Age categories(3)	3.191	1	.074
		Age categories(4)	66.610	1	<.001
		Age categories(5)	71.827	1	<.001
	Overall Statistics		996.631	17	<.001

Block 1: Method = Enter

Omnibus Tests of Model Coefficients

		Chi-square	df	Sig.
Step 1	Step	891.029	17	<.001
	Block	891.029	17	<.001
	Model	891.029	17	<.001

Model Summary

Step	-2 Log likelihood	Cox & Snell R Square	Nagelkerke R Square
1	4309.525[a]	.113	.224

a. Estimation terminated at iteration number 6 because parameter estimates changed by less than .001.

Classification Table[a]

Observed			Predicted: Self harm 1.00	Predicted: Self harm 2.00	Percentage Correct
Step 1	Self harm	1.00	6544	68	99.0
		2.00	731	98	11.8
	Overall Percentage				89.3

a. The cut value is .500

Variables in the Equation

		B	S.E.	Wald	df	Sig.
Step 1[a]	Lifetimeabuseindex			454.938	7	<.001
	Lifetimeabuseindex(1)	.819	.133	38.039	1	<.001
	Lifetimeabuseindex(2)	1.124	.182	38.164	1	<.001
	Lifetimeabuseindex(3)	1.735	.181	91.949	1	<.001
	Lifetimeabuseindex(4)	1.046	.128	66.512	1	<.001
	Lifetimeabuseindex(5)	1.780	.141	159.579	1	<.001
	Lifetimeabuseindex(6)	1.812	.181	99.785	1	<.001
	Lifetimeabuseindex(7)	2.857	.150	364.645	1	<.001
	Householddysf_parent(1)	.160	.090	3.134	1	.077
	Sex(1)	.458	.084	29.572	1	<.001
	Residence type(1)	-.022	.100	.047	1	.828
	Parent education(1)	-.245	.098	6.316	1	.012
	Immigrant status(1)	-.610	.174	12.303	1	<.001
	Age categories			172.484	5	<.001
	Age categories(1)	-.271	.120	5.054	1	.025
	Age categories(2)	-.898	.128	49.234	1	<.001
	Age categories(3)	-1.205	.138	75.815	1	<.001
	Age categories(4)	-1.651	.162	104.142	1	<.001
	Age categories(5)	-2.008	.222	81.544	1	<.001
	Constant	-2.207	.118	349.667	1	<.001

Variables in the Equation

		Exp(B)	95% C.I.for EXP(B) Lower	Upper
Step 1[a]	Lifetimeabuseindex			
	Lifetimeabuseindex(1)	2.267	1.748	2.941
	Lifetimeabuseindex(2)	3.078	2.155	4.398
	Lifetimeabuseindex(3)	5.670	3.977	8.084
	Lifetimeabuseindex(4)	2.847	2.214	3.661
	Lifetimeabuseindex(5)	5.931	4.500	7.818
	Lifetimeabuseindex(6)	6.124	4.291	8.738
	Lifetimeabuseindex(7)	17.407	12.983	23.338
	Householddysf_parent(1)	1.173	.983	1.400
	Sex(1)	1.581	1.341	1.865
	Residence type(1)	.979	.805	1.190
	Parent education(1)	.783	.647	.947
	Immigrant status(1)	.543	.386	.764
	Age categories			
	Age categories(1)	.763	.602	.966
	Age categories(2)	.407	.317	.523
	Age categories(3)	.300	.228	.393
	Age categories(4)	.192	.140	.263
	Age categories(5)	.134	.087	.208
	Constant	.110		

a. Variable(s) entered on step 1: Lifetimeabuseindex, Householddysf_parent, Sex, Residence type, Parent education, Immigrant status, Age categories.

Logistic Regression

Notes

Output Created		05-OCT-2024 11:47:35
Comments		
Input	Data	/Users/stevenlc/Library/CloudStorage/OneDrive-Privat/ICloud filer/Doktorander/Rickard/Artikel 3/Revision art 3/Artikel3_revision.sav
	Active Dataset	DataSet7
	Filter	<none>
	Weight	<none>
	Split File	<none>
	N of Rows in Working Data File	10337
Missing Value Handling	Definition of Missing	User-defined missing values are treated as missing
Syntax		LOGISTIC REGRESSION VARIABLES Symtom_score_måttlig_pos /METHOD=ENTER Maltreatment_0to3 /CONTRAST (Maltreatment_0to3)=Indicator(1) /PRINT=CI(95) /CRITERIA=PIN(0.05) POUT(0.10) ITERATE(20) CUT(0.5).
Resources	Processor Time	00:00:00,38
	Elapsed Time	00:00:00,00

Case Processing Summary

Unweighted Cases[a]		N	Percent
Selected Cases	Included in Analysis	8470	81.9
	Missing Cases	1867	18.1
	Total	10337	100.0
Unselected Cases		0	.0
Total		10337	100.0

a. If weight is in effect, see classification table for the total number of cases.

Dependent Variable Encoding

Original Value	Internal Value
1.00	0
2.00	1

Categorical Variables Codings

		Frequency	Parameter coding (1)	(2)	(3)
Maltreatment_0to3	.00	5610	.000	.000	.000
	1.00	1461	1.000	.000	.000
	2.00	679	.000	1.000	.000
	3.00	720	.000	.000	1.000

Block 0: Beginning Block

Classification Table[a,b]

Observed			Predicted Somatisering 1.00	2.00	Percentage Correct
Step 0	Somatisering	1.00	7973	0	100.0
		2.00	497	0	.0
	Overall Percentage				94.1

a. Constant is included in the model.

b. The cut value is .500

Variables in the Equation

		B	S.E.	Wald	df	Sig.	Exp(B)
Step 0	Constant	-2.775	.046	3603.226	1	.000	.062

Variables not in the Equation

			Score	df	Sig.
Step 0	Variables	Maltreatment_0to3	221.479	3	<.001
		Maltreatment_0to3(1)	11.969	1	<.001
		Maltreatment_0to3(2)	31.870	1	<.001
		Maltreatment_0to3(3)	137.568	1	<.001
	Overall Statistics		221.479	3	<.001

Block 1: Method = Enter

Omnibus Tests of Model Coefficients

		Chi-square	df	Sig.
Step 1	Step	186.731	3	<.001
	Block	186.731	3	<.001
	Model	186.731	3	<.001

Model Summary

Step	-2 Log likelihood	Cox & Snell R Square	Nagelkerke R Square
1	3596.201[a]	.022	.061

a. Estimation terminated at iteration number 6 because parameter estimates changed by less than .001.

Classification Table[a]

Observed			Predicted: Somatisering 1.00	Predicted: Somatisering 2.00	Predicted: Percentage Correct
Step 1	Somatisering	1.00	7973	0	100.0
		2.00	497	0	.0
	Overall Percentage				94.1

a. The cut value is .500

Variables in the Equation

		B	S.E.	Wald	df	Sig.
Step 1[a]	Maltreatment_0to3			194.171	3	<.001
	Maltreatment_0to3(1)	.844	.122	48.202	1	<.001
	Maltreatment_0to3(2)	1.197	.144	69.514	1	<.001
	Maltreatment_0to3(3)	1.632	.126	169.066	1	<.001
	Constant	-3.313	.073	2086.783	1	.000

Variables in the Equation

		Exp(B)	95% C.I.for EXP(B) Lower	95% C.I.for EXP(B) Upper
Step 1[a]	Maltreatment_0to3			
	Maltreatment_0to3(1)	2.325	1.832	2.951
	Maltreatment_0to3(2)	3.310	2.498	4.386
	Maltreatment_0to3(3)	5.115	4.000	6.542
	Constant	.036		

a. Variable(s) entered on step 1: Maltreatment_0to3.

Logistic Regression

Notes

Output Created		05-OCT-2024 11:47:35
Comments		
Input	Data	/Users/stevenlc/Library/CloudStorage/OneDrive-Privat/ICloud filer/Doktorander/Rickard/Artikel 3/Revision art 3/Artikel3_revision.sav
	Active Dataset	DataSet7
	Filter	<none>
	Weight	<none>
	Split File	<none>
	N of Rows in Working Data File	10337
Missing Value Handling	Definition of Missing	User-defined missing values are treated as missing
Syntax		LOGISTIC REGRESSION VARIABLES Symtom_score_måttlig_pos /METHOD=ENTER Maltreatment_0to3 Householddysf_parent Kon barnboendeny utbildningmammapappa_ny fodelselandmammapappa_ny alderskategorier /CONTRAST (Maltreatment_0to3)=Indicator(1) /CONTRAST (Householddysf_parent)=Indicator(1) /CONTRAST (Kon)=Indicator(1) /CONTRAST (barnboendeny)=Indicator(1) /CONTRAST (utbildningmammapappa_ny)=Indicator(1) /CONTRAST (fodelselandmammapappa_ny)=Indicator(1) /CONTRAST (alderskategorier)=Indicator(1) /PRINT=CI(95) /CRITERIA=PIN(0.05) POUT(0.10) ITERATE(20) CUT(0.5).
Resources	Processor Time	00:00:00,44
	Elapsed Time	00:00:01,00

Case Processing Summary

Unweighted Cases[a]		N	Percent
Selected Cases	Included in Analysis	7071	68.4
	Missing Cases	3266	31.6
	Total	10337	100.0
Unselected Cases		0	.0
Total		10337	100.0

a. If weight is in effect, see classification table for the total number of cases.

Dependent Variable Encoding

Original Value	Internal Value
1.00	0
2.00	1

Categorical Variables Codings

			Parameter coding		
		Frequency	(1)	(2)	(3)
Age categories	17-25	860	.000	.000	.000
	26-35	1137	1.000	.000	.000
	36-45	1353	.000	1.000	.000
	46-55	1419	.000	.000	1.000
	56-65	1430	.000	.000	.000
	66-74	872	.000	.000	.000
Maltreatment_0to3	.00	4803	.000	.000	.000
	1.00	1194	1.000	.000	.000
	2.00	545	.000	1.000	.000
	3.00	529	.000	.000	1.000
Parent education	At least one parent high school	3828	.000		
	Both parents below high school	3243	1.000		
Householddysf_parent	.00	5333	.000		
	1.00	1738	1.000		
Sex	Man	3180	.000		
	Kvinna	3891	1.000		
Immigrant status	At least one Nordic parent	6633	.000		
	Both parents born elsewhere	438	1.000		
Residence type	Owned home	5306	.000		
	Rental	1765	1.000		

Categorical Variables Codings

		Parameter coding (4)	Parameter coding (5)
Age categories	17-25	.000	.000
	26-35	.000	.000
	36-45	.000	.000
	46-55	.000	.000
	56-65	1.000	.000
	66-74	.000	1.000
Maltreatment_0to3	.00		
	1.00		
	2.00		
	3.00		
Parent education	At least one parent high school		
	Both parents below high school		
Householddysf_parent	.00		
	1.00		
Sex	Man		
	Kvinna		
Immigrant status	At least one Nordic parent		
	Both parents born elsewhere		
Residence type	Owned home		
	Rental		

Block 0: Beginning Block

Classification Table[a,b]

Observed			Predicted: Somatisering 1.00	Predicted: Somatisering 2.00	Percentage Correct
Step 0	Somatisering	1.00	6681	0	100.0
		2.00	390	0	.0
	Overall Percentage				94.5

a. Constant is included in the model.

b. The cut value is .500

Variables in the Equation

		B	S.E.	Wald	df	Sig.	Exp(B)
Step 0	Constant	-2.841	.052	2973.924	1	.000	.058

Variables not in the Equation

			Score	df	Sig.
Step 0	Variables	Maltreatment_0to3	177.101	3	<.001
		Maltreatment_0to3(1)	9.483	1	.002
		Maltreatment_0to3(2)	31.953	1	<.001
		Maltreatment_0to3(3)	105.297	1	<.001
		Householddysf_parent(1)	16.078	1	<.001
		Sex(1)	49.805	1	<.001
		Residence type(1)	9.534	1	.002
		Parent education(1)	6.904	1	.009
		Immigrant status(1)	16.581	1	<.001
		Age categories	13.351	5	.020
		Age categories(1)	4.964	1	.026
		Age categories(2)	.555	1	.456
		Age categories(3)	5.321	1	.021
		Age categories(4)	2.474	1	.116
		Age categories(5)	.091	1	.763
	Overall Statistics		241.266	13	<.001

Block 1: Method = Enter

Omnibus Tests of Model Coefficients

		Chi-square	df	Sig.
Step 1	Step	213.224	13	<.001
	Block	213.224	13	<.001
	Model	213.224	13	<.001

Model Summary

Step	-2 Log likelihood	Cox & Snell R Square	Nagelkerke R Square
1	2804.995[a]	.030	.085

a. Estimation terminated at iteration number 6 because parameter estimates changed by less than .001.

Classification Table[a]

			Predicted		
			Somatisering		Percentage Correct
	Observed		1.00	2.00	
Step 1	Somatisering	1.00	6681	0	100.0
		2.00	390	0	.0
	Overall Percentage				94.5

a. The cut value is .500

Variables in the Equation

		B	S.E.	Wald	df	Sig.
Step 1[a]	Maltreatment_0to3			108.414	3	<.001
	Maltreatment_0to3(1)	.770	.139	30.775	1	<.001
	Maltreatment_0to3(2)	1.119	.164	46.621	1	<.001
	Maltreatment_0to3(3)	1.496	.158	90.070	1	<.001
	Householddysf_parent(1)	.115	.127	.816	1	.366
	Sex(1)	.767	.118	42.475	1	<.001
	Residence type(1)	-.002	.121	.000	1	.987
	Parent education(1)	.159	.120	1.742	1	.187
	Immigrant status(1)	.594	.179	10.976	1	<.001
	Age categories			7.875	5	.163
	Age categories(1)	-.135	.229	.346	1	.557
	Age categories(2)	.107	.215	.250	1	.617
	Age categories(3)	.327	.211	2.392	1	.122
	Age categories(4)	.321	.220	2.128	1	.145
	Age categories(5)	.317	.246	1.662	1	.197
	Constant	-4.139	.205	407.108	1	<.001

Variables in the Equation

		Exp(B)	95% C.I.for EXP(B)	
			Lower	Upper
Step 1[a]	Maltreatment_0to3			
	Maltreatment_0to3(1)	2.159	1.645	2.834
	Maltreatment_0to3(2)	3.063	2.221	4.224
	Maltreatment_0to3(3)	4.462	3.276	6.077
	Householddysf_parent(1)	1.121	.875	1.438
	Sex(1)	2.153	1.710	2.712
	Residence type(1)	.998	.787	1.266
	Parent education(1)	1.172	.926	1.485
	Immigrant status(1)	1.811	1.274	2.573
	Age categories			
	Age categories(1)	.874	.557	1.370
	Age categories(2)	1.113	.731	1.697
	Age categories(3)	1.386	.916	2.097
	Age categories(4)	1.379	.896	2.122
	Age categories(5)	1.373	.848	2.222
	Constant	.016		

a. Variable(s) entered on step 1: Maltreatment_0to3, Householddysf_parent, Sex, Residence type, Parent education, Immigrant status, Age categories.

Logistic Regression

Notes

Output Created		05-OCT-2024 11:47:36
Comments		
Input	Data	/Users/stevenlc/Library/CloudStorage/OneDrive-Privat/ICloud filer/Doktorander/Rickard/Artikel 3/Revision art 3/Artikel3_revision.sav
	Active Dataset	DataSet7
	Filter	<none>
	Weight	<none>
	Split File	<none>
	N of Rows in Working Data File	10337
Missing Value Handling	Definition of Missing	User-defined missing values are treated as missing
Syntax		LOGISTIC REGRESSION VARIABLES Symtom_score_måttlig_pos /METHOD=ENTER Lifetimeabuseindex /CONTRAST (Lifetimeabuseindex)=Indicator(1) /PRINT=CI(95) /CRITERIA=PIN(0.05) POUT(0.10) ITERATE(20) CUT(0.5).
Resources	Processor Time	00:00:00,40
	Elapsed Time	00:00:00,00

Case Processing Summary

Unweighted Cases[a]		N	Percent
Selected Cases	Included in Analysis	8279	80.1
	Missing Cases	2058	19.9
	Total	10337	100.0
Unselected Cases		0	.0
Total		10337	100.0

a. If weight is in effect, see classification table for the total number of cases.

Dependent Variable Encoding

Original Value	Internal Value
1.00	0
2.00	1

Categorical Variables Codings

		Frequency	Parameter coding (1)	(2)	(3)	(4)	(5)
Lifetimeabuseindex	.00	4676	.000	.000	.000	.000	.000
	1.00	983	1.000	.000	.000	.000	.000
	2.00	413	.000	1.000	.000	.000	.000
	3.00	352	.000	.000	1.000	.000	.000
	4.00	818	.000	.000	.000	1.000	.000
	5.00	440	.000	.000	.000	.000	1.000
	6.00	248	.000	.000	.000	.000	.000
	7.00	349	.000	.000	.000	.000	.000

Categorical Variables Codings

		Parameter coding (6)	(7)
Lifetimeabuseindex	.00	.000	.000
	1.00	.000	.000
	2.00	.000	.000
	3.00	.000	.000
	4.00	.000	.000
	5.00	.000	.000
	6.00	1.000	.000
	7.00	.000	1.000

Block 0: Beginning Block

Classification Table[a,b]

Observed			Predicted Somatisering 1.00	Somatisering 2.00	Percentage Correct
Step 0	Somatisering	1.00	7792	0	100.0
		2.00	487	0	.0
	Overall Percentage				94.1

a. Constant is included in the model.

b. The cut value is .500

Variables in the Equation

		B	S.E.	Wald	df	Sig.	Exp(B)
Step 0	Constant	-2.773	.047	3523.473	1	.000	.063

Variables not in the Equation

			Score	df	Sig.
Step 0	Variables	Lifetimeabuseindex	295.711	7	<.001
		Lifetimeabuseindex(1)	.559	1	.455
		Lifetimeabuseindex(2)	1.499	1	.221
		Lifetimeabuseindex(3)	16.029	1	<.001
		Lifetimeabuseindex(4)	.584	1	.445
		Lifetimeabuseindex(5)	23.170	1	<.001
		Lifetimeabuseindex(6)	56.417	1	<.001
		Lifetimeabuseindex(7)	137.637	1	<.001
	Overall Statistics		295.711	7	<.001

Block 1: Method = Enter

Omnibus Tests of Model Coefficients

		Chi-square	df	Sig.
Step 1	Step	236.943	7	<.001
	Block	236.943	7	<.001
	Model	236.943	7	<.001

Model Summary

Step	-2 Log likelihood	Cox & Snell R Square	Nagelkerke R Square
1	3467.381[a]	.028	.078

a. Estimation terminated at iteration number 6 because parameter estimates changed by less than .001.

Classification Table[a]

Observed			Predicted: Somatisering 1.00	Predicted: Somatisering 2.00	Percentage Correct
Step 1	Somatisering	1.00	7792	0	100.0
		2.00	487	0	.0
	Overall Percentage				94.1

a. The cut value is .500

Variables in the Equation

		B	S.E.	Wald	df	Sig.
Step 1[a]	Lifetimeabuseindex			245.465	7	<.001
	Lifetimeabuseindex(1)	.790	.156	25.685	1	<.001
	Lifetimeabuseindex(2)	.924	.208	19.737	1	<.001
	Lifetimeabuseindex(3)	1.359	.192	50.170	1	<.001
	Lifetimeabuseindex(4)	.801	.166	23.355	1	<.001
	Lifetimeabuseindex(5)	1.394	.174	64.173	1	<.001
	Lifetimeabuseindex(6)	1.881	.190	98.306	1	<.001
	Lifetimeabuseindex(7)	2.106	.158	177.429	1	<.001
	Constant	-3.471	.086	1647.351	1	.000

Variables in the Equation

		Exp(B)	95% C.I.for EXP(B)	
			Lower	Upper
Step 1[a]	Lifetimeabuseindex			
	Lifetimeabuseindex(1)	2.202	1.623	2.989
	Lifetimeabuseindex(2)	2.519	1.676	3.787
	Lifetimeabuseindex(3)	3.892	2.672	5.669
	Lifetimeabuseindex(4)	2.228	1.610	3.084
	Lifetimeabuseindex(5)	4.031	2.866	5.669
	Lifetimeabuseindex(6)	6.558	4.522	9.510
	Lifetimeabuseindex(7)	8.214	6.026	11.198
	Constant	.031		

a. Variable(s) entered on step 1: Lifetimeabuseindex.

Logistic Regression

Notes

Output Created		05-OCT-2024 11:47:36
Comments		
Input	Data	/Users/stevenlc/Library/CloudStorage/OneDrive-Privat/ICloud filer/Doktorander/Rickard/Artikel 3/Revision art 3/Artikel3_revision.sav
	Active Dataset	DataSet7
	Filter	<none>
	Weight	<none>
	Split File	<none>
	N of Rows in Working Data File	10337
Missing Value Handling	Definition of Missing	User-defined missing values are treated as missing
Syntax		LOGISTIC REGRESSION VARIABLES Symtom_score_måttlig_pos<br>/METHOD=ENTER Lifetimeabuseindex Householddysf_parent Kon barnboendeny utbildningmammapappa_ny fodelselandmammapappa_ny alderskategorier<br>/CONTRAST (Lifetimeabuseindex)=Indicator(1)<br>/CONTRAST (Householddysf_parent)=Indicator(1)<br>/CONTRAST (Kon)=Indicator(1)<br>/CONTRAST (barnboendeny)=Indicator(1)<br>/CONTRAST (utbildningmammapappa_ny)=Indicator(1)<br>/CONTRAST (fodelselandmammapappa_ny)=Indicator(1)<br>/CONTRAST (alderskategorier)=Indicator(1)<br>/PRINT=CI(95)<br>/CRITERIA=PIN(0.05) POUT(0.10) ITERATE(20) CUT(0.5).
Resources	Processor Time	00:00:00,46
	Elapsed Time	00:00:01,00

Case Processing Summary

Unweighted Cases[a]		N	Percent
Selected Cases	Included in Analysis	6926	67.0
	Missing Cases	3411	33.0
	Total	10337	100.0
Unselected Cases		0	.0
Total		10337	100.0

a. If weight is in effect, see classification table for the total number of cases.

Dependent Variable Encoding

Original Value	Internal Value
1.00	0
2.00	1

Categorical Variables Codings

		Frequency	Parameter coding		
			(1)	(2)	(3)
Lifetimeabuseindex	.00	4019	.000	.000	.000
	1.00	801	1.000	.000	.000
	2.00	325	.000	1.000	.000
	3.00	252	.000	.000	1.000
	4.00	692	.000	.000	.000
	5.00	367	.000	.000	.000
	6.00	206	.000	.000	.000
	7.00	264	.000	.000	.000
Age categories	17-25	840	.000	.000	.000
	26-35	1117	1.000	.000	.000
	36-45	1326	.000	1.000	.000
	46-55	1391	.000	.000	1.000
	56-65	1399	.000	.000	.000
	66-74	853	.000	.000	.000
Parent education	At least one parent high school	3734	.000		
	Both parents below high school	3192	1.000		
Householddysf_parent	.00	5231	.000		
	1.00	1695	1.000		
Sex	Man	3110	.000		
	Kvinna	3816	1.000		
Immigrant status	At least one Nordic parent	6497	.000		
	Both parents born elsewhere	429	1.000		
Residence type	Owned home	5192	.000		
	Rental	1734	1.000		

Categorical Variables Codings

		Parameter coding			
		(4)	(5)	(6)	(7)
Lifetimeabuseindex	.00	.000	.000	.000	.000
	1.00	.000	.000	.000	.000
	2.00	.000	.000	.000	.000
	3.00	.000	.000	.000	.000
	4.00	1.000	.000	.000	.000
	5.00	.000	1.000	.000	.000
	6.00	.000	.000	1.000	.000
	7.00	.000	.000	.000	1.000
Age categories	17-25	.000	.000		
	26-35	.000	.000		
	36-45	.000	.000		
	46-55	.000	.000		
	56-65	1.000	.000		
	66-74	.000	1.000		
Parent education	At least one parent high school				
	Both parents below high school				
Householddysf_parent	.00				
	1.00				
Sex	Man				
	Kvinna				
Immigrant status	At least one Nordic parent				
	Both parents born elsewhere				
Residence type	Owned home				
	Rental				

Block 0: Beginning Block

Classification Table[a,b]

			Predicted		
			Somatisering		Percentage Correct
	Observed		1.00	2.00	
Step 0	Somatisering	1.00	6541	0	100.0
		2.00	385	0	.0
	Overall Percentage				94.4

a. Constant is included in the model.

b. The cut value is .500

Variables in the Equation

		B	S.E.	Wald	df	Sig.	Exp(B)
Step 0	Constant	-2.833	.052	2917.384	1	.000	.059

Variables not in the Equation

			Score	df	Sig.
Step 0	Variables	Lifetimeabuseindex	258.392	7	<.001
		Lifetimeabuseindex(1)	.006	1	.938
		Lifetimeabuseindex(2)	.952	1	.329
		Lifetimeabuseindex(3)	15.357	1	<.001
		Lifetimeabuseindex(4)	2.227	1	.136
		Lifetimeabuseindex(5)	23.257	1	<.001
		Lifetimeabuseindex(6)	62.209	1	<.001
		Lifetimeabuseindex(7)	104.503	1	<.001
		Householddysf_parent(1)	15.027	1	<.001
		Sex(1)	49.720	1	<.001
		Residence type(1)	8.169	1	.004
		Parent education(1)	6.146	1	.013
		Immigrant status(1)	17.364	1	<.001
		Age categories	12.159	5	.033
		Age categories(1)	4.631	1	.031
		Age categories(2)	.579	1	.447
		Age categories(3)	4.766	1	.029
		Age categories(4)	2.153	1	.142
		Age categories(5)	.170	1	.680
	Overall Statistics		321.633	17	<.001

Block 1: Method = Enter

Omnibus Tests of Model Coefficients

		Chi-square	df	Sig.
Step 1	Step	268.988	17	<.001
	Block	268.988	17	<.001
	Model	268.988	17	<.001

Model Summary

Step	-2 Log likelihood	Cox & Snell R Square	Nagelkerke R Square
1	2704.345[a]	.038	.109

a. Estimation terminated at iteration number 6 because parameter estimates changed by less than .001.

Classification Table[a]

Observed			Predicted: Somatisering 1.00	Predicted: Somatisering 2.00	Percentage Correct
Step 1	Somatisering	1.00	6541	0	100.0
		2.00	385	0	.0
	Overall Percentage				94.4

a. The cut value is .500

Variables in the Equation

		B	S.E.	Wald	df	Sig.
Step 1[a]	Lifetimeabuseindex			166.145	7	<.001
	Lifetimeabuseindex(1)	.683	.182	14.025	1	<.001
	Lifetimeabuseindex(2)	.837	.244	11.768	1	<.001
	Lifetimeabuseindex(3)	1.336	.231	33.501	1	<.001
	Lifetimeabuseindex(4)	.963	.181	28.427	1	<.001
	Lifetimeabuseindex(5)	1.453	.196	55.057	1	<.001
	Lifetimeabuseindex(6)	1.876	.211	79.030	1	<.001
	Lifetimeabuseindex(7)	2.060	.195	111.090	1	<.001
	Householddysf_parent(1)	.064	.129	.244	1	.621
	Sex(1)	.732	.119	37.574	1	<.001
	Residence type(1)	-.036	.124	.086	1	.770
	Parent education(1)	.198	.122	2.618	1	.106
	Immigrant status(1)	.619	.182	11.577	1	<.001
	Age categories			8.912	5	.113
	Age categories(1)	-.179	.231	.596	1	.440
	Age categories(2)	.031	.217	.020	1	.888
	Age categories(3)	.239	.214	1.253	1	.263
	Age categories(4)	.324	.223	2.120	1	.145
	Age categories(5)	.386	.248	2.413	1	.120
	Constant	-4.288	.213	406.354	1	<.001

Variables in the Equation

		Exp(B)	95% C.I.for EXP(B) Lower	Upper
Step 1[a]	Lifetimeabuseindex			
	Lifetimeabuseindex(1)	1.979	1.385	2.829
	Lifetimeabuseindex(2)	2.310	1.432	3.726
	Lifetimeabuseindex(3)	3.803	2.419	5.978
	Lifetimeabuseindex(4)	2.619	1.838	3.732
	Lifetimeabuseindex(5)	4.274	2.912	6.273
	Lifetimeabuseindex(6)	6.527	4.316	9.870
	Lifetimeabuseindex(7)	7.844	5.348	11.504
	Householddysf_parent(1)	1.066	.828	1.373
	Sex(1)	2.079	1.645	2.627
	Residence type(1)	.964	.757	1.229
	Parent education(1)	1.219	.959	1.550
	Immigrant status(1)	1.857	1.300	2.653
	Age categories			
	Age categories(1)	.836	.532	1.316
	Age categories(2)	1.031	.673	1.578
	Age categories(3)	1.270	.836	1.931
	Age categories(4)	1.383	.894	2.139
	Age categories(5)	1.471	.904	2.393
	Constant	.014		

a. Variable(s) entered on step 1: Lifetimeabuseindex, Householddysf_parent, Sex, Residence type, Parent education, Immigrant status, Age categories.

Logistic Regression

Notes

Output Created		05-OCT-2024 11:47:37
Comments		
Input	Data	/Users/stevenlc/Library/CloudStorage/OneDrive-Privat/ICloud filer/Doktorander/Rickard/Artikel 3/Revision art 3/Artikel3_revision.sav
	Active Dataset	DataSet7
	Filter	<none>
	Weight	<none>
	Split File	<none>
	N of Rows in Working Data File	10337
Missing Value Handling	Definition of Missing	User-defined missing values are treated as missing
Syntax		LOGISTIC REGRESSION VARIABLES IBS /METHOD=ENTER Maltreatment_0to3 /CONTRAST (Maltreatment_0to3)=Indicator(1) /PRINT=CI(95) /CRITERIA=PIN(0.05) POUT(0.10) ITERATE(20) CUT(0.5).
Resources	Processor Time	00:00:00,38
	Elapsed Time	00:00:00,00

Case Processing Summary

Unweighted Cases[a]		N	Percent
Selected Cases	Included in Analysis	9169	88.7
	Missing Cases	1168	11.3
	Total	10337	100.0
Unselected Cases		0	.0
Total		10337	100.0

a. If weight is in effect, see classification table for the total number of cases.

Dependent Variable Encoding

Original Value	Internal Value
1.00	0
2.00	1

Categorical Variables Codings

		Frequency	Parameter coding (1)	(2)	(3)
Maltreatment_0to3	.00	6043	.000	.000	.000
	1.00	1589	1.000	.000	.000
	2.00	744	.000	1.000	.000
	3.00	793	.000	.000	1.000

Block 0: Beginning Block

Classification Table[a,b]

Observed			Predicted IBS 1.00	2.00	Percentage Correct
Step 0	IBS	1.00	8755	0	100.0
		2.00	414	0	.0
	Overall Percentage				95.5

a. Constant is included in the model.

b. The cut value is .500

Variables in the Equation

		B	S.E.	Wald	df	Sig.	Exp(B)
Step 0	Constant	-3.052	.050	3680.996	1	.000	.047

Variables not in the Equation

			Score	df	Sig.
Step 0	Variables	Maltreatment_0to3	44.369	3	<.001
		Maltreatment_0to3(1)	.028	1	.868
		Maltreatment_0to3(2)	6.098	1	.014
		Maltreatment_0to3(3)	33.186	1	<.001
	Overall Statistics		44.369	3	<.001

Block 1: Method = Enter

Omnibus Tests of Model Coefficients

		Chi-square	df	Sig.
Step 1	Step	38.192	3	<.001
	Block	38.192	3	<.001
	Model	38.192	3	<.001

Model Summary

Step	-2 Log likelihood	Cox & Snell R Square	Nagelkerke R Square
1	3335.737[a]	.004	.014

a. Estimation terminated at iteration number 6 because parameter estimates changed by less than .001.

Classification Table[a]

Observed			Predicted IBS 1.00	Predicted IBS 2.00	Percentage Correct
Step 1	IBS	1.00	8755	0	100.0
		2.00	414	0	.0
	Overall Percentage				95.5

a. The cut value is .500

Variables in the Equation

		B	S.E.	Wald	df	Sig.
Step 1[a]	Maltreatment_0to3			42.415	3	<.001
	Maltreatment_0to3(1)	.215	.138	2.430	1	.119
	Maltreatment_0to3(2)	.551	.165	11.132	1	<.001
	Maltreatment_0to3(3)	.881	.144	37.558	1	<.001
	Constant	-3.248	.068	2295.029	1	.000

Variables in the Equation

		Exp(B)	95% C.I.for EXP(B) Lower	95% C.I.for EXP(B) Upper
Step 1[a]	Maltreatment_0to3			
	Maltreatment_0to3(1)	1.239	.946	1.623
	Maltreatment_0to3(2)	1.736	1.255	2.399
	Maltreatment_0to3(3)	2.414	1.821	3.200
	Constant	.039		

a. Variable(s) entered on step 1: Maltreatment_0to3.

Logistic Regression

Notes

Output Created		05-OCT-2024 11:47:37
Comments		
Input	Data	/Users/stevenlc/Library/CloudStorage/OneDrive-Privat/ICloud filer/Doktorander/Rickard/Artikel 3/Revision art 3/Artikel3_revision.sav
	Active Dataset	DataSet7
	Filter	<none>
	Weight	<none>
	Split File	<none>
	N of Rows in Working Data File	10337
Missing Value Handling	Definition of Missing	User-defined missing values are treated as missing
Syntax		LOGISTIC REGRESSION VARIABLES IBS<br>/METHOD=ENTER Maltreatment_0to3 Householddysf_parent Kon barnboendeny utbildningmammapappa_ny fodelselandmammapappa_ny alderskategorier<br>/CONTRAST (Maltreatment_0to3)=Indicator(1)<br>/CONTRAST (Householddysf_parent)=Indicator(1)<br>/CONTRAST (Kon)=Indicator(1)<br>/CONTRAST (barnboendeny)=Indicator(1)<br>/CONTRAST (utbildningmammapappa_ny)=Indicator(1)<br>/CONTRAST (fodelselandmammapappa_ny)=Indicator(1)<br>/CONTRAST (alderskategorier)=Indicator(1)<br>/PRINT=CI(95)<br>/CRITERIA=PIN(0.05) POUT(0.10) ITERATE(20) CUT(0.5).
Resources	Processor Time	00:00:00,44
	Elapsed Time	00:00:00,00

Case Processing Summary

Unweighted Cases[a]		N	Percent
Selected Cases	Included in Analysis	7629	73.8
	Missing Cases	2708	26.2
	Total	10337	100.0
Unselected Cases		0	.0
Total		10337	100.0

a. If weight is in effect, see classification table for the total number of cases.

Dependent Variable Encoding

Original Value	Internal Value
1.00	0
2.00	1

Categorical Variables Codings

		Frequency	Parameter coding (1)	(2)	(3)
Age categories	17-25	911	.000	.000	.000
	26-35	1194	1.000	.000	.000
	36-45	1430	.000	1.000	.000
	46-55	1512	.000	.000	1.000
	56-65	1592	.000	.000	.000
	66-74	990	.000	.000	.000
Maltreatment_0to3	.00	5160	.000	.000	.000
	1.00	1292	1.000	.000	.000
	2.00	590	.000	1.000	.000
	3.00	587	.000	.000	1.000
Parent education	At least one parent high school	4067	.000		
	Both parents below high school	3562	1.000		
Householddysf_parent	.00	5768	.000		
	1.00	1861	1.000		
Sex	Man	3417	.000		
	Kvinna	4212	1.000		
Immigrant status	At least one Nordic parent	7143	.000		
	Both parents born elsewhere	486	1.000		
Residence type	Owned home	5713	.000		
	Rental	1916	1.000		

Categorical Variables Codings

		Parameter coding (4)	Parameter coding (5)
Age categories	17-25	.000	.000
	26-35	.000	.000
	36-45	.000	.000
	46-55	.000	.000
	56-65	1.000	.000
	66-74	.000	1.000
Maltreatment_0to3	.00		
	1.00		
	2.00		
	3.00		
Parent education	At least one parent high school		
	Both parents below high school		
Householddysf_parent	.00		
	1.00		
Sex	Man		
	Kvinna		
Immigrant status	At least one Nordic parent		
	Both parents born elsewhere		
Residence type	Owned home		
	Rental		

Block 0: Beginning Block

Classification Table[a,b]

Observed			Predicted IBS 1.00	Predicted IBS 2.00	Percentage Correct
Step 0	IBS	1.00	7280	0	100.0
		2.00	349	0	.0
	Overall Percentage				95.4

a. Constant is included in the model.

b. The cut value is .500

Variables in the Equation

		B	S.E.	Wald	df	Sig.	Exp(B)
Step 0	Constant	-3.038	.055	3073.347	1	.000	.048

Variables not in the Equation

			Score	df	Sig.
Step 0	Variables	Maltreatment_0to3	43.572	3	<.001
		Maltreatment_0to3(1)	.206	1	.650
		Maltreatment_0to3(2)	10.786	1	.001
		Maltreatment_0to3(3)	28.903	1	<.001
		Householddysf_parent(1)	4.098	1	.043
		Sex(1)	53.401	1	<.001
		Residence type(1)	12.832	1	<.001
		Parent education(1)	.600	1	.439
		Immigrant status(1)	1.956	1	.162
		Age categories	18.197	5	.003
		Age categories(1)	4.863	1	.027
		Age categories(2)	.007	1	.935
		Age categories(3)	1.159	1	.282
		Age categories(4)	2.694	1	.101
		Age categories(5)	3.647	1	.056
	Overall Statistics		122.365	13	<.001

Block 1: Method = Enter

Omnibus Tests of Model Coefficients

		Chi-square	df	Sig.
Step 1	Step	119.887	13	<.001
	Block	119.887	13	<.001
	Model	119.887	13	<.001

Model Summary

Step	-2 Log likelihood	Cox & Snell R Square	Nagelkerke R Square
1	2714.977[a]	.016	.050

a. Estimation terminated at iteration number 6 because parameter estimates changed by less than .001.

Classification Table[a]

			Predicted		
			IBS		Percentage Correct
Observed			1.00	2.00	
Step 1	IBS	1.00	7280	0	100.0
		2.00	349	0	.0
	Overall Percentage				95.4

a. The cut value is .500

Variables in the Equation

		B	S.E.	Wald	df	Sig.
Step 1[a]	Maltreatment_0to3			26.208	3	<.001
	Maltreatment_0to3(1)	.070	.157	.199	1	.655
	Maltreatment_0to3(2)	.590	.179	10.862	1	<.001
	Maltreatment_0to3(3)	.778	.175	19.824	1	<.001
	Householddysf_parent(1)	.078	.136	.331	1	.565
	Sex(1)	.880	.126	49.139	1	<.001
	Residence type(1)	.243	.125	3.796	1	.051
	Parent education(1)	-.224	.125	3.216	1	.073
	Immigrant status(1)	-.441	.266	2.742	1	.098
	Age categories			18.986	5	.002
	Age categories(1)	.307	.264	1.350	1	.245
	Age categories(2)	.651	.246	6.991	1	.008
	Age categories(3)	.735	.247	8.831	1	.003
	Age categories(4)	.856	.253	11.464	1	<.001
	Age categories(5)	1.022	.269	14.407	1	<.001
	Constant	-4.382	.238	338.156	1	<.001

Variables in the Equation

		Exp(B)	95% C.I.for EXP(B)	
			Lower	Upper
Step 1[a]	Maltreatment_0to3			
	Maltreatment_0to3(1)	1.073	.788	1.459
	Maltreatment_0to3(2)	1.804	1.270	2.563
	Maltreatment_0to3(3)	2.177	1.546	3.066
	Householddysf_parent(1)	1.082	.828	1.413
	Sex(1)	2.411	1.885	3.084
	Residence type(1)	1.275	.999	1.627
	Parent education(1)	.799	.625	1.021
	Immigrant status(1)	.643	.382	1.084
	Age categories			
	Age categories(1)	1.359	.810	2.280
	Age categories(2)	1.917	1.183	3.105
	Age categories(3)	2.086	1.284	3.388
	Age categories(4)	2.355	1.434	3.865
	Age categories(5)	2.778	1.639	4.708
	Constant	.013		

a. Variable(s) entered on step 1: Maltreatment_0to3, Householddysf_parent, Sex, Residence type, Parent education, Immigrant status, Age categories.

Logistic Regression

Notes

Output Created		05-OCT-2024 11:47:37
Comments		
Input	Data	/Users/stevenlc/Library/CloudStorage/OneDrive-Privat/ICloud filer/Doktorander/Rickard/Artikel 3/Revision art 3/Artikel3_revision.sav
	Active Dataset	DataSet7
	Filter	<none>
	Weight	<none>
	Split File	<none>
	N of Rows in Working Data File	10337
Missing Value Handling	Definition of Missing	User-defined missing values are treated as missing
Syntax		LOGISTIC REGRESSION VARIABLES IBS /METHOD=ENTER Lifetimeabuseindex /CONTRAST (Lifetimeabuseindex)=Indicator(1) /PRINT=CI(95) /CRITERIA=PIN(0.05) POUT(0.10) ITERATE(20) CUT(0.5).
Resources	Processor Time	00:00:00,40
	Elapsed Time	00:00:01,00

Case Processing Summary

Unweighted Cases[a]		N	Percent
Selected Cases	Included in Analysis	8951	86.6
	Missing Cases	1386	13.4
	Total	10337	100.0
Unselected Cases		0	.0
Total		10337	100.0

a. If weight is in effect, see classification table for the total number of cases.

Dependent Variable Encoding

Original Value	Internal Value
1.00	0
2.00	1

Categorical Variables Codings

		Frequency	Parameter coding (1)	(2)	(3)	(4)	(5)
Lifetimeabuseindex	.00	5038	.000	.000	.000	.000	.000
	1.00	1062	1.000	.000	.000	.000	.000
	2.00	454	.000	1.000	.000	.000	.000
	3.00	377	.000	.000	1.000	.000	.000
	4.00	875	.000	.000	.000	1.000	.000
	5.00	483	.000	.000	.000	.000	1.000
	6.00	269	.000	.000	.000	.000	.000
	7.00	393	.000	.000	.000	.000	.000

Categorical Variables Codings

		Parameter coding (6)	(7)
Lifetimeabuseindex	.00	.000	.000
	1.00	.000	.000
	2.00	.000	.000
	3.00	.000	.000
	4.00	.000	.000
	5.00	.000	.000
	6.00	1.000	.000
	7.00	.000	1.000

Block 0: Beginning Block

Classification Table[a,b]

Observed			Predicted IBS 1.00	2.00	Percentage Correct
Step 0	IBS	1.00	8549	0	100.0
		2.00	402	0	.0
	Overall Percentage				95.5

a. Constant is included in the model.

b. The cut value is .500

Variables in the Equation

		B	S.E.	Wald	df	Sig.	Exp(B)
Step 0	Constant	-3.057	.051	3588.344	1	.000	.047

Variables not in the Equation

			Score	df	Sig.
Step 0	Variables	Lifetimeabuseindex	52.389	7	<.001
		Lifetimeabuseindex(1)	.072	1	.789
		Lifetimeabuseindex(2)	.369	1	.544
		Lifetimeabuseindex(3)	11.026	1	<.001
		Lifetimeabuseindex(4)	.216	1	.642
		Lifetimeabuseindex(5)	1.437	1	.231
		Lifetimeabuseindex(6)	12.693	1	<.001
		Lifetimeabuseindex(7)	16.586	1	<.001
	Overall Statistics		52.389	7	<.001

Block 1: Method = Enter

Omnibus Tests of Model Coefficients

		Chi-square	df	Sig.
Step 1	Step	45.419	7	<.001
	Block	45.419	7	<.001
	Model	45.419	7	<.001

Model Summary

Step	-2 Log likelihood	Cox & Snell R Square	Nagelkerke R Square
1	3235.117[a]	.005	.016

a. Estimation terminated at iteration number 6 because parameter estimates changed by less than .001.

Classification Table[a]

Observed			Predicted IBS 1.00	Predicted IBS 2.00	Predicted Percentage Correct
Step 1	IBS	1.00	8549	0	100.0
		2.00	402	0	.0
	Overall Percentage				95.5

a. The cut value is .500

Variables in the Equation

		B	S.E.	Wald	df	Sig.
Step 1[a]	Lifetimeabuseindex			49.784	7	<.001
	Lifetimeabuseindex(1)	.224	.169	1.750	1	.186
	Lifetimeabuseindex(2)	.388	.227	2.914	1	.088
	Lifetimeabuseindex(3)	.871	.205	18.002	1	<.001
	Lifetimeabuseindex(4)	.331	.176	3.554	1	.059
	Lifetimeabuseindex(5)	.492	.212	5.367	1	.021
	Lifetimeabuseindex(6)	.996	.227	19.193	1	<.001
	Lifetimeabuseindex(7)	.962	.195	24.287	1	<.001
	Constant	-3.319	.077	1870.729	1	.000

Variables in the Equation

		Exp(B)	95% C.I.for EXP(B)	
			Lower	Upper
Step 1[a]	Lifetimeabuseindex			
	Lifetimeabuseindex(1)	1.251	.898	1.742
	Lifetimeabuseindex(2)	1.474	.944	2.302
	Lifetimeabuseindex(3)	2.388	1.597	3.571
	Lifetimeabuseindex(4)	1.393	.987	1.966
	Lifetimeabuseindex(5)	1.636	1.079	2.480
	Lifetimeabuseindex(6)	2.706	1.734	4.224
	Lifetimeabuseindex(7)	2.616	1.785	3.835
	Constant	.036		

a. Variable(s) entered on step 1: Lifetimeabuseindex.

Logistic Regression

Notes

Output Created		05-OCT-2024 11:47:38
Comments		
Input	Data	/Users/stevenlc/Library/CloudStorage/OneDrive-Privat/ICloud filer/Doktorander/Rickard/Artikel 3/Revision art 3/Artikel3_revision.sav
	Active Dataset	DataSet7
	Filter	<none>
	Weight	<none>
	Split File	<none>
	N of Rows in Working Data File	10337
Missing Value Handling	Definition of Missing	User-defined missing values are treated as missing
Syntax		LOGISTIC REGRESSION VARIABLES IBS /METHOD=ENTER Lifetimeabuseindex Householddysf_parent Kon barnboendeny utbildningmammapappa_ny fodelselandmammapappa_ny alderskategorier /CONTRAST (Lifetimeabuseindex)=Indicator(1) /CONTRAST (Householddysf_parent)=Indicator(1) /CONTRAST (Kon)=Indicator(1) /CONTRAST (barnboendeny)=Indicator(1) /CONTRAST (utbildningmammapappa_ny)=Indicator(1) /CONTRAST (fodelselandmammapappa_ny)=Indicator(1) /CONTRAST (alderskategorier)=Indicator(1) /PRINT=CI(95) /CRITERIA=PIN(0.05) POUT(0.10) ITERATE(20) CUT(0.5).
Resources	Processor Time	00:00:00,47
	Elapsed Time	00:00:00,00

Case Processing Summary

Unweighted Cases[a]		N	Percent
Selected Cases	Included in Analysis	7471	72.3
	Missing Cases	2866	27.7
	Total	10337	100.0
Unselected Cases		0	.0
Total		10337	100.0

a. If weight is in effect, see classification table for the total number of cases.

Dependent Variable Encoding

Original Value	Internal Value
1.00	0
2.00	1

Categorical Variables Codings

		Frequency	Parameter coding		
			(1)	(2)	(3)
Lifetimeabuseindex	.00	4321	.000	.000	.000
	1.00	865	1.000	.000	.000
	2.00	353	.000	1.000	.000
	3.00	273	.000	.000	1.000
	4.00	739	.000	.000	.000
	5.00	399	.000	.000	.000
	6.00	222	.000	.000	.000
	7.00	299	.000	.000	.000
Age categories	17-25	888	.000	.000	.000
	26-35	1171	1.000	.000	.000
	36-45	1403	.000	1.000	.000
	46-55	1480	.000	.000	1.000
	56-65	1561	.000	.000	.000
	66-74	968	.000	.000	.000
Parent education	At least one parent high school	3968	.000		
	Both parents below high school	3503	1.000		
Householddysf_parent	.00	5656	.000		
	1.00	1815	1.000		
Sex	Man	3341	.000		
	Kvinna	4130	1.000		
Immigrant status	At least one Nordic parent	6995	.000		
	Both parents born elsewhere	476	1.000		
Residence type	Owned home	5588	.000		
	Rental	1883	1.000		

Categorical Variables Codings

		Parameter coding (4)	(5)	(6)	(7)
Lifetimeabuseindex	.00	.000	.000	.000	.000
	1.00	.000	.000	.000	.000
	2.00	.000	.000	.000	.000
	3.00	.000	.000	.000	.000
	4.00	1.000	.000	.000	.000
	5.00	.000	1.000	.000	.000
	6.00	.000	.000	1.000	.000
	7.00	.000	.000	.000	1.000
Age categories	17-25	.000	.000		
	26-35	.000	.000		
	36-45	.000	.000		
	46-55	.000	.000		
	56-65	1.000	.000		
	66-74	.000	1.000		
Parent education	At least one parent high school				
	Both parents below high school				
Householddysf_parent	.00				
	1.00				
Sex	Man				
	Kvinna				
Immigrant status	At least one Nordic parent				
	Both parents born elsewhere				
Residence type	Owned home				
	Rental				

Block 0: Beginning Block

Classification Table[a,b]

Observed			Predicted IBS 1.00	2.00	Percentage Correct
Step 0	IBS	1.00	7132	0	100.0
		2.00	339	0	.0
	Overall Percentage				95.5

a. Constant is included in the model.

b. The cut value is .500

Variables in the Equation

		B	S.E.	Wald	df	Sig.	Exp(B)
Step 0	Constant	-3.046	.056	3003.247	1	.000	.048

Variables not in the Equation

			Score	df	Sig.
Step 0	Variables	Lifetimeabuseindex	53.443	7	<.001
		Lifetimeabuseindex(1)	1.179	1	.278
		Lifetimeabuseindex(2)	1.704	1	.192
		Lifetimeabuseindex(3)	5.086	1	.024
		Lifetimeabuseindex(4)	1.036	1	.309
		Lifetimeabuseindex(5)	1.465	1	.226
		Lifetimeabuseindex(6)	15.245	1	<.001
		Lifetimeabuseindex(7)	19.156	1	<.001
		Householddysf_parent(1)	4.112	1	.043
		Sex(1)	55.442	1	<.001
		Residence type(1)	11.561	1	<.001
		Parent education(1)	.617	1	.432
		Immigrant status(1)	1.624	1	.203
		Age categories	15.720	5	.008
		Age categories(1)	4.033	1	.045
		Age categories(2)	.002	1	.962
		Age categories(3)	1.197	1	.274
		Age categories(4)	1.933	1	.164
		Age categories(5)	3.362	1	.067
	Overall Statistics		129.513	17	<.001

Block 1: Method = Enter

Omnibus Tests of Model Coefficients

		Chi-square	df	Sig.
Step 1	Step	125.655	17	<.001
	Block	125.655	17	<.001
	Model	125.655	17	<.001

Model Summary

Step	-2 Log likelihood	Cox & Snell R Square	Nagelkerke R Square
1	2633.632[a]	.017	.054

a. Estimation terminated at iteration number 6 because parameter estimates changed by less than .001.

Classification Table[a]

Observed			Predicted IBS 1.00	Predicted IBS 2.00	Percentage Correct
Step 1	IBS	1.00	7132	0	100.0
		2.00	339	0	.0
	Overall Percentage				95.5

a. The cut value is .500

Variables in the Equation

		B	S.E.	Wald	df	Sig.
Step 1[a]	Lifetimeabuseindex			34.116	7	<.001
	Lifetimeabuseindex(1)	.037	.198	.034	1	.853
	Lifetimeabuseindex(2)	.488	.244	4.002	1	.045
	Lifetimeabuseindex(3)	.666	.256	6.777	1	.009
	Lifetimeabuseindex(4)	.434	.186	5.442	1	.020
	Lifetimeabuseindex(5)	.440	.234	3.529	1	.060
	Lifetimeabuseindex(6)	.969	.245	15.631	1	<.001
	Lifetimeabuseindex(7)	.929	.225	16.972	1	<.001
	Householddysf_parent(1)	.075	.138	.292	1	.589
	Sex(1)	.902	.129	49.088	1	<.001
	Residence type(1)	.231	.127	3.321	1	.068
	Parent education(1)	-.181	.127	2.013	1	.156
	Immigrant status(1)	-.408	.267	2.335	1	.126
	Age categories			17.128	5	.004
	Age categories(1)	.291	.264	1.213	1	.271
	Age categories(2)	.586	.248	5.588	1	.018
	Age categories(3)	.671	.249	7.258	1	.007
	Age categories(4)	.799	.255	9.818	1	.002
	Age categories(5)	.997	.272	13.464	1	<.001
	Constant	-4.458	.243	336.961	1	<.001

Variables in the Equation

		Exp(B)	95% C.I.for EXP(B) Lower	Upper
Step 1[a]	Lifetimeabuseindex			
	Lifetimeabuseindex(1)	1.037	.704	1.528
	Lifetimeabuseindex(2)	1.628	1.010	2.626
	Lifetimeabuseindex(3)	1.947	1.179	3.214
	Lifetimeabuseindex(4)	1.544	1.072	2.224
	Lifetimeabuseindex(5)	1.553	.981	2.458
	Lifetimeabuseindex(6)	2.637	1.630	4.263
	Lifetimeabuseindex(7)	2.531	1.627	3.937
	Householddysf_parent(1)	1.078	.822	1.412
	Sex(1)	2.463	1.914	3.170
	Residence type(1)	1.260	.983	1.614
	Parent education(1)	.835	.650	1.071
	Immigrant status(1)	.665	.394	1.122
	Age categories			
	Age categories(1)	1.338	.797	2.247
	Age categories(2)	1.796	1.105	2.918
	Age categories(3)	1.957	1.201	3.189
	Age categories(4)	2.224	1.349	3.668
	Age categories(5)	2.711	1.591	4.619
	Constant	.012		

a. Variable(s) entered on step 1: Lifetimeabuseindex, Householddysf_parent, Sex, Residence type, Parent education, Immigrant status, Age categories.

Logistic Regression

Notes

Output Created		05-OCT-2024 11:47:38
Comments		
Input	Data	/Users/stevenlc/Library/CloudStorage/OneDrive-Privat/ICloud filer/Doktorander/Rickard/Artikel 3/Revision art 3/Artikel3_revision.sav
	Active Dataset	DataSet7
	Filter	<none>
	Weight	<none>
	Split File	<none>
	N of Rows in Working Data File	10337
Missing Value Handling	Definition of Missing	User-defined missing values are treated as missing
Syntax		LOGISTIC REGRESSION VARIABLES Fibromyalgi /METHOD=ENTER Maltreatment_0to3 /CONTRAST (Maltreatment_0to3)=Indicator(1) /PRINT=CI(95) /CRITERIA=PIN(0.05) POUT(0.10) ITERATE(20) CUT(0.5).
Resources	Processor Time	00:00:00,39
	Elapsed Time	00:00:01,00

Case Processing Summary

Unweighted Cases[a]		N	Percent
Selected Cases	Included in Analysis	9169	88.7
	Missing Cases	1168	11.3
	Total	10337	100.0
Unselected Cases		0	.0
Total		10337	100.0

a. If weight is in effect, see classification table for the total number of cases.

Dependent Variable Encoding

Original Value	Internal Value
1.00	0
2.00	1

Categorical Variables Codings

		Frequency	Parameter coding (1)	(2)	(3)
Maltreatment_0to3	.00	6043	.000	.000	.000
	1.00	1589	1.000	.000	.000
	2.00	744	.000	1.000	.000
	3.00	793	.000	.000	1.000

Block 0: Beginning Block

Classification Table[a,b]

Observed			Predicted Fibromyalgi 1.00	2.00	Percentage Correct
Step 0	Fibromyalgi	1.00	8988	0	100.0
		2.00	181	0	.0
	Overall Percentage				98.0

a. Constant is included in the model.

b. The cut value is .500

Variables in the Equation

		B	S.E.	Wald	df	Sig.	Exp(B)
Step 0	Constant	-3.905	.075	2705.794	1	.000	.020

Variables not in the Equation

			Score	df	Sig.
Step 0	Variables	Maltreatment_0to3	36.682	3	<.001
		Maltreatment_0to3(1)	1.248	1	.264
		Maltreatment_0to3(2)	5.224	1	.022
		Maltreatment_0to3(3)	24.010	1	<.001
	Overall Statistics		36.682	3	<.001

Block 1: Method = Enter

Omnibus Tests of Model Coefficients

		Chi-square	df	Sig.
Step 1	Step	31.398	3	<.001
	Block	31.398	3	<.001
	Model	31.398	3	<.001

Model Summary

Step	-2 Log likelihood	Cox & Snell R Square	Nagelkerke R Square
1	1747.886[a]	.003	.019

a. Estimation terminated at iteration number 7 because parameter estimates changed by less than .001.

Classification Table[a]

Observed			Predicted: Fibromyalgi 1.00	Predicted: Fibromyalgi 2.00	Predicted: Percentage Correct
Step 1	Fibromyalgi	1.00	8988	0	100.0
		2.00	181	0	.0
	Overall Percentage				98.0

a. The cut value is .500

Variables in the Equation

		B	S.E.	Wald	df	Sig.
Step 1[a]	Maltreatment_0to3			34.142	3	<.001
	Maltreatment_0to3(1)	.490	.198	6.101	1	.014
	Maltreatment_0to3(2)	.781	.238	10.793	1	.001
	Maltreatment_0to3(3)	1.121	.206	29.623	1	<.001
	Constant	-4.226	.108	1531.549	1	.000

Variables in the Equation

		Exp(B)	95% C.I.for EXP(B) Lower	95% C.I.for EXP(B) Upper
Step 1[a]	Maltreatment_0to3			
	Maltreatment_0to3(1)	1.632	1.106	2.407
	Maltreatment_0to3(2)	2.184	1.370	3.480
	Maltreatment_0to3(3)	3.067	2.048	4.591
	Constant	.015		

a. Variable(s) entered on step 1: Maltreatment_0to3.

Logistic Regression

Notes

Output Created		05-OCT-2024 11:47:39
Comments		
Input	Data	/Users/stevenlc/Library/CloudStorage/OneDrive-Privat/ICloud filer/Doktorander/Rickard/Artikel 3/Revision art 3/Artikel3_revision.sav
	Active Dataset	DataSet7
	Filter	<none>
	Weight	<none>
	Split File	<none>
	N of Rows in Working Data File	10337
Missing Value Handling	Definition of Missing	User-defined missing values are treated as missing
Syntax		LOGISTIC REGRESSION VARIABLES Fibromyalgi /METHOD=ENTER Maltreatment_0to3 Householddysf_parent Kon barnboendeny utbildningmammapappa_ny fodelselandmammapappa_ny alderskategorier /CONTRAST (Maltreatment_0to3)=Indicator(1) /CONTRAST (Householddysf_parent)=Indicator(1) /CONTRAST (Kon)=Indicator(1) /CONTRAST (barnboendeny)=Indicator(1) /CONTRAST (utbildningmammapappa_ny)=Indicator(1) /CONTRAST (fodelselandmammapappa_ny)=Indicator(1) /CONTRAST (alderskategorier)=Indicator(1) /PRINT=CI(95) /CRITERIA=PIN(0.05) POUT(0.10) ITERATE(20) CUT(0.5).
Resources	Processor Time	00:00:00,47
	Elapsed Time	00:00:00,00

Case Processing Summary

Unweighted Cases[a]		N	Percent
Selected Cases	Included in Analysis	7629	73.8
	Missing Cases	2708	26.2
	Total	10337	100.0
Unselected Cases		0	.0
Total		10337	100.0

a. If weight is in effect, see classification table for the total number of cases.

Dependent Variable Encoding

Original Value	Internal Value
1.00	0
2.00	1

Categorical Variables Codings

		Frequency	Parameter coding (1)	(2)	(3)
Age categories	17-25	911	.000	.000	.000
	26-35	1194	1.000	.000	.000
	36-45	1430	.000	1.000	.000
	46-55	1512	.000	.000	1.000
	56-65	1592	.000	.000	.000
	66-74	990	.000	.000	.000
Maltreatment_0to3	.00	5160	.000	.000	.000
	1.00	1292	1.000	.000	.000
	2.00	590	.000	1.000	.000
	3.00	587	.000	.000	1.000
Parent education	At least one parent high school	4067	.000		
	Both parents below high school	3562	1.000		
Householddysf_parent	.00	5768	.000		
	1.00	1861	1.000		
Sex	Man	3417	.000		
	Kvinna	4212	1.000		
Immigrant status	At least one Nordic parent	7143	.000		
	Both parents born elsewhere	486	1.000		
Residence type	Owned home	5713	.000		
	Rental	1916	1.000		

Categorical Variables Codings

		Parameter coding (4)	Parameter coding (5)
Age categories	17-25	.000	.000
	26-35	.000	.000
	36-45	.000	.000
	46-55	.000	.000
	56-65	1.000	.000
	66-74	.000	1.000
Maltreatment_0to3	.00		
	1.00		
	2.00		
	3.00		
Parent education	At least one parent high school		
	Both parents below high school		
Householddysf_parent	.00		
	1.00		
Sex	Man		
	Kvinna		
Immigrant status	At least one Nordic parent		
	Both parents born elsewhere		
Residence type	Owned home		
	Rental		

Block 0: Beginning Block

Classification Table[a,b]

Observed			Predicted Fibromyalgi 1.00	Predicted Fibromyalgi 2.00	Percentage Correct
Step 0	Fibromyalgi	1.00	7473	0	100.0
		2.00	156	0	.0
	Overall Percentage				98.0

a. Constant is included in the model.

b. The cut value is .500

Variables in the Equation

		B	S.E.	Wald	df	Sig.	Exp(B)
Step 0	Constant	-3.869	.081	2287.670	1	.000	.021

Variables not in the Equation

			Score	df	Sig.
Step 0	Variables	Maltreatment_0to3	20.070	3	<.001
		Maltreatment_0to3(1)	.976	1	.323
		Maltreatment_0to3(2)	4.411	1	.036
		Maltreatment_0to3(3)	11.143	1	<.001
		Householddysf_parent(1)	.000	1	.992
		Sex(1)	71.205	1	<.001
		Residence type(1)	9.845	1	.002
		Parent education(1)	40.325	1	<.001
		Immigrant status(1)	3.868	1	.049
		Age categories	65.262	5	<.001
		Age categories(1)	16.811	1	<.001
		Age categories(2)	3.669	1	.055
		Age categories(3)	1.523	1	.217
		Age categories(4)	25.661	1	<.001
		Age categories(5)	10.966	1	<.001
	Overall Statistics		168.720	13	<.001

Block 1: Method = Enter

Omnibus Tests of Model Coefficients

		Chi-square	df	Sig.
Step 1	Step	192.432	13	<.001
	Block	192.432	13	<.001
	Model	192.432	13	<.001

Model Summary

Step	-2 Log likelihood	Cox & Snell R Square	Nagelkerke R Square
1	1329.991[a]	.025	.138

a. Estimation terminated at iteration number 10 because parameter estimates changed by less than .001.

Classification Table[a]

	Observed		Predicted: Fibromyalgi 1.00	Predicted: Fibromyalgi 2.00	Percentage Correct
Step 1	Fibromyalgi	1.00	7473	0	100.0
		2.00	156	0	.0
	Overall Percentage				98.0

a. The cut value is .500

Variables in the Equation

		B	S.E.	Wald	df	Sig.
Step 1[a]	Maltreatment_0to3			10.178	3	.017
	Maltreatment_0to3(1)	.365	.218	2.797	1	.094
	Maltreatment_0to3(2)	.515	.267	3.736	1	.053
	Maltreatment_0to3(3)	.738	.261	8.007	1	.005
	Householddysf_parent(1)	.040	.212	.036	1	.849
	Sex(1)	1.916	.253	57.373	1	<.001
	Residence type(1)	.130	.179	.527	1	.468
	Parent education(1)	.468	.195	5.771	1	.016
	Immigrant status(1)	-.700	.517	1.833	1	.176
	Age categories			29.929	5	<.001
	Age categories(1)	1.523	1.082	1.979	1	.159
	Age categories(2)	2.437	1.029	5.613	1	.018
	Age categories(3)	2.863	1.022	7.855	1	.005
	Age categories(4)	3.278	1.021	10.307	1	.001
	Age categories(5)	3.282	1.029	10.166	1	.001
	Constant	-8.535	1.032	68.366	1	<.001

Variables in the Equation

		Exp(B)	95% C.I.for EXP(B)	
			Lower	Upper
Step 1[a]	Maltreatment_0to3			
	Maltreatment_0to3(1)	1.440	.939	2.209
	Maltreatment_0to3(2)	1.674	.993	2.823
	Maltreatment_0to3(3)	2.092	1.255	3.489
	Householddysf_parent(1)	1.041	.688	1.577
	Sex(1)	6.794	4.138	11.154
	Residence type(1)	1.139	.802	1.618
	Parent education(1)	1.597	1.090	2.340
	Immigrant status(1)	.496	.180	1.368
	Age categories			
	Age categories(1)	4.585	.549	38.253
	Age categories(2)	11.442	1.523	85.945
	Age categories(3)	17.518	2.365	129.750
	Age categories(4)	26.528	3.586	196.266
	Age categories(5)	26.636	3.542	200.320
	Constant	.000		

a. Variable(s) entered on step 1: Maltreatment_0to3, Householddysf_parent, Sex, Residence type, Parent education, Immigrant status, Age categories.

Logistic Regression

Notes

Output Created		05-OCT-2024 11:47:39
Comments		
Input	Data	/Users/stevenlc/Library/CloudStorage/OneDrive-Privat/ICloud filer/Doktorander/Rickard/Artikel 3/Revision art 3/Artikel3_revision.sav
	Active Dataset	DataSet7
	Filter	<none>
	Weight	<none>
	Split File	<none>
	N of Rows in Working Data File	10337
Missing Value Handling	Definition of Missing	User-defined missing values are treated as missing
Syntax		LOGISTIC REGRESSION VARIABLES Fibromyalgi /METHOD=ENTER Lifetimeabuseindex /CONTRAST (Lifetimeabuseindex)=Indicator(1) /PRINT=CI(95) /CRITERIA=PIN(0.05) POUT(0.10) ITERATE(20) CUT(0.5).
Resources	Processor Time	00:00:00,41
	Elapsed Time	00:00:01,00

Case Processing Summary

Unweighted Cases[a]		N	Percent
Selected Cases	Included in Analysis	8951	86.6
	Missing Cases	1386	13.4
	Total	10337	100.0
Unselected Cases		0	.0
Total		10337	100.0

a. If weight is in effect, see classification table for the total number of cases.

Dependent Variable Encoding

Original Value	Internal Value
1.00	0
2.00	1

Categorical Variables Codings

		Frequency	Parameter coding (1)	(2)	(3)	(4)	(5)
Lifetimeabuseindex	.00	5038	.000	.000	.000	.000	.000
	1.00	1062	1.000	.000	.000	.000	.000
	2.00	454	.000	1.000	.000	.000	.000
	3.00	377	.000	.000	1.000	.000	.000
	4.00	875	.000	.000	.000	1.000	.000
	5.00	483	.000	.000	.000	.000	1.000
	6.00	269	.000	.000	.000	.000	.000
	7.00	393	.000	.000	.000	.000	.000

Categorical Variables Codings

		Parameter coding (6)	(7)
Lifetimeabuseindex	.00	.000	.000
	1.00	.000	.000
	2.00	.000	.000
	3.00	.000	.000
	4.00	.000	.000
	5.00	.000	.000
	6.00	1.000	.000
	7.00	.000	1.000

Block 0: Beginning Block

Classification Table[a,b]

Observed			Predicted: Fibromyalgi 1.00	Fibromyalgi 2.00	Percentage Correct
Step 0	Fibromyalgi	1.00	8775	0	100.0
		2.00	176	0	.0
	Overall Percentage				98.0

a. Constant is included in the model.

b. The cut value is .500

Variables in the Equation

		B	S.E.	Wald	df	Sig.	Exp(B)
Step 0	Constant	-3.909	.076	2636.690	1	.000	.020

Variables not in the Equation

			Score	df	Sig.
Step 0	Variables	Lifetimeabuseindex	43.383	7	<.001
		Lifetimeabuseindex(1)	.043	1	.836
		Lifetimeabuseindex(2)	3.098	1	.078
		Lifetimeabuseindex(3)	3.023	1	.082
		Lifetimeabuseindex(4)	.513	1	.474
		Lifetimeabuseindex(5)	3.438	1	.064
		Lifetimeabuseindex(6)	2.738	1	.098
		Lifetimeabuseindex(7)	20.795	1	<.001
	Overall Statistics		43.383	7	<.001

Block 1: Method = Enter

Omnibus Tests of Model Coefficients

		Chi-square	df	Sig.
Step 1	Step	37.031	7	<.001
	Block	37.031	7	<.001
	Model	37.031	7	<.001

Model Summary

Step	-2 Log likelihood	Cox & Snell R Square	Nagelkerke R Square
1	1694.506[a]	.004	.023

a. Estimation terminated at iteration number 7 because parameter estimates changed by less than .001.

Classification Table[a]

			Predicted		
			Fibromyalgi		Percentage Correct
	Observed		1.00	2.00	
Step 1	Fibromyalgi	1.00	8775	0	100.0
		2.00	176	0	.0
	Overall Percentage				98.0

a. The cut value is .500

Variables in the Equation

		B	S.E.	Wald	df	Sig.
Step 1[a]	Lifetimeabuseindex			39.681	7	<.001
	Lifetimeabuseindex(1)	.369	.258	2.051	1	.152
	Lifetimeabuseindex(2)	.874	.298	8.582	1	.003
	Lifetimeabuseindex(3)	.907	.318	8.110	1	.004
	Lifetimeabuseindex(4)	.567	.258	4.825	1	.028
	Lifetimeabuseindex(5)	.882	.290	9.233	1	.002
	Lifetimeabuseindex(6)	.958	.361	7.050	1	.008
	Lifetimeabuseindex(7)	1.396	.261	28.648	1	<.001
	Constant	-4.322	.124	1216.665	1	<.001

Variables in the Equation

			95% C.I.for EXP(B)	
		Exp(B)	Lower	Upper
Step 1[a]	Lifetimeabuseindex			
	Lifetimeabuseindex(1)	1.446	.873	2.395
	Lifetimeabuseindex(2)	2.397	1.336	4.302
	Lifetimeabuseindex(3)	2.477	1.327	4.623
	Lifetimeabuseindex(4)	1.762	1.063	2.921
	Lifetimeabuseindex(5)	2.415	1.367	4.263
	Lifetimeabuseindex(6)	2.608	1.285	5.291
	Lifetimeabuseindex(7)	4.039	2.423	6.735
	Constant	.013		

a. Variable(s) entered on step 1: Lifetimeabuseindex.

Logistic Regression

Notes

Output Created		05-OCT-2024 11:47:40
Comments		
Input	Data	/Users/stevenlc/Library/CloudStorage/OneDrive-Privat/ICloud filer/Doktorander/Rickard/Artikel 3/Revision art 3/Artikel3_revision.sav
	Active Dataset	DataSet7
	Filter	<none>
	Weight	<none>
	Split File	<none>
	N of Rows in Working Data File	10337
Missing Value Handling	Definition of Missing	User-defined missing values are treated as missing
Syntax		LOGISTIC REGRESSION VARIABLES Fibromyalgi /METHOD=ENTER Lifetimeabuseindex Householddysf_parent Kon barnboendeny utbildningmammapappa_ny fodelselandmammapappa_ny alderskategorier /CONTRAST (Lifetimeabuseindex)=Indicator(1) /CONTRAST (Householddysf_parent)=Indicator(1) /CONTRAST (Kon)=Indicator(1) /CONTRAST (barnboendeny)=Indicator(1) /CONTRAST (utbildningmammapappa_ny)=Indicator(1) /CONTRAST (fodelselandmammapappa_ny)=Indicator(1) /CONTRAST (alderskategorier)=Indicator(1) /PRINT=CI(95) /CRITERIA=PIN(0.05) POUT(0.10) ITERATE(20) CUT(0.5).
Resources	Processor Time	00:00:00,50
	Elapsed Time	00:00:00,00

Case Processing Summary

Unweighted Cases[a]		N	Percent
Selected Cases	Included in Analysis	7471	72.3
	Missing Cases	2866	27.7
	Total	10337	100.0
Unselected Cases		0	.0
Total		10337	100.0

a. If weight is in effect, see classification table for the total number of cases.

Dependent Variable Encoding

Original Value	Internal Value
1.00	0
2.00	1

Categorical Variables Codings

		Frequency	Parameter coding (1)	(2)	(3)
Lifetimeabuseindex	.00	4321	.000	.000	.000
	1.00	865	1.000	.000	.000
	2.00	353	.000	1.000	.000
	3.00	273	.000	.000	1.000
	4.00	739	.000	.000	.000
	5.00	399	.000	.000	.000
	6.00	222	.000	.000	.000
	7.00	299	.000	.000	.000
Age categories	17-25	888	.000	.000	.000
	26-35	1171	1.000	.000	.000
	36-45	1403	.000	1.000	.000
	46-55	1480	.000	.000	1.000
	56-65	1561	.000	.000	.000
	66-74	968	.000	.000	.000
Parent education	At least one parent high school	3968	.000		
	Both parents below high school	3503	1.000		
Householddysf_parent	.00	5656	.000		
	1.00	1815	1.000		
Sex	Man	3341	.000		
	Kvinna	4130	1.000		
Immigrant status	At least one Nordic parent	6995	.000		
	Both parents born elsewhere	476	1.000		
Residence type	Owned home	5588	.000		
	Rental	1883	1.000		

Categorical Variables Codings

		Parameter coding (4)	(5)	(6)	(7)
Lifetimeabuseindex	.00	.000	.000	.000	.000
	1.00	.000	.000	.000	.000
	2.00	.000	.000	.000	.000
	3.00	.000	.000	.000	.000
	4.00	1.000	.000	.000	.000
	5.00	.000	1.000	.000	.000
	6.00	.000	.000	1.000	.000
	7.00	.000	.000	.000	1.000
Age categories	17-25	.000	.000		
	26-35	.000	.000		
	36-45	.000	.000		
	46-55	.000	.000		
	56-65	1.000	.000		
	66-74	.000	1.000		
Parent education	At least one parent high school				
	Both parents below high school				
Householddysf_parent	.00				
	1.00				
Sex	Man				
	Kvinna				
Immigrant status	At least one Nordic parent				
	Both parents born elsewhere				
Residence type	Owned home				
	Rental				

Block 0: Beginning Block

Classification Table[a,b]

Observed			Predicted Fibromyalgi 1.00	Fibromyalgi 2.00	Percentage Correct
Step 0	Fibromyalgi	1.00	7318	0	100.0
		2.00	153	0	.0
	Overall Percentage				98.0

a. Constant is included in the model.

b. The cut value is .500

Variables in the Equation

		B	S.E.	Wald	df	Sig.	Exp(B)
Step 0	Constant	-3.868	.082	2241.818	1	.000	.021

Variables not in the Equation

			Score	df	Sig.
Step 0	Variables	Lifetimeabuseindex	25.524	7	<.001
		Lifetimeabuseindex(1)	.005	1	.942
		Lifetimeabuseindex(2)	2.108	1	.147
		Lifetimeabuseindex(3)	3.685	1	.055
		Lifetimeabuseindex(4)	1.773	1	.183
		Lifetimeabuseindex(5)	1.935	1	.164
		Lifetimeabuseindex(6)	2.760	1	.097
		Lifetimeabuseindex(7)	5.998	1	.014
		Householddysf_parent(1)	.025	1	.874
		Sex(1)	71.368	1	<.001
		Residence type(1)	9.564	1	.002
		Parent education(1)	37.201	1	<.001
		Immigrant status(1)	3.696	1	.055
		Age categories	61.852	5	<.001
		Age categories(1)	16.323	1	<.001
		Age categories(2)	3.336	1	.068
		Age categories(3)	1.880	1	.170
		Age categories(4)	21.415	1	<.001
		Age categories(5)	11.890	1	<.001
	Overall Statistics		173.040	17	<.001

Block 1: Method = Enter

Omnibus Tests of Model Coefficients

		Chi-square	df	Sig.
Step 1	Step	196.319	17	<.001
	Block	196.319	17	<.001
	Model	196.319	17	<.001

Model Summary

Step	-2 Log likelihood	Cox & Snell R Square	Nagelkerke R Square
1	1296.360 [a]	.026	.143

a. Estimation terminated at iteration number 10 because parameter estimates changed by less than .001.

Classification Table[a]

Observed			Predicted: Fibromyalgi 1.00	Predicted: Fibromyalgi 2.00	Percentage Correct
Step 1	Fibromyalgi	1.00	7318	0	100.0
		2.00	153	0	.0
	Overall Percentage				98.0

a. The cut value is .500

Variables in the Equation

		B	S.E.	Wald	df	Sig.
Step 1[a]	Lifetimeabuseindex			17.116	7	.017
	Lifetimeabuseindex(1)	.328	.275	1.418	1	.234
	Lifetimeabuseindex(2)	.603	.341	3.133	1	.077
	Lifetimeabuseindex(3)	.776	.363	4.553	1	.033
	Lifetimeabuseindex(4)	.729	.267	7.457	1	.006
	Lifetimeabuseindex(5)	.781	.331	5.564	1	.018
	Lifetimeabuseindex(6)	.758	.393	3.711	1	.054
	Lifetimeabuseindex(7)	.918	.344	7.121	1	.008
	Householddysf_parent(1)	.062	.212	.085	1	.771
	Sex(1)	1.941	.260	55.806	1	<.001
	Residence type(1)	.123	.181	.463	1	.496
	Parent education(1)	.461	.197	5.481	1	.019
	Immigrant status(1)	-.677	.518	1.710	1	.191
	Age categories			30.309	5	<.001
	Age categories(1)	1.524	1.083	1.981	1	.159
	Age categories(2)	2.414	1.029	5.505	1	.019
	Age categories(3)	2.852	1.022	7.788	1	.005
	Age categories(4)	3.244	1.022	10.074	1	.002
	Age categories(5)	3.357	1.030	10.619	1	.001
	Constant	-8.683	1.036	70.252	1	<.001

Variables in the Equation

		Exp(B)	95% C.I.for EXP(B)	
			Lower	Upper
Step 1[a]	Lifetimeabuseindex			
	Lifetimeabuseindex(1)	1.388	.809	2.380
	Lifetimeabuseindex(2)	1.827	.937	3.561
	Lifetimeabuseindex(3)	2.172	1.065	4.427
	Lifetimeabuseindex(4)	2.072	1.228	3.496
	Lifetimeabuseindex(5)	2.183	1.141	4.176
	Lifetimeabuseindex(6)	2.133	.987	4.612
	Lifetimeabuseindex(7)	2.503	1.276	4.911
	Householddysf_parent(1)	1.064	.702	1.612
	Sex(1)	6.969	4.187	11.598
	Residence type(1)	1.131	.793	1.613
	Parent education(1)	1.586	1.078	2.332
	Immigrant status(1)	.508	.184	1.402
	Age categories			
	Age categories(1)	4.589	.550	38.304
	Age categories(2)	11.183	1.488	84.034
	Age categories(3)	17.329	2.337	128.465
	Age categories(4)	25.631	3.458	189.985
	Age categories(5)	28.701	3.811	216.156
	Constant	.000		

a. Variable(s) entered on step 1: Lifetimeabuseindex, Householddysf_parent, Sex, Residence type, Parent education, Immigrant status, Age categories.

Logistic Regression

Notes

Output Created		05-OCT-2024 11:47:40
Comments		
Input	Data	/Users/stevenlc/Library/CloudStorage/OneDrive-Privat/ICloud filer/Doktorander/Rickard/Artikel 3/Revision art 3/Artikel3_revision.sav
	Active Dataset	DataSet7
	Filter	<none>
	Weight	<none>
	Split File	<none>
	N of Rows in Working Data File	10337
Missing Value Handling	Definition of Missing	User-defined missing values are treated as missing
Syntax		LOGISTIC REGRESSION VARIABLES Ischemic_HD /METHOD=ENTER Maltreatment_0to3 /CONTRAST (Maltreatment_0to3)=Indicator(1) /PRINT=CI(95) /CRITERIA=PIN(0.05) POUT(0.10) ITERATE(20) CUT(0.5).
Resources	Processor Time	00:00:00,39
	Elapsed Time	00:00:01,00

Case Processing Summary

Unweighted Cases[a]		N	Percent
Selected Cases	Included in Analysis	9169	88.7
	Missing Cases	1168	11.3
	Total	10337	100.0
Unselected Cases		0	.0
Total		10337	100.0

a. If weight is in effect, see classification table for the total number of cases.

Dependent Variable Encoding

Original Value	Internal Value
1.00	0
2.00	1

Categorical Variables Codings

		Frequency	Parameter coding (1)	(2)	(3)
Maltreatment_0to3	.00	6043	.000	.000	.000
	1.00	1589	1.000	.000	.000
	2.00	744	.000	1.000	.000
	3.00	793	.000	.000	1.000

Block 0: Beginning Block

Classification Table[a,b]

Observed			Predicted Ischemic heart disease 1.00	2.00	Percentage Correct
Step 0	Ischemic heart disease	1.00	8731	0	100.0
		2.00	438	0	.0
	Overall Percentage				95.2

a. Constant is included in the model.

b. The cut value is .500

Variables in the Equation

		B	S.E.	Wald	df	Sig.	Exp(B)
Step 0	Constant	-2.992	.049	3734.738	1	.000	.050

Variables not in the Equation

			Score	df	Sig.
Step 0	Variables	Maltreatment_0to3	7.347	3	.062
		Maltreatment_0to3(1)	.141	1	.707
		Maltreatment_0to3(2)	6.723	1	.010
		Maltreatment_0to3(3)	.295	1	.587
	Overall Statistics		7.347	3	.062

Block 1: Method = Enter

Omnibus Tests of Model Coefficients

		Chi-square	df	Sig.
Step 1	Step	6.690	3	.082
	Block	6.690	3	.082
	Model	6.690	3	.082

Model Summary

Step	-2 Log likelihood	Cox & Snell R Square	Nagelkerke R Square
1	3512.281[a]	.001	.002

a. Estimation terminated at iteration number 6 because parameter estimates changed by less than .001.

Classification Table[a]

Observed			Predicted: Ischemic heart disease 1.00	2.00	Percentage Correct
Step 1	Ischemic heart disease	1.00	8731	0	100.0
		2.00	438	0	.0
	Overall Percentage				95.2

a. The cut value is .500

Variables in the Equation

		B	S.E.	Wald	df	Sig.
Step 1[a]	Maltreatment_0to3			7.259	3	.064
	Maltreatment_0to3(1)	.014	.135	.010	1	.919
	Maltreatment_0to3(2)	.417	.159	6.872	1	.009
	Maltreatment_0to3(3)	.138	.172	.644	1	.422
	Constant	-3.047	.062	2428.730	1	.000

Variables in the Equation

		Exp(B)	95% C.I.for EXP(B) Lower	Upper
Step 1[a]	Maltreatment_0to3			
	Maltreatment_0to3(1)	1.014	.778	1.321
	Maltreatment_0to3(2)	1.517	1.111	2.071
	Maltreatment_0to3(3)	1.148	.820	1.608
	Constant	.047		

a. Variable(s) entered on step 1: Maltreatment_0to3.

Logistic Regression

Notes

Output Created		05-OCT-2024 11:47:41
Comments		
Input	Data	/Users/stevenlc/Library/CloudStorage/OneDrive-Privat/ICloud filer/Doktorander/Rickard/Artikel 3/Revision art 3/Artikel3_revision.sav
	Active Dataset	DataSet7
	Filter	<none>
	Weight	<none>
	Split File	<none>
	N of Rows in Working Data File	10337
Missing Value Handling	Definition of Missing	User-defined missing values are treated as missing
Syntax		LOGISTIC REGRESSION VARIABLES Ischemic_HD /METHOD=ENTER Maltreatment_0to3 Householddysf_parent Kon barnboendeny utbildningmammapappa_ny fodelselandmammapappa_ny alderskategorier /CONTRAST (Maltreatment_0to3)=Indicator(1) /CONTRAST (Householddysf_parent)=Indicator(1) /CONTRAST (Kon)=Indicator(1) /CONTRAST (barnboendeny)=Indicator(1) /CONTRAST (utbildningmammapappa_ny)=Indicator(1) /CONTRAST (fodelselandmammapappa_ny)=Indicator(1) /CONTRAST (alderskategorier)=Indicator(1) /PRINT=CI(95) /CRITERIA=PIN(0.05) POUT(0.10) ITERATE(20) CUT(0.5).
Resources	Processor Time	00:00:00,46
	Elapsed Time	00:00:00,00

Case Processing Summary

Unweighted Cases[a]		N	Percent
Selected Cases	Included in Analysis	7629	73.8
	Missing Cases	2708	26.2
	Total	10337	100.0
Unselected Cases		0	.0
Total		10337	100.0

a. If weight is in effect, see classification table for the total number of cases.

Dependent Variable Encoding

Original Value	Internal Value
1.00	0
2.00	1

Categorical Variables Codings

		Frequency	Parameter coding (1)	(2)	(3)
Age categories	17-25	911	.000	.000	.000
	26-35	1194	1.000	.000	.000
	36-45	1430	.000	1.000	.000
	46-55	1512	.000	.000	1.000
	56-65	1592	.000	.000	.000
	66-74	990	.000	.000	.000
Maltreatment_0to3	.00	5160	.000	.000	.000
	1.00	1292	1.000	.000	.000
	2.00	590	.000	1.000	.000
	3.00	587	.000	.000	1.000
Parent education	At least one parent high school	4067	.000		
	Both parents below high school	3562	1.000		
Householddysf_parent	.00	5768	.000		
	1.00	1861	1.000		
Sex	Man	3417	.000		
	Kvinna	4212	1.000		
Immigrant status	At least one Nordic parent	7143	.000		
	Both parents born elsewhere	486	1.000		
Residence type	Owned home	5713	.000		
	Rental	1916	1.000		

Categorical Variables Codings

		Parameter coding (4)	Parameter coding (5)
Age categories	17-25	.000	.000
	26-35	.000	.000
	36-45	.000	.000
	46-55	.000	.000
	56-65	1.000	.000
	66-74	.000	1.000
Maltreatment_0to3	.00		
	1.00		
	2.00		
	3.00		
Parent education	At least one parent high school		
	Both parents below high school		
Householddysf_parent	.00		
	1.00		
Sex	Man		
	Kvinna		
Immigrant status	At least one Nordic parent		
	Both parents born elsewhere		
Residence type	Owned home		
	Rental		

Block 0: Beginning Block

Classification Table[a,b]

	Observed		Predicted: Ischemic heart disease 1.00	Predicted: Ischemic heart disease 2.00	Percentage Correct
Step 0	Ischemic heart disease	1.00	7277	0	100.0
		2.00	352	0	.0
	Overall Percentage				95.4

a. Constant is included in the model.

b. The cut value is .500

Variables in the Equation

		B	S.E.	Wald	df	Sig.	Exp(B)
Step 0	Constant	-3.029	.055	3080.214	1	.000	.048

Variables not in the Equation

			Score	df	Sig.
Step 0	Variables	Maltreatment_0to3	9.148	3	.027
		Maltreatment_0to3(1)	3.368	1	.066
		Maltreatment_0to3(2)	5.790	1	.016
		Maltreatment_0to3(3)	1.013	1	.314
		Householddysf_parent(1)	10.804	1	.001
		Sex(1)	68.365	1	<.001
		Residence type(1)	14.825	1	<.001
		Parent education(1)	98.328	1	<.001
		Immigrant status(1)	2.061	1	.151
		Age categories	426.406	5	<.001
		Age categories(1)	45.870	1	<.001
		Age categories(2)	45.016	1	<.001
		Age categories(3)	14.446	1	<.001
		Age categories(4)	37.414	1	<.001
		Age categories(5)	320.990	1	<.001
	Overall Statistics		484.860	13	<.001

Block 1: Method = Enter

Omnibus Tests of Model Coefficients

		Chi-square	df	Sig.
Step 1	Step	431.785	13	<.001
	Block	431.785	13	<.001
	Model	431.785	13	<.001

Model Summary

Step	-2 Log likelihood	Cox & Snell R Square	Nagelkerke R Square
1	2421.279[a]	.055	.176

a. Estimation terminated at iteration number 8 because parameter estimates changed by less than .001.

Classification Table[a]

	Observed		Predicted: Ischemic heart disease 1.00	Predicted: Ischemic heart disease 2.00	Predicted: Percentage Correct
Step 1	Ischemic heart disease	1.00	7277	0	100.0
		2.00	352	0	.0
	Overall Percentage				95.4

a. The cut value is .500

Variables in the Equation

		B	S.E.	Wald	df	Sig.
Step 1[a]	Maltreatment_0to3			7.378	3	.061
	Maltreatment_0to3(1)	-.161	.170	.901	1	.343
	Maltreatment_0to3(2)	.386	.190	4.158	1	.041
	Maltreatment_0to3(3)	.285	.212	1.816	1	.178
	Householddysf_parent(1)	.064	.163	.152	1	.696
	Sex(1)	-.839	.119	50.017	1	<.001
	Residence type(1)	.076	.124	.383	1	.536
	Parent education(1)	.218	.136	2.586	1	.108
	Immigrant status(1)	.001	.273	.000	1	.997
	Age categories			210.122	5	<.001
	Age categories(1)	.037	.496	.006	1	.940
	Age categories(2)	.399	.450	.787	1	.375
	Age categories(3)	1.152	.418	7.603	1	.006
	Age categories(4)	2.127	.405	27.591	1	<.001
	Age categories(5)	2.952	.406	52.957	1	<.001
	Constant	-4.519	.389	134.964	1	<.001

Variables in the Equation

		Exp(B)	95% C.I.for EXP(B) Lower	Upper
Step 1[a]	Maltreatment_0to3			
	Maltreatment_0to3(1)	.851	.610	1.187
	Maltreatment_0to3(2)	1.472	1.015	2.134
	Maltreatment_0to3(3)	1.330	.878	2.014
	Householddysf_parent(1)	1.066	.774	1.466
	Sex(1)	.432	.342	.545
	Residence type(1)	1.079	.847	1.375
	Parent education(1)	1.244	.953	1.623
	Immigrant status(1)	1.001	.586	1.710
	Age categories			
	Age categories(1)	1.038	.393	2.743
	Age categories(2)	1.491	.617	3.604
	Age categories(3)	3.165	1.395	7.177
	Age categories(4)	8.388	3.793	18.547
	Age categories(5)	19.136	8.642	42.374
	Constant	.011		

a. Variable(s) entered on step 1: Maltreatment_0to3, Householddysf_parent, Sex, Residence type, Parent education, Immigrant status, Age categories.

Logistic Regression

Notes

Output Created		05-OCT-2024 11:47:41
Comments		
Input	Data	/Users/stevenlc/Library/CloudStorage/OneDrive-Privat/ICloud filer/Doktorander/Rickard/Artikel 3/Revision art 3/Artikel3_revision.sav
	Active Dataset	DataSet7
	Filter	<none>
	Weight	<none>
	Split File	<none>
	N of Rows in Working Data File	10337
Missing Value Handling	Definition of Missing	User-defined missing values are treated as missing
Syntax		LOGISTIC REGRESSION VARIABLES Ischemic_HD<br>/METHOD=ENTER Lifetimeabuseindex<br>/CONTRAST (Lifetimeabuseindex)=Indicator(1)<br>/PRINT=CI(95)<br>/CRITERIA=PIN(0.05) POUT(0.10) ITERATE(20) CUT(0.5).
Resources	Processor Time	00:00:00,40
	Elapsed Time	00:00:00,00

Case Processing Summary

Unweighted Cases[a]		N	Percent
Selected Cases	Included in Analysis	8951	86.6
	Missing Cases	1386	13.4
	Total	10337	100.0
Unselected Cases		0	.0
Total		10337	100.0

a. If weight is in effect, see classification table for the total number of cases.

Dependent Variable Encoding

Original Value	Internal Value
1.00	0
2.00	1

Categorical Variables Codings

		Frequency	Parameter coding (1)	(2)	(3)	(4)	(5)
Lifetimeabuseindex	.00	5038	.000	.000	.000	.000	.000
	1.00	1062	1.000	.000	.000	.000	.000
	2.00	454	.000	1.000	.000	.000	.000
	3.00	377	.000	.000	1.000	.000	.000
	4.00	875	.000	.000	.000	1.000	.000
	5.00	483	.000	.000	.000	.000	1.000
	6.00	269	.000	.000	.000	.000	.000
	7.00	393	.000	.000	.000	.000	.000

Categorical Variables Codings

		Parameter coding (6)	(7)
Lifetimeabuseindex	.00	.000	.000
	1.00	.000	.000
	2.00	.000	.000
	3.00	.000	.000
	4.00	.000	.000
	5.00	.000	.000
	6.00	1.000	.000
	7.00	.000	1.000

Block 0: Beginning Block

Classification Table[a,b]

Observed			Predicted: Ischemic heart disease 1.00	2.00	Percentage Correct
Step 0	Ischemic heart disease	1.00	8521	0	100.0
		2.00	430	0	.0
	Overall Percentage				95.2

a. Constant is included in the model.

b. The cut value is .500

Variables in the Equation

		B	S.E.	Wald	df	Sig.	Exp(B)
Step 0	Constant	-2.987	.049	3651.015	1	.000	.050

Variables not in the Equation

			Score	df	Sig.
Step 0	Variables	Lifetimeabuseindex	15.648	7	.029
		Lifetimeabuseindex(1)	.023	1	.881
		Lifetimeabuseindex(2)	8.827	1	.003
		Lifetimeabuseindex(3)	1.447	1	.229
		Lifetimeabuseindex(4)	4.706	1	.030
		Lifetimeabuseindex(5)	.845	1	.358
		Lifetimeabuseindex(6)	.362	1	.548
		Lifetimeabuseindex(7)	.483	1	.487
	Overall Statistics		15.648	7	.029

Block 1: Method = Enter

Omnibus Tests of Model Coefficients

		Chi-square	df	Sig.
Step 1	Step	14.854	7	.038
	Block	14.854	7	.038
	Model	14.854	7	.038

Model Summary

Step	-2 Log likelihood	Cox & Snell R Square	Nagelkerke R Square
1	3434.882[a]	.002	.005

a. Estimation terminated at iteration number 6 because parameter estimates changed by less than .001.

Classification Table[a]

	Observed		Predicted		
			Ischemic heart disease		Percentage Correct
			1.00	2.00	
Step 1	Ischemic heart disease	1.00	8521	0	100.0
		2.00	430	0	.0
	Overall Percentage				95.2

a. The cut value is .500

Variables in the Equation

		B	S.E.	Wald	df	Sig.
Step 1[a]	Lifetimeabuseindex			15.301	7	.032
	Lifetimeabuseindex(1)	.024	.157	.024	1	.876
	Lifetimeabuseindex(2)	.508	.188	7.320	1	.007
	Lifetimeabuseindex(3)	.257	.225	1.305	1	.253
	Lifetimeabuseindex(4)	-.382	.200	3.651	1	.056
	Lifetimeabuseindex(5)	-.204	.243	.707	1	.400
	Lifetimeabuseindex(6)	.162	.274	.349	1	.555
	Lifetimeabuseindex(7)	-.169	.264	.409	1	.522
	Constant	-2.991	.066	2052.800	1	.000

Variables in the Equation

		Exp(B)	95% C.I.for EXP(B)	
			Lower	Upper
Step 1[a]	Lifetimeabuseindex			
	Lifetimeabuseindex(1)	1.025	.754	1.393
	Lifetimeabuseindex(2)	1.663	1.150	2.403
	Lifetimeabuseindex(3)	1.293	.832	2.010
	Lifetimeabuseindex(4)	.682	.461	1.010
	Lifetimeabuseindex(5)	.815	.506	1.313
	Lifetimeabuseindex(6)	1.175	.687	2.010
	Lifetimeabuseindex(7)	.845	.504	1.416
	Constant	.050		

a. Variable(s) entered on step 1: Lifetimeabuseindex.

Logistic Regression

Notes

Output Created		05-OCT-2024 11:47:41
Comments		
Input	Data	/Users/stevenlc/Library/CloudStorage/OneDrive-Privat/ICloud filer/Doktorander/Rickard/Artikel 3/Revision art 3/Artikel3_revision.sav
	Active Dataset	DataSet7
	Filter	<none>
	Weight	<none>
	Split File	<none>
	N of Rows in Working Data File	10337
Missing Value Handling	Definition of Missing	User-defined missing values are treated as missing
Syntax		LOGISTIC REGRESSION VARIABLES Ischemic_HD /METHOD=ENTER Lifetimeabuseindex Householddysf_parent Kon barnboendeny utbildningmammapappa_ny fodelselandmammapappa_ny alderskategorier /CONTRAST (Lifetimeabuseindex)=Indicator(1) /CONTRAST (Householddysf_parent)=Indicator(1) /CONTRAST (Kon)=Indicator(1) /CONTRAST (barnboendeny)=Indicator(1) /CONTRAST (utbildningmammapappa_ny)=Indicator(1) /CONTRAST (fodelselandmammapappa_ny)=Indicator(1) /CONTRAST (alderskategorier)=Indicator(1) /PRINT=CI(95) /CRITERIA=PIN(0.05) POUT(0.10) ITERATE(20) CUT(0.5).
Resources	Processor Time	00:00:00,49
	Elapsed Time	00:00:01,00

Case Processing Summary

Unweighted Cases[a]		N	Percent
Selected Cases	Included in Analysis	7471	72.3
	Missing Cases	2866	27.7
	Total	10337	100.0
Unselected Cases		0	.0
Total		10337	100.0

a. If weight is in effect, see classification table for the total number of cases.

Dependent Variable Encoding

Original Value	Internal Value
1.00	0
2.00	1

Categorical Variables Codings

		Frequency	Parameter coding		
			(1)	(2)	(3)
Lifetimeabuseindex	.00	4321	.000	.000	.000
	1.00	865	1.000	.000	.000
	2.00	353	.000	1.000	.000
	3.00	273	.000	.000	1.000
	4.00	739	.000	.000	.000
	5.00	399	.000	.000	.000
	6.00	222	.000	.000	.000
	7.00	299	.000	.000	.000
Age categories	17-25	888	.000	.000	.000
	26-35	1171	1.000	.000	.000
	36-45	1403	.000	1.000	.000
	46-55	1480	.000	.000	1.000
	56-65	1561	.000	.000	.000
	66-74	968	.000	.000	.000
Parent education	At least one parent high school	3968	.000		
	Both parents below high school	3503	1.000		
Householddysf_parent	.00	5656	.000		
	1.00	1815	1.000		
Sex	Man	3341	.000		
	Kvinna	4130	1.000		
Immigrant status	At least one Nordic parent	6995	.000		
	Both parents born elsewhere	476	1.000		
Residence type	Owned home	5588	.000		
	Rental	1883	1.000		

Categorical Variables Codings

		Parameter coding (4)	(5)	(6)	(7)
Lifetimeabuseindex	.00	.000	.000	.000	.000
	1.00	.000	.000	.000	.000
	2.00	.000	.000	.000	.000
	3.00	.000	.000	.000	.000
	4.00	1.000	.000	.000	.000
	5.00	.000	1.000	.000	.000
	6.00	.000	.000	1.000	.000
	7.00	.000	.000	.000	1.000
Age categories	17-25	.000	.000		
	26-35	.000	.000		
	36-45	.000	.000		
	46-55	.000	.000		
	56-65	1.000	.000		
	66-74	.000	1.000		
Parent education	At least one parent high school				
	Both parents below high school				
Householddysf_parent	.00				
	1.00				
Sex	Man				
	Kvinna				
Immigrant status	At least one Nordic parent				
	Both parents born elsewhere				
Residence type	Owned home				
	Rental				

Block 0: Beginning Block

Classification Table[a,b]

	Observed		Predicted: Ischemic heart disease 1.00	2.00	Percentage Correct
Step 0	Ischemic heart disease	1.00	7125	0	100.0
		2.00	346	0	.0
	Overall Percentage				95.4

a. Constant is included in the model.

b. The cut value is .500

Variables in the Equation

		B	S.E.	Wald	df	Sig.	Exp(B)
Step 0	Constant	-3.025	.055	3019.339	1	.000	.049

Variables not in the Equation

			Score	df	Sig.
Step 0	Variables	Lifetimeabuseindex	18.135	7	.011
		Lifetimeabuseindex(1)	.758	1	.384
		Lifetimeabuseindex(2)	7.638	1	.006
		Lifetimeabuseindex(3)	3.478	1	.062
		Lifetimeabuseindex(4)	2.300	1	.129
		Lifetimeabuseindex(5)	4.309	1	.038
		Lifetimeabuseindex(6)	.310	1	.577
		Lifetimeabuseindex(7)	.269	1	.604
		Householddysf_parent(1)	10.345	1	.001
		Sex(1)	64.028	1	<.001
		Residence type(1)	15.244	1	<.001
		Parent education(1)	100.259	1	<.001
		Immigrant status(1)	1.856	1	.173
		Age categories	422.102	5	<.001
		Age categories(1)	46.910	1	<.001
		Age categories(2)	43.846	1	<.001
		Age categories(3)	13.440	1	<.001
		Age categories(4)	35.025	1	<.001
		Age categories(5)	320.250	1	<.001
	Overall Statistics		477.865	17	<.001

Block 1: Method = Enter

Omnibus Tests of Model Coefficients

		Chi-square	df	Sig.
Step 1	Step	424.718	17	<.001
	Block	424.718	17	<.001
	Model	424.718	17	<.001

Model Summary

Step	-2 Log likelihood	Cox & Snell R Square	Nagelkerke R Square
1	2377.068[a]	.055	.177

a. Estimation terminated at iteration number 8 because parameter estimates changed by less than .001.

Classification Table[a]

Observed			Predicted: Ischemic heart disease 1.00	Predicted: Ischemic heart disease 2.00	Percentage Correct
Step 1	Ischemic heart disease	1.00	7125	0	100.0
		2.00	346	0	.0
	Overall Percentage				95.4

a. The cut value is .500

Variables in the Equation

		B	S.E.	Wald	df	Sig.
Step 1[a]	Lifetimeabuseindex			8.063	7	.327
	Lifetimeabuseindex(1)	-.162	.194	.694	1	.405
	Lifetimeabuseindex(2)	.393	.225	3.043	1	.081
	Lifetimeabuseindex(3)	.290	.267	1.184	1	.276
	Lifetimeabuseindex(4)	.053	.220	.058	1	.810
	Lifetimeabuseindex(5)	-.289	.338	.727	1	.394
	Lifetimeabuseindex(6)	.413	.320	1.658	1	.198
	Lifetimeabuseindex(7)	.256	.322	.632	1	.426
	Householddysf_parent(1)	.067	.164	.165	1	.685
	Sex(1)	-.808	.120	45.696	1	<.001
	Residence type(1)	.087	.124	.494	1	.482
	Parent education(1)	.245	.138	3.147	1	.076
	Immigrant status(1)	.047	.274	.030	1	.863
	Age categories			203.671	5	<.001
	Age categories(1)	-.074	.507	.022	1	.883
	Age categories(2)	.385	.451	.731	1	.393
	Age categories(3)	1.139	.418	7.415	1	.006
	Age categories(4)	2.080	.406	26.314	1	<.001
	Age categories(5)	2.919	.406	51.628	1	<.001
	Constant	-4.517	.390	133.861	1	<.001

Variables in the Equation

		Exp(B)	95% C.I.for EXP(B)	
			Lower	Upper
Step 1[a]	Lifetimeabuseindex			
	Lifetimeabuseindex(1)	.851	.581	1.245
	Lifetimeabuseindex(2)	1.481	.953	2.303
	Lifetimeabuseindex(3)	1.337	.793	2.255
	Lifetimeabuseindex(4)	1.054	.685	1.624
	Lifetimeabuseindex(5)	.749	.386	1.455
	Lifetimeabuseindex(6)	1.511	.806	2.830
	Lifetimeabuseindex(7)	1.291	.688	2.426
	Householddysf_parent(1)	1.069	.775	1.475
	Sex(1)	.446	.352	.563
	Residence type(1)	1.091	.855	1.393
	Parent education(1)	1.277	.975	1.674
	Immigrant status(1)	1.048	.613	1.794
	Age categories			
	Age categories(1)	.928	.344	2.507
	Age categories(2)	1.470	.608	3.555
	Age categories(3)	3.124	1.376	7.091
	Age categories(4)	8.006	3.616	17.725
	Age categories(5)	18.524	8.355	41.072
	Constant	.011		

a. Variable(s) entered on step 1: Lifetimeabuseindex, Householddysf_parent, Sex, Residence type, Parent education, Immigrant status, Age categories.

Logistic Regression

Notes

Output Created		05-OCT-2024 11:47:42
Comments		
Input	Data	/Users/stevenlc/Library/CloudStorage/OneDrive-Privat/ICloud filer/Doktorander/Rickard/Artikel 3/Revision art 3/Artikel3_revision.sav
	Active Dataset	DataSet7
	Filter	<none>
	Weight	<none>
	Split File	<none>
	N of Rows in Working Data File	10337
Missing Value Handling	Definition of Missing	User-defined missing values are treated as missing
Syntax		LOGISTIC REGRESSION VARIABLES KOL /METHOD=ENTER Maltreatment_0to3 /CONTRAST (Maltreatment_0to3)=Indicator(1) /PRINT=CI(95) /CRITERIA=PIN(0.05) POUT(0.10) ITERATE(20) CUT(0.5).
Resources	Processor Time	00:00:00,39
	Elapsed Time	00:00:00,00

Case Processing Summary

Unweighted Cases[a]		N	Percent
Selected Cases	Included in Analysis	9169	88.7
	Missing Cases	1168	11.3
	Total	10337	100.0
Unselected Cases		0	.0
Total		10337	100.0

a. If weight is in effect, see classification table for the total number of cases.

Dependent Variable Encoding

Original Value	Internal Value
1.00	0
2.00	1

Categorical Variables Codings

		Frequency	Parameter coding (1)	(2)	(3)
Maltreatment_0to3	.00	6043	.000	.000	.000
	1.00	1589	1.000	.000	.000
	2.00	744	.000	1.000	.000
	3.00	793	.000	.000	1.000

Block 0: Beginning Block

Classification Table[a,b]

Observed			Predicted COPD 1.00	2.00	Percentage Correct
Step 0	COPD	1.00	9012	0	100.0
		2.00	157	0	.0
	Overall Percentage				98.3

a. Constant is included in the model.

b. The cut value is .500

Variables in the Equation

		B	S.E.	Wald	df	Sig.	Exp(B)
Step 0	Constant	-4.050	.081	2531.181	1	.000	.017

Variables not in the Equation

			Score	df	Sig.
Step 0	Variables	Maltreatment_0to3	28.719	3	<.001
		Maltreatment_0to3(1)	2.086	1	.149
		Maltreatment_0to3(2)	.444	1	.505
		Maltreatment_0to3(3)	22.119	1	<.001
	Overall Statistics		28.719	3	<.001

Block 1: Method = Enter

Omnibus Tests of Model Coefficients

		Chi-square	df	Sig.
Step 1	Step	24.023	3	<.001
	Block	24.023	3	<.001
	Model	24.023	3	<.001

Model Summary

Step	-2 Log likelihood	Cox & Snell R Square	Nagelkerke R Square
1	1564.417[a]	.003	.016

a. Estimation terminated at iteration number 7 because parameter estimates changed by less than .001.

Classification Table[a]

			Predicted		
			COPD		Percentage Correct
Observed			1.00	2.00	
Step 1	COPD	1.00	9012	0	100.0
		2.00	157	0	.0
	Overall Percentage				98.3

a. The cut value is .500

Variables in the Equation

		B	S.E.	Wald	df	Sig.
Step 1[a]	Maltreatment_0to3			26.741	3	<.001
	Maltreatment_0to3(1)	.514	.207	6.140	1	.013
	Maltreatment_0to3(2)	.453	.285	2.536	1	.111
	Maltreatment_0to3(3)	1.101	.218	25.444	1	<.001
	Constant	-4.337	.114	1448.179	1	.000

Variables in the Equation

		Exp(B)	95% C.I.for EXP(B) Lower	95% C.I.for EXP(B) Upper
Step 1[a]	Maltreatment_0to3			
	Maltreatment_0to3(1)	1.672	1.113	2.511
	Maltreatment_0to3(2)	1.574	.901	2.749
	Maltreatment_0to3(3)	3.007	1.960	4.612
	Constant	.013		

a. Variable(s) entered on step 1: Maltreatment_0to3.

Logistic Regression

Notes

Output Created		05-OCT-2024 11:47:42
Comments		
Input	Data	/Users/stevenlc/Library/CloudStorage/OneDrive-Privat/ICloud filer/Doktorander/Rickard/Artikel 3/Revision art 3/Artikel3_revision.sav
	Active Dataset	DataSet7
	Filter	<none>
	Weight	<none>
	Split File	<none>
	N of Rows in Working Data File	10337
Missing Value Handling	Definition of Missing	User-defined missing values are treated as missing
Syntax		LOGISTIC REGRESSION VARIABLES KOL /METHOD=ENTER Maltreatment_0to3 Householddysf_parent Kon barnboendeny utbildningmammapappa_ny fodelselandmammapappa_ny alderskategorier /CONTRAST (Maltreatment_0to3)=Indicator(1) /CONTRAST (Householddysf_parent)=Indicator(1) /CONTRAST (Kon)=Indicator(1) /CONTRAST (barnboendeny)=Indicator(1) /CONTRAST (utbildningmammapappa_ny)=Indicator(1) /CONTRAST (fodelselandmammapappa_ny)=Indicator(1) /CONTRAST (alderskategorier)=Indicator(1) /PRINT=CI(95) /CRITERIA=PIN(0.05) POUT(0.10) ITERATE(20) CUT(0.5).
Resources	Processor Time	00:00:00,47
	Elapsed Time	00:00:01,00

Case Processing Summary

Unweighted Cases[a]		N	Percent
Selected Cases	Included in Analysis	7629	73.8
	Missing Cases	2708	26.2
	Total	10337	100.0
Unselected Cases		0	.0
Total		10337	100.0

a. If weight is in effect, see classification table for the total number of cases.

Dependent Variable Encoding

Original Value	Internal Value
1.00	0
2.00	1

Categorical Variables Codings

		Frequency	Parameter coding (1)	(2)	(3)
Age categories	17-25	911	.000	.000	.000
	26-35	1194	1.000	.000	.000
	36-45	1430	.000	1.000	.000
	46-55	1512	.000	.000	1.000
	56-65	1592	.000	.000	.000
	66-74	990	.000	.000	.000
Maltreatment_0to3	.00	5160	.000	.000	.000
	1.00	1292	1.000	.000	.000
	2.00	590	.000	1.000	.000
	3.00	587	.000	.000	1.000
Parent education	At least one parent high school	4067	.000		
	Both parents below high school	3562	1.000		
Householddysf_parent	.00	5768	.000		
	1.00	1861	1.000		
Sex	Man	3417	.000		
	Kvinna	4212	1.000		
Immigrant status	At least one Nordic parent	7143	.000		
	Both parents born elsewhere	486	1.000		
Residence type	Owned home	5713	.000		
	Rental	1916	1.000		

Categorical Variables Codings

		Parameter coding (4)	Parameter coding (5)
Age categories	17-25	.000	.000
	26-35	.000	.000
	36-45	.000	.000
	46-55	.000	.000
	56-65	1.000	.000
	66-74	.000	1.000
Maltreatment_0to3	.00		
	1.00		
	2.00		
	3.00		
Parent education	At least one parent high school		
	Both parents below high school		
Householddysf_parent	.00		
	1.00		
Sex	Man		
	Kvinna		
Immigrant status	At least one Nordic parent		
	Both parents born elsewhere		
Residence type	Owned home		
	Rental		

Block 0: Beginning Block

Classification Table[a,b]

Observed			Predicted COPD 1.00	Predicted COPD 2.00	Percentage Correct
Step 0	COPD	1.00	7511	0	100.0
		2.00	118	0	.0
	Overall Percentage				98.5

a. Constant is included in the model.

b. The cut value is .500

Variables in the Equation

		B	S.E.	Wald	df	Sig.	Exp(B)
Step 0	Constant	-4.153	.093	2004.139	1	.000	.016

Variables not in the Equation

			Score	df	Sig.
Step 0	Variables	Maltreatment_0to3	22.325	3	<.001
		Maltreatment_0to3(1)	2.215	1	.137
		Maltreatment_0to3(2)	.092	1	.761
		Maltreatment_0to3(3)	17.222	1	<.001
		Householddysf_parent(1)	.668	1	.414
		Sex(1)	.001	1	.978
		Residence type(1)	17.163	1	<.001
		Parent education(1)	21.451	1	<.001
		Immigrant status(1)	.034	1	.854
		Age categories	117.323	5	<.001
		Age categories(1)	15.600	1	<.001
		Age categories(2)	16.561	1	<.001
		Age categories(3)	.309	1	.579
		Age categories(4)	7.984	1	.005
		Age categories(5)	86.501	1	<.001
	Overall Statistics		144.540	13	<.001

Block 1: Method = Enter

Omnibus Tests of Model Coefficients

		Chi-square	df	Sig.
Step 1	Step	129.915	13	<.001
	Block	129.915	13	<.001
	Model	129.915	13	<.001

Model Summary

Step	-2 Log likelihood	Cox & Snell R Square	Nagelkerke R Square
1	1088.141[a]	.017	.114

a. Estimation terminated at iteration number 9 because parameter estimates changed by less than .001.

Classification Table[a]

			Predicted		
			COPD		Percentage Correct
	Observed		1.00	2.00	
Step 1	COPD	1.00	7511	0	100.0
		2.00	118	0	.0
	Overall Percentage				98.5

a. The cut value is .500

Variables in the Equation

		B	S.E.	Wald	df	Sig.
Step 1[a]	Maltreatment_0to3			17.639	3	<.001
	Maltreatment_0to3(1)	.571	.242	5.598	1	.018
	Maltreatment_0to3(2)	.253	.351	.520	1	.471
	Maltreatment_0to3(3)	1.119	.280	16.029	1	<.001
	Householddysf_parent(1)	-.031	.253	.015	1	.903
	Sex(1)	.090	.190	.225	1	.635
	Residence type(1)	.332	.197	2.861	1	.091
	Parent education(1)	.053	.216	.061	1	.805
	Immigrant status(1)	.317	.383	.683	1	.408
	Age categories			66.641	5	<.001
	Age categories(1)	-.348	.819	.180	1	.671
	Age categories(2)	-.015	.735	.000	1	.984
	Age categories(3)	1.287	.630	4.177	1	.041
	Age categories(4)	1.809	.622	8.465	1	.004
	Age categories(5)	2.673	.622	18.477	1	<.001
	Constant	-6.073	.604	101.090	1	<.001

Variables in the Equation

		Exp(B)	95% C.I.for EXP(B)	
			Lower	Upper
Step 1[a]	Maltreatment_0to3			
	Maltreatment_0to3(1)	1.771	1.103	2.843
	Maltreatment_0to3(2)	1.288	.648	2.560
	Maltreatment_0to3(3)	3.063	1.771	5.298
	Householddysf_parent(1)	.970	.591	1.592
	Sex(1)	1.094	.754	1.588
	Residence type(1)	1.394	.949	2.050
	Parent education(1)	1.055	.691	1.610
	Immigrant status(1)	1.373	.648	2.908
	Age categories			
	Age categories(1)	.706	.142	3.518
	Age categories(2)	.985	.233	4.160
	Age categories(3)	3.621	1.054	12.437
	Age categories(4)	6.102	1.804	20.637
	Age categories(5)	14.482	4.281	48.992
	Constant	.002		

a. Variable(s) entered on step 1: Maltreatment_0to3, Householddysf_parent, Sex, Residence type, Parent education, Immigrant status, Age categories.

Logistic Regression

Notes

Output Created		05-OCT-2024 11:47:43
Comments		
Input	Data	/Users/stevenlc/Library/CloudStorage/OneDrive-Privat/ICloud filer/Doktorander/Rickard/Artikel 3/Revision art 3/Artikel3_revision.sav
	Active Dataset	DataSet7
	Filter	<none>
	Weight	<none>
	Split File	<none>
	N of Rows in Working Data File	10337
Missing Value Handling	Definition of Missing	User-defined missing values are treated as missing
Syntax		LOGISTIC REGRESSION VARIABLES KOL /METHOD=ENTER Lifetimeabuseindex /CONTRAST (Lifetimeabuseindex)=Indicator(1) /PRINT=CI(95) /CRITERIA=PIN(0.05) POUT(0.10) ITERATE(20) CUT(0.5).
Resources	Processor Time	00:00:00,41
	Elapsed Time	00:00:00,00

Case Processing Summary

Unweighted Cases[a]		N	Percent
Selected Cases	Included in Analysis	8951	86.6
	Missing Cases	1386	13.4
	Total	10337	100.0
Unselected Cases		0	.0
Total		10337	100.0

a. If weight is in effect, see classification table for the total number of cases.

Dependent Variable Encoding

Original Value	Internal Value
1.00	0
2.00	1

Categorical Variables Codings

		Frequency	Parameter coding (1)	(2)	(3)	(4)	(5)
Lifetimeabuseindex	.00	5038	.000	.000	.000	.000	.000
	1.00	1062	1.000	.000	.000	.000	.000
	2.00	454	.000	1.000	.000	.000	.000
	3.00	377	.000	.000	1.000	.000	.000
	4.00	875	.000	.000	.000	1.000	.000
	5.00	483	.000	.000	.000	.000	1.000
	6.00	269	.000	.000	.000	.000	.000
	7.00	393	.000	.000	.000	.000	.000

Categorical Variables Codings

		Parameter coding (6)	(7)
Lifetimeabuseindex	.00	.000	.000
	1.00	.000	.000
	2.00	.000	.000
	3.00	.000	.000
	4.00	.000	.000
	5.00	.000	.000
	6.00	1.000	.000
	7.00	.000	1.000

Block 0: Beginning Block

Classification Table[a,b]

Observed			Predicted COPD 1.00	2.00	Percentage Correct
Step 0	COPD	1.00	8794	0	100.0
		2.00	157	0	.0
	Overall Percentage				98.2

a. Constant is included in the model.

b. The cut value is .500

Variables in the Equation

		B	S.E.	Wald	df	Sig.	Exp(B)
Step 0	Constant	-4.026	.081	2499.604	1	.000	.018

Variables not in the Equation

			Score	df	Sig.
Step 0	Variables	Lifetimeabuseindex	36.454	7	<.001
		Lifetimeabuseindex(1)	1.790	1	.181
		Lifetimeabuseindex(2)	.519	1	.471
		Lifetimeabuseindex(3)	4.664	1	.031
		Lifetimeabuseindex(4)	3.968	1	.046
		Lifetimeabuseindex(5)	.297	1	.586
		Lifetimeabuseindex(6)	4.078	1	.043
		Lifetimeabuseindex(7)	19.052	1	<.001
	Overall Statistics		36.454	7	<.001

Block 1: Method = Enter

Omnibus Tests of Model Coefficients

		Chi-square	df	Sig.
Step 1	Step	30.165	7	<.001
	Block	30.165	7	<.001
	Model	30.165	7	<.001

Model Summary

Step	-2 Log likelihood	Cox & Snell R Square	Nagelkerke R Square
1	1550.654[a]	.003	.021

a. Estimation terminated at iteration number 7 because parameter estimates changed by less than .001.

Classification Table[a]

	Observed		Predicted		
			COPD		Percentage Correct
			1.00	2.00	
Step 1	COPD	1.00	8794	0	100.0
		2.00	157	0	.0
	Overall Percentage				98.2

a. The cut value is .500

Variables in the Equation

		B	S.E.	Wald	df	Sig.
Step 1[a]	Lifetimeabuseindex			33.275	7	<.001
	Lifetimeabuseindex(1)	.495	.239	4.295	1	.038
	Lifetimeabuseindex(2)	-.051	.428	.014	1	.906
	Lifetimeabuseindex(3)	.847	.317	7.139	1	.008
	Lifetimeabuseindex(4)	-.423	.375	1.274	1	.259
	Lifetimeabuseindex(5)	.406	.341	1.412	1	.235
	Lifetimeabuseindex(6)	.899	.360	6.241	1	.012
	Lifetimeabuseindex(7)	1.226	.270	20.663	1	<.001
	Constant	-4.262	.120	1254.021	1	<.001

Variables in the Equation

		Exp(B)	95% C.I.for EXP(B)	
			Lower	Upper
Step 1[a]	Lifetimeabuseindex			
	Lifetimeabuseindex(1)	1.641	1.027	2.621
	Lifetimeabuseindex(2)	.951	.411	2.200
	Lifetimeabuseindex(3)	2.333	1.253	4.344
	Lifetimeabuseindex(4)	.655	.314	1.366
	Lifetimeabuseindex(5)	1.500	.768	2.930
	Lifetimeabuseindex(6)	2.457	1.214	4.973
	Lifetimeabuseindex(7)	3.407	2.008	5.779
	Constant	.014		

a. Variable(s) entered on step 1: Lifetimeabuseindex.

Logistic Regression

Notes

Output Created		05-OCT-2024 11:47:43
Comments		
Input	Data	/Users/stevenlc/Library/CloudStorage/OneDrive-Privat/ICloud filer/Doktorander/Rickard/Artikel 3/Revision art 3/Artikel3_revision.sav
	Active Dataset	DataSet7
	Filter	<none>
	Weight	<none>
	Split File	<none>
	N of Rows in Working Data File	10337
Missing Value Handling	Definition of Missing	User-defined missing values are treated as missing
Syntax		LOGISTIC REGRESSION VARIABLES KOL<br>/METHOD=ENTER Lifetimeabuseindex Householddysf_parent Kon barnboendeny utbildningmammapappa_ny fodelselandmammapappa_ny alderskategorier<br>/CONTRAST (Lifetimeabuseindex)=Indicator(1)<br>/CONTRAST (Householddysf_parent)=Indicator(1)<br>/CONTRAST (Kon)=Indicator(1)<br>/CONTRAST (barnboendeny)=Indicator(1)<br>/CONTRAST (utbildningmammapappa_ny)=Indicator(1)<br>/CONTRAST (fodelselandmammapappa_ny)=Indicator(1)<br>/CONTRAST (alderskategorier)=Indicator(1)<br>/PRINT=CI(95)<br>/CRITERIA=PIN(0.05) POUT(0.10) ITERATE(20) CUT(0.5).
Resources	Processor Time	00:00:00,50
	Elapsed Time	00:00:01,00

Case Processing Summary

Unweighted Cases[a]		N	Percent
Selected Cases	Included in Analysis	7471	72.3
	Missing Cases	2866	27.7
	Total	10337	100.0
Unselected Cases		0	.0
Total		10337	100.0

a. If weight is in effect, see classification table for the total number of cases.

Dependent Variable Encoding

Original Value	Internal Value
1.00	0
2.00	1

Categorical Variables Codings

		Frequency	Parameter coding		
			(1)	(2)	(3)
Lifetimeabuseindex	.00	4321	.000	.000	.000
	1.00	865	1.000	.000	.000
	2.00	353	.000	1.000	.000
	3.00	273	.000	.000	1.000
	4.00	739	.000	.000	.000
	5.00	399	.000	.000	.000
	6.00	222	.000	.000	.000
	7.00	299	.000	.000	.000
Age categories	17-25	888	.000	.000	.000
	26-35	1171	1.000	.000	.000
	36-45	1403	.000	1.000	.000
	46-55	1480	.000	.000	1.000
	56-65	1561	.000	.000	.000
	66-74	968	.000	.000	.000
Parent education	At least one parent high school	3968	.000		
	Both parents below high school	3503	1.000		
Householddysf_parent	.00	5656	.000		
	1.00	1815	1.000		
Sex	Man	3341	.000		
	Kvinna	4130	1.000		
Immigrant status	At least one Nordic parent	6995	.000		
	Both parents born elsewhere	476	1.000		
Residence type	Owned home	5588	.000		
	Rental	1883	1.000		

Categorical Variables Codings

		Parameter coding (4)	(5)	(6)	(7)
Lifetimeabuseindex	.00	.000	.000	.000	.000
	1.00	.000	.000	.000	.000
	2.00	.000	.000	.000	.000
	3.00	.000	.000	.000	.000
	4.00	1.000	.000	.000	.000
	5.00	.000	1.000	.000	.000
	6.00	.000	.000	1.000	.000
	7.00	.000	.000	.000	1.000
Age categories	17-25	.000	.000		
	26-35	.000	.000		
	36-45	.000	.000		
	46-55	.000	.000		
	56-65	1.000	.000		
	66-74	.000	1.000		
Parent education	At least one parent high school				
	Both parents below high school				
Householddysf_parent	.00				
	1.00				
Sex	Man				
	Kvinna				
Immigrant status	At least one Nordic parent				
	Both parents born elsewhere				
Residence type	Owned home				
	Rental				

Block 0: Beginning Block

Classification Table[a,b]

Observed			Predicted COPD 1.00	2.00	Percentage Correct
Step 0	COPD	1.00	7353	0	100.0
		2.00	118	0	.0
	Overall Percentage				98.4

a. Constant is included in the model.

b. The cut value is .500

Variables in the Equation

		B	S.E.	Wald	df	Sig.	Exp(B)
Step 0	Constant	-4.132	.093	1983.015	1	.000	.016

Variables not in the Equation

			Score	df	Sig.
Step 0	Variables	Lifetimeabuseindex	27.968	7	<.001
		Lifetimeabuseindex(1)	2.396	1	.122
		Lifetimeabuseindex(2)	1.269	1	.260
		Lifetimeabuseindex(3)	7.913	1	.005
		Lifetimeabuseindex(4)	2.109	1	.146
		Lifetimeabuseindex(5)	.083	1	.773
		Lifetimeabuseindex(6)	3.645	1	.056
		Lifetimeabuseindex(7)	8.832	1	.003
		Householddysf_parent(1)	.629	1	.428
		Sex(1)	.002	1	.966
		Residence type(1)	16.942	1	<.001
		Parent education(1)	21.047	1	<.001
		Immigrant status(1)	.034	1	.855
		Age categories	117.546	5	<.001
		Age categories(1)	15.642	1	<.001
		Age categories(2)	16.623	1	<.001
		Age categories(3)	.306	1	.580
		Age categories(4)	7.939	1	.005
		Age categories(5)	86.765	1	<.001
	Overall Statistics		151.365	17	<.001

Block 1: Method = Enter

Omnibus Tests of Model Coefficients

		Chi-square	df	Sig.
Step 1	Step	136.548	17	<.001
	Block	136.548	17	<.001
	Model	136.548	17	<.001

Model Summary

Step	-2 Log likelihood	Cox & Snell R Square	Nagelkerke R Square
1	1076.530 [a]	.018	.121

a. Estimation terminated at iteration number 9 because parameter estimates changed by less than .001.

Classification Table[a]

Observed			Predicted COPD 1.00	Predicted COPD 2.00	Percentage Correct
Step 1	COPD	1.00	7353	0	100.0
		2.00	118	0	.0
	Overall Percentage				98.4

a. The cut value is .500

Variables in the Equation

		B	S.E.	Wald	df	Sig.
Step 1[a]	Lifetimeabuseindex			23.910	7	.001
	Lifetimeabuseindex(1)	.570	.274	4.317	1	.038
	Lifetimeabuseindex(2)	-.554	.601	.851	1	.356
	Lifetimeabuseindex(3)	.952	.368	6.690	1	.010
	Lifetimeabuseindex(4)	.018	.408	.002	1	.965
	Lifetimeabuseindex(5)	.600	.416	2.083	1	.149
	Lifetimeabuseindex(6)	1.007	.420	5.754	1	.016
	Lifetimeabuseindex(7)	1.347	.363	13.802	1	<.001
	Householddysf_parent(1)	-.033	.254	.017	1	.898
	Sex(1)	.062	.191	.104	1	.747
	Residence type(1)	.327	.197	2.755	1	.097
	Parent education(1)	.069	.216	.102	1	.749
	Immigrant status(1)	.335	.384	.762	1	.383
	Age categories			67.752	5	<.001
	Age categories(1)	-.365	.820	.198	1	.656
	Age categories(2)	-.032	.735	.002	1	.965
	Age categories(3)	1.261	.630	4.003	1	.045
	Age categories(4)	1.827	.622	8.634	1	.003
	Age categories(5)	2.692	.622	18.715	1	<.001
	Constant	-6.058	.607	99.646	1	<.001

Variables in the Equation

		Exp(B)	95% C.I.for EXP(B) Lower	Upper
Step 1[a]	Lifetimeabuseindex			
	Lifetimeabuseindex(1)	1.768	1.033	3.027
	Lifetimeabuseindex(2)	.575	.177	1.865
	Lifetimeabuseindex(3)	2.592	1.259	5.332
	Lifetimeabuseindex(4)	1.018	.458	2.265
	Lifetimeabuseindex(5)	1.822	.807	4.113
	Lifetimeabuseindex(6)	2.738	1.202	6.237
	Lifetimeabuseindex(7)	3.845	1.889	7.825
	Householddysf_parent(1)	.968	.589	1.592
	Sex(1)	1.064	.731	1.547
	Residence type(1)	1.386	.943	2.039
	Parent education(1)	1.072	.702	1.637
	Immigrant status(1)	1.398	.659	2.967
	Age categories			
	Age categories(1)	.694	.139	3.460
	Age categories(2)	.968	.229	4.092
	Age categories(3)	3.527	1.026	12.127
	Age categories(4)	6.216	1.837	21.028
	Age categories(5)	14.758	4.359	49.964
	Constant	.002		

a. Variable(s) entered on step 1: Lifetimeabuseindex, Householddysf_parent, Sex, Residence type, Parent education, Immigrant status, Age categories.

Logistic Regression

Notes

Output Created		05-OCT-2024 11:47:44
Comments		
Input	Data	/Users/stevenlc/Library/CloudStorage/OneDrive-Privat/ICloud filer/Doktorander/Rickard/Artikel 3/Revision art 3/Artikel3_revision.sav
	Active Dataset	DataSet7
	Filter	<none>
	Weight	<none>
	Split File	<none>
	N of Rows in Working Data File	10337
Missing Value Handling	Definition of Missing	User-defined missing values are treated as missing
Syntax		LOGISTIC REGRESSION VARIABLES Diabetes_II /METHOD=ENTER Maltreatment_0to3 /CONTRAST (Maltreatment_0to3)=Indicator(1) /PRINT=CI(95) /CRITERIA=PIN(0.05) POUT(0.10) ITERATE(20) CUT(0.5).
Resources	Processor Time	00:00:00,39
	Elapsed Time	00:00:00,00

Case Processing Summary

Unweighted Cases[a]		N	Percent
Selected Cases	Included in Analysis	9169	88.7
	Missing Cases	1168	11.3
	Total	10337	100.0
Unselected Cases		0	.0
Total		10337	100.0

a. If weight is in effect, see classification table for the total number of cases.

Dependent Variable Encoding

Original Value	Internal Value
1.00	0
2.00	1

Categorical Variables Codings

		Frequency	Parameter coding (1)	(2)	(3)
Maltreatment_0to3	.00	6043	.000	.000	.000
	1.00	1589	1.000	.000	.000
	2.00	744	.000	1.000	.000
	3.00	793	.000	.000	1.000

Block 0: Beginning Block

Classification Table[a,b]

Observed			Predicted Diabetes_II 1.00	2.00	Percentage Correct
Step 0	Diabetes_II	1.00	8843	0	100.0
		2.00	326	0	.0
	Overall Percentage				96.4

a. Constant is included in the model.

b. The cut value is .500

Variables in the Equation

		B	S.E.	Wald	df	Sig.	Exp(B)
Step 0	Constant	-3.300	.056	3424.921	1	.000	.037

Variables not in the Equation

			Score	df	Sig.
Step 0	Variables	Maltreatment_0to3	3.868	3	.276
		Maltreatment_0to3(1)	1.248	1	.264
		Maltreatment_0to3(2)	2.430	1	.119
		Maltreatment_0to3(3)	.583	1	.445
	Overall Statistics		3.868	3	.276

Block 1: Method = Enter

Omnibus Tests of Model Coefficients

		Chi-square	df	Sig.
Step 1	Step	3.715	3	.294
	Block	3.715	3	.294
	Model	3.715	3	.294

Model Summary

Step	-2 Log likelihood	Cox & Snell R Square	Nagelkerke R Square
1	2812.074[a]	.000	.002

a. Estimation terminated at iteration number 6 because parameter estimates changed by less than .001.

Classification Table[a]

Observed			Predicted Diabetes_II 1.00	Predicted Diabetes_II 2.00	Percentage Correct
Step 1	Diabetes_II	1.00	8843	0	100.0
		2.00	326	0	.0
	Overall Percentage				96.4

a. The cut value is .500

Variables in the Equation

		B	S.E.	Wald	df	Sig.
Step 1[a]	Maltreatment_0to3			3.844	3	.279
	Maltreatment_0to3(1)	-.128	.161	.635	1	.425
	Maltreatment_0to3(2)	.280	.189	2.200	1	.138
	Maltreatment_0to3(3)	.150	.194	.603	1	.437
	Constant	-3.319	.070	2243.516	1	.000

Variables in the Equation

		Exp(B)	95% C.I.for EXP(B) Lower	95% C.I.for EXP(B) Upper
Step 1[a]	Maltreatment_0to3			
	Maltreatment_0to3(1)	.879	.641	1.206
	Maltreatment_0to3(2)	1.324	.914	1.917
	Maltreatment_0to3(3)	1.162	.795	1.699
	Constant	.036		

a. Variable(s) entered on step 1: Maltreatment_0to3.

Logistic Regression

Notes

Output Created		05-OCT-2024 11:47:44
Comments		
Input	Data	/Users/stevenlc/Library/CloudStorage/OneDrive-Privat/ICloud filer/Doktorander/Rickard/Artikel 3/Revision art 3/Artikel3_revision.sav
	Active Dataset	DataSet7
	Filter	<none>
	Weight	<none>
	Split File	<none>
	N of Rows in Working Data File	10337
Missing Value Handling	Definition of Missing	User-defined missing values are treated as missing
Syntax		LOGISTIC REGRESSION VARIABLES Diabetes_II /METHOD=ENTER Maltreatment_0to3 Householddysf_parent Kon barnboendeny utbildningmammapappa_ny fodelselandmammapappa_ny alderskategorier /CONTRAST (Maltreatment_0to3)=Indicator(1) /CONTRAST (Householddysf_parent)=Indicator(1) /CONTRAST (Kon)=Indicator(1) /CONTRAST (barnboendeny)=Indicator(1) /CONTRAST (utbildningmammapappa_ny)=Indicator(1) /CONTRAST (fodelselandmammapappa_ny)=Indicator(1) /CONTRAST (alderskategorier)=Indicator(1) /PRINT=CI(95) /CRITERIA=PIN(0.05) POUT(0.10) ITERATE(20) CUT(0.5).
Resources	Processor Time	00:00:00,46
	Elapsed Time	00:00:01,00

Case Processing Summary

Unweighted Cases[a]		N	Percent
Selected Cases	Included in Analysis	7629	73.8
	Missing Cases	2708	26.2
	Total	10337	100.0
Unselected Cases		0	.0
Total		10337	100.0

a. If weight is in effect, see classification table for the total number of cases.

Dependent Variable Encoding

Original Value	Internal Value
1.00	0
2.00	1

Categorical Variables Codings

		Frequency	Parameter coding (1)	(2)	(3)
Age categories	17-25	911	.000	.000	.000
	26-35	1194	1.000	.000	.000
	36-45	1430	.000	1.000	.000
	46-55	1512	.000	.000	1.000
	56-65	1592	.000	.000	.000
	66-74	990	.000	.000	.000
Maltreatment_0to3	.00	5160	.000	.000	.000
	1.00	1292	1.000	.000	.000
	2.00	590	.000	1.000	.000
	3.00	587	.000	.000	1.000
Parent education	At least one parent high school	4067	.000		
	Both parents below high school	3562	1.000		
Householddysf_parent	.00	5768	.000		
	1.00	1861	1.000		
Sex	Man	3417	.000		
	Kvinna	4212	1.000		
Immigrant status	At least one Nordic parent	7143	.000		
	Both parents born elsewhere	486	1.000		
Residence type	Owned home	5713	.000		
	Rental	1916	1.000		

Categorical Variables Codings

		Parameter coding (4)	Parameter coding (5)
Age categories	17-25	.000	.000
	26-35	.000	.000
	36-45	.000	.000
	46-55	.000	.000
	56-65	1.000	.000
	66-74	.000	1.000
Maltreatment_0to3	.00		
	1.00		
	2.00		
	3.00		
Parent education	At least one parent high school		
	Both parents below high school		
Householddysf_parent	.00		
	1.00		
Sex	Man		
	Kvinna		
Immigrant status	At least one Nordic parent		
	Both parents born elsewhere		
Residence type	Owned home		
	Rental		

Block 0: Beginning Block

Classification Table[a,b]

Observed			Predicted Diabetes_II 1.00	Predicted Diabetes_II 2.00	Percentage Correct
Step 0	Diabetes_II	1.00	7370	0	100.0
		2.00	259	0	.0
	Overall Percentage				96.6

a. Constant is included in the model.

b. The cut value is .500

Variables in the Equation

		B	S.E.	Wald	df	Sig.	Exp(B)
Step 0	Constant	-3.348	.063	2805.175	1	.000	.035

Variables not in the Equation

			Score	df	Sig.
Step 0	Variables	Maltreatment_0to3	2.108	3	.550
		Maltreatment_0to3(1)	.233	1	.629
		Maltreatment_0to3(2)	1.996	1	.158
		Maltreatment_0to3(3)	.048	1	.826
		Householddysf_parent(1)	9.721	1	.002
		Sex(1)	32.721	1	<.001
		Residence type(1)	12.193	1	<.001
		Parent education(1)	83.407	1	<.001
		Immigrant status(1)	1.357	1	.244
		Age categories	269.882	5	<.001
		Age categories(1)	42.655	1	<.001
		Age categories(2)	33.159	1	<.001
		Age categories(3)	1.008	1	.315
		Age categories(4)	40.591	1	<.001
		Age categories(5)	165.533	1	<.001
	Overall Statistics		296.508	13	<.001

Block 1: Method = Enter

Omnibus Tests of Model Coefficients

		Chi-square	df	Sig.
Step 1	Step	301.882	13	<.001
	Block	301.882	13	<.001
	Model	301.882	13	<.001

Model Summary

Step	-2 Log likelihood	Cox & Snell R Square	Nagelkerke R Square
1	1959.558[a]	.039	.151

a. Estimation terminated at iteration number 9 because parameter estimates changed by less than .001.

Classification Table[a]

			Predicted		
			Diabetes_II		Percentage Correct
	Observed		1.00	2.00	
Step 1	Diabetes_II	1.00	7370	0	100.0
		2.00	259	0	.0
	Overall Percentage				96.6

a. The cut value is .500

Variables in the Equation

		B	S.E.	Wald	df	Sig.
Step 1[a]	Maltreatment_0to3			1.054	3	.788
	Maltreatment_0to3(1)	.001	.182	.000	1	.997
	Maltreatment_0to3(2)	.221	.223	.981	1	.322
	Maltreatment_0to3(3)	-.021	.261	.006	1	.936
	Householddysf_parent(1)	.034	.188	.032	1	.858
	Sex(1)	-.623	.133	21.908	1	<.001
	Residence type(1)	.094	.140	.455	1	.500
	Parent education(1)	.295	.155	3.623	1	.057
	Immigrant status(1)	.075	.311	.058	1	.809
	Age categories			113.661	5	<.001
	Age categories(1)	.782	1.156	.458	1	.499
	Age categories(2)	2.011	1.040	3.739	1	.053
	Age categories(3)	3.156	1.016	9.652	1	.002
	Age categories(4)	3.804	1.013	14.102	1	<.001
	Age categories(5)	4.367	1.014	18.539	1	<.001
	Constant	-6.563	1.006	42.594	1	<.001

Variables in the Equation

		Exp(B)	95% C.I.for EXP(B)	
			Lower	Upper
Step 1[a]	Maltreatment_0to3			
	Maltreatment_0to3(1)	1.001	.700	1.430
	Maltreatment_0to3(2)	1.247	.806	1.931
	Maltreatment_0to3(3)	.979	.587	1.634
	Householddysf_parent(1)	1.034	.715	1.495
	Sex(1)	.537	.413	.696
	Residence type(1)	1.099	.835	1.446
	Parent education(1)	1.343	.991	1.819
	Immigrant status(1)	1.078	.586	1.982
	Age categories			
	Age categories(1)	2.186	.227	21.070
	Age categories(2)	7.473	.973	57.392
	Age categories(3)	23.473	3.206	171.885
	Age categories(4)	44.892	6.164	326.950
	Age categories(5)	78.792	10.794	575.130
	Constant	.001		

a. Variable(s) entered on step 1: Maltreatment_0to3, Householddysf_parent, Sex, Residence type, Parent education, Immigrant status, Age categories.

Logistic Regression

Notes

Output Created		05-OCT-2024 11:47:45
Comments		
Input	Data	/Users/stevenlc/Library/CloudStorage/OneDrive-Privat/ICloud filer/Doktorander/Rickard/Artikel 3/Revision art 3/Artikel3_revision.sav
	Active Dataset	DataSet7
	Filter	<none>
	Weight	<none>
	Split File	<none>
	N of Rows in Working Data File	10337
Missing Value Handling	Definition of Missing	User-defined missing values are treated as missing
Syntax		LOGISTIC REGRESSION VARIABLES Diabetes_II /METHOD=ENTER Lifetimeabuseindex /CONTRAST (Lifetimeabuseindex)=Indicator(1) /PRINT=CI(95) /CRITERIA=PIN(0.05) POUT(0.10) ITERATE(20) CUT(0.5).
Resources	Processor Time	00:00:00,40
	Elapsed Time	00:00:00,00

Case Processing Summary

Unweighted Cases[a]		N	Percent
Selected Cases	Included in Analysis	8951	86.6
	Missing Cases	1386	13.4
	Total	10337	100.0
Unselected Cases		0	.0
Total		10337	100.0

a. If weight is in effect, see classification table for the total number of cases.

Dependent Variable Encoding

Original Value	Internal Value
1.00	0
2.00	1

Categorical Variables Codings

		Frequency	Parameter coding (1)	(2)	(3)	(4)	(5)
Lifetimeabuseindex	.00	5038	.000	.000	.000	.000	.000
	1.00	1062	1.000	.000	.000	.000	.000
	2.00	454	.000	1.000	.000	.000	.000
	3.00	377	.000	.000	1.000	.000	.000
	4.00	875	.000	.000	.000	1.000	.000
	5.00	483	.000	.000	.000	.000	1.000
	6.00	269	.000	.000	.000	.000	.000
	7.00	393	.000	.000	.000	.000	.000

Categorical Variables Codings

		Parameter coding (6)	(7)
Lifetimeabuseindex	.00	.000	.000
	1.00	.000	.000
	2.00	.000	.000
	3.00	.000	.000
	4.00	.000	.000
	5.00	.000	.000
	6.00	1.000	.000
	7.00	.000	1.000

Block 0: Beginning Block

Classification Table[a,b]

Observed			Predicted Diabetes_II 1.00	2.00	Percentage Correct
Step 0	Diabetes_II	1.00	8635	0	100.0
		2.00	316	0	.0
	Overall Percentage				96.5

a. Constant is included in the model.

b. The cut value is .500

Variables in the Equation

		B	S.E.	Wald	df	Sig.	Exp(B)
Step 0	Constant	-3.308	.057	3335.539	1	.000	.037

Variables not in the Equation

			Score	df	Sig.
Step 0	Variables	Lifetimeabuseindex	6.372	7	.497
		Lifetimeabuseindex(1)	.633	1	.426
		Lifetimeabuseindex(2)	.064	1	.800
		Lifetimeabuseindex(3)	.589	1	.443
		Lifetimeabuseindex(4)	1.290	1	.256
		Lifetimeabuseindex(5)	.598	1	.439
		Lifetimeabuseindex(6)	3.408	1	.065
		Lifetimeabuseindex(7)	.099	1	.753
	Overall Statistics		6.372	7	.497

Block 1: Method = Enter

Omnibus Tests of Model Coefficients

		Chi-square	df	Sig.
Step 1	Step	5.990	7	.541
	Block	5.990	7	.541
	Model	5.990	7	.541

Model Summary

Step	-2 Log likelihood	Cox & Snell R Square	Nagelkerke R Square
1	2727.988 [a]	.001	.003

a. Estimation terminated at iteration number 6 because parameter estimates changed by less than .001.

Classification Table[a]

			Predicted		
			Diabetes_II		Percentage Correct
Observed			1.00	2.00	
Step 1	Diabetes_II	1.00	8635	0	100.0
		2.00	316	0	.0
	Overall Percentage				96.5

a. The cut value is .500

Variables in the Equation

		B	S.E.	Wald	df	Sig.
Step 1[a]	Lifetimeabuseindex			6.275	7	.508
	Lifetimeabuseindex(1)	-.150	.192	.609	1	.435
	Lifetimeabuseindex(2)	.043	.259	.028	1	.868
	Lifetimeabuseindex(3)	.173	.266	.423	1	.515
	Lifetimeabuseindex(4)	-.237	.217	1.194	1	.274
	Lifetimeabuseindex(5)	-.222	.282	.621	1	.431
	Lifetimeabuseindex(6)	.460	.276	2.777	1	.096
	Lifetimeabuseindex(7)	.063	.274	.053	1	.819
	Constant	-3.290	.076	1888.407	1	.000

Variables in the Equation

			95% C.I.for EXP(B)	
		Exp(B)	Lower	Upper
Step 1[a]	Lifetimeabuseindex			
	Lifetimeabuseindex(1)	.861	.590	1.255
	Lifetimeabuseindex(2)	1.044	.629	1.733
	Lifetimeabuseindex(3)	1.189	.705	2.005
	Lifetimeabuseindex(4)	.789	.516	1.207
	Lifetimeabuseindex(5)	.801	.461	1.391
	Lifetimeabuseindex(6)	1.585	.922	2.723
	Lifetimeabuseindex(7)	1.065	.622	1.822
	Constant	.037		

a. Variable(s) entered on step 1: Lifetimeabuseindex.

Logistic Regression

Notes

Output Created		05-OCT-2024 11:47:45
Comments		
Input	Data	/Users/stevenlc/Library/CloudStorage/OneDrive-Privat/ICloud filer/Doktorander/Rickard/Artikel 3/Revision art 3/Artikel3_revision.sav
	Active Dataset	DataSet7
	Filter	<none>
	Weight	<none>
	Split File	<none>
	N of Rows in Working Data File	10337
Missing Value Handling	Definition of Missing	User-defined missing values are treated as missing
Syntax		LOGISTIC REGRESSION VARIABLES Diabetes_II<br>/METHOD=ENTER Lifetimeabuseindex Householddysf_parent Kon barnboendeny utbildningmammapappa_ny fodelselandmammapappa_ny alderskategorier<br>/CONTRAST (Lifetimeabuseindex)=Indicator(1)<br>/CONTRAST (Householddysf_parent)=Indicator(1)<br>/CONTRAST (Kon)=Indicator(1)<br>/CONTRAST (barnboendeny)=Indicator(1)<br>/CONTRAST (utbildningmammapappa_ny)=Indicator(1)<br>/CONTRAST (fodelselandmammapappa_ny)=Indicator(1)<br>/CONTRAST (alderskategorier)=Indicator(1)<br>/PRINT=CI(95)<br>/CRITERIA=PIN(0.05) POUT(0.10) ITERATE(20) CUT(0.5).
Resources	Processor Time	00:00:00,50
	Elapsed Time	00:00:00,00

Case Processing Summary

Unweighted Cases[a]		N	Percent
Selected Cases	Included in Analysis	7471	72.3
	Missing Cases	2866	27.7
	Total	10337	100.0
Unselected Cases		0	.0
Total		10337	100.0

a. If weight is in effect, see classification table for the total number of cases.

Dependent Variable Encoding

Original Value	Internal Value
1.00	0
2.00	1

Categorical Variables Codings

		Frequency	Parameter coding		
			(1)	(2)	(3)
Lifetimeabuseindex	.00	4321	.000	.000	.000
	1.00	865	1.000	.000	.000
	2.00	353	.000	1.000	.000
	3.00	273	.000	.000	1.000
	4.00	739	.000	.000	.000
	5.00	399	.000	.000	.000
	6.00	222	.000	.000	.000
	7.00	299	.000	.000	.000
Age categories	17-25	888	.000	.000	.000
	26-35	1171	1.000	.000	.000
	36-45	1403	.000	1.000	.000
	46-55	1480	.000	.000	1.000
	56-65	1561	.000	.000	.000
	66-74	968	.000	.000	.000
Parent education	At least one parent high school	3968	.000		
	Both parents below high school	3503	1.000		
Householddysf_parent	.00	5656	.000		
	1.00	1815	1.000		
Sex	Man	3341	.000		
	Kvinna	4130	1.000		
Immigrant status	At least one Nordic parent	6995	.000		
	Both parents born elsewhere	476	1.000		
Residence type	Owned home	5588	.000		
	Rental	1883	1.000		

Categorical Variables Codings

		Parameter coding			
		(4)	(5)	(6)	(7)
Lifetimeabuseindex	.00	.000	.000	.000	.000
	1.00	.000	.000	.000	.000
	2.00	.000	.000	.000	.000
	3.00	.000	.000	.000	.000
	4.00	1.000	.000	.000	.000
	5.00	.000	1.000	.000	.000
	6.00	.000	.000	1.000	.000
	7.00	.000	.000	.000	1.000
Age categories	17-25	.000	.000		
	26-35	.000	.000		
	36-45	.000	.000		
	46-55	.000	.000		
	56-65	1.000	.000		
	66-74	.000	1.000		
Parent education	At least one parent high school				
	Both parents below high school				
Householddysf_parent	.00				
	1.00				
Sex	Man				
	Kvinna				
Immigrant status	At least one Nordic parent				
	Both parents born elsewhere				
Residence type	Owned home				
	Rental				

Block 0: Beginning Block

Classification Table[a,b]

Observed			Predicted: Diabetes_II 1.00	Predicted: Diabetes_II 2.00	Percentage Correct
Step 0	Diabetes_II	1.00	7218	0	100.0
		2.00	253	0	.0
	Overall Percentage				96.6

a. Constant is included in the model.

b. The cut value is .500

Variables in the Equation

		B	S.E.	Wald	df	Sig.	Exp(B)
Step 0	Constant	-3.351	.064	2744.688	1	.000	.035

Variables not in the Equation

			Score	df	Sig.
Step 0	Variables	Lifetimeabuseindex	4.482	7	.723
		Lifetimeabuseindex(1)	.067	1	.796
		Lifetimeabuseindex(2)	.099	1	.753
		Lifetimeabuseindex(3)	.358	1	.550
		Lifetimeabuseindex(4)	.048	1	.826
		Lifetimeabuseindex(5)	.185	1	.667
		Lifetimeabuseindex(6)	2.851	1	.091
		Lifetimeabuseindex(7)	1.040	1	.308
		Householddysf_parent(1)	8.427	1	.004
		Sex(1)	31.835	1	<.001
		Residence type(1)	12.745	1	<.001
		Parent education(1)	81.359	1	<.001
		Immigrant status(1)	1.164	1	.281
		Age categories	269.345	5	<.001
		Age categories(1)	41.588	1	<.001
		Age categories(2)	33.825	1	<.001
		Age categories(3)	.964	1	.326
		Age categories(4)	37.914	1	<.001
		Age categories(5)	168.821	1	<.001
	Overall Statistics		299.475	17	<.001

Block 1: Method = Enter

Omnibus Tests of Model Coefficients

		Chi-square	df	Sig.
Step 1	Step	303.795	17	<.001
	Block	303.795	17	<.001
	Model	303.795	17	<.001

Model Summary

Step	-2 Log likelihood	Cox & Snell R Square	Nagelkerke R Square
1	1906.548 [a]	.040	.156

a. Estimation terminated at iteration number 9 because parameter estimates changed by less than .001.

Classification Table[a]

Observed			Predicted Diabetes_II 1.00	Predicted Diabetes_II 2.00	Percentage Correct
Step 1	Diabetes_II	1.00	7218	0	100.0
		2.00	253	0	.0
	Overall Percentage				96.6

a. The cut value is .500

Variables in the Equation

		B	S.E.	Wald	df	Sig.
Step 1[a]	Lifetimeabuseindex			7.263	7	.402
	Lifetimeabuseindex(1)	-.045	.216	.043	1	.835
	Lifetimeabuseindex(2)	-.082	.303	.073	1	.787
	Lifetimeabuseindex(3)	.006	.334	.000	1	.985
	Lifetimeabuseindex(4)	.313	.231	1.825	1	.177
	Lifetimeabuseindex(5)	.220	.316	.484	1	.487
	Lifetimeabuseindex(6)	.716	.322	4.939	1	.026
	Lifetimeabuseindex(7)	-.061	.407	.023	1	.880
	Householddysf_parent(1)	.072	.189	.145	1	.703
	Sex(1)	-.635	.135	22.073	1	<.001
	Residence type(1)	.100	.141	.497	1	.481
	Parent education(1)	.309	.157	3.844	1	.050
	Immigrant status(1)	.135	.312	.186	1	.667
	Age categories			116.720	5	<.001
	Age categories(1)	.761	1.156	.433	1	.511
	Age categories(2)	1.906	1.043	3.336	1	.068
	Age categories(3)	3.124	1.016	9.448	1	.002
	Age categories(4)	3.783	1.013	13.931	1	<.001
	Age categories(5)	4.386	1.015	18.684	1	<.001
	Constant	-6.602	1.006	43.022	1	<.001

Variables in the Equation

		Exp(B)	95% C.I.for EXP(B) Lower	Upper
Step 1[a]	Lifetimeabuseindex			
	Lifetimeabuseindex(1)	.956	.626	1.459
	Lifetimeabuseindex(2)	.921	.509	1.669
	Lifetimeabuseindex(3)	1.006	.523	1.937
	Lifetimeabuseindex(4)	1.367	.869	2.151
	Lifetimeabuseindex(5)	1.246	.671	2.313
	Lifetimeabuseindex(6)	2.047	1.088	3.850
	Lifetimeabuseindex(7)	.940	.424	2.088
	Householddysf_parent(1)	1.075	.742	1.556
	Sex(1)	.530	.406	.691
	Residence type(1)	1.105	.837	1.458
	Parent education(1)	1.361	1.000	1.854
	Immigrant status(1)	1.144	.620	2.109
	Age categories			
	Age categories(1)	2.140	.222	20.627
	Age categories(2)	6.726	.870	51.988
	Age categories(3)	22.734	3.102	166.636
	Age categories(4)	43.928	6.027	320.163
	Age categories(5)	80.291	10.991	586.557
	Constant	.001		

a. Variable(s) entered on step 1: Lifetimeabuseindex, Householddysf_parent, Sex, Residence type, Parent education, Immigrant status, Age categories.

Logistic Regression

Notes

Output Created		05-OCT-2024 11:47:45
Comments		
Input	Data	/Users/stevenlc/Library/CloudStorage/OneDrive-Privat/ICloud filer/Doktorander/Rickard/Artikel 3/Revision art 3/Artikel3_revision.sav
	Active Dataset	DataSet7
	Filter	<none>
	Weight	<none>
	Split File	<none>
	N of Rows in Working Data File	10337
Missing Value Handling	Definition of Missing	User-defined missing values are treated as missing
Syntax		LOGISTIC REGRESSION VARIABLES Tumörsjd /METHOD=ENTER Maltreatment_0to3 /CONTRAST (Maltreatment_0to3)=Indicator(1) /PRINT=CI(95) /CRITERIA=PIN(0.05) POUT(0.10) ITERATE(20) CUT(0.5).
Resources	Processor Time	00:00:00,39
	Elapsed Time	00:00:01,00

Case Processing Summary

Unweighted Cases[a]		N	Percent
Selected Cases	Included in Analysis	9169	88.7
	Missing Cases	1168	11.3
	Total	10337	100.0
Unselected Cases		0	.0
Total		10337	100.0

a. If weight is in effect, see classification table for the total number of cases.

Dependent Variable Encoding

Original Value	Internal Value
1.00	0
2.00	1

Categorical Variables Codings

		Frequency	Parameter coding (1)	(2)	(3)
Maltreatment_0to3	.00	6043	.000	.000	.000
	1.00	1589	1.000	.000	.000
	2.00	744	.000	1.000	.000
	3.00	793	.000	.000	1.000

Block 0: Beginning Block

Classification Table[a,b]

Observed			Predicted Cancer 1.00	2.00	Percentage Correct
Step 0	Cancer	1.00	8778	0	100.0
		2.00	391	0	.0
	Overall Percentage				95.7

a. Constant is included in the model.

b. The cut value is .500

Variables in the Equation

		B	S.E.	Wald	df	Sig.	Exp(B)
Step 0	Constant	-3.111	.052	3623.540	1	.000	.045

Variables not in the Equation

			Score	df	Sig.
Step 0	Variables	Maltreatment_0to3	1.391	3	.708
		Maltreatment_0to3(1)	.142	1	.706
		Maltreatment_0to3(2)	.654	1	.419
		Maltreatment_0to3(3)	.592	1	.442
	Overall Statistics		1.391	3	.708

Block 1: Method = Enter

Omnibus Tests of Model Coefficients

		Chi-square	df	Sig.
Step 1	Step	1.349	3	.718
	Block	1.349	3	.718
	Model	1.349	3	.718

Model Summary

Step	-2 Log likelihood	Cox & Snell R Square	Nagelkerke R Square
1	3230.848[a]	.000	.000

a. Estimation terminated at iteration number 6 because parameter estimates changed by less than .001.

Classification Table[a]

Observed			Predicted: Cancer 1.00	Predicted: Cancer 2.00	Predicted: Percentage Correct
Step 1	Cancer	1.00	8778	0	100.0
		2.00	391	0	.0
	Overall Percentage				95.7

a. The cut value is .500

Variables in the Equation

		B	S.E.	Wald	df	Sig.
Step 1[a]	Maltreatment_0to3			1.388	3	.708
	Maltreatment_0to3(1)	-.020	.142	.020	1	.888
	Maltreatment_0to3(2)	.156	.183	.727	1	.394
	Maltreatment_0to3(3)	.145	.178	.666	1	.414
	Constant	-3.135	.064	2372.873	1	.000

Variables in the Equation

		Exp(B)	95% C.I.for EXP(B) Lower	Upper
Step 1[a]	Maltreatment_0to3			
	Maltreatment_0to3(1)	.980	.742	1.295
	Maltreatment_0to3(2)	1.168	.817	1.671
	Maltreatment_0to3(3)	1.157	.816	1.640
	Constant	.044		

a. Variable(s) entered on step 1: Maltreatment_0to3.

Logistic Regression

Notes

Output Created		05-OCT-2024 11:47:46
Comments		
Input	Data	/Users/stevenlc/Library/CloudStorage/OneDrive-Privat/ICloud filer/Doktorander/Rickard/Artikel 3/Revision art 3/Artikel3_revision.sav
	Active Dataset	DataSet7
	Filter	<none>
	Weight	<none>
	Split File	<none>
	N of Rows in Working Data File	10337
Missing Value Handling	Definition of Missing	User-defined missing values are treated as missing
Syntax		LOGISTIC REGRESSION VARIABLES Tumörsjd /METHOD=ENTER Maltreatment_0to3 Householddysf_parent Kon barnboendeny utbildningmammapappa_ny fodelselandmammapappa_ny alderskategorier /CONTRAST (Maltreatment_0to3)=Indicator(1) /CONTRAST (Householddysf_parent)=Indicator(1) /CONTRAST (Kon)=Indicator(1) /CONTRAST (barnboendeny)=Indicator(1) /CONTRAST (utbildningmammapappa_ny)=Indicator(1) /CONTRAST (fodelselandmammapappa_ny)=Indicator(1) /CONTRAST (alderskategorier)=Indicator(1) /PRINT=CI(95) /CRITERIA=PIN(0.05) POUT(0.10) ITERATE(20) CUT(0.5).
Resources	Processor Time	00:00:00,46
	Elapsed Time	00:00:00,00

Case Processing Summary

Unweighted Cases[a]		N	Percent
Selected Cases	Included in Analysis	7629	73.8
	Missing Cases	2708	26.2
	Total	10337	100.0
Unselected Cases		0	.0
Total		10337	100.0

a. If weight is in effect, see classification table for the total number of cases.

Dependent Variable Encoding

Original Value	Internal Value
1.00	0
2.00	1

Categorical Variables Codings

		Frequency	Parameter coding		
			(1)	(2)	(3)
Age categories	17-25	911	.000	.000	.000
	26-35	1194	1.000	.000	.000
	36-45	1430	.000	1.000	.000
	46-55	1512	.000	.000	1.000
	56-65	1592	.000	.000	.000
	66-74	990	.000	.000	.000
Maltreatment_0to3	.00	5160	.000	.000	.000
	1.00	1292	1.000	.000	.000
	2.00	590	.000	1.000	.000
	3.00	587	.000	.000	1.000
Parent education	At least one parent high school	4067	.000		
	Both parents below high school	3562	1.000		
Householddysf_parent	.00	5768	.000		
	1.00	1861	1.000		
Sex	Man	3417	.000		
	Kvinna	4212	1.000		
Immigrant status	At least one Nordic parent	7143	.000		
	Both parents born elsewhere	486	1.000		
Residence type	Owned home	5713	.000		
	Rental	1916	1.000		

Categorical Variables Codings

		Parameter coding (4)	Parameter coding (5)
Age categories	17-25	.000	.000
	26-35	.000	.000
	36-45	.000	.000
	46-55	.000	.000
	56-65	1.000	.000
	66-74	.000	1.000
Maltreatment_0to3	.00		
	1.00		
	2.00		
	3.00		
Parent education	At least one parent high school		
	Both parents below high school		
Householddysf_parent	.00		
	1.00		
Sex	Man		
	Kvinna		
Immigrant status	At least one Nordic parent		
	Both parents born elsewhere		
Residence type	Owned home		
	Rental		

Block 0: Beginning Block

Classification Table[a,b]

Observed			Predicted Cancer 1.00	Predicted Cancer 2.00	Percentage Correct
Step 0	Cancer	1.00	7301	0	100.0
		2.00	328	0	.0
	Overall Percentage				95.7

a. Constant is included in the model.

b. The cut value is .500

Variables in the Equation

		B	S.E.	Wald	df	Sig.	Exp(B)
Step 0	Constant	-3.103	.056	3021.920	1	.000	.045

Variables not in the Equation

			Score	df	Sig.
Step 0	Variables	Maltreatment_0to3	2.621	3	.454
		Maltreatment_0to3(1)	.697	1	.404
		Maltreatment_0to3(2)	.119	1	.730
		Maltreatment_0to3(3)	2.051	1	.152
		Householddysf_parent(1)	5.604	1	.018
		Sex(1)	.197	1	.657
		Residence type(1)	21.496	1	<.001
		Parent education(1)	50.567	1	<.001
		Immigrant status(1)	.810	1	.368
		Age categories	204.538	5	<.001
		Age categories(1)	37.337	1	<.001
		Age categories(2)	16.967	1	<.001
		Age categories(3)	.322	1	.571
		Age categories(4)	38.296	1	<.001
		Age categories(5)	109.972	1	<.001
	Overall Statistics		212.361	13	<.001

Block 1: Method = Enter

Omnibus Tests of Model Coefficients

		Chi-square	df	Sig.
Step 1	Step	217.248	13	<.001
	Block	217.248	13	<.001
	Model	217.248	13	<.001

Model Summary

Step	-2 Log likelihood	Cox & Snell R Square	Nagelkerke R Square
1	2488.678[a]	.028	.094

a. Estimation terminated at iteration number 8 because parameter estimates changed by less than .001.

Classification Table[a]

			Predicted		
			Cancer		Percentage Correct
	Observed		1.00	2.00	
Step 1	Cancer	1.00	7301	0	100.0
		2.00	328	0	.0
	Overall Percentage				95.7

a. The cut value is .500

Variables in the Equation

		B	S.E.	Wald	df	Sig.
Step 1[a]	Maltreatment_0to3			1.502	3	.682
	Maltreatment_0to3(1)	-.096	.164	.345	1	.557
	Maltreatment_0to3(2)	-.033	.214	.024	1	.876
	Maltreatment_0to3(3)	.193	.208	.859	1	.354
	Householddysf_parent(1)	.001	.159	.000	1	.993
	Sex(1)	.157	.116	1.827	1	.176
	Residence type(1)	.226	.124	3.312	1	.069
	Parent education(1)	.050	.130	.146	1	.702
	Immigrant status(1)	.061	.262	.054	1	.816
	Age categories			116.088	5	<.001
	Age categories(1)	.828	.580	2.042	1	.153
	Age categories(2)	1.665	.533	9.767	1	.002
	Age categories(3)	2.208	.523	17.842	1	<.001
	Age categories(4)	2.780	.520	28.611	1	<.001
	Age categories(5)	3.237	.523	38.314	1	<.001
	Constant	-5.566	.511	118.693	1	<.001

Variables in the Equation

		Exp(B)	95% C.I.for EXP(B)	
			Lower	Upper
Step 1[a]	Maltreatment_0to3			
	Maltreatment_0to3(1)	.908	.659	1.252
	Maltreatment_0to3(2)	.967	.636	1.472
	Maltreatment_0to3(3)	1.213	.806	1.824
	Householddysf_parent(1)	1.001	.733	1.368
	Sex(1)	1.170	.932	1.468
	Residence type(1)	1.253	.983	1.598
	Parent education(1)	1.051	.814	1.357
	Immigrant status(1)	1.063	.636	1.775
	Age categories			
	Age categories(1)	2.289	.735	7.128
	Age categories(2)	5.286	1.861	15.020
	Age categories(3)	9.100	3.266	25.355
	Age categories(4)	16.124	5.821	44.659
	Age categories(5)	25.448	9.132	70.916
	Constant	.004		

a. Variable(s) entered on step 1: Maltreatment_0to3, Householddysf_parent, Sex, Residence type, Parent education, Immigrant status, Age categories.

Logistic Regression

Notes

Output Created		05-OCT-2024 11:47:46
Comments		
Input	Data	/Users/stevenlc/Library/CloudStorage/OneDrive-Privat/ICloud filer/Doktorander/Rickard/Artikel 3/Revision art 3/Artikel3_revision.sav
	Active Dataset	DataSet7
	Filter	<none>
	Weight	<none>
	Split File	<none>
	N of Rows in Working Data File	10337
Missing Value Handling	Definition of Missing	User-defined missing values are treated as missing
Syntax		LOGISTIC REGRESSION VARIABLES Tumörsjd /METHOD=ENTER Lifetimeabuseindex /CONTRAST (Lifetimeabuseindex)=Indicator(1) /PRINT=CI(95) /CRITERIA=PIN(0.05) POUT(0.10) ITERATE(20) CUT(0.5).
Resources	Processor Time	00:00:00,41
	Elapsed Time	00:00:01,00

Case Processing Summary

Unweighted Cases[a]		N	Percent
Selected Cases	Included in Analysis	8951	86.6
	Missing Cases	1386	13.4
	Total	10337	100.0
Unselected Cases		0	.0
Total		10337	100.0

a. If weight is in effect, see classification table for the total number of cases.

Dependent Variable Encoding

Original Value	Internal Value
1.00	0
2.00	1

Categorical Variables Codings

		Frequency	Parameter coding (1)	(2)	(3)	(4)	(5)
Lifetimeabuseindex	.00	5038	.000	.000	.000	.000	.000
	1.00	1062	1.000	.000	.000	.000	.000
	2.00	454	.000	1.000	.000	.000	.000
	3.00	377	.000	.000	1.000	.000	.000
	4.00	875	.000	.000	.000	1.000	.000
	5.00	483	.000	.000	.000	.000	1.000
	6.00	269	.000	.000	.000	.000	.000
	7.00	393	.000	.000	.000	.000	.000

Categorical Variables Codings

		Parameter coding (6)	(7)
Lifetimeabuseindex	.00	.000	.000
	1.00	.000	.000
	2.00	.000	.000
	3.00	.000	.000
	4.00	.000	.000
	5.00	.000	.000
	6.00	1.000	.000
	7.00	.000	1.000

Block 0: Beginning Block

Classification Table[a,b]

Observed			Predicted Cancer 1.00	Cancer 2.00	Percentage Correct
Step 0	Cancer	1.00	8563	0	100.0
		2.00	388	0	.0
	Overall Percentage				95.7

a. Constant is included in the model.

b. The cut value is .500

Variables in the Equation

		B	S.E.	Wald	df	Sig.	Exp(B)
Step 0	Constant	-3.094	.052	3553.719	1	.000	.045

Variables not in the Equation

			Score	df	Sig.
Step 0	Variables	Lifetimeabuseindex	4.171	7	.760
		Lifetimeabuseindex(1)	.024	1	.877
		Lifetimeabuseindex(2)	.098	1	.755
		Lifetimeabuseindex(3)	.120	1	.729
		Lifetimeabuseindex(4)	.262	1	.609
		Lifetimeabuseindex(5)	.455	1	.500
		Lifetimeabuseindex(6)	1.031	1	.310
		Lifetimeabuseindex(7)	2.283	1	.131
	Overall Statistics		4.171	7	.760

Block 1: Method = Enter

Omnibus Tests of Model Coefficients

		Chi-square	df	Sig.
Step 1	Step	3.911	7	.790
	Block	3.911	7	.790
	Model	3.911	7	.790

Model Summary

Step	-2 Log likelihood	Cox & Snell R Square	Nagelkerke R Square
1	3190.509 [a]	.000	.001

a. Estimation terminated at iteration number 6 because parameter estimates changed by less than .001.

Classification Table[a]

			Predicted		
			Cancer		Percentage Correct
Observed			1.00	2.00	
Step 1	Cancer	1.00	8563	0	100.0
		2.00	388	0	.0
	Overall Percentage				95.7

a. The cut value is .500

Variables in the Equation

		B	S.E.	Wald	df	Sig.
Step 1[a]	Lifetimeabuseindex			4.137	7	.764
	Lifetimeabuseindex(1)	.043	.165	.068	1	.795
	Lifetimeabuseindex(2)	.089	.234	.145	1	.703
	Lifetimeabuseindex(3)	-.068	.273	.063	1	.802
	Lifetimeabuseindex(4)	-.063	.186	.113	1	.736
	Lifetimeabuseindex(5)	-.136	.250	.297	1	.586
	Lifetimeabuseindex(6)	.286	.275	1.084	1	.298
	Lifetimeabuseindex(7)	.337	.226	2.229	1	.135
	Constant	-3.115	.070	1988.775	1	.000

Variables in the Equation

		Exp(B)	95% C.I.for EXP(B)	
			Lower	Upper
Step 1[a]	Lifetimeabuseindex			
	Lifetimeabuseindex(1)	1.044	.756	1.442
	Lifetimeabuseindex(2)	1.093	.691	1.730
	Lifetimeabuseindex(3)	.934	.547	1.594
	Lifetimeabuseindex(4)	.939	.652	1.353
	Lifetimeabuseindex(5)	.873	.534	1.425
	Lifetimeabuseindex(6)	1.331	.777	2.281
	Lifetimeabuseindex(7)	1.401	.900	2.182
	Constant	.044		

a. Variable(s) entered on step 1: Lifetimeabuseindex.

Logistic Regression

Notes

Output Created		05-OCT-2024 11:47:47
Comments		
Input	Data	/Users/stevenlc/Library/CloudStorage/OneDrive-Privat/ICloud filer/Doktorander/Rickard/Artikel 3/Revision art 3/Artikel3_revision.sav
	Active Dataset	DataSet7
	Filter	<none>
	Weight	<none>
	Split File	<none>
	N of Rows in Working Data File	10337
Missing Value Handling	Definition of Missing	User-defined missing values are treated as missing
Syntax		LOGISTIC REGRESSION VARIABLES Tumörsjd /METHOD=ENTER Lifetimeabuseindex Householddysf_parent Kon barnboendeny utbildningmammapappa_ny fodelselandmammapappa_ny alderskategorier /CONTRAST (Lifetimeabuseindex)=Indicator(1) /CONTRAST (Householddysf_parent)=Indicator(1) /CONTRAST (Kon)=Indicator(1) /CONTRAST (barnboendeny)=Indicator(1) /CONTRAST (utbildningmammapappa_ny)=Indicator(1) /CONTRAST (fodelselandmammapappa_ny)=Indicator(1) /CONTRAST (alderskategorier)=Indicator(1) /PRINT=CI(95) /CRITERIA=PIN(0.05) POUT(0.10) ITERATE(20) CUT(0.5).
Resources	Processor Time	00:00:00,49
	Elapsed Time	00:00:00,00

Case Processing Summary

Unweighted Cases[a]		N	Percent
Selected Cases	Included in Analysis	7471	72.3
	Missing Cases	2866	27.7
	Total	10337	100.0
Unselected Cases		0	.0
Total		10337	100.0

a. If weight is in effect, see classification table for the total number of cases.

Dependent Variable Encoding

Original Value	Internal Value
1.00	0
2.00	1

Categorical Variables Codings

			Parameter coding		
		Frequency	(1)	(2)	(3)
Lifetimeabuseindex	.00	4321	.000	.000	.000
	1.00	865	1.000	.000	.000
	2.00	353	.000	1.000	.000
	3.00	273	.000	.000	1.000
	4.00	739	.000	.000	.000
	5.00	399	.000	.000	.000
	6.00	222	.000	.000	.000
	7.00	299	.000	.000	.000
Age categories	17-25	888	.000	.000	.000
	26-35	1171	1.000	.000	.000
	36-45	1403	.000	1.000	.000
	46-55	1480	.000	.000	1.000
	56-65	1561	.000	.000	.000
	66-74	968	.000	.000	.000
Parent education	At least one parent high school	3968	.000		
	Both parents below high school	3503	1.000		
Householddysf_parent	.00	5656	.000		
	1.00	1815	1.000		
Sex	Man	3341	.000		
	Kvinna	4130	1.000		
Immigrant status	At least one Nordic parent	6995	.000		
	Both parents born elsewhere	476	1.000		
Residence type	Owned home	5588	.000		
	Rental	1883	1.000		

Categorical Variables Codings

		Parameter coding (4)	(5)	(6)	(7)
Lifetimeabuseindex	.00	.000	.000	.000	.000
	1.00	.000	.000	.000	.000
	2.00	.000	.000	.000	.000
	3.00	.000	.000	.000	.000
	4.00	1.000	.000	.000	.000
	5.00	.000	1.000	.000	.000
	6.00	.000	.000	1.000	.000
	7.00	.000	.000	.000	1.000
Age categories	17-25	.000	.000		
	26-35	.000	.000		
	36-45	.000	.000		
	46-55	.000	.000		
	56-65	1.000	.000		
	66-74	.000	1.000		
Parent education	At least one parent high school				
	Both parents below high school				
Householddysf_parent	.00				
	1.00				
Sex	Man				
	Kvinna				
Immigrant status	At least one Nordic parent				
	Both parents born elsewhere				
Residence type	Owned home				
	Rental				

Block 0: Beginning Block

Classification Table[a,b]

Observed			Predicted Cancer 1.00	Cancer 2.00	Percentage Correct
Step 0	Cancer	1.00	7145	0	100.0
		2.00	326	0	.0
	Overall Percentage				95.6

a. Constant is included in the model.

b. The cut value is .500

Variables in the Equation

		B	S.E.	Wald	df	Sig.	Exp(B)
Step 0	Constant	-3.087	.057	2971.601	1	.000	.046

Variables not in the Equation

			Score	df	Sig.
Step 0	Variables	Lifetimeabuseindex	6.400	7	.494
		Lifetimeabuseindex(1)	.002	1	.964
		Lifetimeabuseindex(2)	.012	1	.914
		Lifetimeabuseindex(3)	.001	1	.979
		Lifetimeabuseindex(4)	.182	1	.670
		Lifetimeabuseindex(5)	1.857	1	.173
		Lifetimeabuseindex(6)	.595	1	.440
		Lifetimeabuseindex(7)	4.036	1	.045
		Householddysf_parent(1)	5.158	1	.023
		Sex(1)	.186	1	.666
		Residence type(1)	21.848	1	<.001
		Parent education(1)	49.742	1	<.001
		Immigrant status(1)	.764	1	.382
		Age categories	201.663	5	<.001
		Age categories(1)	37.094	1	<.001
		Age categories(2)	16.747	1	<.001
		Age categories(3)	.259	1	.611
		Age categories(4)	37.373	1	<.001
		Age categories(5)	108.481	1	<.001
	Overall Statistics		215.782	17	<.001

Block 1: Method = Enter

Omnibus Tests of Model Coefficients

		Chi-square	df	Sig.
Step 1	Step	220.608	17	<.001
	Block	220.608	17	<.001
	Model	220.608	17	<.001

Model Summary

Step	-2 Log likelihood	Cox & Snell R Square	Nagelkerke R Square
1	2458.945[a]	.029	.097

a. Estimation terminated at iteration number 8 because parameter estimates changed by less than .001.

Classification Table[a]

Observed			Predicted: Cancer 1.00	Predicted: Cancer 2.00	Percentage Correct
Step 1	Cancer	1.00	7145	0	100.0
		2.00	326	0	.0
	Overall Percentage				95.6

a. The cut value is .500

Variables in the Equation

		B	S.E.	Wald	df	Sig.
Step 1[a]	Lifetimeabuseindex			7.797	7	.351
	Lifetimeabuseindex(1)	-.007	.186	.001	1	.970
	Lifetimeabuseindex(2)	-.173	.280	.383	1	.536
	Lifetimeabuseindex(3)	-.160	.315	.258	1	.612
	Lifetimeabuseindex(4)	.135	.205	.432	1	.511
	Lifetimeabuseindex(5)	-.234	.309	.574	1	.448
	Lifetimeabuseindex(6)	.250	.314	.635	1	.426
	Lifetimeabuseindex(7)	.570	.259	4.838	1	.028
	Householddysf_parent(1)	.003	.160	.000	1	.985
	Sex(1)	.145	.117	1.542	1	.214
	Residence type(1)	.232	.124	3.467	1	.063
	Parent education(1)	.058	.131	.199	1	.656
	Immigrant status(1)	.081	.262	.095	1	.758
	Age categories			116.550	5	<.001
	Age categories(1)	.814	.580	1.972	1	.160
	Age categories(2)	1.649	.533	9.566	1	.002
	Age categories(3)	2.187	.523	17.484	1	<.001
	Age categories(4)	2.774	.520	28.443	1	<.001
	Age categories(5)	3.240	.523	38.335	1	<.001
	Constant	-5.568	.512	118.301	1	<.001

Variables in the Equation

		Exp(B)	95% C.I.for EXP(B) Lower	Upper
Step 1[a]	Lifetimeabuseindex			
	Lifetimeabuseindex(1)	.993	.690	1.429
	Lifetimeabuseindex(2)	.841	.486	1.455
	Lifetimeabuseindex(3)	.852	.460	1.579
	Lifetimeabuseindex(4)	1.144	.765	1.711
	Lifetimeabuseindex(5)	.791	.432	1.449
	Lifetimeabuseindex(6)	1.285	.694	2.378
	Lifetimeabuseindex(7)	1.769	1.064	2.941
	Householddysf_parent(1)	1.003	.733	1.372
	Sex(1)	1.156	.919	1.454
	Residence type(1)	1.261	.988	1.608
	Parent education(1)	1.060	.820	1.371
	Immigrant status(1)	1.084	.649	1.813
	Age categories			
	Age categories(1)	2.257	.725	7.030
	Age categories(2)	5.200	1.829	14.781
	Age categories(3)	8.910	3.196	24.839
	Age categories(4)	16.016	5.779	44.383
	Age categories(5)	25.533	9.155	71.208
	Constant	.004		

a. Variable(s) entered on step 1: Lifetimeabuseindex, Householddysf_parent, Sex, Residence type, Parent education, Immigrant status, Age categories.

Logistic Regression

Notes

Output Created		05-OCT-2024 11:47:47
Comments		
Input	Data	/Users/stevenlc/Library/CloudStorage/OneDrive-Privat/ICloud filer/Doktorander/Rickard/Artikel 3/Revision art 3/Artikel3_revision.sav
	Active Dataset	DataSet7
	Filter	<none>
	Weight	<none>
	Split File	<none>
	N of Rows in Working Data File	10337
Missing Value Handling	Definition of Missing	User-defined missing values are treated as missing
Syntax		LOGISTIC REGRESSION VARIABLES Fetma /METHOD=ENTER Maltreatment_0to3 /CONTRAST (Maltreatment_0to3)=Indicator(1) /PRINT=CI(95) /CRITERIA=PIN(0.05) POUT(0.10) ITERATE(20) CUT(0.5).
Resources	Processor Time	00:00:00,38
	Elapsed Time	00:00:01,00

Case Processing Summary

Unweighted Cases[a]		N	Percent
Selected Cases	Included in Analysis	9005	87.1
	Missing Cases	1332	12.9
	Total	10337	100.0
Unselected Cases		0	.0
Total		10337	100.0

a. If weight is in effect, see classification table for the total number of cases.

Dependent Variable Encoding

Original Value	Internal Value
1.00	0
2.00	1

Categorical Variables Codings

		Frequency	Parameter coding (1)	(2)	(3)
Maltreatment_0to3	.00	5940	.000	.000	.000
	1.00	1568	1.000	.000	.000
	2.00	726	.000	1.000	.000
	3.00	771	.000	.000	1.000

Block 0: Beginning Block

Classification Table[a,b]

	Observed		Predicted Obesitas 1.00	2.00	Percentage Correct
Step 0	Obesitas	1.00	7806	0	100.0
		2.00	1199	0	.0
	Overall Percentage				86.7

a. Constant is included in the model.

b. The cut value is .500

Variables in the Equation

		B	S.E.	Wald	df	Sig.	Exp(B)
Step 0	Constant	-1.873	.031	3647.768	1	.000	.154

Variables not in the Equation

			Score	df	Sig.
Step 0	Variables	Maltreatment_0to3	21.776	3	<.001
		Maltreatment_0to3(1)	.000	1	.985
		Maltreatment_0to3(2)	10.421	1	.001
		Maltreatment_0to3(3)	8.528	1	.003
	Overall Statistics		21.776	3	<.001

Block 1: Method = Enter

Omnibus Tests of Model Coefficients

		Chi-square	df	Sig.
Step 1	Step	20.675	3	<.001
	Block	20.675	3	<.001
	Model	20.675	3	<.001

Model Summary

Step	-2 Log likelihood	Cox & Snell R Square	Nagelkerke R Square
1	7045.151[a]	.002	.004

a. Estimation terminated at iteration number 5 because parameter estimates changed by less than .001.

Classification Table[a]

Observed			Predicted Obesitas 1.00	Predicted Obesitas 2.00	Percentage Correct
Step 1	Obesitas	1.00	7806	0	100.0
		2.00	1199	0	.0
	Overall Percentage				86.7

a. The cut value is .500

Variables in the Equation

		B	S.E.	Wald	df	Sig.
Step 1[a]	Maltreatment_0to3			21.603	3	<.001
	Maltreatment_0to3(1)	.084	.084	.993	1	.319
	Maltreatment_0to3(2)	.386	.106	13.263	1	<.001
	Maltreatment_0to3(3)	.351	.104	11.355	1	<.001
	Constant	-1.956	.039	2466.865	1	.000

Variables in the Equation

		Exp(B)	95% C.I.for EXP(B) Lower	95% C.I.for EXP(B) Upper
Step 1[a]	Maltreatment_0to3			
	Maltreatment_0to3(1)	1.087	.922	1.282
	Maltreatment_0to3(2)	1.471	1.195	1.810
	Maltreatment_0to3(3)	1.421	1.158	1.743
	Constant	.141		

a. Variable(s) entered on step 1: Maltreatment_0to3.

Logistic Regression

Notes

Output Created		05-OCT-2024 11:47:48
Comments		
Input	Data	/Users/stevenlc/Library/<br>CloudStorage/OneDrive-<br>Privat/ICloud<br>filer/Doktorander/Rickar<br>d/Artikel 3/Revision art<br>3/Artikel3_revision.sav
	Active Dataset	DataSet7
	Filter	<none>
	Weight	<none>
	Split File	<none>
	N of Rows in Working Data File	10337
Missing Value Handling	Definition of Missing	User-defined missing values are treated as missing
Syntax		LOGISTIC REGRESSION<br>VARIABLES Fetma<br>/METHOD=ENTER<br>Maltreatment_0to3<br>Householddysf_parent<br>Kon barnboendeny<br>utbildningmammapappa_<br>ny<br>fodelselandmammapapp<br>a_ny alderskategorier<br>/CONTRAST<br>(Maltreatment_0to3)<br>=Indicator(1)<br>/CONTRAST<br>(Householddysf_parent)<br>=Indicator(1)<br>/CONTRAST (Kon)<br>=Indicator(1)<br>/CONTRAST<br>(barnboendeny)<br>=Indicator(1)<br>/CONTRAST<br>(utbildningmammapappa<br>_ny)=Indicator(1)<br>/CONTRAST<br>(fodelselandmammapap<br>pa_ny)=Indicator(1)<br>/CONTRAST<br>(alderskategorier)<br>=Indicator(1)<br>/PRINT=CI(95)<br>/CRITERIA=PIN(0.05)<br>POUT(0.10) ITERATE(20)<br>CUT(0.5).
Resources	Processor Time	00:00:00,44
	Elapsed Time	00:00:00,00

Case Processing Summary

Unweighted Cases[a]		N	Percent
Selected Cases	Included in Analysis	7506	72.6
	Missing Cases	2831	27.4
	Total	10337	100.0
Unselected Cases		0	.0
Total		10337	100.0

a. If weight is in effect, see classification table for the total number of cases.

Dependent Variable Encoding

Original Value	Internal Value
1.00	0
2.00	1

Categorical Variables Codings

			Parameter coding		
		Frequency	(1)	(2)	(3)
Age categories	17-25	897	.000	.000	.000
	26-35	1178	1.000	.000	.000
	36-45	1416	.000	1.000	.000
	46-55	1484	.000	.000	1.000
	56-65	1566	.000	.000	.000
	66-74	965	.000	.000	.000
Maltreatment_0to3	.00	5077	.000	.000	.000
	1.00	1279	1.000	.000	.000
	2.00	578	.000	1.000	.000
	3.00	572	.000	.000	1.000
Parent education	At least one parent high school	4009	.000		
	Both parents below high school	3497	1.000		
Householddysf_parent	.00	5668	.000		
	1.00	1838	1.000		
Sex	Man	3372	.000		
	Kvinna	4134	1.000		
Immigrant status	At least one Nordic parent	7032	.000		
	Both parents born elsewhere	474	1.000		
Residence type	Owned home	5627	.000		
	Rental	1879	1.000		

Categorical Variables Codings

		Parameter coding (4)	Parameter coding (5)
Age categories	17-25	.000	.000
	26-35	.000	.000
	36-45	.000	.000
	46-55	.000	.000
	56-65	1.000	.000
	66-74	.000	1.000
Maltreatment_0to3	.00		
	1.00		
	2.00		
	3.00		
Parent education	At least one parent high school		
	Both parents below high school		
Householddysf_parent	.00		
	1.00		
Sex	Man		
	Kvinna		
Immigrant status	At least one Nordic parent		
	Both parents born elsewhere		
Residence type	Owned home		
	Rental		

Block 0: Beginning Block

Classification Table[a,b]

Observed			Predicted Obesitas 1.00	Predicted Obesitas 2.00	Percentage Correct
Step 0	Obesitas	1.00	6545	0	100.0
		2.00	961	0	.0
	Overall Percentage				87.2

a. Constant is included in the model.

b. The cut value is .500

Variables in the Equation

		B	S.E.	Wald	df	Sig.	Exp(B)
Step 0	Constant	-1.918	.035	3084.182	1	.000	.147

Variables not in the Equation

			Score	df	Sig.
Step 0	Variables	Maltreatment_0to3	16.880	3	<.001
		Maltreatment_0to3(1)	.233	1	.630
		Maltreatment_0to3(2)	7.403	1	.007
		Maltreatment_0to3(3)	6.623	1	.010
		Householddysf_parent(1)	.447	1	.504
		Sex(1)	3.082	1	.079
		Residence type(1)	14.305	1	<.001
		Parent education(1)	80.149	1	<.001
		Immigrant status(1)	3.247	1	.072
		Age categories	82.292	5	<.001
		Age categories(1)	18.936	1	<.001
		Age categories(2)	.023	1	.880
		Age categories(3)	24.446	1	<.001
		Age categories(4)	7.174	1	.007
		Age categories(5)	3.244	1	.072
	Overall Statistics		137.342	13	<.001

Block 1: Method = Enter

Omnibus Tests of Model Coefficients

		Chi-square	df	Sig.
Step 1	Step	143.430	13	<.001
	Block	143.430	13	<.001
	Model	143.430	13	<.001

Model Summary

Step	-2 Log likelihood	Cox & Snell R Square	Nagelkerke R Square
1	5600.557[a]	.019	.035

a. Estimation terminated at iteration number 5 because parameter estimates changed by less than .001.

Classification Table[a]

			Predicted		
			Obesitas		Percentage Correct
	Observed		1.00	2.00	
Step 1	Obesitas	1.00	6545	0	100.0
		2.00	961	0	.0
	Overall Percentage				87.2

a. The cut value is .500

Variables in the Equation

		B	S.E.	Wald	df	Sig.
Step 1[a]	Maltreatment_0to3			11.367	3	.010
	Maltreatment_0to3(1)	.115	.095	1.467	1	.226
	Maltreatment_0to3(2)	.333	.123	7.297	1	.007
	Maltreatment_0to3(3)	.314	.129	5.878	1	.015
	Householddysf_parent(1)	-.053	.090	.344	1	.558
	Sex(1)	-.113	.070	2.607	1	.106
	Residence type(1)	.169	.081	4.408	1	.036
	Parent education(1)	.438	.080	29.952	1	<.001
	Immigrant status(1)	-.224	.160	1.968	1	.161
	Age categories			35.855	5	<.001
	Age categories(1)	.397	.175	5.116	1	.024
	Age categories(2)	.706	.165	18.317	1	<.001
	Age categories(3)	.884	.164	28.944	1	<.001
	Age categories(4)	.653	.170	14.773	1	<.001
	Age categories(5)	.612	.181	11.408	1	<.001
	Constant	-2.799	.152	337.778	1	<.001

Variables in the Equation

		Exp(B)	95% C.I.for EXP(B)	
			Lower	Upper
Step 1[a]	Maltreatment_0to3			
	Maltreatment_0to3(1)	1.122	.931	1.352
	Maltreatment_0to3(2)	1.396	1.096	1.778
	Maltreatment_0to3(3)	1.369	1.062	1.764
	Householddysf_parent(1)	.949	.795	1.132
	Sex(1)	.893	.778	1.025
	Residence type(1)	1.184	1.011	1.387
	Parent education(1)	1.549	1.324	1.812
	Immigrant status(1)	.799	.584	1.093
	Age categories			
	Age categories(1)	1.487	1.054	2.098
	Age categories(2)	2.025	1.466	2.798
	Age categories(3)	2.421	1.754	3.341
	Age categories(4)	1.920	1.377	2.679
	Age categories(5)	1.844	1.293	2.630
	Constant	.061		

a. Variable(s) entered on step 1: Maltreatment_0to3, Householddysf_parent, Sex, Residence type, Parent education, Immigrant status, Age categories.

Logistic Regression

Notes

Output Created		05-OCT-2024 11:47:48
Comments		
Input	Data	/Users/stevenlc/Library/CloudStorage/OneDrive-Privat/ICloud filer/Doktorander/Rickard/Artikel 3/Revision art 3/Artikel3_revision.sav
	Active Dataset	DataSet7
	Filter	<none>
	Weight	<none>
	Split File	<none>
	N of Rows in Working Data File	10337
Missing Value Handling	Definition of Missing	User-defined missing values are treated as missing
Syntax		LOGISTIC REGRESSION VARIABLES Fetma /METHOD=ENTER Lifetimeabuseindex /CONTRAST (Lifetimeabuseindex)=Indicator(1) /PRINT=CI(95) /CRITERIA=PIN(0.05) POUT(0.10) ITERATE(20) CUT(0.5).
Resources	Processor Time	00:00:00,40
	Elapsed Time	00:00:00,00

Case Processing Summary

Unweighted Cases[a]		N	Percent
Selected Cases	Included in Analysis	8797	85.1
	Missing Cases	1540	14.9
	Total	10337	100.0
Unselected Cases		0	.0
Total		10337	100.0

a. If weight is in effect, see classification table for the total number of cases.

Dependent Variable Encoding

Original Value	Internal Value
1.00	0
2.00	1

Categorical Variables Codings

		Frequency	Parameter coding (1)	(2)	(3)	(4)	(5)
Lifetimeabuseindex	.00	4951	.000	.000	.000	.000	.000
	1.00	1050	1.000	.000	.000	.000	.000
	2.00	444	.000	1.000	.000	.000	.000
	3.00	368	.000	.000	1.000	.000	.000
	4.00	863	.000	.000	.000	1.000	.000
	5.00	478	.000	.000	.000	.000	1.000
	6.00	262	.000	.000	.000	.000	.000
	7.00	381	.000	.000	.000	.000	.000

Categorical Variables Codings

		Parameter coding (6)	(7)
Lifetimeabuseindex	.00	.000	.000
	1.00	.000	.000
	2.00	.000	.000
	3.00	.000	.000
	4.00	.000	.000
	5.00	.000	.000
	6.00	1.000	.000
	7.00	.000	1.000

Block 0: Beginning Block

Classification Table[a,b]

Observed			Predicted: Obesitas 1.00	Obesitas 2.00	Percentage Correct
Step 0	Obesitas	1.00	7626	0	100.0
		2.00	1171	0	.0
	Overall Percentage				86.7

a. Constant is included in the model.

b. The cut value is .500

Variables in the Equation

		B	S.E.	Wald	df	Sig.	Exp(B)
Step 0	Constant	-1.874	.031	3563.868	1	.000	.154

Variables not in the Equation

			Score	df	Sig.
Step 0	Variables	Lifetimeabuseindex	29.215	7	<.001
		Lifetimeabuseindex(1)	.072	1	.789
		Lifetimeabuseindex(2)	11.739	1	<.001
		Lifetimeabuseindex(3)	1.209	1	.271
		Lifetimeabuseindex(4)	.735	1	.391
		Lifetimeabuseindex(5)	.218	1	.641
		Lifetimeabuseindex(6)	.580	1	.446
		Lifetimeabuseindex(7)	8.841	1	.003
	Overall Statistics		29.215	7	<.001

Block 1: Method = Enter

Omnibus Tests of Model Coefficients

		Chi-square	df	Sig.
Step 1	Step	27.516	7	<.001
	Block	27.516	7	<.001
	Model	27.516	7	<.001

Model Summary

Step	-2 Log likelihood	Cox & Snell R Square	Nagelkerke R Square
1	6873.958 [a]	.003	.006

a. Estimation terminated at iteration number 5 because parameter estimates changed by less than .001.

Classification Table [a]

Observed			Predicted: Obesitas 1.00	Predicted: Obesitas 2.00	Percentage Correct
Step 1	Obesitas	1.00	7626	0	100.0
		2.00	1171	0	.0
	Overall Percentage				86.7

a. The cut value is .500

Variables in the Equation

		B	S.E.	Wald	df	Sig.
Step 1[a]	Lifetimeabuseindex			28.862	7	<.001
	Lifetimeabuseindex(1)	.092	.101	.823	1	.364
	Lifetimeabuseindex(2)	.519	.129	16.092	1	<.001
	Lifetimeabuseindex(3)	.271	.152	3.202	1	.074
	Lifetimeabuseindex(4)	.194	.107	3.317	1	.069
	Lifetimeabuseindex(5)	.175	.139	1.588	1	.208
	Lifetimeabuseindex(6)	.245	.179	1.877	1	.171
	Lifetimeabuseindex(7)	.498	.139	12.755	1	<.001
	Constant	-1.989	.044	2073.676	1	.000

Variables in the Equation

		Exp(B)	95% C.I.for EXP(B) Lower	Upper
Step 1[a]	Lifetimeabuseindex			
	Lifetimeabuseindex(1)	1.096	.899	1.338
	Lifetimeabuseindex(2)	1.680	1.304	2.165
	Lifetimeabuseindex(3)	1.312	.974	1.765
	Lifetimeabuseindex(4)	1.215	.985	1.497
	Lifetimeabuseindex(5)	1.191	.907	1.564
	Lifetimeabuseindex(6)	1.278	.900	1.815
	Lifetimeabuseindex(7)	1.645	1.252	2.161
	Constant	.137		

a. Variable(s) entered on step 1: Lifetimeabuseindex.

Logistic Regression

Notes

Output Created		05-OCT-2024 11:47:49
Comments		
Input	Data	/Users/stevenlc/Library/CloudStorage/OneDrive-Privat/ICloud filer/Doktorander/Rickard/Artikel 3/Revision art 3/Artikel3_revision.sav
	Active Dataset	DataSet7
	Filter	<none>
	Weight	<none>
	Split File	<none>
	N of Rows in Working Data File	10337
Missing Value Handling	Definition of Missing	User-defined missing values are treated as missing
Syntax		LOGISTIC REGRESSION VARIABLES Fetma<br>/METHOD=ENTER Lifetimeabuseindex Householddysf_parent Kon barnboendeny utbildningmammapappa_ny fodelselandmammapappa_ny alderskategorier<br>/CONTRAST (Lifetimeabuseindex)=Indicator(1)<br>/CONTRAST (Householddysf_parent)=Indicator(1)<br>/CONTRAST (Kon)=Indicator(1)<br>/CONTRAST (barnboendeny)=Indicator(1)<br>/CONTRAST (utbildningmammapappa_ny)=Indicator(1)<br>/CONTRAST (fodelselandmammapappa_ny)=Indicator(1)<br>/CONTRAST (alderskategorier)=Indicator(1)<br>/PRINT=CI(95)<br>/CRITERIA=PIN(0.05) POUT(0.10) ITERATE(20) CUT(0.5).
Resources	Processor Time	00:00:00,46
	Elapsed Time	00:00:00,00

Case Processing Summary

Unweighted Cases[a]		N	Percent
Selected Cases	Included in Analysis	7353	71.1
	Missing Cases	2984	28.9
	Total	10337	100.0
Unselected Cases		0	.0
Total		10337	100.0

a. If weight is in effect, see classification table for the total number of cases.

Dependent Variable Encoding

Original Value	Internal Value
1.00	0
2.00	1

Categorical Variables Codings

		Frequency	Parameter coding		
			(1)	(2)	(3)
Lifetimeabuseindex	.00	4251	.000	.000	.000
	1.00	857	1.000	.000	.000
	2.00	346	.000	1.000	.000
	3.00	265	.000	.000	1.000
	4.00	729	.000	.000	.000
	5.00	395	.000	.000	.000
	6.00	218	.000	.000	.000
	7.00	292	.000	.000	.000
Age categories	17-25	876	.000	.000	.000
	26-35	1155	1.000	.000	.000
	36-45	1390	.000	1.000	.000
	46-55	1452	.000	.000	1.000
	56-65	1535	.000	.000	.000
	66-74	945	.000	.000	.000
Parent education	At least one parent high school	3914	.000		
	Both parents below high school	3439	1.000		
Householddysf_parent	.00	5560	.000		
	1.00	1793	1.000		
Sex	Man	3299	.000		
	Kvinna	4054	1.000		
Immigrant status	At least one Nordic parent	6888	.000		
	Both parents born elsewhere	465	1.000		
Residence type	Owned home	5507	.000		
	Rental	1846	1.000		

Categorical Variables Codings

		Parameter coding (4)	(5)	(6)	(7)
Lifetimeabuseindex	.00	.000	.000	.000	.000
	1.00	.000	.000	.000	.000
	2.00	.000	.000	.000	.000
	3.00	.000	.000	.000	.000
	4.00	1.000	.000	.000	.000
	5.00	.000	1.000	.000	.000
	6.00	.000	.000	1.000	.000
	7.00	.000	.000	.000	1.000
Age categories	17-25	.000	.000		
	26-35	.000	.000		
	36-45	.000	.000		
	46-55	.000	.000		
	56-65	1.000	.000		
	66-74	.000	1.000		
Parent education	At least one parent high school				
	Both parents below high school				
Householddysf_parent	.00				
	1.00				
Sex	Man				
	Kvinna				
Immigrant status	At least one Nordic parent				
	Both parents born elsewhere				
Residence type	Owned home				
	Rental				

Block 0: Beginning Block

Classification Table[a,b]

Observed			Predicted Obesitas 1.00	2.00	Percentage Correct
Step 0	Obesitas	1.00	6412	0	100.0
		2.00	941	0	.0
	Overall Percentage				87.2

a. Constant is included in the model.

b. The cut value is .500

Variables in the Equation

		B	S.E.	Wald	df	Sig.	Exp(B)
Step 0	Constant	-1.919	.035	3021.767	1	.000	.147

Variables not in the Equation

			Score	df	Sig.
Step 0	Variables	Lifetimeabuseindex	19.543	7	.007
		Lifetimeabuseindex(1)	.131	1	.718
		Lifetimeabuseindex(2)	6.717	1	.010
		Lifetimeabuseindex(3)	2.294	1	.130
		Lifetimeabuseindex(4)	.302	1	.582
		Lifetimeabuseindex(5)	.050	1	.822
		Lifetimeabuseindex(6)	1.102	1	.294
		Lifetimeabuseindex(7)	4.323	1	.038
		Householddysf_parent(1)	.473	1	.492
		Sex(1)	3.282	1	.070
		Residence type(1)	12.410	1	<.001
		Parent education(1)	80.068	1	<.001
		Immigrant status(1)	3.754	1	.053
		Age categories	84.205	5	<.001
		Age categories(1)	18.482	1	<.001
		Age categories(2)	.036	1	.850
		Age categories(3)	26.932	1	<.001
		Age categories(4)	5.603	1	.018
		Age categories(5)	3.550	1	.060
	Overall Statistics		142.132	17	<.001

Block 1: Method = Enter

Omnibus Tests of Model Coefficients

		Chi-square	df	Sig.
Step 1	Step	148.753	17	<.001
	Block	148.753	17	<.001
	Model	148.753	17	<.001

Model Summary

Step	-2 Log likelihood	Cox & Snell R Square	Nagelkerke R Square
1	5476.571 [a]	.020	.037

a. Estimation terminated at iteration number 5 because parameter estimates changed by less than .001.

Classification Table[a]

Observed			Predicted: Obesitas 1.00	Predicted: Obesitas 2.00	Percentage Correct
Step 1	Obesitas	1.00	6412	0	100.0
		2.00	941	0	.0
	Overall Percentage				87.2

a. The cut value is .500

Variables in the Equation

		B	S.E.	Wald	df	Sig.
Step 1[a]	Lifetimeabuseindex			15.147	7	.034
	Lifetimeabuseindex(1)	.129	.113	1.302	1	.254
	Lifetimeabuseindex(2)	.412	.153	7.247	1	.007
	Lifetimeabuseindex(3)	.289	.181	2.551	1	.110
	Lifetimeabuseindex(4)	.204	.120	2.871	1	.090
	Lifetimeabuseindex(5)	.181	.159	1.299	1	.254
	Lifetimeabuseindex(6)	.309	.199	2.419	1	.120
	Lifetimeabuseindex(7)	.422	.172	6.032	1	.014
	Householddysf_parent(1)	-.060	.091	.427	1	.513
	Sex(1)	-.118	.071	2.770	1	.096
	Residence type(1)	.150	.082	3.394	1	.065
	Parent education(1)	.447	.081	30.394	1	<.001
	Immigrant status(1)	-.238	.163	2.130	1	.144
	Age categories			37.269	5	<.001
	Age categories(1)	.429	.179	5.719	1	.017
	Age categories(2)	.730	.169	18.699	1	<.001
	Age categories(3)	.929	.168	30.452	1	<.001
	Age categories(4)	.671	.174	14.873	1	<.001
	Age categories(5)	.660	.185	12.712	1	<.001
	Constant	-2.860	.157	330.259	1	<.001

Variables in the Equation

		Exp(B)	95% C.I.for EXP(B) Lower	Upper
Step 1[a]	Lifetimeabuseindex			
	Lifetimeabuseindex(1)	1.138	.911	1.421
	Lifetimeabuseindex(2)	1.509	1.118	2.037
	Lifetimeabuseindex(3)	1.335	.936	1.904
	Lifetimeabuseindex(4)	1.226	.969	1.553
	Lifetimeabuseindex(5)	1.199	.878	1.638
	Lifetimeabuseindex(6)	1.362	.923	2.010
	Lifetimeabuseindex(7)	1.525	1.089	2.136
	Householddysf_parent(1)	.942	.788	1.127
	Sex(1)	.888	.773	1.021
	Residence type(1)	1.162	.990	1.363
	Parent education(1)	1.563	1.334	1.832
	Immigrant status(1)	.788	.573	1.085
	Age categories			
	Age categories(1)	1.536	1.080	2.183
	Age categories(2)	2.076	1.491	2.891
	Age categories(3)	2.531	1.820	3.521
	Age categories(4)	1.956	1.391	2.750
	Age categories(5)	1.934	1.346	2.780
	Constant	.057		

a. Variable(s) entered on step 1: Lifetimeabuseindex, Householddysf_parent, Sex, Residence type, Parent education, Immigrant status, Age categories.

Logistic Regression

Notes

Output Created		05-OCT-2024 11:47:49
Comments		
Input	Data	/Users/stevenlc/Library/CloudStorage/OneDrive-Privat/ICloud filer/Doktorander/Rickard/Artikel 3/Revision art 3/Artikel3_revision.sav
	Active Dataset	DataSet7
	Filter	<none>
	Weight	<none>
	Split File	<none>
	N of Rows in Working Data File	10337
Missing Value Handling	Definition of Missing	User-defined missing values are treated as missing
Syntax		LOGISTIC REGRESSION VARIABLES storrökare /METHOD=ENTER Maltreatment_0to3 /CONTRAST (Maltreatment_0to3)=Indicator(1) /PRINT=CI(95) /CRITERIA=PIN(0.05) POUT(0.10) ITERATE(20) CUT(0.5).
Resources	Processor Time	00:00:00,39
	Elapsed Time	00:00:00,00

Case Processing Summary

Unweighted Cases[a]		N	Percent
Selected Cases	Included in Analysis	9067	87.7
	Missing Cases	1270	12.3
	Total	10337	100.0
Unselected Cases		0	.0
Total		10337	100.0

a. If weight is in effect, see classification table for the total number of cases.

Dependent Variable Encoding

Original Value	Internal Value
1.00	0
2.00	1

Categorical Variables Codings

		Frequency	Parameter coding (1)	(2)	(3)
Maltreatment_0to3	.00	5989	.000	.000	.000
	1.00	1560	1.000	.000	.000
	2.00	731	.000	1.000	.000
	3.00	787	.000	.000	1.000

Block 0: Beginning Block

Classification Table[a,b]

	Observed		Predicted: Heavy smoking 1.00	2.00	Percentage Correct
Step 0	Heavy smoking	1.00	8700	0	100.0
		2.00	367	0	.0
	Overall Percentage				96.0

a. Constant is included in the model.

b. The cut value is .500

Variables in the Equation

		B	S.E.	Wald	df	Sig.	Exp(B)
Step 0	Constant	-3.166	.053	3529.114	1	.000	.042

Variables not in the Equation

			Score	df	Sig.
Step 0	Variables	Maltreatment_0to3	79.520	3	<.001
		Maltreatment_0to3(1)	1.564	1	.211
		Maltreatment_0to3(2)	24.740	1	<.001
		Maltreatment_0to3(3)	39.358	1	<.001
	Overall Statistics		79.520	3	<.001

Block 1: Method = Enter

Omnibus Tests of Model Coefficients

		Chi-square	df	Sig.
Step 1	Step	69.010	3	<.001
	Block	69.010	3	<.001
	Model	69.010	3	<.001

Model Summary

Step	-2 Log likelihood	Cox & Snell R Square	Nagelkerke R Square
1	3003.894[a]	.008	.026

a. Estimation terminated at iteration number 6 because parameter estimates changed by less than .001.

Classification Table[a]

Observed			Predicted: Heavy smoking 1.00	Predicted: Heavy smoking 2.00	Percentage Correct
Step 1	Heavy smoking	1.00	8700	0	100.0
		2.00	367	0	.0
	Overall Percentage				96.0

a. The cut value is .500

Variables in the Equation

		B	S.E.	Wald	df	Sig.
Step 1[a]	Maltreatment_0to3			74.161	3	<.001
	Maltreatment_0to3(1)	.475	.143	11.021	1	<.001
	Maltreatment_0to3(2)	.994	.160	38.704	1	<.001
	Maltreatment_0to3(3)	1.096	.151	52.981	1	<.001
	Constant	-3.503	.077	2084.962	1	.000

Variables in the Equation

		Exp(B)	95% C.I.for EXP(B) Lower	95% C.I.for EXP(B) Upper
Step 1[a]	Maltreatment_0to3			
	Maltreatment_0to3(1)	1.608	1.215	2.128
	Maltreatment_0to3(2)	2.703	1.976	3.697
	Maltreatment_0to3(3)	2.991	2.227	4.017
	Constant	.030		

a. Variable(s) entered on step 1: Maltreatment_0to3.

Logistic Regression

Notes

Output Created		05-OCT-2024 11:47:49
Comments		
Input	Data	/Users/stevenlc/Library/CloudStorage/OneDrive-Privat/ICloud filer/Doktorander/Rickard/Artikel 3/Revision art 3/Artikel3_revision.sav
	Active Dataset	DataSet7
	Filter	<none>
	Weight	<none>
	Split File	<none>
	N of Rows in Working Data File	10337
Missing Value Handling	Definition of Missing	User-defined missing values are treated as missing
Syntax		LOGISTIC REGRESSION VARIABLES storrökare /METHOD=ENTER Maltreatment_0to3 Householddysf_parent Kon barnboendeny utbildningmammapappa_ny fodelselandmammapappa_ny alderskategorier /CONTRAST (Maltreatment_0to3)=Indicator(1) /CONTRAST (Householddysf_parent)=Indicator(1) /CONTRAST (Kon)=Indicator(1) /CONTRAST (barnboendeny)=Indicator(1) /CONTRAST (utbildningmammapappa_ny)=Indicator(1) /CONTRAST (fodelselandmammapappa_ny)=Indicator(1) /CONTRAST (alderskategorier)=Indicator(1) /PRINT=CI(95) /CRITERIA=PIN(0.05) POUT(0.10) ITERATE(20) CUT(0.5).
Resources	Processor Time	00:00:00,47
	Elapsed Time	00:00:01,00

Case Processing Summary

Unweighted Cases[a]		N	Percent
Selected Cases	Included in Analysis	7556	73.1
	Missing Cases	2781	26.9
	Total	10337	100.0
Unselected Cases		0	.0
Total		10337	100.0

a. If weight is in effect, see classification table for the total number of cases.

Dependent Variable Encoding

Original Value	Internal Value
1.00	0
2.00	1

Categorical Variables Codings

			Parameter coding		
		Frequency	(1)	(2)	(3)
Age categories	17-25	905	.000	.000	.000
	26-35	1191	1.000	.000	.000
	36-45	1421	.000	1.000	.000
	46-55	1498	.000	.000	1.000
	56-65	1571	.000	.000	.000
	66-74	970	.000	.000	.000
Maltreatment_0to3	.00	5121	.000	.000	.000
	1.00	1271	1.000	.000	.000
	2.00	579	.000	1.000	.000
	3.00	585	.000	.000	1.000
Parent education	At least one parent high school	4040	.000		
	Both parents below high school	3516	1.000		
Householddysf_parent	.00	5714	.000		
	1.00	1842	1.000		
Sex	Man	3382	.000		
	Kvinna	4174	1.000		
Immigrant status	At least one Nordic parent	7076	.000		
	Both parents born elsewhere	480	1.000		
Residence type	Owned home	5656	.000		
	Rental	1900	1.000		

Categorical Variables Codings

		Parameter coding (4)	Parameter coding (5)
Age categories	17-25	.000	.000
	26-35	.000	.000
	36-45	.000	.000
	46-55	.000	.000
	56-65	1.000	.000
	66-74	.000	1.000
Maltreatment_0to3	.00		
	1.00		
	2.00		
	3.00		
Parent education	At least one parent high school		
	Both parents below high school		
Householddysf_parent	.00		
	1.00		
Sex	Man		
	Kvinna		
Immigrant status	At least one Nordic parent		
	Both parents born elsewhere		
Residence type	Owned home		
	Rental		

Block 0: Beginning Block

Classification Table[a,b]

	Observed		Predicted Heavy smoking 1.00	Predicted Heavy smoking 2.00	Percentage Correct
Step 0	Heavy smoking	1.00	7270	0	100.0
		2.00	286	0	.0
	Overall Percentage				96.2

a. Constant is included in the model.

b. The cut value is .500

Variables in the Equation

		B	S.E.	Wald	df	Sig.	Exp(B)
Step 0	Constant	-3.236	.060	2880.691	1	.000	.039

Variables not in the Equation

			Score	df	Sig.
Step 0	Variables	Maltreatment_0to3	79.824	3	<.001
		Maltreatment_0to3(1)	2.054	1	.152
		Maltreatment_0to3(2)	25.050	1	<.001
		Maltreatment_0to3(3)	39.482	1	<.001
		Householddysf_parent(1)	5.885	1	.015
		Sex(1)	35.273	1	<.001
		Residence type(1)	31.020	1	<.001
		Parent education(1)	17.808	1	<.001
		Immigrant status(1)	21.663	1	<.001
		Age categories	92.731	5	<.001
		Age categories(1)	15.870	1	<.001
		Age categories(2)	14.621	1	<.001
		Age categories(3)	6.074	1	.014
		Age categories(4)	39.926	1	<.001
		Age categories(5)	9.703	1	.002
	Overall Statistics		229.973	13	<.001

Block 1: Method = Enter

Omnibus Tests of Model Coefficients

		Chi-square	df	Sig.
Step 1	Step	226.716	13	<.001
	Block	226.716	13	<.001
	Model	226.716	13	<.001

Model Summary

Step	-2 Log likelihood	Cox & Snell R Square	Nagelkerke R Square
1	2207.108[a]	.030	.107

a. Estimation terminated at iteration number 8 because parameter estimates changed by less than .001.

Classification Table[a]

	Observed		Predicted		
			Heavy smoking		Percentage Correct
			1.00	2.00	
Step 1	Heavy smoking	1.00	7270	0	100.0
		2.00	286	0	.0
	Overall Percentage				96.2

a. The cut value is .500

Variables in the Equation

		B	S.E.	Wald	df	Sig.
Step 1[a]	Maltreatment_0to3			43.809	3	<.001
	Maltreatment_0to3(1)	.492	.165	8.853	1	.003
	Maltreatment_0to3(2)	.985	.187	27.878	1	<.001
	Maltreatment_0to3(3)	1.003	.189	28.173	1	<.001
	Householddysf_parent(1)	.327	.150	4.761	1	.029
	Sex(1)	-.753	.127	35.139	1	<.001
	Residence type(1)	.282	.132	4.570	1	.033
	Parent education(1)	-.008	.137	.004	1	.952
	Immigrant status(1)	.865	.196	19.506	1	<.001
	Age categories			63.848	5	<.001
	Age categories(1)	.733	.441	2.759	1	.097
	Age categories(2)	.954	.427	5.000	1	.025
	Age categories(3)	1.826	.405	20.269	1	<.001
	Age categories(4)	2.124	.406	27.387	1	<.001
	Age categories(5)	2.062	.421	24.035	1	<.001
	Constant	-5.036	.394	163.062	1	<.001

Variables in the Equation

		Exp(B)	95% C.I.for EXP(B) Lower	Upper
Step 1[a]	Maltreatment_0to3			
	Maltreatment_0to3(1)	1.636	1.183	2.262
	Maltreatment_0to3(2)	2.677	1.858	3.859
	Maltreatment_0to3(3)	2.726	1.882	3.947
	Householddysf_parent(1)	1.387	1.034	1.861
	Sex(1)	.471	.367	.604
	Residence type(1)	1.325	1.024	1.716
	Parent education(1)	.992	.759	1.297
	Immigrant status(1)	2.374	1.618	3.485
	Age categories			
	Age categories(1)	2.082	.876	4.946
	Age categories(2)	2.597	1.125	5.994
	Age categories(3)	6.206	2.803	13.739
	Age categories(4)	8.366	3.776	18.537
	Age categories(5)	7.863	3.448	17.933
	Constant	.007		

a. Variable(s) entered on step 1: Maltreatment_0to3, Householddysf_parent, Sex, Residence type, Parent education, Immigrant status, Age categories.

Logistic Regression

Notes

Output Created		05-OCT-2024 11:47:50
Comments		
Input	Data	/Users/stevenlc/Library/CloudStorage/OneDrive-Privat/ICloud filer/Doktorander/Rickard/Artikel 3/Revision art 3/Artikel3_revision.sav
	Active Dataset	DataSet7
	Filter	<none>
	Weight	<none>
	Split File	<none>
	N of Rows in Working Data File	10337
Missing Value Handling	Definition of Missing	User-defined missing values are treated as missing
Syntax		LOGISTIC REGRESSION VARIABLES storrökare /METHOD=ENTER Lifetimeabuseindex /CONTRAST (Lifetimeabuseindex)=Indicator(1) /PRINT=CI(95) /CRITERIA=PIN(0.05) POUT(0.10) ITERATE(20) CUT(0.5).
Resources	Processor Time	00:00:00,40
	Elapsed Time	00:00:00,00

Case Processing Summary

Unweighted Cases[a]		N	Percent
Selected Cases	Included in Analysis	8856	85.7
	Missing Cases	1481	14.3
	Total	10337	100.0
Unselected Cases		0	.0
Total		10337	100.0

a. If weight is in effect, see classification table for the total number of cases.

Dependent Variable Encoding

Original Value	Internal Value
1.00	0
2.00	1

Categorical Variables Codings

		Frequency	Parameter coding (1)	(2)	(3)	(4)	(5)
Lifetimeabuseindex	.00	4994	.000	.000	.000	.000	.000
	1.00	1044	1.000	.000	.000	.000	.000
	2.00	442	.000	1.000	.000	.000	.000
	3.00	376	.000	.000	1.000	.000	.000
	4.00	870	.000	.000	.000	1.000	.000
	5.00	473	.000	.000	.000	.000	1.000
	6.00	268	.000	.000	.000	.000	.000
	7.00	389	.000	.000	.000	.000	.000

Categorical Variables Codings

		Parameter coding (6)	(7)
Lifetimeabuseindex	.00	.000	.000
	1.00	.000	.000
	2.00	.000	.000
	3.00	.000	.000
	4.00	.000	.000
	5.00	.000	.000
	6.00	1.000	.000
	7.00	.000	1.000

Block 0: Beginning Block

Classification Table[a,b]

Observed			Predicted: Heavy smoking 1.00	Heavy smoking 2.00	Percentage Correct
Step 0	Heavy smoking	1.00	8500	0	100.0
		2.00	356	0	.0
	Overall Percentage				96.0

a. Constant is included in the model.

b. The cut value is .500

Variables in the Equation

		B	S.E.	Wald	df	Sig.	Exp(B)
Step 0	Constant	-3.173	.054	3439.866	1	.000	.042

Variables not in the Equation

			Score	df	Sig.
Step 0	Variables	Lifetimeabuseindex	85.457	7	<.001
		Lifetimeabuseindex(1)	1.816	1	.178
		Lifetimeabuseindex(2)	5.260	1	.022
		Lifetimeabuseindex(3)	7.035	1	.008
		Lifetimeabuseindex(4)	1.179	1	.278
		Lifetimeabuseindex(5)	.056	1	.812
		Lifetimeabuseindex(6)	26.259	1	<.001
		Lifetimeabuseindex(7)	28.896	1	<.001
	Overall Statistics		85.457	7	<.001

Block 1: Method = Enter

Omnibus Tests of Model Coefficients

		Chi-square	df	Sig.
Step 1	Step	71.226	7	<.001
	Block	71.226	7	<.001
	Model	71.226	7	<.001

Model Summary

Step	-2 Log likelihood	Cox & Snell R Square	Nagelkerke R Square
1	2914.578[a]	.008	.028

a. Estimation terminated at iteration number 6 because parameter estimates changed by less than .001.

Classification Table[a]

			Predicted		
			Heavy smoking		Percentage Correct
	Observed		1.00	2.00	
Step 1	Heavy smoking	1.00	8500	0	100.0
		2.00	356	0	.0
	Overall Percentage				96.0

a. The cut value is .500

Variables in the Equation

		B	S.E.	Wald	df	Sig.
Step 1[a]	Lifetimeabuseindex			78.406	7	<.001
	Lifetimeabuseindex(1)	.542	.168	10.382	1	.001
	Lifetimeabuseindex(2)	.799	.216	13.668	1	<.001
	Lifetimeabuseindex(3)	.889	.224	15.790	1	<.001
	Lifetimeabuseindex(4)	.164	.207	.627	1	.429
	Lifetimeabuseindex(5)	.411	.244	2.843	1	.092
	Lifetimeabuseindex(6)	1.342	.220	37.203	1	<.001
	Lifetimeabuseindex(7)	1.248	.195	41.164	1	<.001
	Constant	-3.531	.085	1720.420	1	.000

Variables in the Equation

			95% C.I.for EXP(B)	
		Exp(B)	Lower	Upper
Step 1[a]	Lifetimeabuseindex			
	Lifetimeabuseindex(1)	1.719	1.236	2.389
	Lifetimeabuseindex(2)	2.223	1.455	3.395
	Lifetimeabuseindex(3)	2.434	1.569	3.774
	Lifetimeabuseindex(4)	1.178	.785	1.768
	Lifetimeabuseindex(5)	1.509	.935	2.433
	Lifetimeabuseindex(6)	3.828	2.487	5.893
	Lifetimeabuseindex(7)	3.485	2.380	5.102
	Constant	.029		

a. Variable(s) entered on step 1: Lifetimeabuseindex.

Logistic Regression

Notes

Output Created		05-OCT-2024 11:47:50
Comments		
Input	Data	/Users/stevenlc/Library/CloudStorage/OneDrive-Privat/ICloud filer/Doktorander/Rickard/Artikel 3/Revision art 3/Artikel3_revision.sav
	Active Dataset	DataSet7
	Filter	<none>
	Weight	<none>
	Split File	<none>
	N of Rows in Working Data File	10337
Missing Value Handling	Definition of Missing	User-defined missing values are treated as missing
Syntax		LOGISTIC REGRESSION VARIABLES storrökare /METHOD=ENTER Lifetimeabuseindex Householddysf_parent Kon barnboendeny utbildningmammapappa_ny fodelselandmammapappa_ny alderskategorier /CONTRAST (Lifetimeabuseindex)=Indicator(1) /CONTRAST (Householddysf_parent)=Indicator(1) /CONTRAST (Kon)=Indicator(1) /CONTRAST (barnboendeny)=Indicator(1) /CONTRAST (utbildningmammapappa_ny)=Indicator(1) /CONTRAST (fodelselandmammapappa_ny)=Indicator(1) /CONTRAST (alderskategorier)=Indicator(1) /PRINT=CI(95) /CRITERIA=PIN(0.05) POUT(0.10) ITERATE(20) CUT(0.5).
Resources	Processor Time	00:00:00,49
	Elapsed Time	00:00:01,00

Case Processing Summary

Unweighted Cases[a]		N	Percent
Selected Cases	Included in Analysis	7402	71.6
	Missing Cases	2935	28.4
	Total	10337	100.0
Unselected Cases		0	.0
Total		10337	100.0

a. If weight is in effect, see classification table for the total number of cases.

Dependent Variable Encoding

Original Value	Internal Value
1.00	0
2.00	1

Categorical Variables Codings

		Frequency	Parameter coding (1)	(2)	(3)
Lifetimeabuseindex	.00	4291	.000	.000	.000
	1.00	853	1.000	.000	.000
	2.00	343	.000	1.000	.000
	3.00	273	.000	.000	1.000
	4.00	734	.000	.000	.000
	5.00	390	.000	.000	.000
	6.00	221	.000	.000	.000
	7.00	297	.000	.000	.000
Age categories	17-25	884	.000	.000	.000
	26-35	1169	1.000	.000	.000
	36-45	1394	.000	1.000	.000
	46-55	1466	.000	.000	1.000
	56-65	1541	.000	.000	.000
	66-74	948	.000	.000	.000
Parent education	At least one parent high school	3945	.000		
	Both parents below high school	3457	1.000		
Householddysf_parent	.00	5605	.000		
	1.00	1797	1.000		
Sex	Man	3310	.000		
	Kvinna	4092	1.000		
Immigrant status	At least one Nordic parent	6931	.000		
	Both parents born elsewhere	471	1.000		
Residence type	Owned home	5534	.000		
	Rental	1868	1.000		

Categorical Variables Codings

		Parameter coding (4)	(5)	(6)	(7)
Lifetimeabuseindex	.00	.000	.000	.000	.000
	1.00	.000	.000	.000	.000
	2.00	.000	.000	.000	.000
	3.00	.000	.000	.000	.000
	4.00	1.000	.000	.000	.000
	5.00	.000	1.000	.000	.000
	6.00	.000	.000	1.000	.000
	7.00	.000	.000	.000	1.000
Age categories	17-25	.000	.000		
	26-35	.000	.000		
	36-45	.000	.000		
	46-55	.000	.000		
	56-65	1.000	.000		
	66-74	.000	1.000		
Parent education	At least one parent high school				
	Both parents below high school				
Householddysf_parent	.00				
	1.00				
Sex	Man				
	Kvinna				
Immigrant status	At least one Nordic parent				
	Both parents born elsewhere				
Residence type	Owned home				
	Rental				

Block 0: Beginning Block

Classification Table[a,b]

Observed			Predicted Heavy smoking 1.00	2.00	Percentage Correct
Step 0	Heavy smoking	1.00	7123	0	100.0
		2.00	279	0	.0
	Overall Percentage				96.2

a. Constant is included in the model.

b. The cut value is .500

Variables in the Equation

		B	S.E.	Wald	df	Sig.	Exp(B)
Step 0	Constant	-3.240	.061	2818.214	1	.000	.039

Variables not in the Equation

			Score	df	Sig.
Step 0	Variables	Lifetimeabuseindex	96.504	7	<.001
		Lifetimeabuseindex(1)	2.860	1	.091
		Lifetimeabuseindex(2)	2.168	1	.141
		Lifetimeabuseindex(3)	6.233	1	.013
		Lifetimeabuseindex(4)	.560	1	.454
		Lifetimeabuseindex(5)	.037	1	.848
		Lifetimeabuseindex(6)	35.733	1	<.001
		Lifetimeabuseindex(7)	34.200	1	<.001
		Householddysf_parent(1)	6.760	1	.009
		Sex(1)	33.620	1	<.001
		Residence type(1)	29.398	1	<.001
		Parent education(1)	18.014	1	<.001
		Immigrant status(1)	18.594	1	<.001
		Age categories	91.068	5	<.001
		Age categories(1)	16.216	1	<.001
		Age categories(2)	14.678	1	<.001
		Age categories(3)	6.574	1	.010
		Age categories(4)	37.826	1	<.001
		Age categories(5)	9.945	1	.002
	Overall Statistics		247.547	17	<.001

Block 1: Method = Enter

Omnibus Tests of Model Coefficients

		Chi-square	df	Sig.
Step 1	Step	239.727	17	<.001
	Block	239.727	17	<.001
	Model	239.727	17	<.001

Model Summary

Step	-2 Log likelihood	Cox & Snell R Square	Nagelkerke R Square
1	2136.910[a]	.032	.116

a. Estimation terminated at iteration number 8 because parameter estimates changed by less than .001.

Classification Table[a]

Observed			Predicted: Heavy smoking 1.00	Predicted: Heavy smoking 2.00	Percentage Correct
Step 1	Heavy smoking	1.00	7123	0	100.0
		2.00	279	0	.0
	Overall Percentage				96.2

a. The cut value is .500

Variables in the Equation

		B	S.E.	Wald	df	Sig.
Step 1[a]	Lifetimeabuseindex			67.600	7	<.001
	Lifetimeabuseindex(1)	.588	.192	9.407	1	.002
	Lifetimeabuseindex(2)	.540	.268	4.050	1	.044
	Lifetimeabuseindex(3)	.615	.278	4.898	1	.027
	Lifetimeabuseindex(4)	.422	.233	3.276	1	.070
	Lifetimeabuseindex(5)	.406	.296	1.879	1	.170
	Lifetimeabuseindex(6)	1.649	.244	45.647	1	<.001
	Lifetimeabuseindex(7)	1.418	.235	36.432	1	<.001
	Householddysf_parent(1)	.365	.152	5.757	1	.016
	Sex(1)	-.796	.130	37.475	1	<.001
	Residence type(1)	.266	.134	3.968	1	.046
	Parent education(1)	.023	.139	.028	1	.867
	Immigrant status(1)	.872	.201	18.875	1	<.001
	Age categories			67.744	5	<.001
	Age categories(1)	.656	.445	2.172	1	.141
	Age categories(2)	.890	.429	4.298	1	.038
	Age categories(3)	1.802	.407	19.624	1	<.001
	Age categories(4)	2.141	.407	27.615	1	<.001
	Age categories(5)	2.095	.423	24.579	1	<.001
	Constant	-5.097	.398	164.170	1	<.001

Variables in the Equation

		Exp(B)	95% C.I.for EXP(B) Lower	Upper
Step 1[a]	Lifetimeabuseindex			
	Lifetimeabuseindex(1)	1.800	1.236	2.620
	Lifetimeabuseindex(2)	1.715	1.014	2.901
	Lifetimeabuseindex(3)	1.850	1.073	3.189
	Lifetimeabuseindex(4)	1.525	.966	2.407
	Lifetimeabuseindex(5)	1.500	.840	2.679
	Lifetimeabuseindex(6)	5.203	3.224	8.395
	Lifetimeabuseindex(7)	4.129	2.606	6.544
	Householddysf_parent(1)	1.440	1.069	1.941
	Sex(1)	.451	.350	.582
	Residence type(1)	1.305	1.004	1.695
	Parent education(1)	1.023	.780	1.344
	Immigrant status(1)	2.392	1.614	3.544
	Age categories			
	Age categories(1)	1.927	.805	4.611
	Age categories(2)	2.435	1.050	5.648
	Age categories(3)	6.062	2.731	13.456
	Age categories(4)	8.510	3.829	18.912
	Age categories(5)	8.125	3.549	18.599
	Constant	.006		

a. Variable(s) entered on step 1: Lifetimeabuseindex, Householddysf_parent, Sex, Residence type, Parent education, Immigrant status, Age categories.

Logistic Regression

Notes

Output Created		05-OCT-2024 11:47:51
Comments		
Input	Data	/Users/stevenlc/Library/CloudStorage/OneDrive-Privat/ICloud filer/Doktorander/Rickard/Artikel 3/Revision art 3/Artikel3_revision.sav
	Active Dataset	DataSet7
	Filter	<none>
	Weight	<none>
	Split File	<none>
	N of Rows in Working Data File	10337
Missing Value Handling	Definition of Missing	User-defined missing values are treated as missing
Syntax		LOGISTIC REGRESSION VARIABLES AUDITRISKinklnyktny /METHOD=ENTER Maltreatment_0to3 /CONTRAST (Maltreatment_0to3)=Indicator(1) /PRINT=CI(95) /CRITERIA=PIN(0.05) POUT(0.10) ITERATE(20) CUT(0.5).
Resources	Processor Time	00:00:00,39
	Elapsed Time	00:00:00,00

Case Processing Summary

Unweighted Cases[a]		N	Percent
Selected Cases	Included in Analysis	8899	86.1
	Missing Cases	1438	13.9
	Total	10337	100.0
Unselected Cases		0	.0
Total		10337	100.0

a. If weight is in effect, see classification table for the total number of cases.

Dependent Variable Encoding

Original Value	Internal Value
1.00	0
2.00	1

Categorical Variables Codings

		Frequency	Parameter coding (1)	(2)	(3)
Maltreatment_0to3	.00	5877	.000	.000	.000
	1.00	1541	1.000	.000	.000
	2.00	717	.000	1.000	.000
	3.00	764	.000	.000	1.000

Block 0: Beginning Block

Classification Table[a,b]

	Observed		Predicted Alkohol riskbruk 1.00	2.00	Percentage Correct
Step 0	Alkohol riskbruk	1.00	7223	0	100.0
		2.00	1676	0	.0
	Overall Percentage				81.2

a. Constant is included in the model.

b. The cut value is .500

Variables in the Equation

		B	S.E.	Wald	df	Sig.	Exp(B)
Step 0	Constant	-1.461	.027	2903.139	1	.000	.232

Variables not in the Equation

			Score	df	Sig.
Step 0	Variables	Maltreatment_0to3	72.463	3	<.001
		Maltreatment_0to3(1)	21.542	1	<.001
		Maltreatment_0to3(2)	4.361	1	.037
		Maltreatment_0to3(3)	30.551	1	<.001
	Overall Statistics		72.463	3	<.001

Block 1: Method = Enter

Omnibus Tests of Model Coefficients

		Chi-square	df	Sig.
Step 1	Step	69.865	3	<.001
	Block	69.865	3	<.001
	Model	69.865	3	<.001

Model Summary

Step	-2 Log likelihood	Cox & Snell R Square	Nagelkerke R Square
1	8540.821[a]	.008	.013

a. Estimation terminated at iteration number 4 because parameter estimates changed by less than .001.

Classification Table[a]

Observed			Predicted: Alkohol riskbruk 1.00	Predicted: Alkohol riskbruk 2.00	Percentage Correct
Step 1	Alkohol riskbruk	1.00	7223	0	100.0
		2.00	1676	0	.0
	Overall Percentage				81.2

a. The cut value is .500

Variables in the Equation

		B	S.E.	Wald	df	Sig.
Step 1[a]	Maltreatment_0to3			71.568	3	<.001
	Maltreatment_0to3(1)	.422	.070	36.393	1	<.001
	Maltreatment_0to3(2)	.349	.097	12.888	1	<.001
	Maltreatment_0to3(3)	.599	.089	44.830	1	<.001
	Constant	-1.629	.035	2137.320	1	.000

Variables in the Equation

		Exp(B)	95% C.I.for EXP(B) Lower	95% C.I.for EXP(B) Upper
Step 1[a]	Maltreatment_0to3			
	Maltreatment_0to3(1)	1.526	1.330	1.750
	Maltreatment_0to3(2)	1.417	1.172	1.714
	Maltreatment_0to3(3)	1.820	1.527	2.168
	Constant	.196		

a. Variable(s) entered on step 1: Maltreatment_0to3.

Logistic Regression

Notes

Output Created		05-OCT-2024 11:47:51
Comments		
Input	Data	/Users/stevenlc/Library/CloudStorage/OneDrive-Privat/ICloud filer/Doktorander/Rickard/Artikel 3/Revision art 3/Artikel3_revision.sav
	Active Dataset	DataSet7
	Filter	<none>
	Weight	<none>
	Split File	<none>
	N of Rows in Working Data File	10337
Missing Value Handling	Definition of Missing	User-defined missing values are treated as missing
Syntax		LOGISTIC REGRESSION VARIABLES AUDITRISKinklnyktny /METHOD=ENTER Maltreatment_0to3 Householddysf_parent Kon barnboendeny utbildningmammapappa_ny fodelselandmammapappa_ny alderskategorier /CONTRAST (Maltreatment_0to3)=Indicator(1) /CONTRAST (Householddysf_parent)=Indicator(1) /CONTRAST (Kon)=Indicator(1) /CONTRAST (barnboendeny)=Indicator(1) /CONTRAST (utbildningmammapappa_ny)=Indicator(1) /CONTRAST (fodelselandmammapappa_ny)=Indicator(1) /CONTRAST (alderskategorier)=Indicator(1) /PRINT=CI(95) /CRITERIA=PIN(0.05) POUT(0.10) ITERATE(20) CUT(0.5).
Resources	Processor Time	00:00:00,43
	Elapsed Time	00:00:01,00

Case Processing Summary

Unweighted Cases[a]		N	Percent
Selected Cases	Included in Analysis	7426	71.8
	Missing Cases	2911	28.2
	Total	10337	100.0
Unselected Cases		0	.0
Total		10337	100.0

a. If weight is in effect, see classification table for the total number of cases.

Dependent Variable Encoding

Original Value	Internal Value
1.00	0
2.00	1

Categorical Variables Codings

			Parameter coding		
		Frequency	(1)	(2)	(3)
Age categories	17-25	895	.000	.000	.000
	26-35	1166	1.000	.000	.000
	36-45	1401	.000	1.000	.000
	46-55	1475	.000	.000	1.000
	56-65	1542	.000	.000	.000
	66-74	947	.000	.000	.000
Maltreatment_0to3	.00	5028	.000	.000	.000
	1.00	1257	1.000	.000	.000
	2.00	571	.000	1.000	.000
	3.00	570	.000	.000	1.000
Parent education	At least one parent high school	3981	.000		
	Both parents below high school	3445	1.000		
Householddysf_parent	.00	5610	.000		
	1.00	1816	1.000		
Sex	Man	3330	.000		
	Kvinna	4096	1.000		
Immigrant status	At least one Nordic parent	6949	.000		
	Both parents born elsewhere	477	1.000		
Residence type	Owned home	5559	.000		
	Rental	1867	1.000		

Categorical Variables Codings

		Parameter coding (4)	Parameter coding (5)
Age categories	17-25	.000	.000
	26-35	.000	.000
	36-45	.000	.000
	46-55	.000	.000
	56-65	1.000	.000
	66-74	.000	1.000
Maltreatment_0to3	.00		
	1.00		
	2.00		
	3.00		
Parent education	At least one parent high school		
	Both parents below high school		
Householddysf_parent	.00		
	1.00		
Sex	Man		
	Kvinna		
Immigrant status	At least one Nordic parent		
	Both parents born elsewhere		
Residence type	Owned home		
	Rental		

Block 0: Beginning Block

Classification Table[a,b]

	Observed		Predicted: Alkohol riskbruk 1.00	Predicted: Alkohol riskbruk 2.00	Percentage Correct
Step 0	Alkohol riskbruk	1.00	6036	0	100.0
		2.00	1390	0	.0
	Overall Percentage				81.3

a. Constant is included in the model.

b. The cut value is .500

Variables in the Equation

		B	S.E.	Wald	df	Sig.	Exp(B)
Step 0	Constant	-1.468	.030	2436.241	1	.000	.230

Variables not in the Equation

			Score	df	Sig.
Step 0	Variables	Maltreatment_0to3	64.183	3	<.001
		Maltreatment_0to3(1)	23.203	1	<.001
		Maltreatment_0to3(2)	4.094	1	.043
		Maltreatment_0to3(3)	23.425	1	<.001
		Householddysf_parent(1)	127.417	1	<.001
		Sex(1)	17.882	1	<.001
		Residence type(1)	.029	1	.866
		Parent education(1)	103.867	1	<.001
		Immigrant status(1)	22.725	1	<.001
		Age categories	384.686	5	<.001
		Age categories(1)	30.692	1	<.001
		Age categories(2)	7.181	1	.007
		Age categories(3)	15.673	1	<.001
		Age categories(4)	36.731	1	<.001
		Age categories(5)	57.827	1	<.001
	Overall Statistics		580.476	13	<.001

Block 1: Method = Enter

Omnibus Tests of Model Coefficients

		Chi-square	df	Sig.
Step 1	Step	551.019	13	<.001
	Block	551.019	13	<.001
	Model	551.019	13	<.001

Model Summary

Step	-2 Log likelihood	Cox & Snell R Square	Nagelkerke R Square
1	6609.253[a]	.072	.116

a. Estimation terminated at iteration number 5 because parameter estimates changed by less than .001.

Classification Table[a]

Observed			Predicted		
			Alkohol riskbruk		Percentage Correct
			1.00	2.00	
Step 1	Alkohol riskbruk	1.00	5940	96	98.4
		2.00	1320	70	5.0
	Overall Percentage				80.9

a. The cut value is .500

Variables in the Equation

		B	S.E.	Wald	df	Sig.
Step 1[a]	Maltreatment_0to3			63.495	3	<.001
	Maltreatment_0to3(1)	.495	.081	37.544	1	<.001
	Maltreatment_0to3(2)	.441	.115	14.597	1	<.001
	Maltreatment_0to3(3)	.668	.113	35.082	1	<.001
	Householddysf_parent(1)	.362	.072	25.120	1	<.001
	Sex(1)	-.392	.063	39.092	1	<.001
	Residence type(1)	.117	.076	2.353	1	.125
	Parent education(1)	-.173	.075	5.296	1	.021
	Immigrant status(1)	-.996	.159	39.385	1	<.001
	Age categories			247.486	5	<.001
	Age categories(1)	-.697	.099	49.480	1	<.001
	Age categories(2)	-1.250	.104	144.631	1	<.001
	Age categories(3)	-1.330	.108	150.261	1	<.001
	Age categories(4)	-1.413	.116	147.462	1	<.001
	Age categories(5)	-1.752	.144	148.801	1	<.001
	Constant	-.411	.084	23.707	1	<.001

Variables in the Equation

		Exp(B)	95% C.I.for EXP(B)	
			Lower	Upper
Step 1[a]	Maltreatment_0to3			
	Maltreatment_0to3(1)	1.641	1.400	1.922
	Maltreatment_0to3(2)	1.554	1.240	1.949
	Maltreatment_0to3(3)	1.951	1.564	2.434
	Householddysf_parent(1)	1.436	1.246	1.654
	Sex(1)	.676	.598	.764
	Residence type(1)	1.124	.968	1.305
	Parent education(1)	.841	.726	.975
	Immigrant status(1)	.369	.271	.504
	Age categories			
	Age categories(1)	.498	.410	.605
	Age categories(2)	.287	.234	.351
	Age categories(3)	.265	.214	.327
	Age categories(4)	.244	.194	.306
	Age categories(5)	.173	.131	.230
	Constant	.663		

a. Variable(s) entered on step 1: Maltreatment_0to3, Householddysf_parent, Sex, Residence type, Parent education, Immigrant status, Age categories.

Logistic Regression

Notes

Output Created		05-OCT-2024 11:47:52
Comments		
Input	Data	/Users/stevenlc/Library/CloudStorage/OneDrive-Privat/ICloud filer/Doktorander/Rickard/Artikel 3/Revision art 3/Artikel3_revision.sav
	Active Dataset	DataSet7
	Filter	<none>
	Weight	<none>
	Split File	<none>
	N of Rows in Working Data File	10337
Missing Value Handling	Definition of Missing	User-defined missing values are treated as missing
Syntax		LOGISTIC REGRESSION VARIABLES AUDITRISKinklnyktny /METHOD=ENTER Lifetimeabuseindex /CONTRAST (Lifetimeabuseindex)=Indicator(1) /PRINT=CI(95) /CRITERIA=PIN(0.05) POUT(0.10) ITERATE(20) CUT(0.5).
Resources	Processor Time	00:00:00,39
	Elapsed Time	00:00:00,00

Case Processing Summary

Unweighted Cases[a]		N	Percent
Selected Cases	Included in Analysis	8699	84.2
	Missing Cases	1638	15.8
	Total	10337	100.0
Unselected Cases		0	.0
Total		10337	100.0

a. If weight is in effect, see classification table for the total number of cases.

Dependent Variable Encoding

Original Value	Internal Value
1.00	0
2.00	1

Categorical Variables Codings

		Frequency	Parameter coding (1)	(2)	(3)	(4)	(5)
Lifetimeabuseindex	.00	4904	.000	.000	.000	.000	.000
	1.00	1033	1.000	.000	.000	.000	.000
	2.00	435	.000	1.000	.000	.000	.000
	3.00	366	.000	.000	1.000	.000	.000
	4.00	853	.000	.000	.000	1.000	.000
	5.00	468	.000	.000	.000	.000	1.000
	6.00	262	.000	.000	.000	.000	.000
	7.00	378	.000	.000	.000	.000	.000

Categorical Variables Codings

		Parameter coding (6)	(7)
Lifetimeabuseindex	.00	.000	.000
	1.00	.000	.000
	2.00	.000	.000
	3.00	.000	.000
	4.00	.000	.000
	5.00	.000	.000
	6.00	1.000	.000
	7.00	.000	1.000

Block 0: Beginning Block

Classification Table[a,b]

Observed			Predicted: Alkohol riskbruk 1.00	2.00	Percentage Correct
Step 0	Alkohol riskbruk	1.00	7067	0	100.0
		2.00	1632	0	.0
	Overall Percentage				81.2

a. Constant is included in the model.

b. The cut value is .500

Variables in the Equation

		B	S.E.	Wald	df	Sig.	Exp(B)
Step 0	Constant	-1.466	.027	2847.964	1	.000	.231

Variables not in the Equation

			Score	df	Sig.
Step 0	Variables	Lifetimeabuseindex	183.953	7	<.001
		Lifetimeabuseindex(1)	.005	1	.946
		Lifetimeabuseindex(2)	.091	1	.763
		Lifetimeabuseindex(3)	1.631	1	.202
		Lifetimeabuseindex(4)	33.817	1	<.001
		Lifetimeabuseindex(5)	55.495	1	<.001
		Lifetimeabuseindex(6)	9.171	1	.002
		Lifetimeabuseindex(7)	38.538	1	<.001
	Overall Statistics		183.953	7	<.001

Block 1: Method = Enter

Omnibus Tests of Model Coefficients

		Chi-square	df	Sig.
Step 1	Step	172.551	7	<.001
	Block	172.551	7	<.001
	Model	172.551	7	<.001

Model Summary

Step	-2 Log likelihood	Cox & Snell R Square	Nagelkerke R Square
1	8226.082[a]	.020	.032

a. Estimation terminated at iteration number 4 because parameter estimates changed by less than .001.

Classification Table[a]

Observed			Predicted: Alkohol riskbruk 1.00	Predicted: Alkohol riskbruk 2.00	Predicted: Percentage Correct
Step 1	Alkohol riskbruk	1.00	7067	0	100.0
		2.00	1632	0	.0
	Overall Percentage				81.2

a. The cut value is .500

Variables in the Equation

		B	S.E.	Wald	df	Sig.
Step 1[a]	Lifetimeabuseindex			177.822	7	<.001
	Lifetimeabuseindex(1)	.289	.089	10.445	1	.001
	Lifetimeabuseindex(2)	.330	.128	6.640	1	.010
	Lifetimeabuseindex(3)	.454	.134	11.477	1	<.001
	Lifetimeabuseindex(4)	.721	.088	67.558	1	<.001
	Lifetimeabuseindex(5)	.999	.107	86.896	1	<.001
	Lifetimeabuseindex(6)	.711	.147	23.553	1	<.001
	Lifetimeabuseindex(7)	.957	.118	65.446	1	<.001
	Constant	-1.760	.040	1902.332	1	.000

Variables in the Equation

		Exp(B)	95% C.I.for EXP(B)	
			Lower	Upper
Step 1[a]	Lifetimeabuseindex			
	Lifetimeabuseindex(1)	1.335	1.120	1.591
	Lifetimeabuseindex(2)	1.391	1.082	1.787
	Lifetimeabuseindex(3)	1.574	1.211	2.046
	Lifetimeabuseindex(4)	2.057	1.732	2.443
	Lifetimeabuseindex(5)	2.714	2.200	3.348
	Lifetimeabuseindex(6)	2.037	1.528	2.715
	Lifetimeabuseindex(7)	2.605	2.066	3.285
	Constant	.172		

a. Variable(s) entered on step 1: Lifetimeabuseindex.

Logistic Regression

Notes

Output Created		05-OCT-2024 11:47:52
Comments		
Input	Data	/Users/stevenlc/Library/CloudStorage/OneDrive-Privat/ICloud filer/Doktorander/Rickard/Artikel 3/Revision art 3/Artikel3_revision.sav
	Active Dataset	DataSet7
	Filter	<none>
	Weight	<none>
	Split File	<none>
	N of Rows in Working Data File	10337
Missing Value Handling	Definition of Missing	User-defined missing values are treated as missing
Syntax		LOGISTIC REGRESSION VARIABLES AUDITRISKinklnyktny<br>/METHOD=ENTER Lifetimeabuseindex Householddysf_parent Kon barnboendeny utbildningmammapappa_ny fodelselandmammapappa_ny alderskategorier<br>/CONTRAST (Lifetimeabuseindex)=Indicator(1)<br>/CONTRAST (Householddysf_parent)=Indicator(1)<br>/CONTRAST (Kon)=Indicator(1)<br>/CONTRAST (barnboendeny)=Indicator(1)<br>/CONTRAST (utbildningmammapappa_ny)=Indicator(1)<br>/CONTRAST (fodelselandmammapappa_ny)=Indicator(1)<br>/CONTRAST (alderskategorier)=Indicator(1)<br>/PRINT=CI(95)<br>/CRITERIA=PIN(0.05) POUT(0.10) ITERATE(20) CUT(0.5).
Resources	Processor Time	00:00:00,46
	Elapsed Time	00:00:00,00

Case Processing Summary

Unweighted Cases[a]		N	Percent
Selected Cases	Included in Analysis	7279	70.4
	Missing Cases	3058	29.6
	Total	10337	100.0
Unselected Cases		0	.0
Total		10337	100.0

a. If weight is in effect, see classification table for the total number of cases.

Dependent Variable Encoding

Original Value	Internal Value
1.00	0
2.00	1

Categorical Variables Codings

			Parameter coding		
		Frequency	(1)	(2)	(3)
Lifetimeabuseindex	.00	4216	.000	.000	.000
	1.00	842	1.000	.000	.000
	2.00	339	.000	1.000	.000
	3.00	264	.000	.000	1.000
	4.00	721	.000	.000	.000
	5.00	388	.000	.000	.000
	6.00	217	.000	.000	.000
	7.00	292	.000	.000	.000
Age categories	17-25	874	.000	.000	.000
	26-35	1146	1.000	.000	.000
	36-45	1374	.000	1.000	.000
	46-55	1443	.000	.000	1.000
	56-65	1514	.000	.000	.000
	66-74	928	.000	.000	.000
Parent education	At least one parent high school	3889	.000		
	Both parents below high school	3390	1.000		
Householddysf_parent	.00	5509	.000		
	1.00	1770	1.000		
Sex	Man	3262	.000		
	Kvinna	4017	1.000		
Immigrant status	At least one Nordic parent	6811	.000		
	Both parents born elsewhere	468	1.000		
Residence type	Owned home	5442	.000		
	Rental	1837	1.000		

Categorical Variables Codings

		Parameter coding (4)	(5)	(6)	(7)
Lifetimeabuseindex	.00	.000	.000	.000	.000
	1.00	.000	.000	.000	.000
	2.00	.000	.000	.000	.000
	3.00	.000	.000	.000	.000
	4.00	1.000	.000	.000	.000
	5.00	.000	1.000	.000	.000
	6.00	.000	.000	1.000	.000
	7.00	.000	.000	.000	1.000
Age categories	17-25	.000	.000		
	26-35	.000	.000		
	36-45	.000	.000		
	46-55	.000	.000		
	56-65	1.000	.000		
	66-74	.000	1.000		
Parent education	At least one parent high school				
	Both parents below high school				
Householddysf_parent	.00				
	1.00				
Sex	Man				
	Kvinna				
Immigrant status	At least one Nordic parent				
	Both parents born elsewhere				
Residence type	Owned home				
	Rental				

Block 0: Beginning Block

Classification Table[a,b]

Observed			Predicted Alkohol riskbruk 1.00	2.00	Percentage Correct
Step 0	Alkohol riskbruk	1.00	5925	0	100.0
		2.00	1354	0	.0
	Overall Percentage				81.4

a. Constant is included in the model.

b. The cut value is .500

Variables in the Equation

		B	S.E.	Wald	df	Sig.	Exp(B)
Step 0	Constant	-1.476	.030	2401.470	1	.000	.229

Variables not in the Equation

			Score	df	Sig.
Step 0	Variables	Lifetimeabuseindex	150.867	7	<.001
		Lifetimeabuseindex(1)	.483	1	.487
		Lifetimeabuseindex(2)	.000	1	.993
		Lifetimeabuseindex(3)	.217	1	.641
		Lifetimeabuseindex(4)	24.296	1	<.001
		Lifetimeabuseindex(5)	44.639	1	<.001
		Lifetimeabuseindex(6)	9.756	1	.002
		Lifetimeabuseindex(7)	35.261	1	<.001
		Householddysf_parent(1)	121.144	1	<.001
		Sex(1)	16.088	1	<.001
		Residence type(1)	.035	1	.851
		Parent education(1)	102.415	1	<.001
		Immigrant status(1)	21.841	1	<.001
		Age categories	382.334	5	<.001
		Age categories(1)	29.639	1	<.001
		Age categories(2)	7.503	1	.006
		Age categories(3)	14.512	1	<.001
		Age categories(4)	34.922	1	<.001
		Age categories(5)	59.798	1	<.001
	Overall Statistics		643.470	17	<.001

Block 1: Method = Enter

Omnibus Tests of Model Coefficients

		Chi-square	df	Sig.
Step 1	Step	610.183	17	<.001
	Block	610.183	17	<.001
	Model	610.183	17	<.001

Model Summary

Step	-2 Log likelihood	Cox & Snell R Square	Nagelkerke R Square
1	6383.366 [a]	.080	.130

a. Estimation terminated at iteration number 5 because parameter estimates changed by less than .001.

Classification Table[a]

Observed			Predicted: Alkohol riskbruk 1.00	Predicted: Alkohol riskbruk 2.00	Percentage Correct
Step 1	Alkohol riskbruk	1.00	5833	92	98.4
		2.00	1258	96	7.1
	Overall Percentage				81.5

a. The cut value is .500

Variables in the Equation

		B	S.E.	Wald	df	Sig.
Step 1[a]	Lifetimeabuseindex			132.726	7	<.001
	Lifetimeabuseindex(1)	.381	.102	14.011	1	<.001
	Lifetimeabuseindex(2)	.338	.155	4.787	1	.029
	Lifetimeabuseindex(3)	.456	.171	7.142	1	.008
	Lifetimeabuseindex(4)	.658	.101	42.807	1	<.001
	Lifetimeabuseindex(5)	1.010	.124	66.473	1	<.001
	Lifetimeabuseindex(6)	.861	.169	25.964	1	<.001
	Lifetimeabuseindex(7)	1.029	.144	51.176	1	<.001
	Householddysf_parent(1)	.343	.074	21.657	1	<.001
	Sex(1)	-.398	.064	38.771	1	<.001
	Residence type(1)	.120	.078	2.378	1	.123
	Parent education(1)	-.147	.076	3.700	1	.054
	Immigrant status(1)	-.980	.161	36.994	1	<.001
	Age categories			251.607	5	<.001
	Age categories(1)	-.745	.101	54.238	1	<.001
	Age categories(2)	-1.326	.106	155.305	1	<.001
	Age categories(3)	-1.383	.111	155.698	1	<.001
	Age categories(4)	-1.427	.118	145.744	1	<.001
	Age categories(5)	-1.778	.147	145.857	1	<.001
	Constant	-.497	.088	32.117	1	<.001

Variables in the Equation

		Exp(B)	95% C.I.for EXP(B) Lower	Upper
Step 1[a]	Lifetimeabuseindex			
	Lifetimeabuseindex(1)	1.464	1.199	1.787
	Lifetimeabuseindex(2)	1.403	1.036	1.900
	Lifetimeabuseindex(3)	1.577	1.129	2.203
	Lifetimeabuseindex(4)	1.931	1.586	2.352
	Lifetimeabuseindex(5)	2.747	2.154	3.502
	Lifetimeabuseindex(6)	2.366	1.699	3.295
	Lifetimeabuseindex(7)	2.798	2.111	3.709
	Householddysf_parent(1)	1.409	1.219	1.627
	Sex(1)	.672	.593	.761
	Residence type(1)	1.127	.968	1.312
	Parent education(1)	.863	.743	1.003
	Immigrant status(1)	.375	.274	.515
	Age categories			
	Age categories(1)	.475	.390	.579
	Age categories(2)	.266	.216	.327
	Age categories(3)	.251	.202	.312
	Age categories(4)	.240	.190	.303
	Age categories(5)	.169	.127	.225
	Constant	.608		

a. Variable(s) entered on step 1: Lifetimeabuseindex, Householddysf_parent, Sex, Residence type, Parent education, Immigrant status, Age categories.

Logistic Regression

Notes

Output Created		05-OCT-2024 11:47:52
Comments		
Input	Data	/Users/stevenlc/Library/CloudStorage/OneDrive-Privat/ICloud filer/Doktorander/Rickard/Artikel 3/Revision art 3/Artikel3_revision.sav
	Active Dataset	DataSet7
	Filter	<none>
	Weight	<none>
	Split File	<none>
	N of Rows in Working Data File	10337
Missing Value Handling	Definition of Missing	User-defined missing values are treated as missing
Syntax		LOGISTIC REGRESSION VARIABLES Drogmissbruk /METHOD=ENTER Maltreatment_0to3 /CONTRAST (Maltreatment_0to3)=Indicator(1) /PRINT=CI(95) /CRITERIA=PIN(0.05) POUT(0.10) ITERATE(20) CUT(0.5).
Resources	Processor Time	00:00:00,40
	Elapsed Time	00:00:01,00

Case Processing Summary

Unweighted Cases[a]		N	Percent
Selected Cases	Included in Analysis	9169	88.7
	Missing Cases	1168	11.3
	Total	10337	100.0
Unselected Cases		0	.0
Total		10337	100.0

a. If weight is in effect, see classification table for the total number of cases.

Dependent Variable Encoding

Original Value	Internal Value
1.00	0
2.00	1

Categorical Variables Codings

		Frequency	Parameter coding (1)	(2)	(3)
Maltreatment_0to3	.00	6043	.000	.000	.000
	1.00	1589	1.000	.000	.000
	2.00	744	.000	1.000	.000
	3.00	793	.000	.000	1.000

Block 0: Beginning Block

Classification Table[a,b]

Observed			Predicted Drogmissbruk 1.00	2.00	Percentage Correct
Step 0	Drogmissbruk	1.00	9128	0	100.0
		2.00	41	0	.0
	Overall Percentage				99.6

a. Constant is included in the model.

b. The cut value is .500

Variables in the Equation

		B	S.E.	Wald	df	Sig.	Exp(B)
Step 0	Constant	-5.406	.157	1192.653	1	<.001	.004

Variables not in the Equation

			Score	df	Sig.
Step 0	Variables	Maltreatment_0to3	32.165	3	<.001
		Maltreatment_0to3(1)	.209	1	.648
		Maltreatment_0to3(2)	2.348	1	.125
		Maltreatment_0to3(3)	27.716	1	<.001
	Overall Statistics		32.165	3	<.001

Block 1: Method = Enter

Omnibus Tests of Model Coefficients

		Chi-square	df	Sig.
Step 1	Step	22.200	3	<.001
	Block	22.200	3	<.001
	Model	22.200	3	<.001

Model Summary

Step	-2 Log likelihood	Cox & Snell R Square	Nagelkerke R Square
1	503.238[a]	.002	.043

a. Estimation terminated at iteration number 9 because parameter estimates changed by less than .001.

Classification Table[a]

Observed			Predicted Drogmissbruk 1.00	Predicted Drogmissbruk 2.00	Predicted Percentage Correct
Step 1	Drogmissbruk	1.00	9128	0	100.0
		2.00	41	0	.0
	Overall Percentage				99.6

a. The cut value is .500

Variables in the Equation

		B	S.E.	Wald	df	Sig.
Step 1[a]	Maltreatment_0to3			25.748	3	<.001
	Maltreatment_0to3(1)	.356	.480	.551	1	.458
	Maltreatment_0to3(2)	1.119	.480	5.430	1	.020
	Maltreatment_0to3(3)	1.837	.375	23.957	1	<.001
	Constant	-5.931	.250	561.417	1	<.001

Variables in the Equation

		Exp(B)	95% C.I.for EXP(B) Lower	95% C.I.for EXP(B) Upper
Step 1[a]	Maltreatment_0to3			
	Maltreatment_0to3(1)	1.428	.558	3.655
	Maltreatment_0to3(2)	3.062	1.195	7.851
	Maltreatment_0to3(3)	6.278	3.009	13.101
	Constant	.003		

a. Variable(s) entered on step 1: Maltreatment_0to3.

Logistic Regression

Notes

Output Created		05-OCT-2024 11:47:53
Comments		
Input	Data	/Users/stevenlc/Library/CloudStorage/OneDrive-Privat/ICloud filer/Doktorander/Rickard/Artikel 3/Revision art 3/Artikel3_revision.sav
	Active Dataset	DataSet7
	Filter	<none>
	Weight	<none>
	Split File	<none>
	N of Rows in Working Data File	10337
Missing Value Handling	Definition of Missing	User-defined missing values are treated as missing
Syntax		LOGISTIC REGRESSION VARIABLES Drogmissbruk /METHOD=ENTER Maltreatment_0to3 Householddysf_parent Kon barnboendeny utbildningmammapappa_ny fodelselandmammapappa_ny alderskategorier /CONTRAST (Maltreatment_0to3)=Indicator(1) /CONTRAST (Householddysf_parent)=Indicator(1) /CONTRAST (Kon)=Indicator(1) /CONTRAST (barnboendeny)=Indicator(1) /CONTRAST (utbildningmammapappa_ny)=Indicator(1) /CONTRAST (fodelselandmammapappa_ny)=Indicator(1) /CONTRAST (alderskategorier)=Indicator(1) /PRINT=CI(95) /CRITERIA=PIN(0.05) POUT(0.10) ITERATE(20) CUT(0.5).
Resources	Processor Time	00:00:00,47
	Elapsed Time	00:00:00,00

Case Processing Summary

Unweighted Cases[a]		N	Percent
Selected Cases	Included in Analysis	7629	73.8
	Missing Cases	2708	26.2
	Total	10337	100.0
Unselected Cases		0	.0
Total		10337	100.0

a. If weight is in effect, see classification table for the total number of cases.

Dependent Variable Encoding

Original Value	Internal Value
1.00	0
2.00	1

Categorical Variables Codings

		Frequency	Parameter coding (1)	(2)	(3)
Age categories	17-25	911	.000	.000	.000
	26-35	1194	1.000	.000	.000
	36-45	1430	.000	1.000	.000
	46-55	1512	.000	.000	1.000
	56-65	1592	.000	.000	.000
	66-74	990	.000	.000	.000
Maltreatment_0to3	.00	5160	.000	.000	.000
	1.00	1292	1.000	.000	.000
	2.00	590	.000	1.000	.000
	3.00	587	.000	.000	1.000
Parent education	At least one parent high school	4067	.000		
	Both parents below high school	3562	1.000		
Householddysf_parent	.00	5768	.000		
	1.00	1861	1.000		
Sex	Man	3417	.000		
	Kvinna	4212	1.000		
Immigrant status	At least one Nordic parent	7143	.000		
	Both parents born elsewhere	486	1.000		
Residence type	Owned home	5713	.000		
	Rental	1916	1.000		

Categorical Variables Codings

		Parameter coding (4)	Parameter coding (5)
Age categories	17-25	.000	.000
	26-35	.000	.000
	36-45	.000	.000
	46-55	.000	.000
	56-65	1.000	.000
	66-74	.000	1.000
Maltreatment_0to3	.00		
	1.00		
	2.00		
	3.00		
Parent education	At least one parent high school		
	Both parents below high school		
Householddysf_parent	.00		
	1.00		
Sex	Man		
	Kvinna		
Immigrant status	At least one Nordic parent		
	Both parents born elsewhere		
Residence type	Owned home		
	Rental		

Block 0: Beginning Block

Classification Table[a,b]

Observed			Predicted Drogmissbruk 1.00	Predicted Drogmissbruk 2.00	Percentage Correct
Step 0	Drogmissbruk	1.00	7600	0	100.0
		2.00	29	0	.0
	Overall Percentage				99.6

a. Constant is included in the model.

b. The cut value is .500

Variables in the Equation

		B	S.E.	Wald	df	Sig.	Exp(B)
Step 0	Constant	-5.569	.186	895.854	1	<.001	.004

Variables not in the Equation

			Score	df	Sig.
Step 0	Variables	Maltreatment_0to3	16.042	3	.001
		Maltreatment_0to3(1)	.204	1	.651
		Maltreatment_0to3(2)	3.688	1	.055
		Maltreatment_0to3(3)	11.083	1	<.001
		Householddysf_parent(1)	11.790	1	<.001
		Sex(1)	2.252	1	.133
		Residence type(1)	10.960	1	<.001
		Parent education(1)	.897	1	.343
		Immigrant status(1)	.014	1	.907
		Age categories	5.298	5	.381
		Age categories(1)	1.588	1	.208
		Age categories(2)	.469	1	.494
		Age categories(3)	.014	1	.906
		Age categories(4)	1.952	1	.162
		Age categories(5)	.179	1	.673
	Overall Statistics		38.959	13	<.001

Block 1: Method = Enter

Omnibus Tests of Model Coefficients

		Chi-square	df	Sig.
Step 1	Step	32.469	13	.002
	Block	32.469	13	.002
	Model	32.469	13	.002

Model Summary

Step	-2 Log likelihood	Cox & Snell R Square	Nagelkerke R Square
1	348.620[a]	.004	.087

a. Estimation terminated at iteration number 9 because parameter estimates changed by less than .001.

Classification Table[a]

			Predicted		
			Drogmissbruk		Percentage Correct
	Observed		1.00	2.00	
Step 1	Drogmissbruk	1.00	7600	0	100.0
		2.00	29	0	.0
	Overall Percentage				99.6

a. The cut value is .500

Variables in the Equation

		B	S.E.	Wald	df	Sig.
Step 1[a]	Maltreatment_0to3			7.598	3	.055
	Maltreatment_0to3(1)	.084	.579	.021	1	.884
	Maltreatment_0to3(2)	1.042	.547	3.625	1	.057
	Maltreatment_0to3(3)	1.234	.519	5.659	1	.017
	Householddysf_parent(1)	.539	.427	1.590	1	.207
	Sex(1)	-.748	.382	3.820	1	.051
	Residence type(1)	1.132	.402	7.916	1	.005
	Parent education(1)	-.182	.445	.166	1	.683
	Immigrant status(1)	-.423	.755	.314	1	.575
	Age categories			4.818	5	.438
	Age categories(1)	-.100	.569	.031	1	.860
	Age categories(2)	-.862	.664	1.688	1	.194
	Age categories(3)	-.636	.626	1.032	1	.310
	Age categories(4)	-1.439	.775	3.443	1	.064
	Age categories(5)	-.808	.797	1.028	1	.311
	Constant	-5.443	.532	104.717	1	<.001

Variables in the Equation

		Exp(B)	95% C.I.for EXP(B)	
			Lower	Upper
Step 1[a]	Maltreatment_0to3			
	Maltreatment_0to3(1)	1.088	.350	3.382
	Maltreatment_0to3(2)	2.834	.970	8.279
	Maltreatment_0to3(3)	3.436	1.243	9.499
	Householddysf_parent(1)	1.714	.742	3.958
	Sex(1)	.474	.224	1.002
	Residence type(1)	3.102	1.410	6.826
	Parent education(1)	.834	.348	1.996
	Immigrant status(1)	.655	.149	2.876
	Age categories			
	Age categories(1)	.905	.297	2.759
	Age categories(2)	.422	.115	1.550
	Age categories(3)	.529	.155	1.805
	Age categories(4)	.237	.052	1.084
	Age categories(5)	.446	.093	2.126
	Constant	.004		

a. Variable(s) entered on step 1: Maltreatment_0to3, Householddysf_parent, Sex, Residence type, Parent education, Immigrant status, Age categories.

Logistic Regression

Notes

Output Created		05-OCT-2024 11:47:53
Comments		
Input	Data	/Users/stevenlc/Library/CloudStorage/OneDrive-Privat/ICloud filer/Doktorander/Rickard/Artikel 3/Revision art 3/Artikel3_revision.sav
	Active Dataset	DataSet7
	Filter	<none>
	Weight	<none>
	Split File	<none>
	N of Rows in Working Data File	10337
Missing Value Handling	Definition of Missing	User-defined missing values are treated as missing
Syntax		LOGISTIC REGRESSION VARIABLES Drogmissbruk<br>/METHOD=ENTER Lifetimeabuseindex<br>/CONTRAST (Lifetimeabuseindex)=Indicator(1)<br>/PRINT=CI(95)<br>/CRITERIA=PIN(0.05) POUT(0.10) ITERATE(20) CUT(0.5).
Resources	Processor Time	00:00:00,42
	Elapsed Time	00:00:01,00

Case Processing Summary

Unweighted Cases[a]		N	Percent
Selected Cases	Included in Analysis	8951	86.6
	Missing Cases	1386	13.4
	Total	10337	100.0
Unselected Cases		0	.0
Total		10337	100.0

a. If weight is in effect, see classification table for the total number of cases.

Dependent Variable Encoding

Original Value	Internal Value
1.00	0
2.00	1

Categorical Variables Codings

		Frequency	Parameter coding (1)	(2)	(3)	(4)	(5)
Lifetimeabuseindex	.00	5038	.000	.000	.000	.000	.000
	1.00	1062	1.000	.000	.000	.000	.000
	2.00	454	.000	1.000	.000	.000	.000
	3.00	377	.000	.000	1.000	.000	.000
	4.00	875	.000	.000	.000	1.000	.000
	5.00	483	.000	.000	.000	.000	1.000
	6.00	269	.000	.000	.000	.000	.000
	7.00	393	.000	.000	.000	.000	.000

Categorical Variables Codings

		Parameter coding (6)	(7)
Lifetimeabuseindex	.00	.000	.000
	1.00	.000	.000
	2.00	.000	.000
	3.00	.000	.000
	4.00	.000	.000
	5.00	.000	.000
	6.00	1.000	.000
	7.00	.000	1.000

Block 0: Beginning Block

Classification Table[a,b]

Observed			Predicted: Drogmissbruk 1.00	Drogmissbruk 2.00	Percentage Correct
Step 0	Drogmissbruk	1.00	8911	0	100.0
		2.00	40	0	.0
	Overall Percentage				99.6

a. Constant is included in the model.

b. The cut value is .500

Variables in the Equation

		B	S.E.	Wald	df	Sig.	Exp(B)
Step 0	Constant	-5.406	.158	1163.839	1	<.001	.004

Variables not in the Equation

			Score	df	Sig.
Step 0	Variables	Lifetimeabuseindex	56.652	7	<.001
		Lifetimeabuseindex(1)	.732	1	.392
		Lifetimeabuseindex(2)	.552	1	.457
		Lifetimeabuseindex(3)	.062	1	.804
		Lifetimeabuseindex(4)	1.039	1	.308
		Lifetimeabuseindex(5)	.348	1	.555
		Lifetimeabuseindex(6)	12.426	1	<.001
		Lifetimeabuseindex(7)	40.655	1	<.001
	Overall Statistics		56.652	7	<.001

Block 1: Method = Enter

Omnibus Tests of Model Coefficients

		Chi-square	df	Sig.
Step 1	Step	31.808	7	<.001
	Block	31.808	7	<.001
	Model	31.808	7	<.001

Model Summary

Step	-2 Log likelihood	Cox & Snell R Square	Nagelkerke R Square
1	480.864[a]	.004	.064

a. Estimation terminated at iteration number 9 because parameter estimates changed by less than .001.

Classification Table[a]

			Predicted		
			Drogmissbruk		Percentage Correct
	Observed		1.00	2.00	
Step 1	Drogmissbruk	1.00	8911	0	100.0
		2.00	40	0	.0
	Overall Percentage				99.6

a. The cut value is .500

Variables in the Equation

		B	S.E.	Wald	df	Sig.
Step 1[a]	Lifetimeabuseindex			39.933	7	<.001
	Lifetimeabuseindex(1)	.016	.637	.001	1	.979
	Lifetimeabuseindex(2)	-.233	1.036	.051	1	.822
	Lifetimeabuseindex(3)	.649	.758	.734	1	.392
	Lifetimeabuseindex(4)	-.196	.757	.067	1	.796
	Lifetimeabuseindex(5)	.808	.638	1.603	1	.205
	Lifetimeabuseindex(6)	1.916	.525	13.335	1	<.001
	Lifetimeabuseindex(7)	2.237	.417	28.732	1	<.001
	Constant	-5.883	.268	483.177	1	<.001

Variables in the Equation

			95% C.I.for EXP(B)	
		Exp(B)	Lower	Upper
Step 1[a]	Lifetimeabuseindex			
	Lifetimeabuseindex(1)	1.017	.292	3.544
	Lifetimeabuseindex(2)	.792	.104	6.038
	Lifetimeabuseindex(3)	1.914	.433	8.452
	Lifetimeabuseindex(4)	.822	.187	3.624
	Lifetimeabuseindex(5)	2.243	.642	7.832
	Lifetimeabuseindex(6)	6.797	2.430	19.011
	Lifetimeabuseindex(7)	9.370	4.134	21.234
	Constant	.003		

a. Variable(s) entered on step 1: Lifetimeabuseindex.

Logistic Regression

Notes

Output Created		05-OCT-2024 11:47:54
Comments		
Input	Data	/Users/stevenlc/Library/CloudStorage/OneDrive-Privat/ICloud filer/Doktorander/Rickard/Artikel 3/Revision art 3/Artikel3_revision.sav
	Active Dataset	DataSet7
	Filter	<none>
	Weight	<none>
	Split File	<none>
	N of Rows in Working Data File	10337
Missing Value Handling	Definition of Missing	User-defined missing values are treated as missing
Syntax		LOGISTIC REGRESSION VARIABLES Drogmissbruk /METHOD=ENTER Lifetimeabuseindex Householddysf_parent Kon barnboendeny utbildningmammapappa_ny fodelselandmammapappa_ny alderskategorier /CONTRAST (Lifetimeabuseindex)=Indicator(1) /CONTRAST (Householddysf_parent)=Indicator(1) /CONTRAST (Kon)=Indicator(1) /CONTRAST (barnboendeny)=Indicator(1) /CONTRAST (utbildningmammapappa_ny)=Indicator(1) /CONTRAST (fodelselandmammapappa_ny)=Indicator(1) /CONTRAST (alderskategorier)=Indicator(1) /PRINT=CI(95) /CRITERIA=PIN(0.05) POUT(0.10) ITERATE(20) CUT(0.5).
Resources	Processor Time	00:00:00,50
	Elapsed Time	00:00:00,00

Case Processing Summary

Unweighted Cases[a]		N	Percent
Selected Cases	Included in Analysis	7471	72.3
	Missing Cases	2866	27.7
	Total	10337	100.0
Unselected Cases		0	.0
Total		10337	100.0

a. If weight is in effect, see classification table for the total number of cases.

Dependent Variable Encoding

Original Value	Internal Value
1.00	0
2.00	1

Categorical Variables Codings

			Parameter coding		
		Frequency	(1)	(2)	(3)
Lifetimeabuseindex	.00	4321	.000	.000	.000
	1.00	865	1.000	.000	.000
	2.00	353	.000	1.000	.000
	3.00	273	.000	.000	1.000
	4.00	739	.000	.000	.000
	5.00	399	.000	.000	.000
	6.00	222	.000	.000	.000
	7.00	299	.000	.000	.000
Age categories	17-25	888	.000	.000	.000
	26-35	1171	1.000	.000	.000
	36-45	1403	.000	1.000	.000
	46-55	1480	.000	.000	1.000
	56-65	1561	.000	.000	.000
	66-74	968	.000	.000	.000
Parent education	At least one parent high school	3968	.000		
	Both parents below high school	3503	1.000		
Householddysf_parent	.00	5656	.000		
	1.00	1815	1.000		
Sex	Man	3341	.000		
	Kvinna	4130	1.000		
Immigrant status	At least one Nordic parent	6995	.000		
	Both parents born elsewhere	476	1.000		
Residence type	Owned home	5588	.000		
	Rental	1883	1.000		

Categorical Variables Codings

		Parameter coding (4)	(5)	(6)	(7)
Lifetimeabuseindex	.00	.000	.000	.000	.000
	1.00	.000	.000	.000	.000
	2.00	.000	.000	.000	.000
	3.00	.000	.000	.000	.000
	4.00	1.000	.000	.000	.000
	5.00	.000	1.000	.000	.000
	6.00	.000	.000	1.000	.000
	7.00	.000	.000	.000	1.000
Age categories	17-25	.000	.000		
	26-35	.000	.000		
	36-45	.000	.000		
	46-55	.000	.000		
	56-65	1.000	.000		
	66-74	.000	1.000		
Parent education	At least one parent high school				
	Both parents below high school				
Householddysf_parent	.00				
	1.00				
Sex	Man				
	Kvinna				
Immigrant status	At least one Nordic parent				
	Both parents born elsewhere				
Residence type	Owned home				
	Rental				

Block 0: Beginning Block

Classification Table[a,b]

	Observed		Predicted Drogmissbruk 1.00	2.00	Percentage Correct
Step 0	Drogmissbruk	1.00	7443	0	100.0
		2.00	28	0	.0
	Overall Percentage				99.6

a. Constant is included in the model.

b. The cut value is .500

Variables in the Equation

		B	S.E.	Wald	df	Sig.	Exp(B)
Step 0	Constant	-5.583	.189	869.431	1	<.001	.004

Variables not in the Equation

			Score	df	Sig.
Step 0	Variables	Lifetimeabuseindex	28.204	7	<.001
		Lifetimeabuseindex(1)	.540	1	.462
		Lifetimeabuseindex(2)	.083	1	.773
		Lifetimeabuseindex(3)	.001	1	.981
		Lifetimeabuseindex(4)	.238	1	.625
		Lifetimeabuseindex(5)	.181	1	.671
		Lifetimeabuseindex(6)	12.479	1	<.001
		Lifetimeabuseindex(7)	14.043	1	<.001
		Householddysf_parent(1)	13.099	1	<.001
		Sex(1)	1.755	1	.185
		Residence type(1)	11.997	1	<.001
		Parent education(1)	.652	1	.419
		Immigrant status(1)	.028	1	.867
		Age categories	6.705	5	.244
		Age categories(1)	1.849	1	.174
		Age categories(2)	.372	1	.542
		Age categories(3)	.046	1	.830
		Age categories(4)	3.215	1	.073
		Age categories(5)	.125	1	.723
	Overall Statistics		53.841	17	<.001

Block 1: Method = Enter

Omnibus Tests of Model Coefficients

		Chi-square	df	Sig.
Step 1	Step	39.706	17	.001
	Block	39.706	17	.001
	Model	39.706	17	.001

Model Summary

Step	-2 Log likelihood	Cox & Snell R Square	Nagelkerke R Square
1	329.038[a]	.005	.110

a. Estimation terminated at iteration number 10 because parameter estimates changed by less than .001.

Classification Table[a]

Observed			Predicted: Drogmissbruk 1.00	Predicted: Drogmissbruk 2.00	Percentage Correct
Step 1	Drogmissbruk	1.00	7443	0	100.0
		2.00	28	0	.0
	Overall Percentage				99.6

a. The cut value is .500

Variables in the Equation

		B	S.E.	Wald	df	Sig.
Step 1[a]	Lifetimeabuseindex			13.492	7	.061
	Lifetimeabuseindex(1)	-.248	.774	.103	1	.749
	Lifetimeabuseindex(2)	-.129	1.058	.015	1	.903
	Lifetimeabuseindex(3)	-.003	1.070	.000	1	.998
	Lifetimeabuseindex(4)	-.012	.774	.000	1	.988
	Lifetimeabuseindex(5)	.527	.781	.455	1	.500
	Lifetimeabuseindex(6)	1.735	.613	8.018	1	.005
	Lifetimeabuseindex(7)	1.442	.594	5.895	1	.015
	Householddysf_parent(1)	.647	.437	2.197	1	.138
	Sex(1)	-.767	.392	3.827	1	.050
	Residence type(1)	1.203	.412	8.534	1	.003
	Parent education(1)	.003	.449	.000	1	.995
	Immigrant status(1)	-.446	.767	.339	1	.561
	Age categories			5.693	5	.337
	Age categories(1)	-.204	.573	.127	1	.721
	Age categories(2)	-.939	.668	1.972	1	.160
	Age categories(3)	-.718	.631	1.296	1	.255
	Age categories(4)	-1.871	.883	4.491	1	.034
	Age categories(5)	-.849	.802	1.121	1	.290
	Constant	-5.455	.551	98.027	1	<.001

Variables in the Equation

		Exp(B)	95% C.I.for EXP(B) Lower	Upper
Step 1[a]	Lifetimeabuseindex			
	Lifetimeabuseindex(1)	.780	.171	3.560
	Lifetimeabuseindex(2)	.879	.111	6.992
	Lifetimeabuseindex(3)	.997	.123	8.112
	Lifetimeabuseindex(4)	.988	.217	4.501
	Lifetimeabuseindex(5)	1.693	.366	7.824
	Lifetimeabuseindex(6)	5.668	1.706	18.832
	Lifetimeabuseindex(7)	4.231	1.321	13.553
	Householddysf_parent(1)	1.910	.812	4.493
	Sex(1)	.464	.215	1.001
	Residence type(1)	3.329	1.485	7.458
	Parent education(1)	1.003	.416	2.418
	Immigrant status(1)	.640	.142	2.877
	Age categories			
	Age categories(1)	.815	.265	2.505
	Age categories(2)	.391	.106	1.450
	Age categories(3)	.487	.141	1.680
	Age categories(4)	.154	.027	.869
	Age categories(5)	.428	.089	2.060
	Constant	.004		

a. Variable(s) entered on step 1: Lifetimeabuseindex, Householddysf_parent, Sex, Residence type, Parent education, Immigrant status, Age categories.
